# Design, synthesis, anti-proliferative evaluation, docking, and MD simulations studies of new thiazolidine-2,4-diones targeting VEGFR-2 and apoptosis pathway

**DOI:** 10.1371/journal.pone.0272362

**Published:** 2022-09-23

**Authors:** Mohammed S. Taghour, Hazem Elkady, Wagdy M. Eldehna, Nehal El-Deeb, Ahmed M. Kenawy, Eslam B. Elkaeed, Bshra A. Alsfouk, Mohamed S. Alesawy, Dalal Z. Husein, Ahmed M. Metwaly, Ibrahim H. Eissa

**Affiliations:** 1 Pharmaceutical Medicinal Chemistry & Drug Design Department, Faculty of Pharmacy (Boys), Al-Azhar University, Cairo, Egypt; 2 Department of Pharmaceutical Chemistry, Faculty of Pharmacy, Kafrelsheikh University, Kafrelsheikh, Egypt; 3 Biopharmaceutical Products Research Department, Genetic Engineering and Biotechnology Research Institute, City of Scientific Research and Technological Applications (SRTA-City), Alexandria, Egypt; 4 Pharmaceutical and Fermentation Industries Development Center, City of Scientific Research and Technological Applications (SRTA City), Alexandria, Egypt; 5 Nucleic Acids Research Department, Genetic Engineering and Biotechnology Research Institute, City of Scientific Research and Technological Applications (SRTA-City), Alexandria, Egypt; 6 Department of Pharmaceutical Sciences, College of Pharmacy, AlMaarefa University, Riyadh, Saudi Arabia; 7 Department of Pharmaceutical Sciences, College of Pharmacy, Princess Nourah Bint Abdulrahman University, Riyadh, Saudi Arabia; 8 Chemistry Department, Faculty of Science, New Valley University, El-Kharja, Egypt; 9 Pharmacognosy and Medicinal Plants Department, Faculty of Pharmacy (Boys), Al-Azhar University, Cairo, Egypt; Aligarh Muslim University, INDIA

## Abstract

We report herein, the design and synthesis of thiazolidine-2,4-diones derivatives as new inhibitors for VEGFR-2. The designed members were assessed for their *in vitro* anticancer activity against four cancer cell lines; A549, Caco-2, HepG-2 and MDA-MB-231. Compound **14a** showed the most potent effects against Caco-2, and HepG-2 cell lines (IC_50_ = of 1.5 and 31.5 μM, respectively). Next, the *in vitro* VEGFR-2 inhibitory activity, safety profiles and selectivity indices were examined for all the synthesized members against the normal Vero cell line. Compound **14a** (the safest member against Caco-2 cell line) was further investigated for its ability to inhibit Caco-2 cells migration and healing. Moreover, the apoptotic induction of compound **14a** against Caco-2 cell line was investigated by assessing against four apoptotic genes (Bcl2, Bcl-xl, TGF, and Survivin). The results revealed that compound **14a** can exert apoptosis through significant reduction of Bcl2, Survivin, and TGF gene expression levels. Finally, deep computational studies including molecular docking, ADMET, toxicity studies, and MD simulation were carried out. Also, the DFT calculations were performed and discussed, and the results confirmed the inhibitory reactivity of **14a** against VEGFR-2. Compound **14a** is expected to be used as a potential lead in the development of new VEGFR-2 inhibitors with increased potency.

## 1. Introduction

Tumor development and reproduction were linked to increased vascularity (angiogenesis) in cancer cells, so the anti-angiogenesis mechanism was considered one of the potential strategies to fight cancer [1]. Vascular endothelial growth factor (VEGF) pathway was identified as a key regulator of angiogenesis. This fact was utilized in the discovery of outstanding numerous chemotherapeutic agents [2, 3]. Vascular endothelial growth factor receptors (VEGFRs) is the receptor of VEGF and include three subtypes (VEGFR-1, VEGFR-2, and VEGFR-3) [4].

The VEGFR-2 subtype is the most critical regulator of the angiogenesis process that plays a substantial role in the dissolution, migration, and proliferation of endothelial cells of cancer [5]. VEGFR-2 exerts its effect in cancer cells through binding to VEGF to boost the autophosphorylation process resulting in the motivation of a downstream signaling cascade that is essential for endothelial cell propagation and angiogenesis [6]. As a result, blocking the VEGF / VEGFR-2 system is a promising approach for the development of an anti-angiogenic therapy for slowing cancer growth [7, 8]. Furthermore, the antitumor effect of VEGFR-2 inhibitors have been enhanced by its ability to induce apoptosis [9–12].

**Fig 1:**
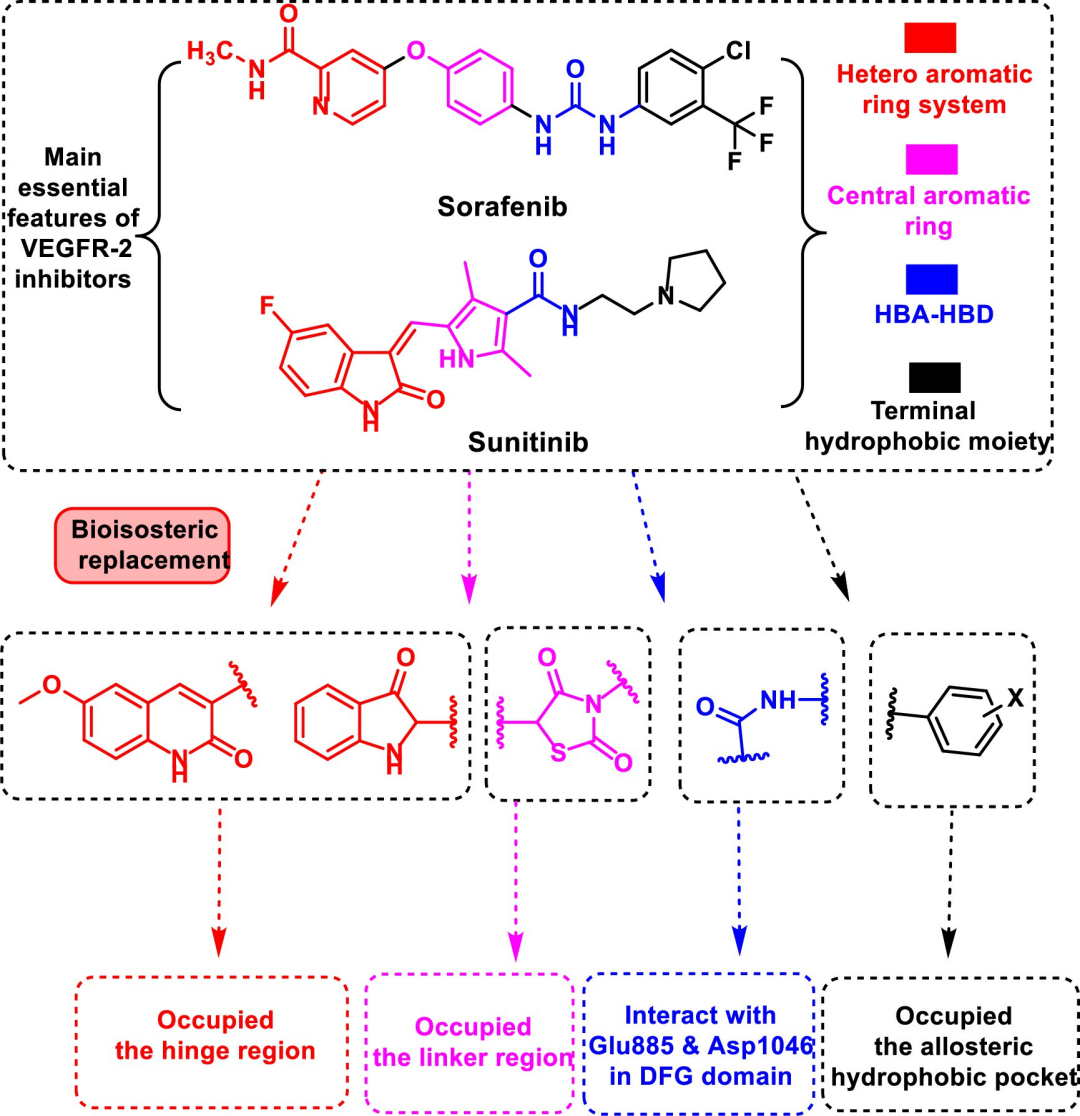
The design rationale of proposed VEGFR-2 inhibitors.

Because of the nature of their large hydrophobic binding site, VEGFR-2 inhibitors have a wide range of structures [7]. However, the crystal structures of the two illustrious VEGFR-2 inhibitors (sorafenib and sunitinib) reveal common key interaction features that are essential for good fitting against VEGFR-2 **(Fig 1)**. These features include primarily a flat heteroaromatic ring system for interaction with the hinge region including the focal amino acid Cys919 [8]. The second feature is a central linker to provide many π-π interactions with Phe1047, Val916, Val848, and Cys1045 in the linker region [9]. The third feature includes a pharmacophore moiety which forms many hydrogen-bonds with the two key amino acids (Glu885 and Asp1046) in the DFG motif. The fourth feature includes hydrophobic moieties that extend to occupy the back hydrophobic pocket [10]. Chemoinformatics (*in silico* techniques) was used as an efficient approach in drug discovery with the advantage of saving time, effort, and costs [11–13]. This includes molecular docking [14, 15], ADMET assessment [15] and MD simulation techniques [14, 16].

In view of the above-mentioned findings and through our trip in the discovery of novel anticancer agents [17–20] especially VEGFR-2 inhibitors [21–28], our research group has paid much attention to develop a new series of anti-angiogenic candidates possessing the main features of sorafenib and sunitinib. Consequently, we used different bioisosteric moieties to occupy the main four regions of VEGFR-2 active pocket. In detail, for the hinge region, 3-oxoindoline and 2-oxo-1,2-dihydroquinolin were used as heteroaromatic moieties. The spacer (thiazolidine-2,4-dione) and pharmacophore (amide) moieties were targeted to occupy the gatekeeper and the DFG-motif regions, respectively. At last, the allosteric pocket was targeted by different aromatic structures (**Fig 1)**. Moreover, all targeted products were subjected to deep biological studies including *in vitro* cytotoxic activities and VEGFR-2 inhibitory activity. Furthermore, the most promising member was further investigated for its apoptotic induction by assessing the gene expression of four genes (Bcl2, Bcl-xl, TGF, and Survivin). Further *in silico* studies including molecular docking, MD simulations, MM-PBSA, ADMET, and toxicity were conducted to correlate the affinity of our compounds against VEGFR-2.

## 2. Results and discussion

### 2.1. Chemistry

**Scheme 1:**
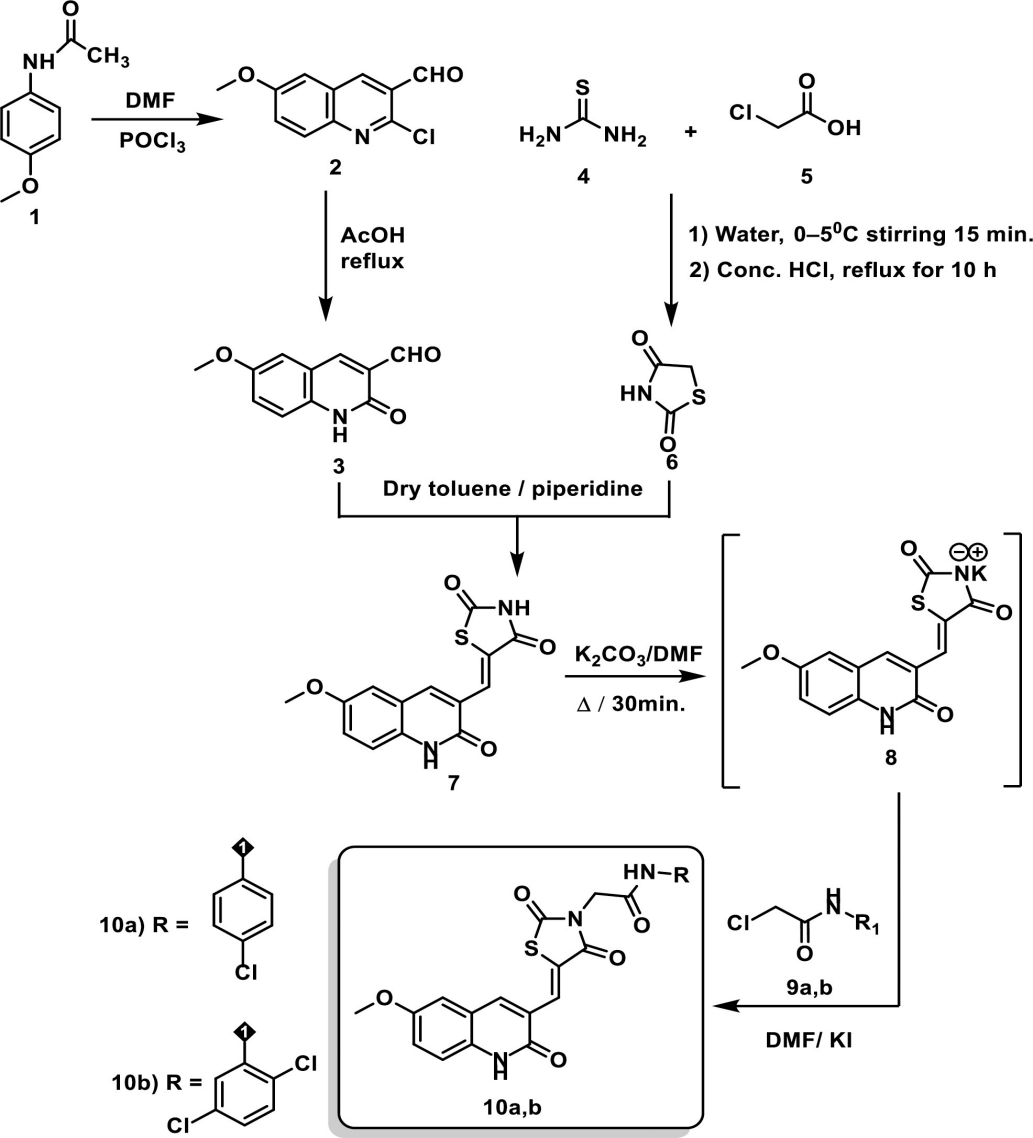
Synthesis of compounds 10a-b.

**Scheme 2:**
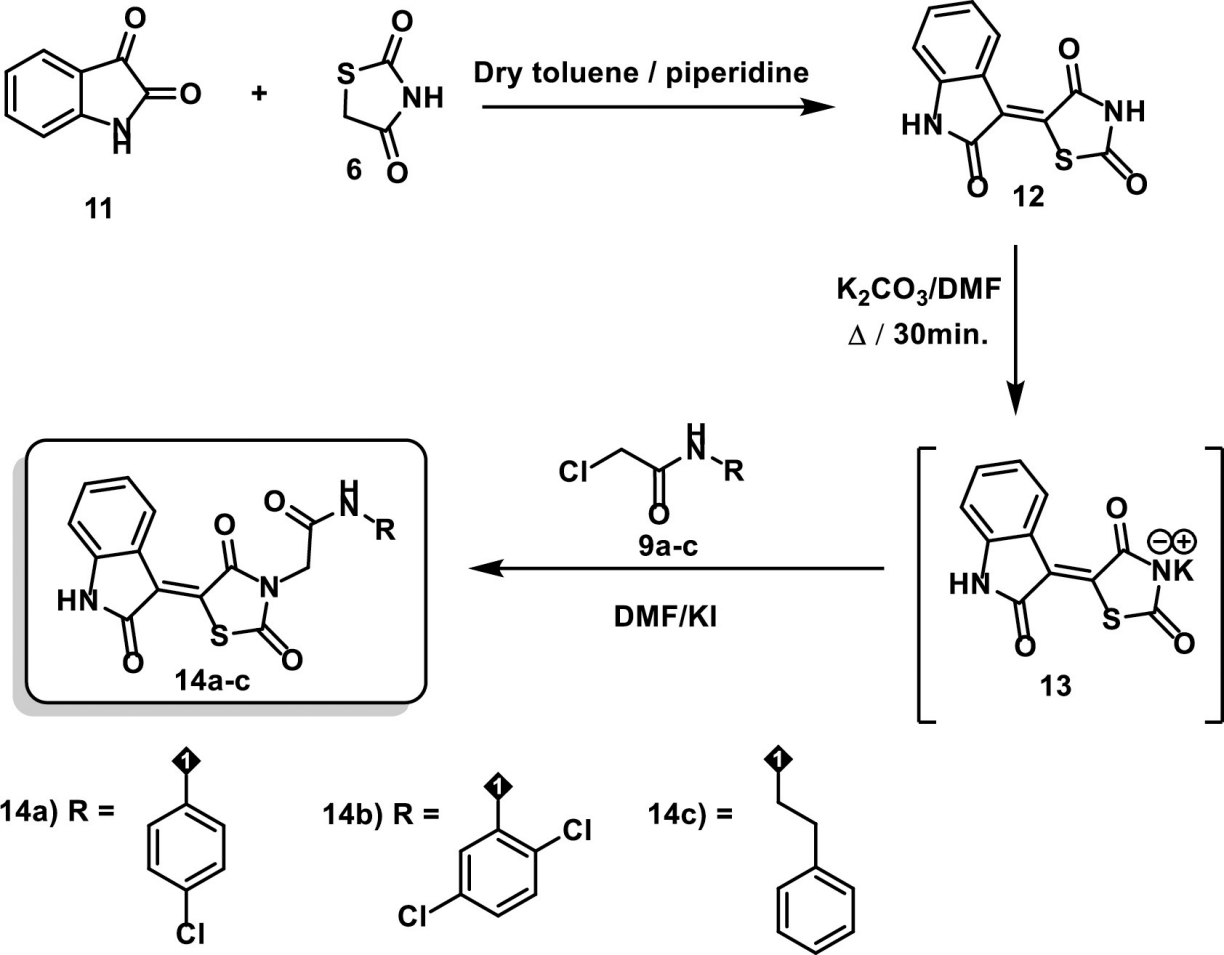
Synthesis of compounds 14a-c.

The target novel derivatives **10a-b** and **14a-c** were synthesized as depicted in **Schemes 1** and **2**. The starting materials **2** was prepared using the Vilsmeier-Haack reaction in which acetanilide is converted into 2-chloroquinoline-3-carbaldehyde by the action of by Vilsmeier-Haack reagent (DMF+POCl_3_) [29]. Next, compounds **3**, and **6** were prepared in high yields according to the literature procedures [30–34]. To prepare the key intermediate **7,** Knoevenagel condensation reaction was utilized [35]. In this reaction, quinoline **3** was condensed with thiazolidine-2,4-dione **6** in the presence of piperidine which acts as organocatalyst. In compound **7**, there are two NH groups, the thiazolidine NH group has a very strong acidic proton flanked by 2 carbonyl groups which stabilize the resulting anion produced after salt formation. Therefore, the thiazolidine NH group is involved in the salt formation rather than the quinoline NH group. Consequently, treatment of compounds **7** with dry K_2_CO_3_ in DMF with continuous stirring afforded the corresponding *in situ* potassium salt **8**. Subsequent heating of potassium salt **8** with 2-chloro-*N*-substitutedacetamide derivatives **9a**, **b** in dry DMF / KI mixture produced the corresponding desired compounds **10a**, **b**, respectively (**Scheme 1**).

On the other hand, compound **12** was prepared via condensation of thiazolidine-2,4-dione **6** with isatin **11** in a dry toluene/piperidine mixture following the reported procedures [34]. In compound **11**, the amide carbonyl is less electrophilic as it is stabilized by the lone pair of electrons of the nearby nitrogen atom. Therefore, the carbonyl in position 3 is more reactive and it is involved in the c-c bond formation with compound **6**. Treating compound **12** with dry K_2_CO_3_ in DMF with continuous stirring afforded the corresponding *in situ* potassium salt **13**. Heating a mixture of compound **13** with 2-chloro-*N*-substitutedacetamide derivatives **9a**-**c** in dry DMF / KI mixture yielded the desired products **14a**-**c**, respectively (**Scheme 2**).

IR spectra of the target derivatives **10a**-**b** and **14a**-**c** confirmed their molecular structures by the presence of C = O bands ranging from 1673 to 1753 cm^-1^ besides NH bands ranging from 3142 to 3449 cm^-1^. Concerning quinoline derivatives **10a**-**b**, singlet signals corresponding to the two amidic NHs were found in ^1^H NMR spectra around 10.65 and 10.24 ppm. With regard to the indoline derivatives **14a**-**c,** the structures of the obtained derivatives were supported by the generated spectral data. The ^1^H NMR spectra of compounds displayed singlet signals around 11.32 and 10.24 ppm for the NHs. Matching these findings. ^13^C NMR spectra displayed the characteristic peaks at the fingerprint regions.

### 2.2. Biological evaluation

#### 2.2.1. Assessment of *in vitro* anti-proliferative activity

The cytotoxicity effects of the synthesized candidates were evaluated against A549, Caco-2, HepG2, and MDA-mb-231 cell lines. MTT assay method was applied using the sub-IC_50_ concentrations of each compound as the treatment dose. Doxorubicin was used as a reference molecule. The obtained results demonstrated the anti-cancer effects of the tested compounds against all tested cell lines with different degrees (**Table 1****)**. Also, it was noticed that Caco-2 was the most sensitive cell line.

**Table 1:** *In vitro* cytotoxicity against A549, Caco2, HepG2, and MDA-mb-231cell lines.

Comp.	Anti-proliferative activity (IC_50_ μM)			
	A549	Caco2	HepG2	MDA-mb-231
**10a**	85.0 ± 7.5	82.5 ± 7.2	173.5 ± 16.3	131.5 ± 5.2
**10b**	92.5 ± 8.6	62.5 ± 5.4	71.0 ± 6.4	31.5 ± 2.5
**14a**	170.0 ± 14.5	1.5 ± 0.08	31.5 ± 2.5	84.0 ± 7.3
**14b**	292.5 ± 25.1	74.0 ± 6.5	42.5 ± 3.2	94.5 ± 8.4
**14c**	281.5 ± 27.3	192.5 ± 18.1	92.0 ± 8.1	484 ± 47.2
**Doxorubicin**	86.44 ± 7.1	3.5 ± 0.22	1.2 ± 0.07	0.98 ± 0.001

The most potent cytotoxic member was compound **14a.** It showed high cytotoxic effects against Caco-2 (IC_50_ = 1.5**μ**M) and HepG-2 (IC_50_ = 31.5 **μ**M). Meanwhile, compound **10b** came in the second order as it showed moderate anticancer effect against MDA mb-231 cell line (IC_50_ = 31.5 **μ**M) and Caco-2 (IC_50_ = 62.5 **μ**M). Regarding A549 cell line, compound **10a** displayed the most potent cytotoxic activity (IC_50_ = 85 **μ**M).

#### 2.2.2. Assessment of VEGFR-2 inhibition

Compounds **10a**, **b** and **14a**-**c** were evaluated for their VEGFR-2inhibitory activity. Sorafenib (the reference drug) produced IC_50_ value of 53.65 nM (**Table 2)**. The quinoline derivative **10a** (IC_50_ = 65.16 nM) is the most active member compared to sorafenib. Concerning the indoline derivatives **14a**-**c**, it was found that different hydrophobic tails gave valuable SAR. In detail, compound **14c** (IC_50_ = 81.46 nM) incorporating phenethyl moiety as a hydrophobic tail was the most active indoline member. This revealed that the terminal aliphatic moieties have the highest positive effect on VEGFR-2 inhibition. Shifting the hydrophobic tail into aromatic moieties as in compounds **14a** (incorporating 4-chlorophenyl moiety, IC_50_ = 91.51 nM) and **14b** (incorporating 2,4-dichlorophenyl moiety, IC_50_ = 85.85 nM) led to a slight decrease in the VEGFR-2 inhibitory activity.

**Table 2:** Inhibitory effects of compounds 10a, b and 14a-c against VEGFR-2.

Comp.	VEGFR-2 IC_50_ (nM)
**10a**	65.16 ± 5.5
**10b**	164.5 ± 15.3
**14a**	91.51 ± 8.2
**14b**	85.85 ± 7.6
**14c**	81.46 ± 7.3
**Sorafenib**	± 4.5

#### 2.2.3. Safety pattern of the tested compounds

In this work, the safety pattern of the synthesized derivatives **10a**, **b** and **14a**-**c** was also evaluated by testing their *in vitro* cytotoxicity against Vero non-cancer cell line using the MTT assay protocol. The obtained results showed an IC_50_ range of 194–1580 **μ**M presenting the safety profile of the examined hits against the Vero normal cell line. Compounds **10b** (IC_50_ = 1580 **μ**M) and **14b** (IC_50_ = 1270 **μ**M) were the safest members **(Table 3).**

**Table 3:** IC_50_ results of 10a, b and 14a-c against Vero cell line.

Compound No.	Cytotoxicity against Vero (IC_50_ μM)
**10a**	194 ± 18.2
**10b**	1580 ± 155.1
**14a**	290 ± 28.3
**14b**	1270 ± 126.5
**14c**	± 96.6

#### 2.2.4. Selectivity index (SI)

To clinch the cyto-protective properties of the compounds, the drug safety parameter for anticancer activity of the compounds was estimated by comparing their cytotoxic effect against tumor cell lines and normal cell lines. The normal human Vero cell line was used as a control in this study. **Table 4** shows the calculated SI for the tested compounds by scaling its IC_50_ value against various tumor cell lines and IC_50_ value against a normal cell line (Vero cell line) [36]. When the SI value of a compound is ≥10 then it is considered a selective anticancer agent [37].

**Table 4:** Selectivity indices of the synthesized compounds.

Compounds	(A549) ^a^	(Caco-2)^b^	(HepG2)^c^	(MDA-MB-231)^d^
**10a**	2.280	2.352	1.118	1.460
**10b**	17.080	25.290	22.250	51.333
**14a**	1.704	212.500	9.196	3.375
**14b**	4.340	17.150	29.917	13.555
**14c**	3.463	5.0654	10.601	2.020

^a^ SI = Cytotoxicity against Vero cells / Cytotoxicity against A549 cell line.

^b^ SI = Cytotoxicity against Vero cells / Cytotoxicity against Caco-2 cell line.

^c^ SI = Cytotoxicity against Vero cells / Cytotoxicity against HepG2 cell line.

^d^ SI = Cytotoxicity against Vero cells / Cytotoxicity against MDA-MB-231 cell line.

**Fig 2:**
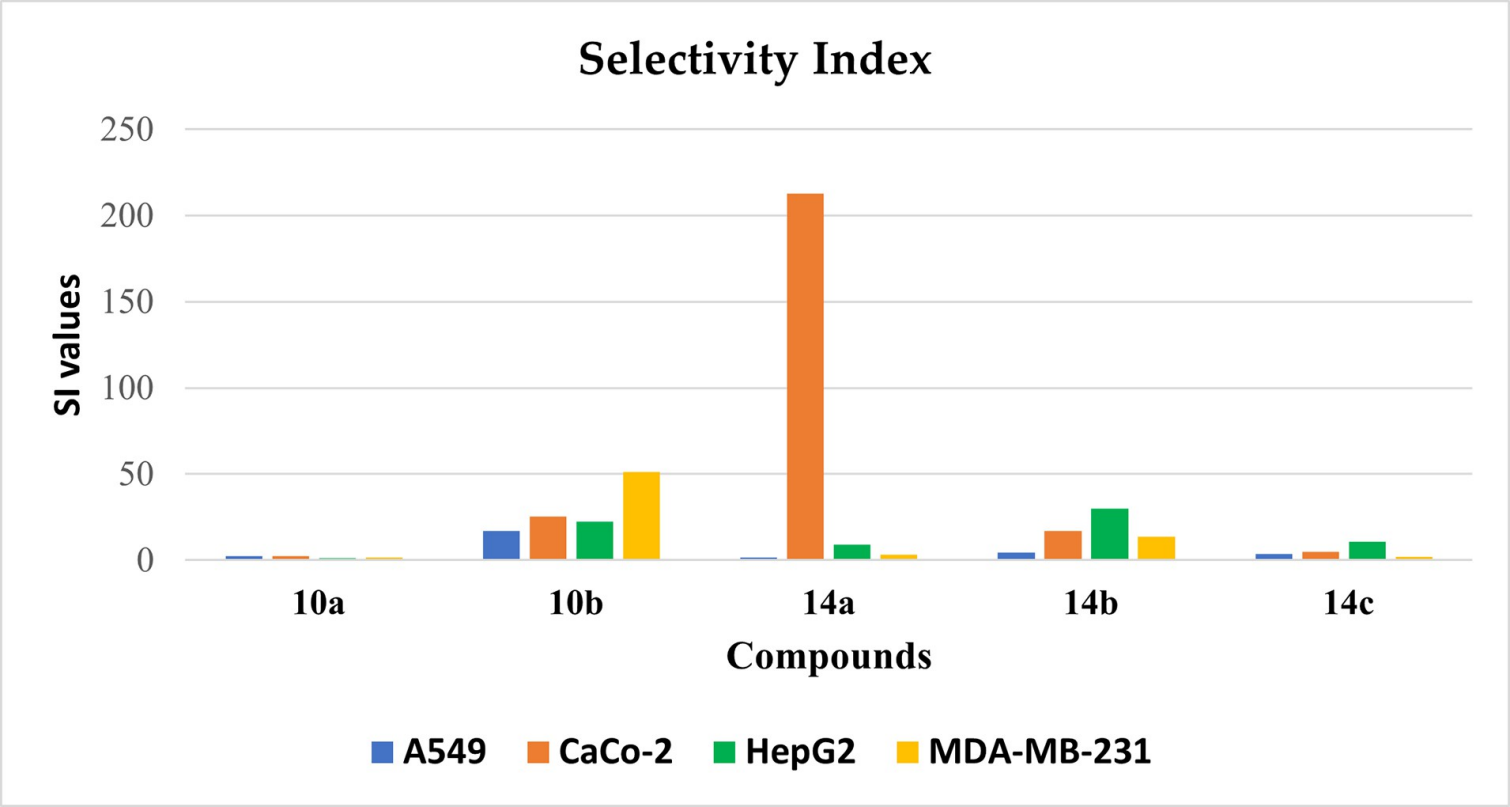
Selectivity indices of the synthesized compounds.

As previously stated in the former test, all the tested derivatives had lower potency against Vero cell lines. These outcomes prompted us to investigate the selectivity profile of the synthesized compounds by calculating selectivity index value for each compound against the four cancer cells. The maximum selectivity index value was recorded for **14a** (212.5) against Caco-2 cell lines as displayed in **Fig 2**. Therefore, compound **14a** was decided to be the safest potent member of synthesized compounds and was nominated for further biological testing.

#### 2.2.5. Effect of compound 14a on Caco-2 cells migration

**Fig 3:**
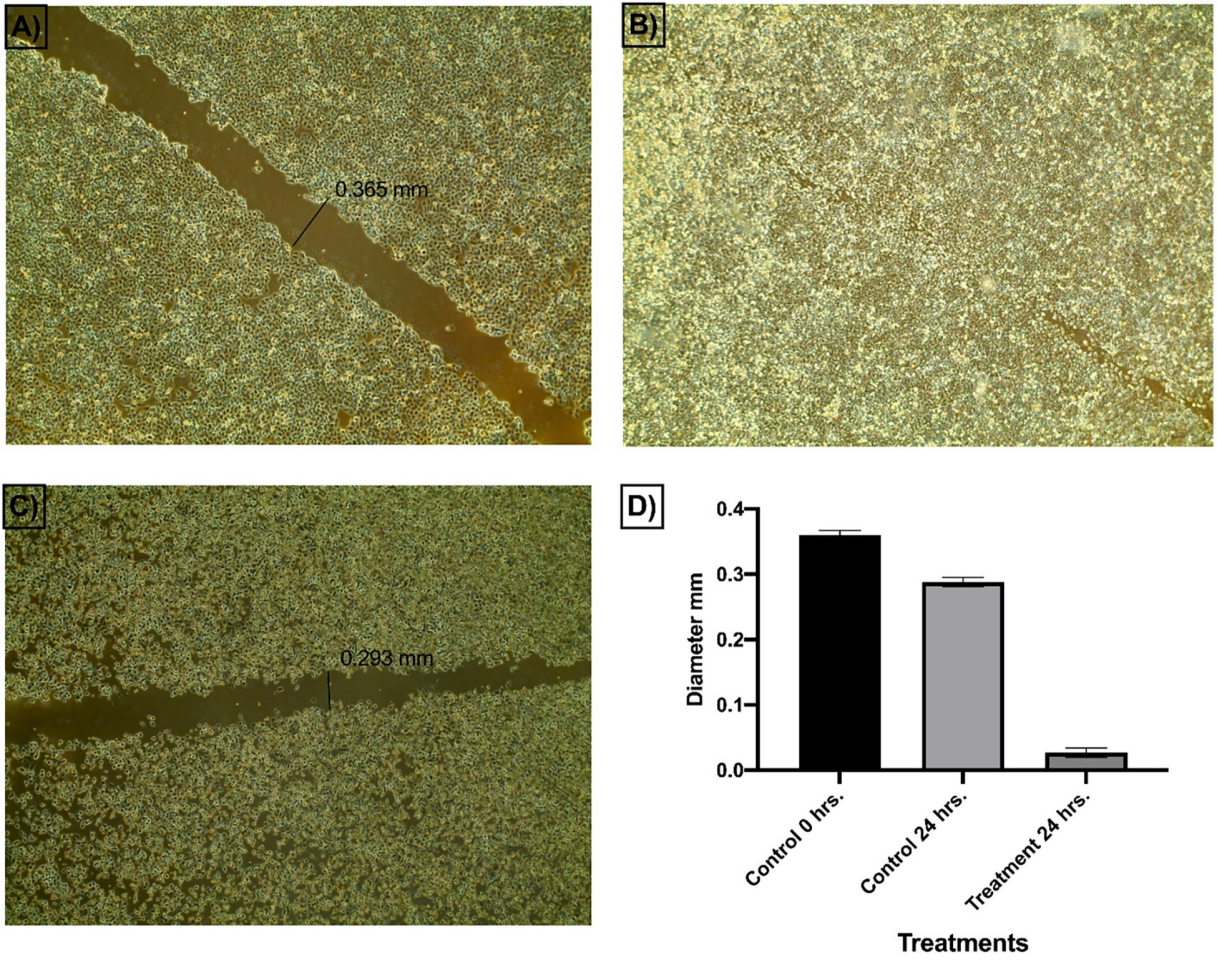
Effect of compound 14a on cells migration and healing efficacy of Caco-2 cells.

The *in vitro* scratch assay[38] was used to assess the prospective of compound **14a** to inhibit the ability of Caco-2 cells to migrate and heal. The basic idea behind this test is to create a scratch in a cancer cell line monolayer, measure the diameter at the start time and at regular intervals to investigate the potential of the cancer cell to migrate and heal. The findings of the treated cell line are then compared to the untreated cell line. **Fig 3** shows images of scratched areas at time points 0 and 24.

The obtained findings revealed that the scratch of the untreated cells was completely closed within 24 h (**Fig 3B**). On the other hand, the width of the scratch in Caco-2 cell lines that were treated with **14a** (1 **μ**M) slightly decreased from the control cells’ scratch width at 0 hr (0.356 to 0.293 mm) as displayed in **Fig 3A** and **3C**. These findings confirmed that even at a low concentration of 1 **μ**M, compound **14a** can inhibit the migration and healing of Caco-2 cells in a significant manner. Also, at the end of the 24-hour incubation period, compound **14a** was able to make a significant phenotypic change in cancer cell morphology which might be linked to the occurrence of apoptosis.

#### 2.2.6. Alternation of cancer cells gene expression after Caco-2 treatment with 14a using RT-qPCR

The apoptosis process (programmed cell death) is mediated by different gene families such as caspases, tumor necrosis factor (TNF) receptor gene superfamily, or B cell lymphoma (Bcl)-2 family. Survivin is a pro-survival protein that is overexpressed in many cancer cells in the G2-M phase. This protein has been linked to tumor progression control and resistance to cancer chemotherapeutics. Furthermore, the transforming growth factor (TGF) is a key protein that can promote the development of normal cells and participate in the suppression mechanism of tumor cells [39]. Dysregulation of TGF-β activation and signaling may result in apoptosis. Moreover, overexpression of the Bcl2 gene can inhibit apoptosis. Meanwhile, overexpression of Bcl-xL enhances autophagic cell death [40].

**Fig 4:**
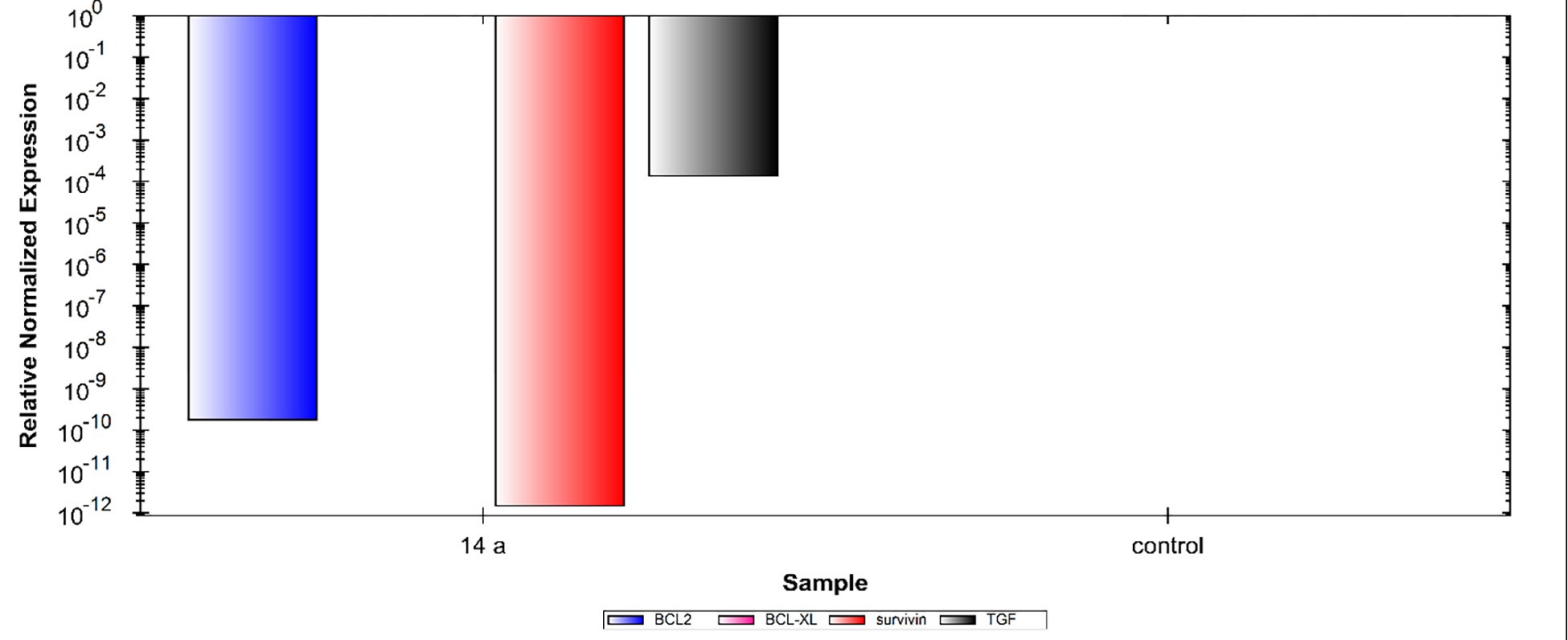
Relative gene expression levels of 4 different genes (Bcl-2, Bcl-xl, Survivin, and TGF) in Caco-2 cell line treated with 14a using RT-qPCR.

In this study, the Caco-2 cell line was treated with 1.5 **μ**M (IC_50_ value) of compound **14a**. The results showed noticeable variations in the expression levels of the four cancer correlated genes (Bcl-2, Bcl-xl, TGF, and Survivin). In detail, compared to control cells, compound **14a** caused significant down-regulation of Bcl-2, and Survivin and TGF gene expression levels. Meanwhile, the gene expression level of Bcl-xl showed non-detectable change. These findings indicate the efficiency of compound **14a** in the induction of apoptosis (**Fig 4)**.

### 2.3. *In silico* studies

#### 2.3.1. Molecular docking

The docking studies can give a good insight about the binding modes of many active molecules [41–43]. These studies aimed to determine the binding modes and orientation of the designed VEGFR2 kinase inhibitors. In the present work, the crystal structure of VEGFR2 (PDB code 4ASD) was retrieved from the protein data bank. The docking protocol was initially validated by re-docking of the co-crystallized ligand (sorafenib) into the active site of VEGFR 2. The respective validation criteria in this study showed an RMSD value = 1.15 Å, and a docking score = -11.25 kcal/mole.

**Fig 5:**
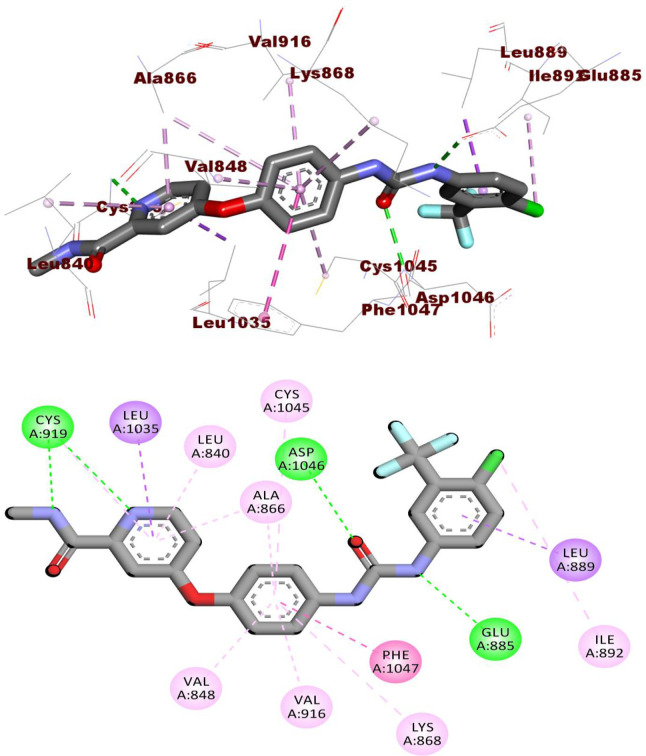
The docked pose of sorafenib against VEGFR-2.

As presented in **Fig 5**, the docking pose of sorafenib involved three H-bonding with Cys919, Glu885, and Asp1046. Also, it interacted with the hydrophobic pocket formed by Leu889, Leu1019, and Ile892 via several hydrophobic interactions.

**Fig 6:**
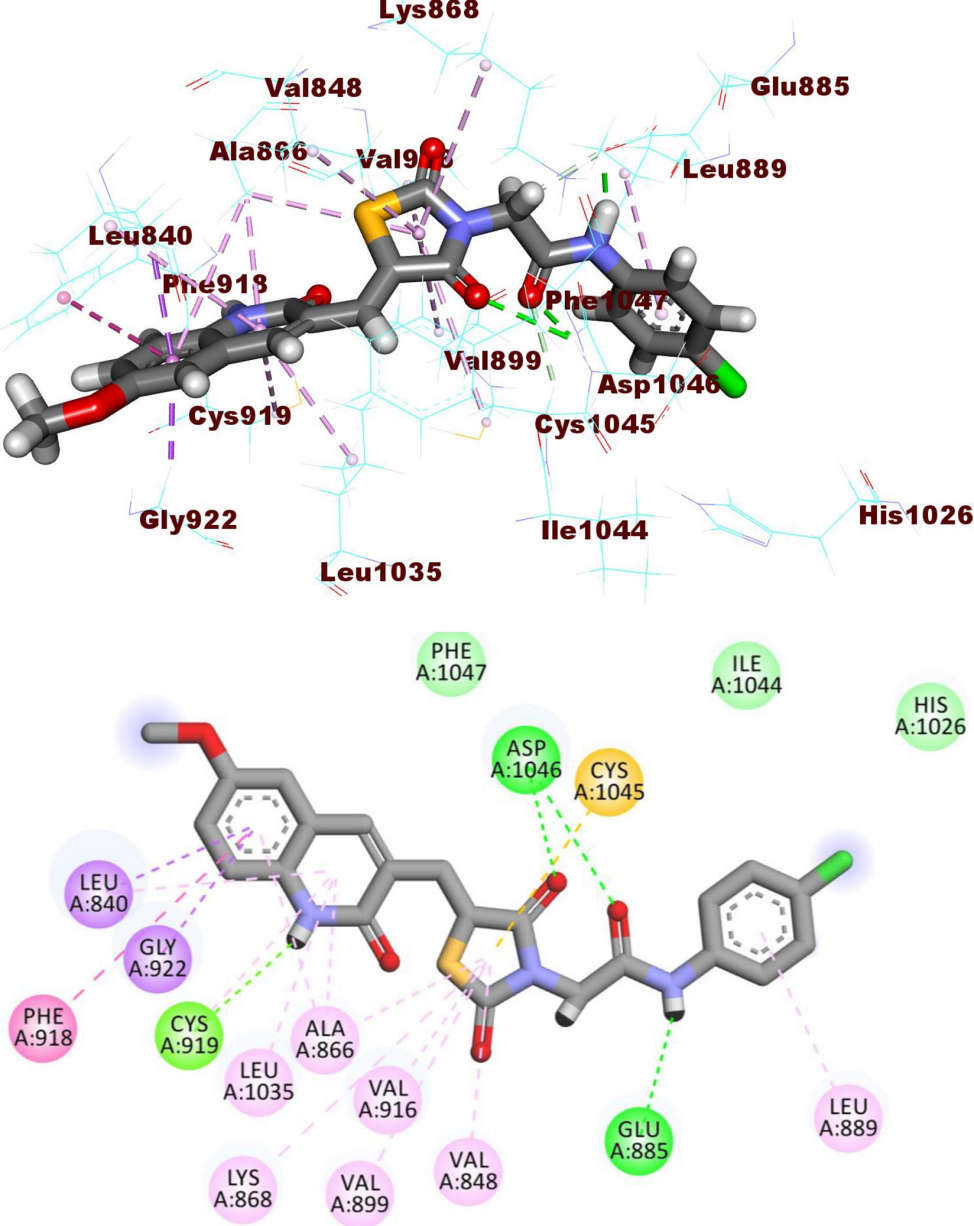
The docked pose of 10a against VEGFR-2.

Compound **10a** occupied the hinge region via its 2-oxoquinoline moiety. It formed a hydrogen bond with Cys919 and eight hydrophobic interactions with Cys919, Leu840, Leu1035, Ala866, and Phe918. Next, the thiazolidine-2,4-dione moiety was incorporated in the linker region via the formation of five pi-pi interactions with Val916, Val848, Val899, Ala866, and Lys868. In addition, it formed one hydrogen bond with Asp1046. As well, the amide moiety was buried in the DFG motif region to form two H bonds with Asp1046 and Glu885. Lastly, the *p*-chlorophenyl group achieved one hydrophobic interaction with Leu889 in the hydrophobic back pocket **(Fig 6).**

**Fig 7:**
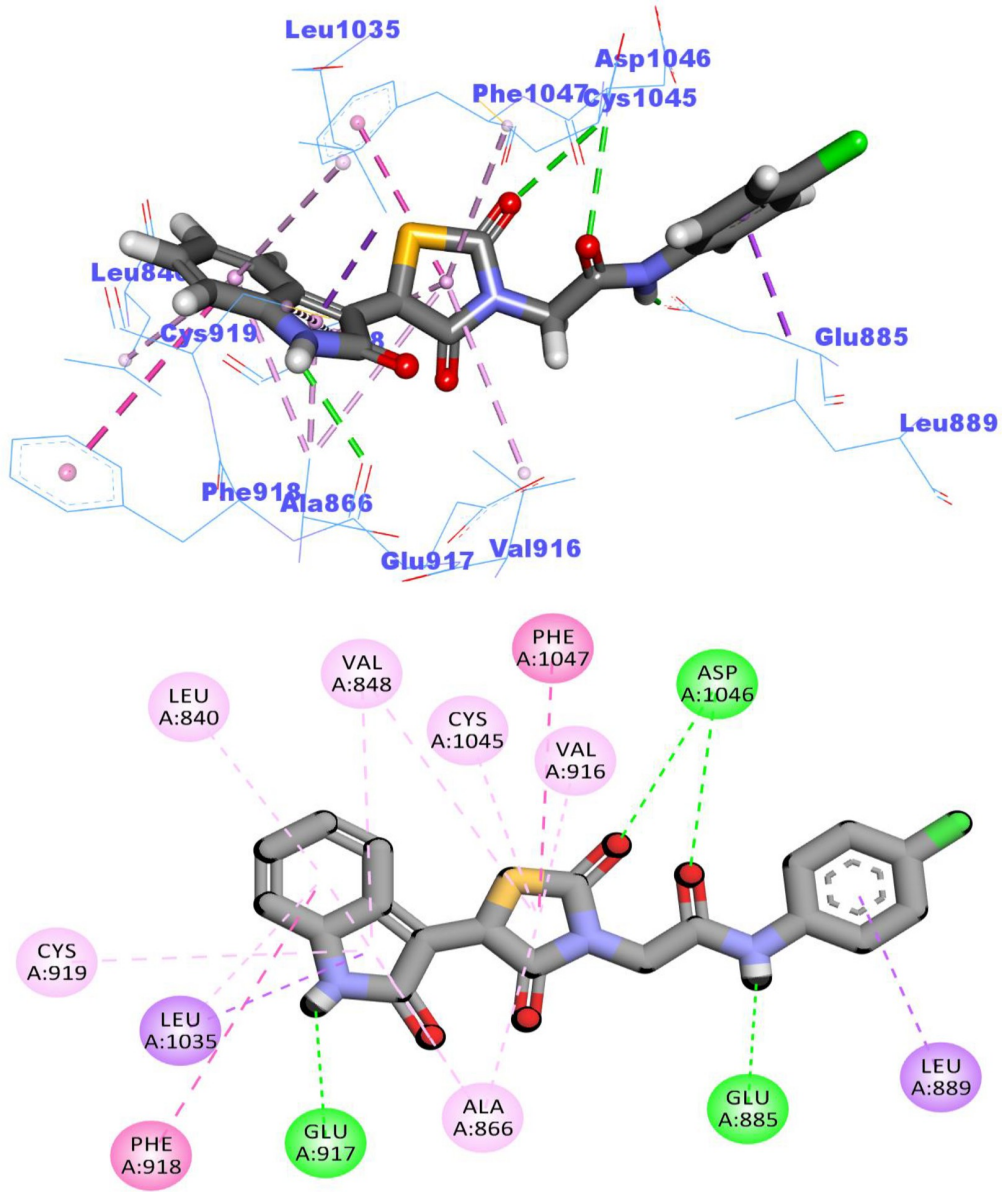
The docked pose of 14a against VEGFR-2.

The docking findings of compound **14a,** the most promising member in this study, revealed similar binding pattern with sorafenib. The 2-oxoindolin moiety was oriented toward the hinge region forming one H bond interaction with Glu917 residue and seven hydrophobic interactions with, Leu840, Leu1035, Ala866, Phe918, and the key amino acid Cys919. Meanwhile, the thiazolidine-2,4-dione moiety was accommodated in the linker region to form five hydrophobic interactions with Val916, Val848, Ala866, Phe1047, and Cys1045. Similarly, compound **14a** interacted *via* one H-bond with Glu885 (1.80 Å) and another one with Asp1046 (2.02 Å) of the conserved DFG motif region. Finally, one hydrophobic interaction was observed between compound **14a** and hydrophobic side chains of Leu899 in the hydrophobic back pocket of VEGFR-2 (**Fig 7**). The binding modes of compounds were presented in **S.2.1.1 and S2.1.2. Sections in S1 File.**

**Fig 8:**
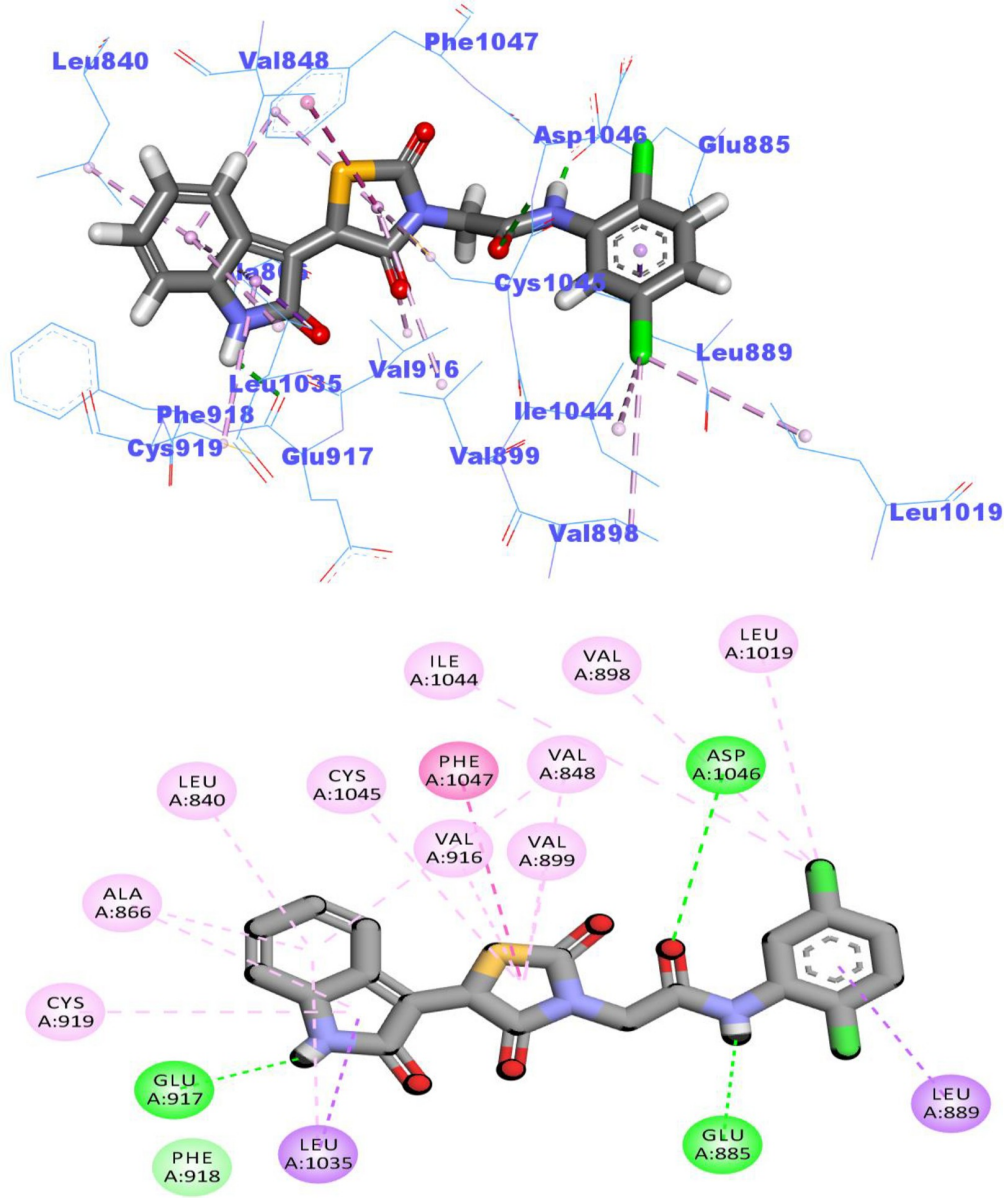
The docked pose of 14b against VEGFR-2.

The docked pose of compound **14b** was depicted in **Fig 8.** The amide group formed two hydrogen bonds with Glu885 (COO^-^, 1.65 Å) and Asp1046 (NH, 2.04 Å). Moreover, it formed four hydrophobic interactions in the linker region with Val916, Val848, Phe1047, and Cys1045 via its thiazolidine-2,4-dione moiety. As well, the 2-oxoindolin moiety was buried in the hinge region forming one H bond interaction with Glu917 besides several hydrophobic interactions with Leu840, Leu1035, Ala866, Val848, and Cys919. Moreover, the 2,5-dichlorophenyl moiety formed four hydrophobic interactions with Leu899, Ile1044, Leu1019, and Val898 in the allosteric hydrophobic pocket.

#### 2.3.2. Flexible alignment study

**Fig 9:**
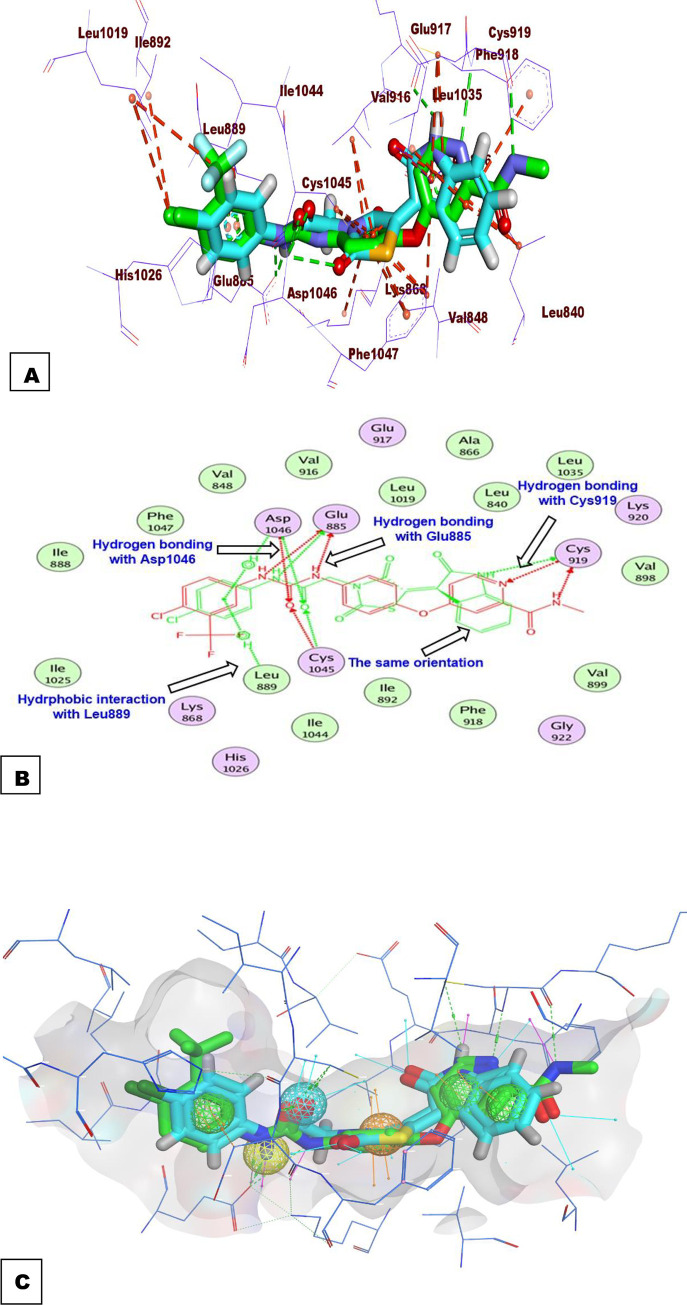
Orientation compound 14a and sorafenib inside the active sites of VEGFR-2.

In this test, 3D- flexible alignment of compound **14a** (the most promising member) with sorafenib was carried out. The results revealed a general good overlap of compound **14a** with sorafenib with the same spatial orientation. In details, 2-oxoindolin, thiazolidine-2,4-dione, amide, and 4-chlorophenyl moieties of compound 14a showed the same orientation of the *N*-methylpicolinamide, phenoxy, urea, and 4-chloro-3-(trifluoromethyl)phenyl) moieties of sorafenib, respectively (**Fig 9A** and **9B**). This study revealed that compound **14a** has the same basic pharmacophoric features of sorafenib and can occupy the VEGFR-2 kinas active pocket with the same orientation of sorafenib as displayed in (**Fig 9C**).

#### 2.3.3. ADMET profiling study

**Fig 10:**
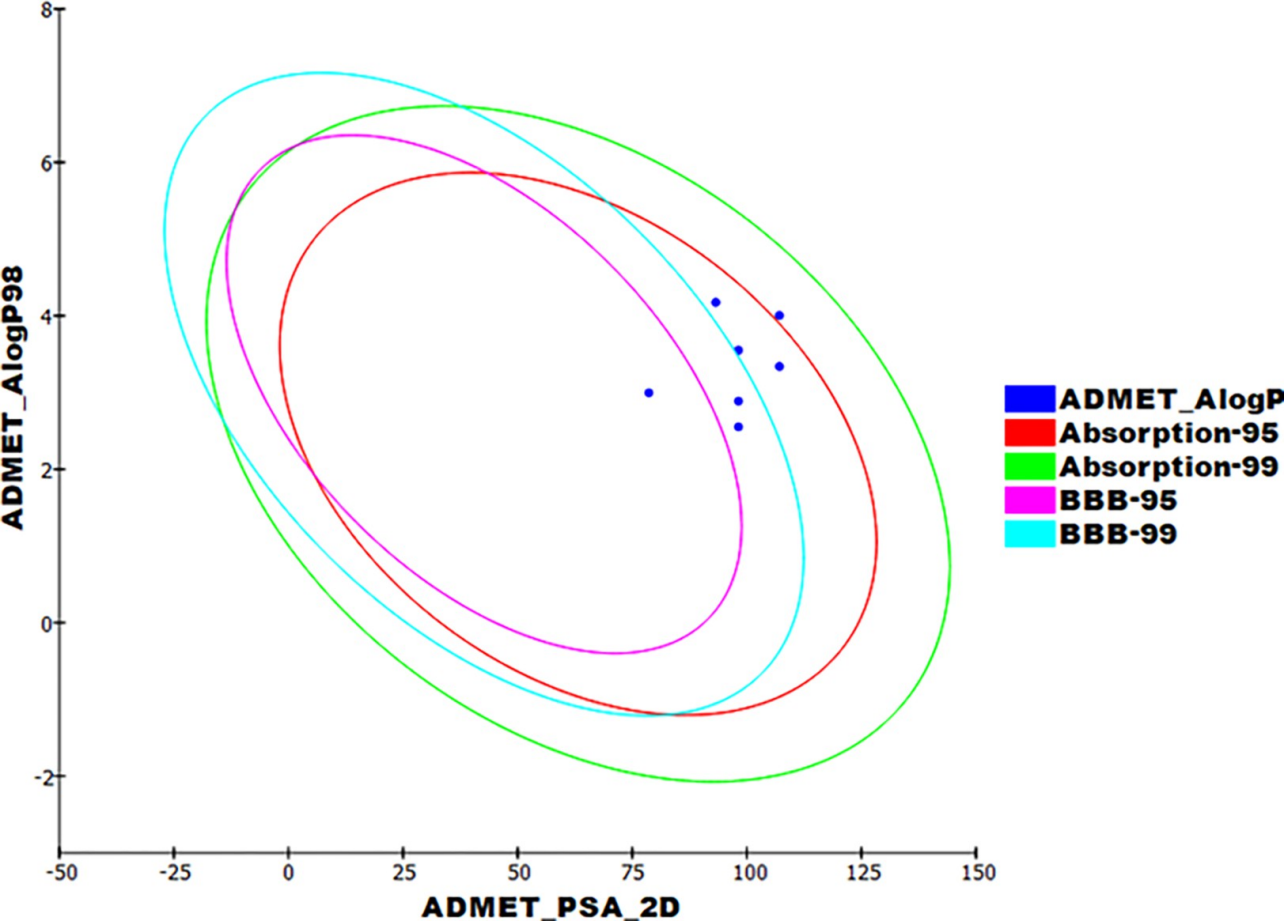
Theoretical ADMET characters.

The pharmacokinetic properties were determined computationally for compounds **10a**, **b** and **14a**-**c** using Discovery studio 4.0 **(Fig 10)**. Sorafenib and sunitinib were used as references.

As shown in **Table 5,** compounds **10a**-**b** and **14a**-**c** achieved good absorption levels upon oral administration. Moreover, the titled compounds could exhibit acceptable BBB penetration levels. Finally, all compounds showed a theoretical non-inhibitory effect against CYP2D6 with plasma protein binding ability than 90%.

**Table 5:** Different theoretical ADMET characters of the tested compounds.

Comp.	BBB^a^	Solubility^b^	Absorption^c^	CYP2D6^d^	PPB^e^
**10a**	■■■■	■■	⌦	⌦	√
**10b**	■■■■	■■	■	⌦	√
**14a**	■■■	■■	⌦	⌦	√
**14b**	■■■■	■■	⌦	⌦	√
**14c**	■■■	■■	⌦	⌦	√
**Sorafenib**	■■■■	■	⌦	⌦	√
**Sunitinib**	■■	■■	⌦	⌦	⌦

^a^BBB level, blood brain barrier level, ⌦ = very high, ■ = high, ■■ = medium, ■■■ = low, ■■■■ = very low.

^b^Solubility level, ■ = very low, ■■ = low, ■■■ = good, ■■■ = optimal.

^c^Absorption level, ⌦ = good, ■ = moderate, ■■ = poor, ■■■ = very poor.

^d^CYP2D6, cytochrome P2D6, √ = inhibitor, ⌦ = non inhibitor.

^e^PBB, plasma protein binding, ⌦ means less than 90%, √ means more than 90%

#### 2.3.4. *In silico* toxicity studies

Toxicity profiles were computed for the tested derivatives **10a**-**b** and **14a**-**c** against references (sorafenib and sunitinib) based on seven constructed toxicity models created in Discovery studio software [44, 45] as presented in **Table 6**.

**Table 6:** *In silico* toxicity studies.

Comp.	Carcinogenicity ^a^	Carcinogenic Potency TD_50_ (mg/kg body weight/day)	Rat Maximum Tolerated Dose (g/kg body weight)	Rat Oral LD_50_ (g/kg body weight)	Rat Chronic LOAEL (g/kg body weight)	Ocular Irritancy^b^	Skin Irritancy^b^
**10a**	⌦	15.044	0.026	0.936	0.002	√	⌦
**10b**	⌦	13.611	0.021	0.316	0.002	√	⌦
**14a**	⌦	14.284	0.059	1.667	0.030	√	⌦
**14b**	⌦	13.040	0.048	0.707	0.028	√	⌦
**14c**	⌦	121.482	0.042	1.933	0.069	√	⌦
**Sorafenib**	⌦	14.244	0.089	0.823	0.005	√	⌦
**Sunitinib**	⌦	4.134	0.178	2.876	0.040	√	⌦

^a^ Carcinogenicity: ⌦ = non-carcinogenic, √ = carcinogenic

^b^ skin and ocular irritancy = ⌦ = non-irritant, √ = irritant

All the tested molecules were predicted as non-carcinogenic. In addition, all members except **10b** and **14b** had carcinogenic potency TD_50_ values ranging from 14.284 to 121.482 mg/kg body weight/day, which were higher than that of sorafenib and sunitinib (14.244, 4.134 mg/kg body weight/day, respectively). In addition, all members had rat maximum tolerated doses lower than that of sorafenib and sunitinib. For the rat oral LD_50_ model, compounds **10a**, **14a** and **12c** displayed oral LD_50_ values of 0.936, 1.667 and 1.933 mg/kg body weight/day. Such values are far more than that of sorafenib (0.823 mg/kg body weight/day). Also, all compounds except **10a** and **10b** showed rat chronic LOAEL values ranging from 0.030 to 0.069 which were higher than that of sorafenib (0.005). Finally, all candidates showed mild irritancy against eye with non-irritancy against skin.

#### 2.3.5. Molecular dynamics simulations

The application of Molecular dynamics (MD) simulations is closely to be a regular *in silico* method in the area of drug development and discovery [46]. The main benefit of these types of work is the extreme accuracy in the analysis of every structural and entropic variation in the considered compound-protein system. Moreover, this experiment occurred at a very accurate atomic resolution through a given time [47]. Respectively, MD simulations can precisely calculate the resulted variations after the compound-protein binding in thermodynamic as well as kinetic levels [48]. These advantages presented the MD as a successful tool to explain the structure-functional changes of the considered compound-protein complex. It reveals essential features such as stability, binding energy, and the kinetics of the examined complex [49].

**Fig 11:**
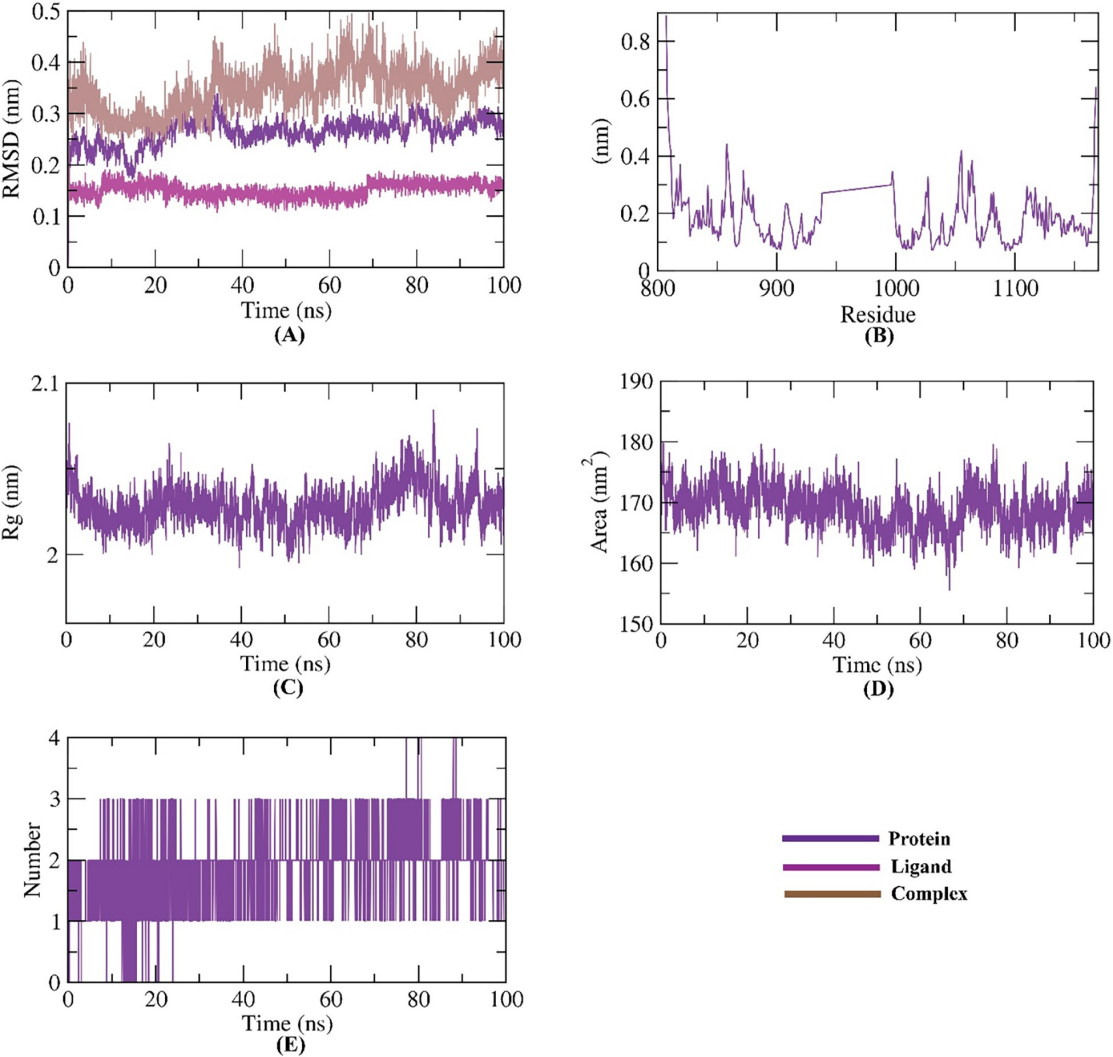
M D simulations experiments: A) RMSD values of **14a**-VEGFR-2 system before and after binding, B) RMSF of **14a**-VEGFR-2 system C) R_g_ of **14a**-VEGFR-2 system D) SASA of **14a**-VEGFR-2 system E) H- bonding between **14a**-VEGFR-2 system.

**Fig 12:**
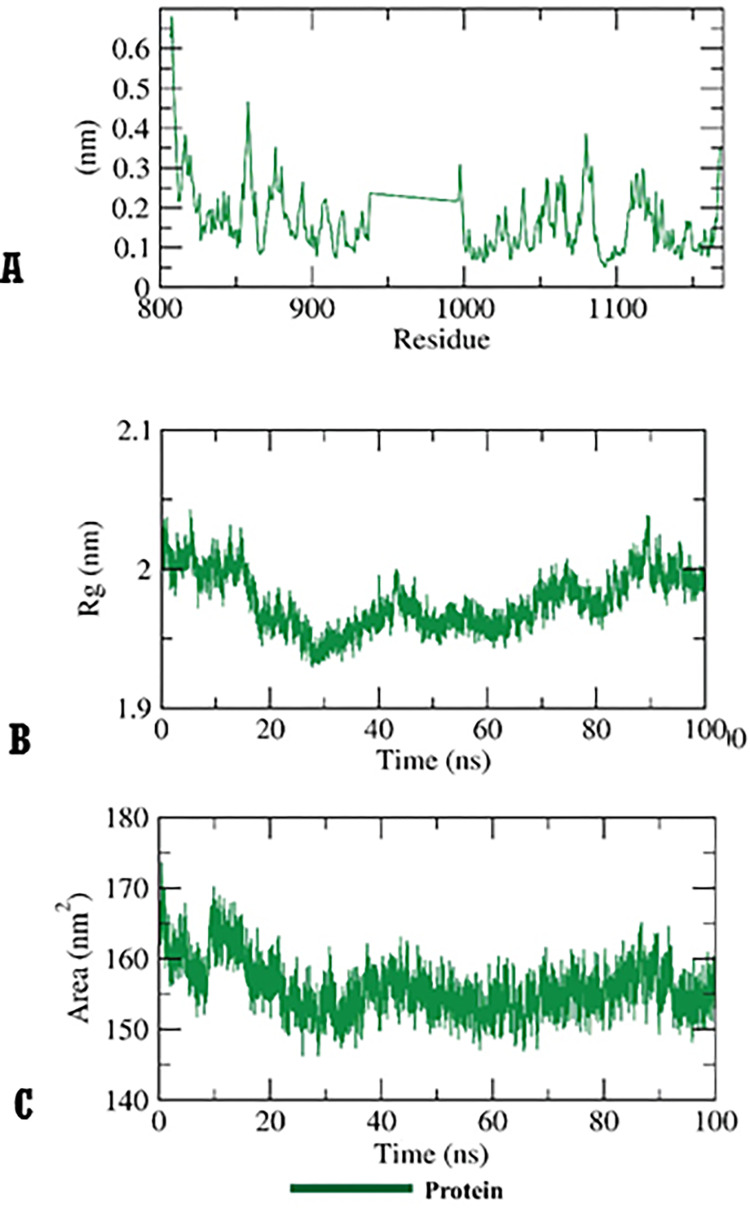
A) RMSF B) R_g_ C) SASA of VEGFR-2 enzyme in apo state.

To identify the conformational alterations that transpired in the VEGFR-2-**14a** complex because of binding, RMSD values were measured before and after **14a** binding with the VEGFR-2. **Fig 11A** illustrates that VEGFR-2, **14a**, and the VEGFR-2-**14a** complex had low RMSD values and didn’t reveal major fluctuations during the MD time (100ns). These results declare a high stability. The flexibility of VEGFR-2 enzyme after binding was checked in terms of RMSF to identify the fluctuated regions during 100 ns of simulation. Fortunately, **14a** didn’t make the VEGFR-2 flexible (**Fig 11B**) after binding comparing the apo state of VEGFR-2 (**Fig 12A**). The radius of gyration (R_g_) of the enzyme VEGFR-2 was computed to explore the compactness of the examined VEGFR-2-**14a** system. Interestingly, the R_g_ of the VEGFR-2-**14a** complex remained stable till the end of the experiment (**Fig 11C**) and was not very distict to that of VEGFR-2 apo state (**Fig 12B**). VEGFR-2-**14a** complex interaction against the solvents in the surrounding media was examined by solvent accessible surface area (SASA) over a period of 100 ns. Excitingly, VEGFR-2 enzyme didn’t exhibit a noticeable expansion nor reduction of the surface area revealing nearly similar SASA values from 0 till 100 ns (**Fig 11D**). The obtained values were near to that of VEGFR-2 apo state (**Fig 12C**) indicating that there is no major conformational alternations occurred in VEGFR-2 enzyme due to **14a** binding. Also, hydrogen bonding in the VEGFR-2-**14a** complex was computed and the maximum incorporated H-bonds number was found to be three (**Fig 11E**).

**Fig 13:**
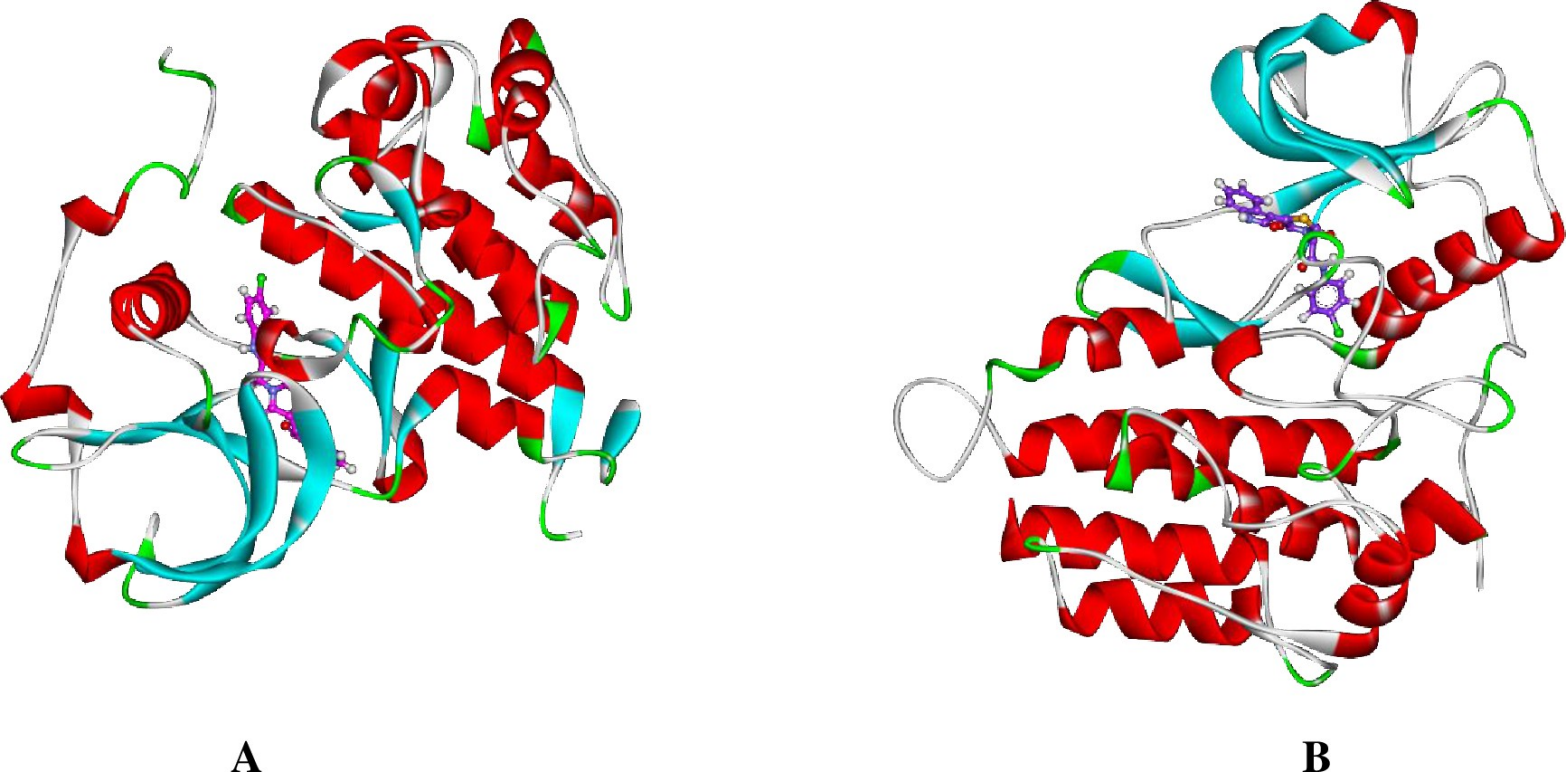
**14a**-VEGFR-2 structure at (A) 1 ns, (B) 100 ns.

To understand the conformational changes that was reported in the RMSD study, the conformational change analysis of the **14a**-VEGFR-2 complex was analyzed during the 1, and 100 ns of the MD production run as illustrated in **Fig 13**. The conformational changes in VEGFR-2 were indicated. Most importantly, the binding stability as well as the integrity of the **14a**-VEGFR-2 complex was confirmed.

**Fig 14:**
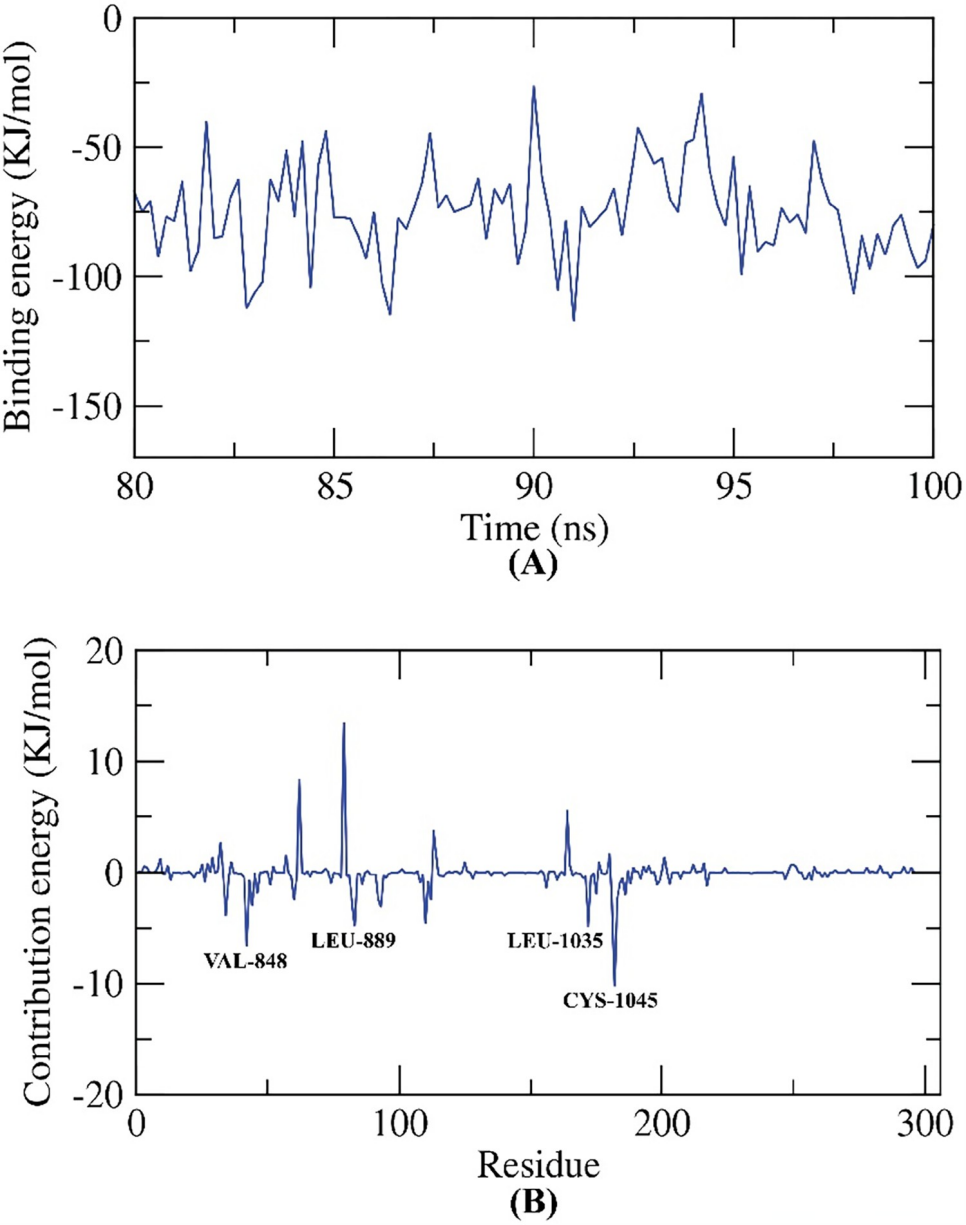
MM-PBSA experiments.

*2*.*3*.*5*.*1*. *MM/PBSA studies*. The binding free energy of **14a**-VEGFR-2 system was computed the last 20 ns of the obtained MD production with a 100 ps interval from the MD trajectories using. Compound **14a** exhibited a binding free energy of -75 KJ/mol with VEGFR-2 enzyme (**Fig 14A**). Moreover, the participation of each individual residue in the binding free energy of **14a**-VEGFR-2 system were disclosed. The total binding free energy of the **14a**-VEGFR-2 system was disintegrated into per individual residue energy. The output of this experiment sheds a light into the pivotal amino acid residues that contributed remarkably to the binding of **14a** and VEGFR-2. The following amino acids: VAL-848, LEU-889, LEU-1035 and CYS-1045 of VEGFR-2 shared more than -5 KJ/mol binding energy and considered as hotspots in the binding with **14a** (**Fig 14B**).

#### 2.3.6. Density Function Theory (DFT) calculations

The enzymatic inhibitory activity of targeted compounds against VEGFR-2 showed that the indoline derivative **14a** was the most active compound as incorporating 4-chlorophenyl moiety raised the IC_50_ to 91.51 nM, **Table 2**. As discussed before, the **14a** derivative was produced from the reaction of **9a** with **13**. The reactivity and stability of formed **14a** will be highlighted from the point of view of DFT calculations. The Molecular electrostatic potential map (MEP) will be discussed as well. The DFT (B3LYP) method with 6-311G++(d,p) basis set to optimize organic structure compounds. Both TDOS and MEP were performed at the same level of theory.

**Fig 15:**
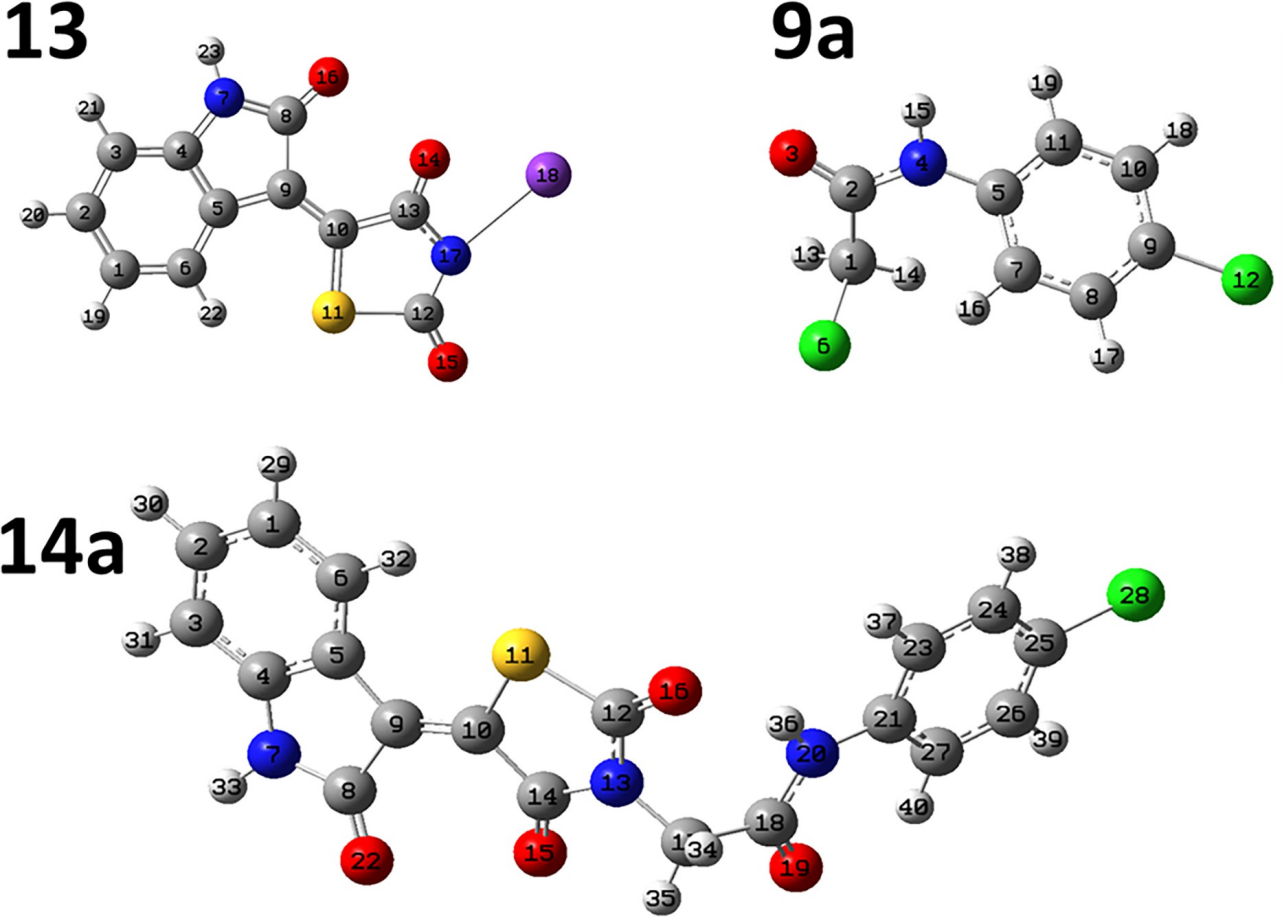
The full optimized structure of 14a, 13 and 9a at B3LYB/6-311G++(d,p) basis set.

The full optimized structure of **14a**, **9a** and **13** are shown in **Fig 15**. The obtained total energy of **14a** system was -55925.2 eV which is higher than those of free systems **13** and **9a**, -47778.3 and -36986.6 eV, respectively. The dipole moment for the **14a** was found to be 8.11 Debye (higher than those of free components, **13**; 7.6Debye; and **9a**; 2.91 Debye). The dipole moment reflects the polarity of the molecule which increases with the increase in electronegativity of atoms. Also, it is related to the electronic distribution in a molecule and the chemical reactivity usually increases with the increase in the dipole moment as the interaction with other systems occurred. The recorded binding energy was 1059.84 eV.

**Fig 16:**
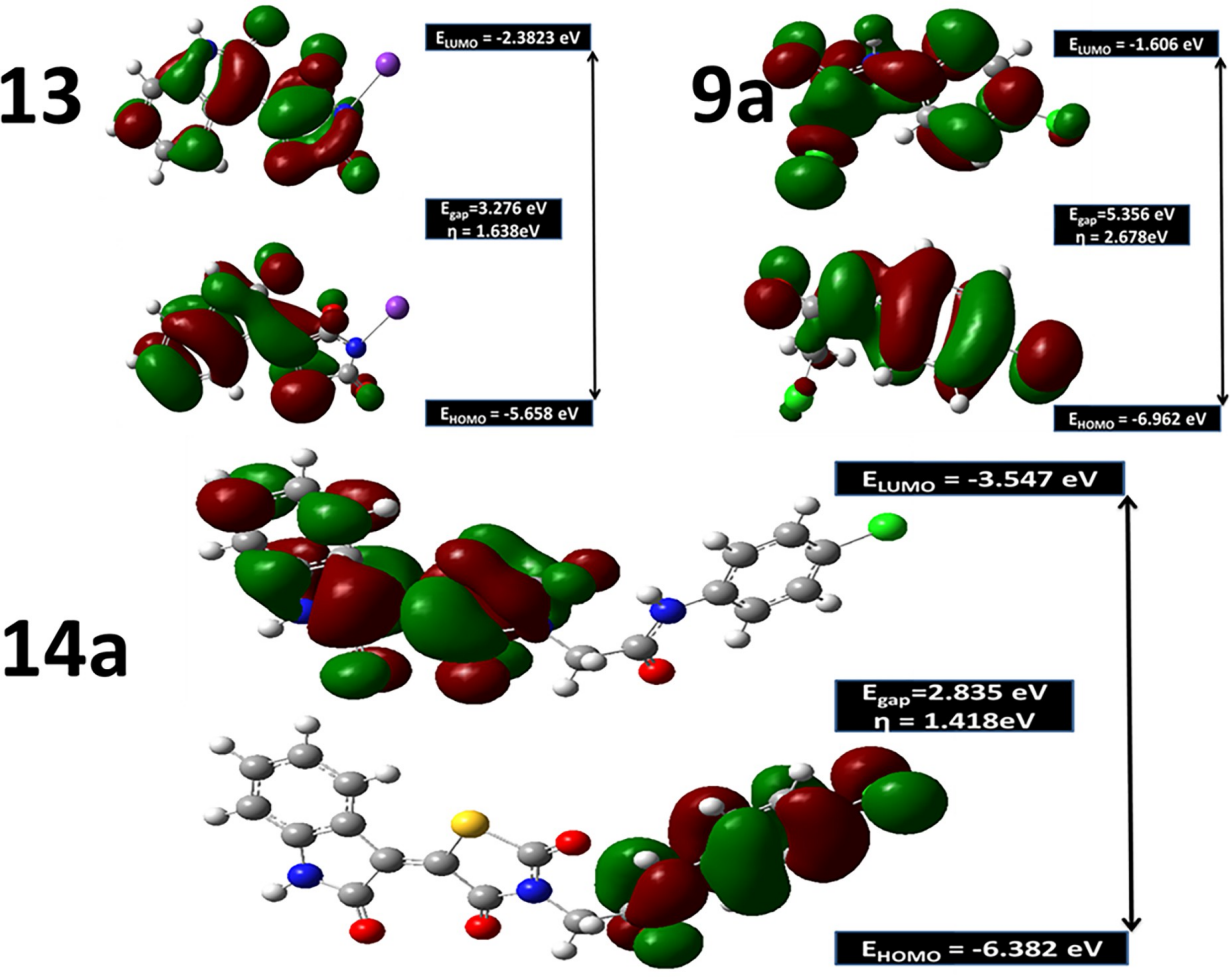
Frontiers molecular orbitals, HOMO, LUMO, energy gaps, ΔE and absolute hardness, η in eV for 14a inhibitor and free individual components, 13 and 9a at B3LYB/6-311++G(d,p) level.

**Fig 17:**
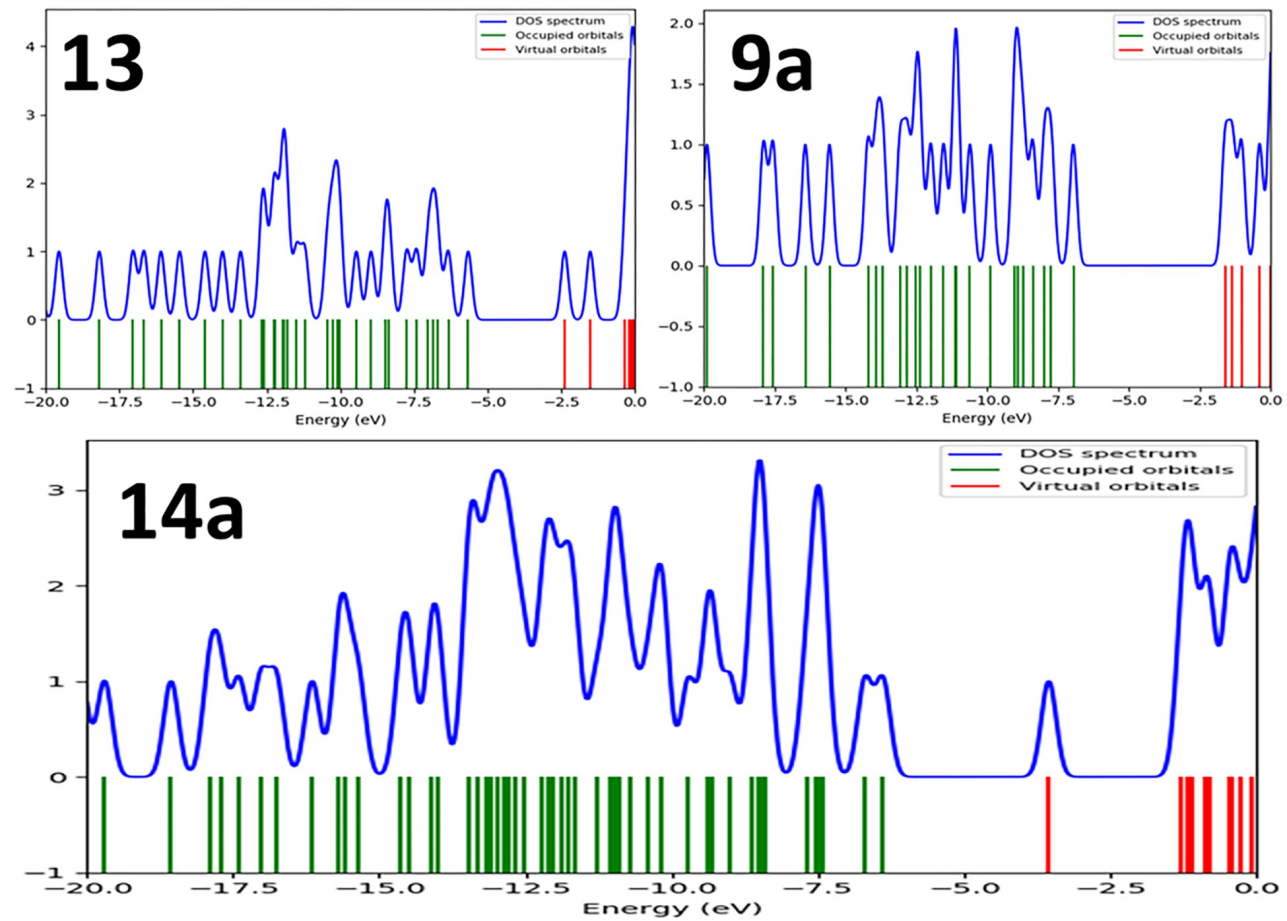
Total density of state, TDOS for 14a inhibitor and free individual components, 13 and 9a at B3LYB/6-311++G(d,p) level.

The energy of the lowest occupied molecular orbital (E_HOMO_); the energy of the highest unoccupied molecular orbital (E_LUMO_) and energy gab, ΔE (the gap between the HOMO and LUMO energy levels) and absolute hardness (η) were calculated and represented in **Fig 16**. The absolute hardness; η ((E_LUMO_-_EHOMO_)/2) measures the molecular stability and reactivity. A hard molecular has a large energy gap while a soft molecule has a small energy gap [50]. The soft molecule is reactive and easily offers electrons to an acceptor. **14a** recorded η value of 1.4 eV as shown in **Fig 16**. In addition, ΔE was found to be 2.835 eV smaller than those recorded for **9** and **13a.** This small ΔE value of **14a** explains its reactivity side by side with proper hardness value which increases its tendency to be a good inhibitor towards VEGFR-2. As demonstrated in **Fig 16**, the electron density in HOMO is localized on 4-chlorophenyl acetamide moiety while in LUMO, the density is centered over the 5-(2-oxoindolin-3-ylidene)thiazolidine-2,4-dione moiety. The total density state confirmed the previous findings as the small energy gap of **14a** was noticed in the same value as presented in **Fig 17**. As the LUMO-HUMO energy gap decreases, the interactions between the reactants species, **13** and **9a** become stronger which results in a stable inhibitor structure.

**Fig 18:**
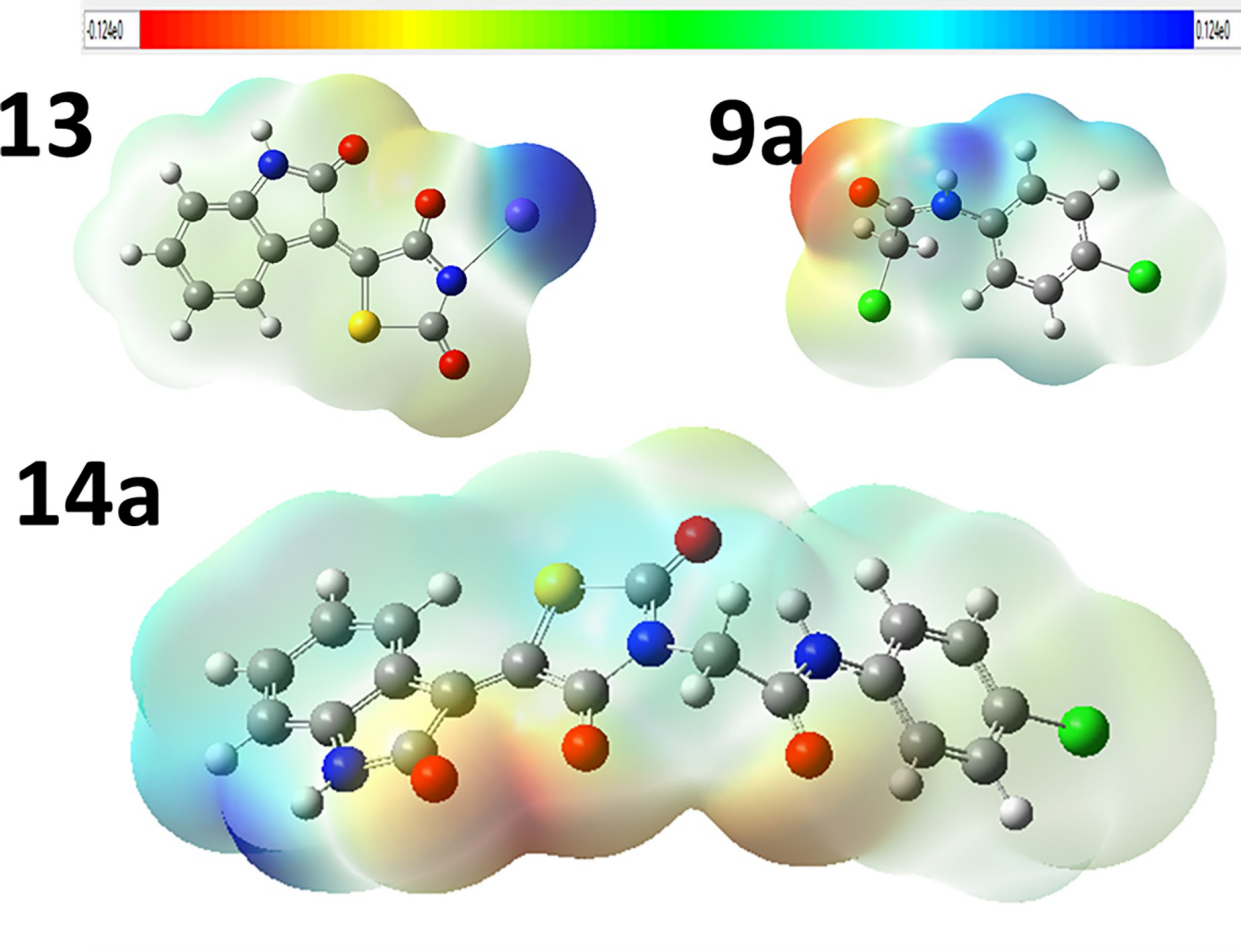
Molecular electrostatic potentials for 14a inhibitor and free individual components, 13 and 9a at B3LYB/6-311++G(d,p) level of theory.

Molecular electrostatic potential (MEP) illustrates the electronic charge distributions of molecules three dimensionally. MEP maps visualize variably charged zones of a molecule. Knowledge of the electron charge distributions can be used to explain how molecules interact with one another. The strength of the electrostatic potentials is well represented by the MEP surface in **Fig 18**. where red and blue regions mention the most electronegative electropositive zones, respectively. As shown in **Fig 18**, the oxygenated groups in all compounds have red color because these groups have negative electrostatic potential. For **14a**, oxygen atoms will behave as nucleophiles while blue regions at hydrogen atoms, mostly, behave as electrophiles [51].

## 3. Conclusion

A new series of thiazolidine-2,4-diones derivatives were designed and synthesized as potential anticancer agents targeting VEGFR-2. These derivatives were tested for their anticancer efficacy against A549, Caco-2, HepG-2, and MDA-MB-231 cell lines. The most active antiproliferative member was found to be compound **14a** (IC_50_ = 1.5 and 31.5 μM) against Caco-2 and HepG2 cell lines, respectively. In addition, when compared to the reference drug, sorafenib, kinase inhibition assay results revealed that all compounds had good inhibitory activity against VEGFR-2. Further, because of its high selectivity index, derivative **14a** was chosen for further testing of its effect on Caco-2 cell migration and alternation of Caco-2 cells gene expression. Compound **14a** significantly inhibited the ability of cancer cells to migrate and heal, according to a cell migration assay. Additionally, the ability of **14a** to downregulate Bcl-2, Survivin, and TGF expression levels was discovered in a subsequent biological assay. The ability of **14a** to recognize the ATP binding pocket of VEGFR-2 and elicit significant interactions with its key amino acids was demonstrated using molecular docking as well as several MD simulations studies. Also, the DFT calculations have been performed for **14a** inhibitor and free individual components, **13** and **9a** at B3LYB/6-311++G(d,p) level of theory. The results conducted that **14a** showed large stabilization due to the strong interaction forces within the molecule and small absolute hardness.

## 4. Experimental

### 4.1. Chemistry

#### 4.1.1. General

The used chemical agents and devices in the synthesis procedures were described in **S.3 Section in S1 File**. Compounds **10a**, **b** and **14a**-**c** were furnished following the reported procedures [30, 31, 33, 34]. In **Table 7**, the colors, yields, and meting points of the new compounds were presented. The ^1^H NMR and ^13^C NMR analyses were carried out at 400 and 100 MHz, respectively in DMSO-d6 as a solvent. the chemical shifts were presented as ppm. The infra-red analyses were carried out using KBr disc and the results were presented as cm-1.

**Table 7:** The colors, yields, and meting points of the new compounds.

Compounds	Color	Yield	Meting points (°C)
**10a**	White powder	74%	265–267
**10b**	Yellow powder	70%	234–236
**14a**	White crystals	76%	224–226
**14b**	Yellow powder	78%	230–232
**14c**	White powder	80%	250–252

#### 4.1.2. General synthesis pathway compounds 10a,b

**Fig 19:**
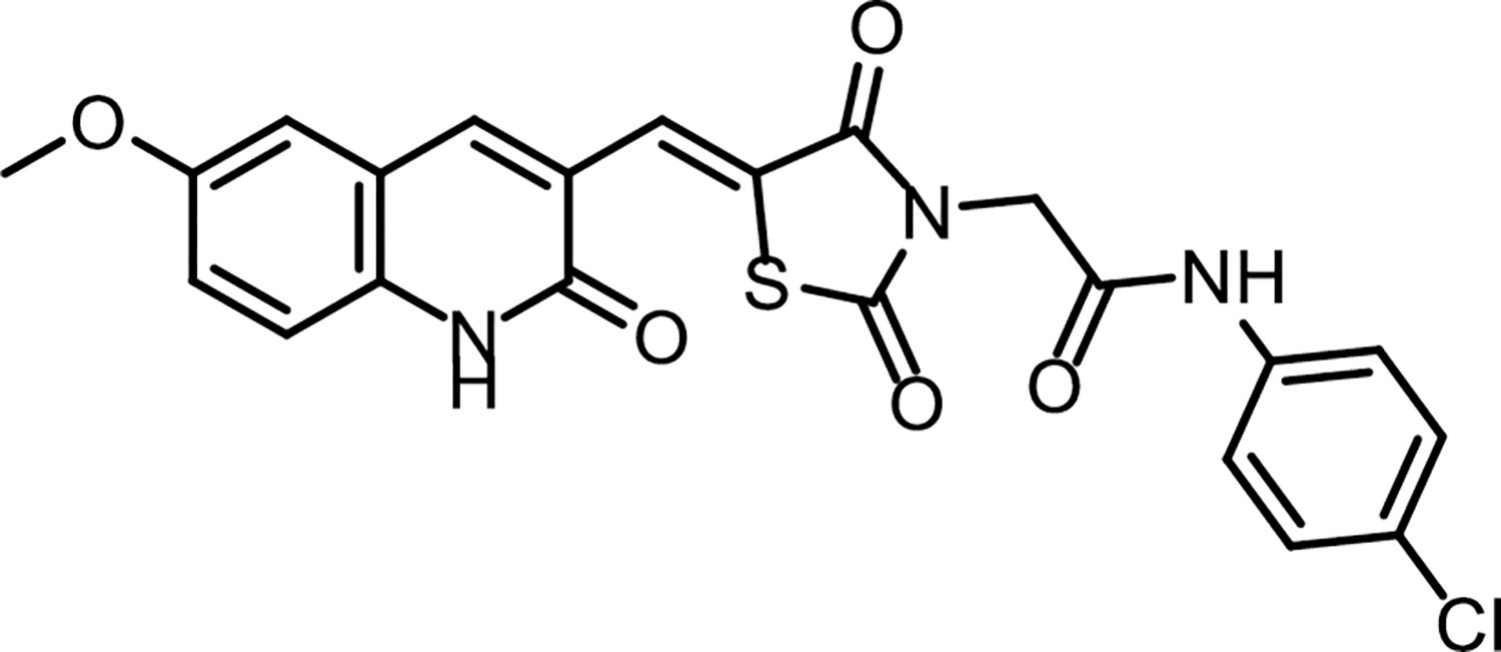
Chemical structure of compound 10a.

**Fig 20:**
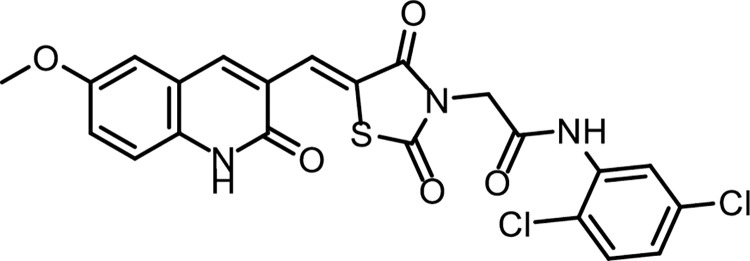
Chemical structure of compound 10b.

Compound **7** (0.001 mol) was heated with K_2_CO_3_ in a dry DMF for 30 min. till formation of the corresponding *in situ* potassium salt **8**. Then, the appropriate 2-chloro-*N*-substituted acetamide derivatives **9a**, **b** (0.001 mol), and KI (0.001 mol) in DMF (10 ml) was added to the previous mixture with reflux on a water bath for 6 h. The reaction mixture was poured on crushed ice. The precipitate was filtered, dried, and crystallized from ethanol to give the corresponding target compounds **10a**, **b** (**Figs 19 & 20**).

*4*.*1*.*2*.*1*. *(Z)-N- (4-Chlorophenyl) -2- (5-((6-methoxy-2-oxo-1*,*2-dihydroquinolin-3-yl) methylene) -2*,*4-dioxothiazolidin-3-yl)acetamide (10a)*. IR: 3449, 3265 (NH), 3005 (CH aromatic) 2932 (CH aliphatic), 1724, 1673 (C = O); ^1^H NMR: 10.79 (s, 1H), 10.24 (s, 1H), 8.45 (s, 1H), 8.21 (s, 1H), 8.00 (d, *J* = 14.0 Hz, 2H), 7.62 (d, *J* = 8.9 Hz, 2H), 7.43 (m, 2H), 7.39 (d, *J* = 2.3 Hz, 1H), 4.54 (s, 2H), 3.81 (s, 3H); ^13^C NMR: 171.63, 161.56, 160.73, 157.08, 155.01, 154.98, 142.25, 137.60, 136.51, 130.14, 130.08, 129.64, 127.02, 126.19, 124.16, 120.06, 119.61, 119.16, 117.30, 111.56, 56.03, 21.16. C_22_H_16_ClN_3_O_5_S (469.90).

*4*.*1*.*2*.*2*. *(Z)-N-(2*,*5-Dichlorophenyl)-2-(5-((6-methoxy-2-oxo-1*,*2-dihydroquinolin-3-yl) methylene)-2*,*4-dioxothiazolidin-3-yl)acetamide (10b)*. IR: 3264 (NH), 3098 (CH aromatic) 2997, 2931 (CH aliphatic), 1753, 1688 (C = O); ^1^H NMR: 10.67 (s, 1H), 10.26 (s, 1H), 8.55 (s, 1H), 8.40 (s, 1H), 7.95 (d, *J* = 8 Hz, 2H), 7.71 (m, 2H), 7.49 (d, *J* = 8.2 Hz, 1H), 7.29–7.20 (m, 1H), 4.54 (s, 2H), 3.81 (s, 3H). C_22_H_15_Cl_2_N_3_O_5_S (504.34).

#### 4.1.3. General synthesis pathway compounds 14a-c

**Fig 21:**
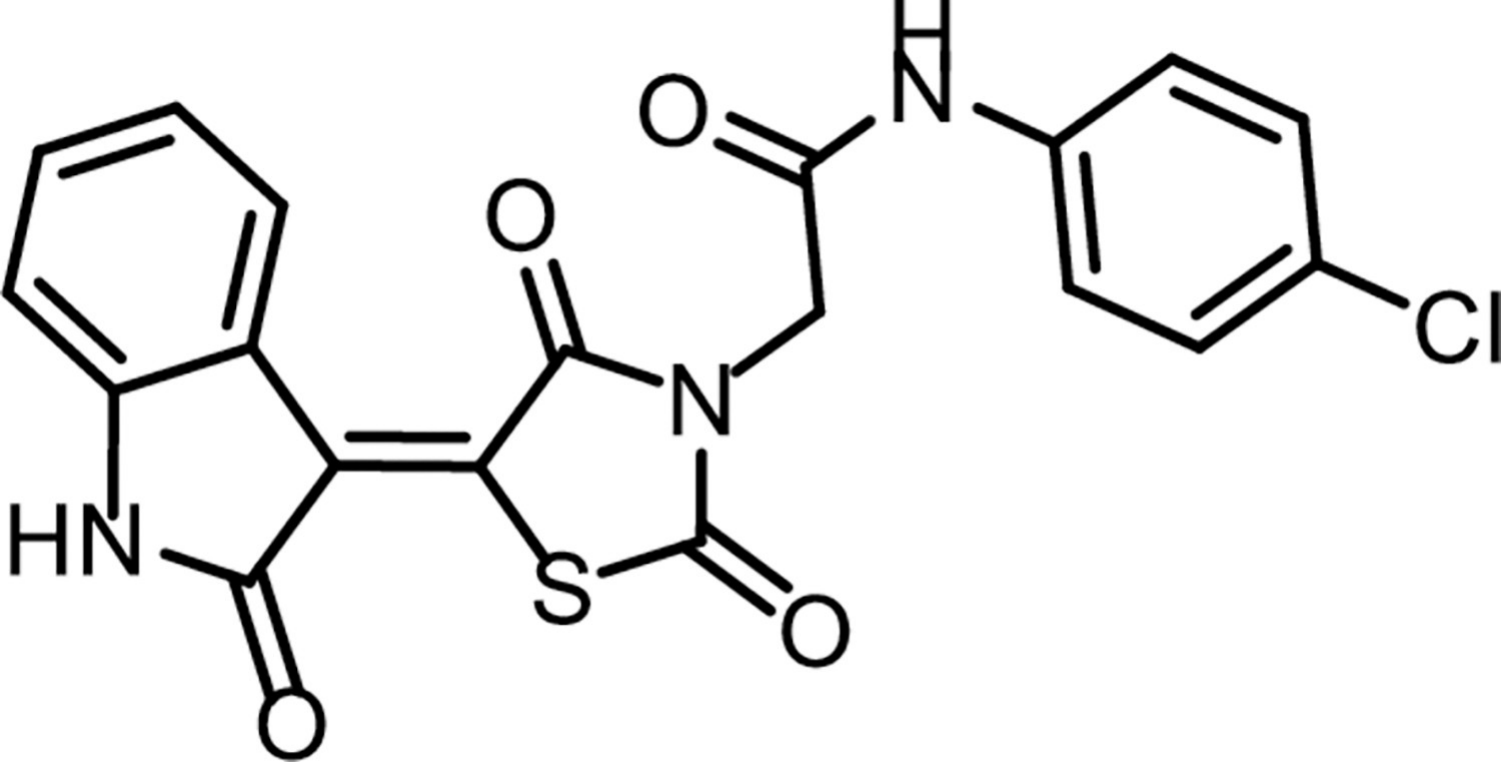
Chemical structure of compound 14a.

**Fig 22:**
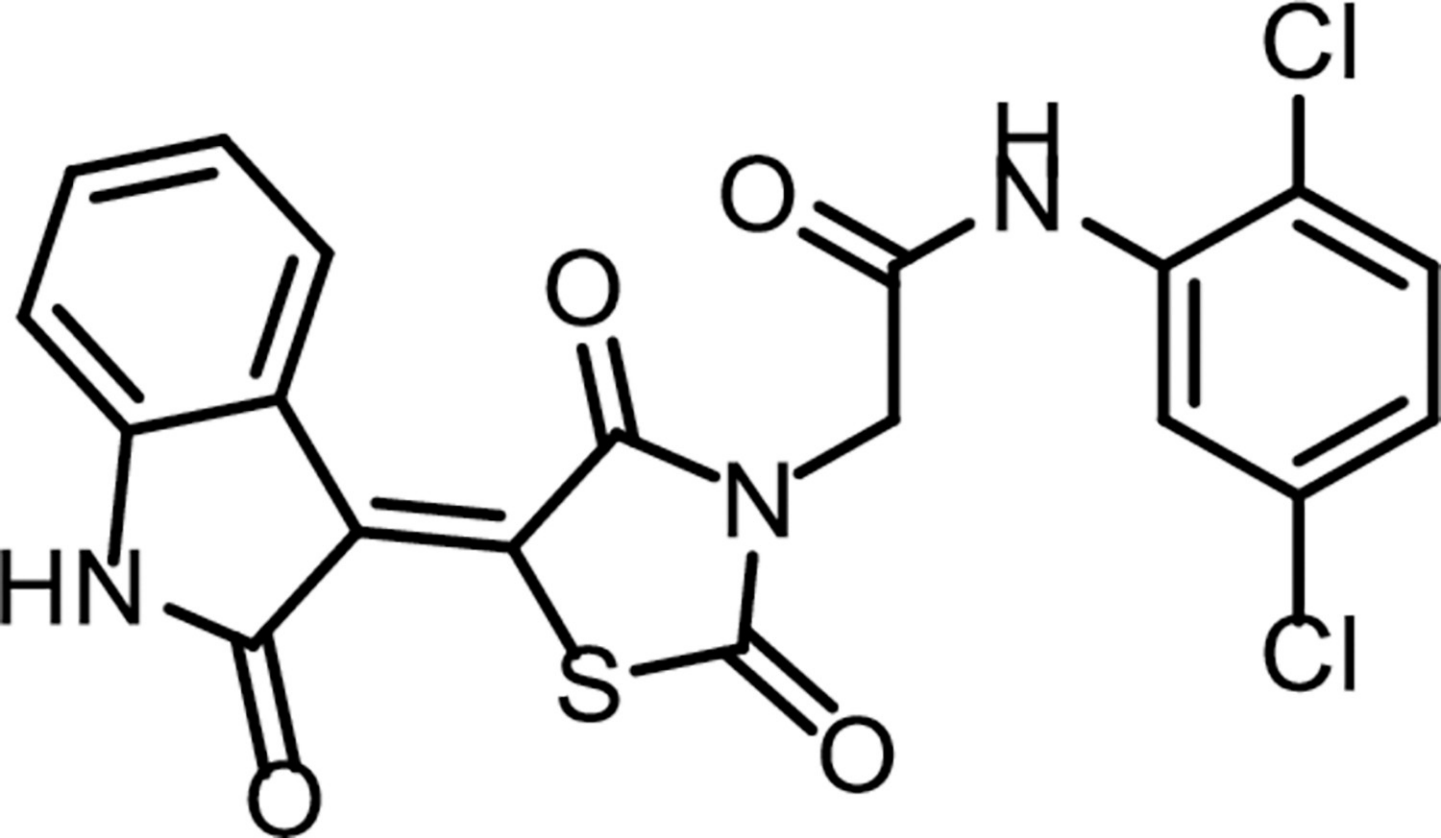
Chemical structure of compound 14b.

**Fig 23:**
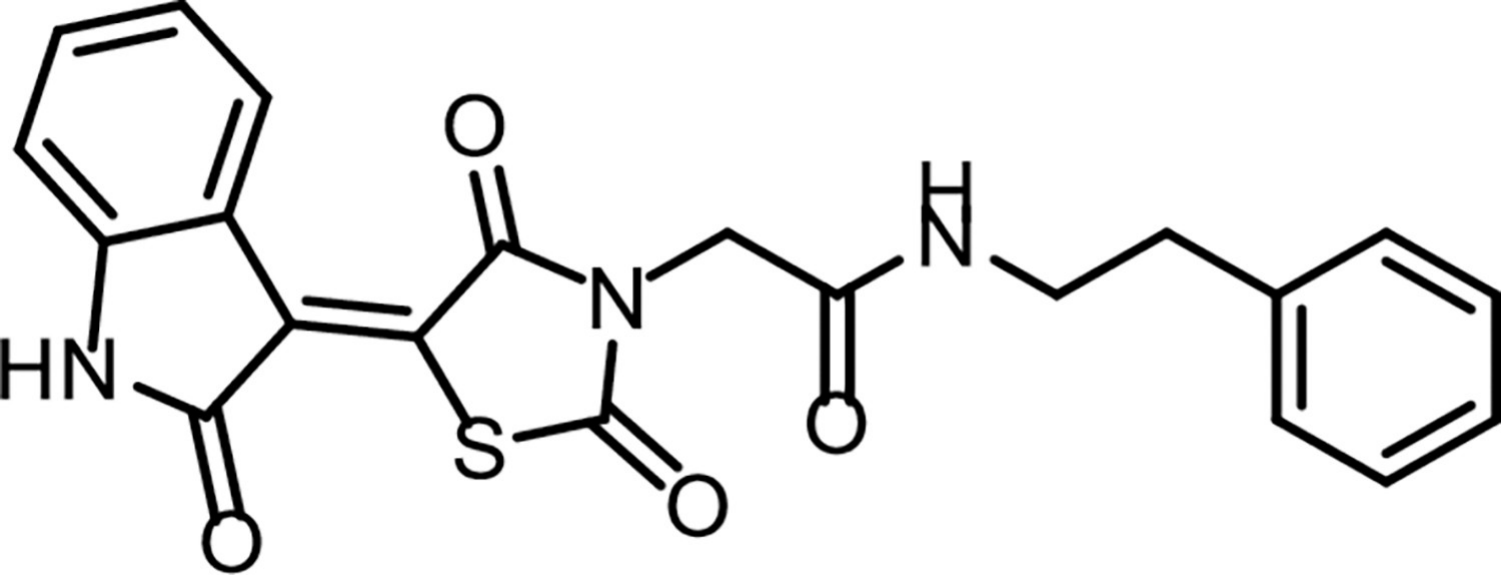
Chemical structure of compound 14c.

Compound **12** (0.001 mol) was heated with K_2_CO_3_ in a dry DMF for 30 min. till formation of the corresponding *in situ* potassium salt **13**. Then, the appropriate 2-chloro-*N*-substitutedacetamide derivatives **9a-c** (0.001 mol), and KI (0.001 mol) in DMF (10 ml) was added to the previous mixture with reflux on a water bath for 6 h. The reaction mixture was poured on crushed ice. The precipitate was filtered, dried, and crystallized from ethanol to give the corresponding target compounds **14a-c (Figs 21–23)**.

*4*.*1*.*3*.*1*. *N-(4-Chlorophenyl)-2-(2*,*4-dioxo-5-(2-oxoindolin-3-ylidene)thiazolidin-3-yl)acetamide (14a)*. IR: 3185, 3142 (NH), 3062 (CH aromatic), 1745, 1690 (C = O); ^1^H NMR: 11.32 (s, 1H), 10.58 (s, 1H), 8.78 (s, 1H), 7.60 (d, J = 8.5 Hz, 2H), 7.48–7.37 (m, 3H), 7.09 (t, J = 7.8 Hz, 1H), 6.99 (d, J = 7.8 Hz, 1H), 4.58 (s, 2H); ^13^C NMR: 170.23, 168.72, 165.68, 164.47, 144.64 (2C), 137.78, 133.59, 129.31 (2C), 128.43, 128.25, 127.83, 122.68, 121.28 (2C), 120.22, 111.17, 44.13. C_19_H_12_ClN_3_O_4_S (413.83).

*4*.*1*.*3*.*2*. *N-(2*,*5-Dichlorophenyl)-2-(2*,*4-dioxo-5-(2-oxoindolin-3-ylidene)thiazolidin-3-yl)acetamide (14b)*. IR: 3211, 3176 (NH), 3065 (CH aromatic) 2994, 2949 (CH aliphatic), 1744, 1696 (C = O); ^1^H NMR: 11.32 (s, 1H), 10.24 (s, 1H), 8.78 (d, *J* = 7.9 Hz, 1H), 7.88 (d, *J* = 2.5 Hz, 1H), 7.58 (d, *J* = 8.7 Hz, 1H), 7.44 (t, *J* = 7.7 Hz, 1H), 7.31 (dd, *J* = 8.6, 2.6 Hz, 1H), 7.12 (d, *J* = 7.8 Hz, 1H), 6.99 (d, *J* = 7.8 Hz, 1H), 4.70 (s, 2H).; ^13^C NMR: 170.26, 168.72, 165.70, 163.97, 144.60, 136.31, 133.56, 133.24, 129.74 (2C), 129.40, 128.42, 128.17, 122.67, 120.21, 119.71 (2C), 111.15, 44.06. C_19_H_11_Cl_2_N_3_O_4_S (448.27).

*4*.*1*.*3*.*3*. *2-(2*,*4-Dioxo-5-(2-oxoindolin-3-ylidene)thiazolidin-3-yl)-N-phenethylacetamide (14c)*. IR: 3303, 3179 (NH), 3062 (CH aromatic) 2935, 2886 (CH aliphatic), 1744, 1691 (C = O); ^1^H NMR: 11.31 (s, 1H), 8.82 (dd, *J* = 18.2, 7.0 Hz, 2H), 7.45 (t, *J* = 7.7 Hz, 1H), 7.35 (t, *J* = 7.5 Hz, 2H), 7.28 (d, *J* = 7.7 Hz, 3H), 7.12 (t, *J* = 7.8 Hz, 1H), 6.99 (d, *J* = 7.8 Hz, 1H), 4.40 (s, 2H), 4.34 (t, 2H), 3.40 (t, 2H); ^13^C NMR: 170.30, 168.74, 165.75, 165.61, 144.54, 139.31, 133.48, 129.88, 128.82 (2C), 128.39, 127.89, 127.66 (2C), 127.42, 122.67, 120.24, 111.14, 43.66, 42.75, 31.18. C_21_H_17_N_3_O_4_S (407.44).

### 4.2. Biological testing

#### 4.2.1. *In vitro* anti-proliferative activity

The synthesized compounds were evaluated for their anti-proliferative activities against A549, Caco-2, HepG2, and MDA-mb-231 cell lines using the MTT assay protocol [52–54] as described in **S.1.2. Section in S1 File.**

#### 4.2.2. *In vitro* VEGFR-2 inhibition

VEGFR-2 ELISA kit was used in this test as described in **S.1.3. Section in S1 File** [24, 55, 56].

#### 4.2.3. Safety assay

The safety profiles of the tested compounds were checked on one non-cancerous cell line (Vero) to determine the treatments concentrations that do not depict toxic effects against the tested cells as described in **S.1.1. Section in S1 File** [57].

#### 4.2.4. Selectivity index (SI)

The selectivity index values of the tested compounds on cancer cells were calculated as described (**S.1.4 Section in S1 File**) [58].

#### 4.2.5. Cell Migration assay

Cell Migration assay was conducted according to the reported protocol as described [59]in **S.1.5. Section in S1 File.**

#### 4.2.6. Gene expression pattern

The molecular anticancer mode of action of **14a** was investigated by screening their ability to control the gene expression levels of Bcl2, Bcl-xl, TGF and Survivin genes as reported [60] in **S.1.6. Section in S1 File.**

### 4.3. *In silico* studies

#### 4.3.1. Docking studies

MOE2019 software was used to perform docking studies against VEGFR-2 [PDB ID: 4ASD] [61–65] as described in **S.2.1. Section in S1 File.**

#### 4.3.2. ADMET studies

Discovery studio 4.0 was used to perform ADMET studies as reported in before [43, 65–69] (**S.2.2. Section in S1 File**).

#### 4.3.3. Toxicity studies

Discovery studio 4.0 was used to carry out the toxicity studies as described [18, 70–72] in **S.2.3. Section in S1 File**.

#### 4.3.4. Molecular dynamics simulation & MM/PBSA

MD simulation experiments and MM/PBSA (Molecular Mechanics/Poisson Boltzmann Surface Area) were carried out using GROMACS as reported in **S.2.4 & S.2.5. Sections in S1 File** [67, 73–75].

#### 4.4.4. Density Function Theory (DFT) calculations

The DFT calculations were performed using Gaussian 09 software and the output files were visualized using GaussianView5. Total density of state (TDOS) was calculated and analyzed using GaussSum software. Chem3D 15 was used to draw the original chemical structures of all compounds. The DFT (B3LYP) method with 6-311G++(d,p) basis set to optimize organic structure compounds.

## Supporting information

S1 File Supporting information related to this manuscript is found in a separate file. (PDF)
