# Supplementary material for: Design, synthesis, anti-proliferative evaluation, docking, and MD simulations studies of new thiazolidine-2,4-diones targeting VEGFR-2 and apoptosis pathway
Source: PLoS One. 2022 Sep 23;17(9):e0272362. doi: 10.1371/journal.pone.0272362 (PMC9506633; doi:10.1371/journal.pone.0272362)
Supplement: S1 File — (PDF) [file pone.0272362.s001.pdf]

Design, synthesis, anti-proliferative evaluation, docking, and MD simulations studies of new thiazolidine-2,4-diones targeting VEGFR-2 and apoptosis pathway

Mohammed S. Taghour[a], Hazem Elkady[a], Wagdy M. Eldehna[b], Nehal El-Deeb[c,d], Ahmed M. Kenawy [e], Eslam B. Elkaeed [f], Bshra A. Alsfouk [g], Mohamed S. Alesawy[a], Dalal Z. Husein[h], Ahmed M. Metwaly [i,d]* Ibrahim H. Eissa[a]*

[a] Pharmaceutical Medicinal Chemistry & Drug Design Department, Faculty of Pharmacy (Boys), Al-Azhar University, Cairo11884, Egypt.

[b] Department of Pharmaceutical Chemistry, Faculty of Pharmacy, Kafrelsheikh University, P.O. Box 33516, Kafrelsheikh, Egypt.

[c] Biopharmaceutical Products Research Department, Genetic Engineering and Biotechnology Research Institute, City of Scientific Research and Technological Applications (SRTA-City), Alexandria 21934, Egypt.

[d] Pharmaceutical and Fermentation Industries Development Center, City of Scientific Research and Technological Applications (SRTA city), Alexandria 21934, Egypt.

[e] Nucleic Acids Research Department, Genetic Engineering and Biotechnology Research Institute. City of Scientific Research and Technological Applications (SRTA-City). Alexandria 21934, Egypt.

[f] Department of Pharmaceutical Sciences, College of Pharmacy, AlMaarefa University, Riyadh 13713, Saudi Arabia.

[g] Department of Pharmaceutical Sciences, College of Pharmacy, Princess Nourah bint Abdulrahman University, P.O. Box 84428, Riyadh 11671, Saudi Arabia.

[h] Chemistry Department, Faculty of Science, New Valley University, El-Kharja 72511, Egypt.

[i] Pharmacognosy and Medicinal Plants Department, Faculty of Pharmacy (Boys), Al-Azhar University, Cairo 11884, Egypt.

***Corresponding authors:**

Ibrahim H. Eissa

Medicinal Chemistry Department, Faculty of Pharmacy (Boys), Al-Azhar University, Cairo 11884, Egypt. **Email:** Ibrahimeissa@azhar.edu.eg

Ahmed M. Metwaly

Pharmacognosy and Medicinal Plants Department, Faculty of Pharmacy (Boys), Al-Azhar University, Cairo 11884, Egypt. **Email:** ametwaly@azhar.edu.eg

Content

**S.1**	**Biological testing**<br><br>S.1.1. Safety assay<br>S.1.2. *In vitro* anti-proliferative activity.<br>S.1.3. *In vitro* VEGFR-2 kinase assay.<br>S.1.4. Selectivity index (SI)<br>S.1.5. Wound healing assay (Migration assay).<br>S.1.6. Gene expression pattern alternation of cancer cell after **14a** treatment.
**S.2**	***In silico* studies**<br><br>S.2.1. Docking studies<br>S.2.2. ADMET studies<br>S.2.3. Toxicity studies<br>S.2.4. MD simulation<br>S.2.5. MMPBSA
**S.3**	**Chemistry and materials**
**S.4**	**Spectral data**
**S.5**	**Raw data for biological testing**
**S.6**	***In silico* cytotoxicity report**

S.1. Biological testing

Mammalian cell lines culture

CaCo-2, and A549 cell lines were cultured on DMEM media, meanwhile MDA-MB-231 and hepG-2 cell line were cultured on RBMI media. The cultured media were supplemented with 200 mM L-glutamine, 10.0% fetal bovine serum (Lonza), and 1.0% penicillin/streptomycin. Cells were seeded into 25.0 cm tissue culture flasks and incubated at 37°C in a 5.0% CO_2 incubator for 24 h or till confluency.

S.1.1. Safety assay

The safety profiles of the tested compounds were checked on one non-cancerous cell line (Vero) to determine the treatments concentrations that do not depict toxic effects against the tested cells. A portion of 100.0 µl of 6×10^4 cell/ml cells was seeded into each well of a 96-well plate and then the plates were incubated at 37°C in a humidified 5.0% CO_2 incubator for 24 h. At the end of incubation period, the exhausted medium was replaced with 100.0 µl of different concentrations of the designated treatment (prepared in RPMI medium starting from 1.0 mM). The inoculated plates were incubated at the same growth conditions for another 24 h. At the end of incubation, cellular viability was assessed using MTS assay kit (Promega) according to the manual instruction.

S.1.2. In-vitro anticancer activity

Anticancer activities of the tested compounds against CaCo-2, MDA-MB-231, and hepG-2 cell lines were quantified using MTS assay kit (Promega) as described by the Manufacturer.

S.1.3. In vitro VEGFR-2 kinase assay

All the synthesized compounds were tested for its inhibitory activity against VEGFR-2. Human VEGFR-2 ELISA kit (Enzyme-Linked Immunosorbent Assay) was utilized in this test. At first, specific antibody for VEGFR-2 was seeded on a 96-well plate and 100 µL of the standard solution or the tested compound was added, all were incubated at room temperature for 2.5 h. Then washed, 100 µL of the prepared biotin antibody was added, then incubated at room temperature for additional 1 h. and washed. Then, 100 µL of streptavidin solution was added and incubated for 45 min. at room temperature. Washed again, 100 µL of TMB Substrate reagent was added and incubated for 30 min. at room temperature. 50 µL of the stop solution was added, then read at 450 nm immediately. The standard curve was drawn, concentrations on the X-axis and the absorbance on the Y-axis.

S.1.4. Selectivity index (SI)

The selectivity index values of the tested compounds on cancer cells were calculated as described by Koch et al. [57], with slight modifications; SI=IC_{50}nc/IC_{50}cc, where IC_{50}nc: the IC_{50} value of the tested compound on normal cells and IC_{50}cc: IC_{50} of the tested compound on cancer cell line**.**

S.1.5. Wound healing assay (Migration assay)

CaCo-2 cells were grown to 95.0% confluency in a complete DMEM medium and then the wounds were formed using a plastic tip. After washing with pre-warmed PBS, the cells were incubated in the specific medium or the **14a** treatment. After incubation at 37°C and 5.0% CO_2 for 24h, the cells were washed with PBS and the wounds distance was determined as the scratch width of the treated and untreated groups using ImageJ software.

S.1.6. **Gene expression pattern alternation of cancer cell after 14a treatment**

The molecular anticancer mode of action of **14a** was investigated by screening their ability to affect the gene expression levels of Bcl2, Bcl-xl, TGF and Survivin genes using specific forward and reverse primers and RTq-PCR technique (Table 1) in CaCo-2 cells (chosen as the most sensitive cancer cell line). After cellular treatment, CaCo-2 cell line was cultured into 12 well plates (6×10^3 cell/ml) for 24 h. with the sub-IC50 concentration of **14a**. After treatment, total RNA extraction was performed using RNA extraction kit (Qiagen, Germany). Then, 1 ug of the obtained RNA was used to synthesiz cDNA using cDNA synthesis kit (Promega Corp., Madison, WI) as recommended by the manufacturer. Simultaneously, GAPDH forward and reverse primers (Table 1) were used to amplify the house keeping gene as internal control for standardization of PCR products. The RTq-PCR was done using SYBR Green dye (QuantiTect SYBR Green PCR Kits) and Light Cycler fluorimeter (Bio-RAD S1000 Tm thermal cycler). The PCR cycling program was as follows: 95ºC for 2 min, followed by 40 cycles of 95ºC for 30s, 55ºC for 30 s, and 60ºC for 45s, and finally 60ºC for 5 min.

-Sequence of the primers

Primer ID	Sequence
Bcl-F	5′-TATAAGCTGTCGCAGAGGGGCTA-3′
Bcl-R	5′-GTACTCAGTCATCCACAGGGCGAT-3′
Bcl-Xlf	5’CAGAGCTTTGAACAGGTAG-3′
Bcl-XlR	5’GCTCTCGGGTGCTGTATTG-3′

Surv-F	5′-TGCCCCGACGTTGCC-3′
Surv-R	5′-CAGTTCTTGAATGTAGAGATGCGGT-3′
TGF-F	5′CAAGGGCTACCATGCCAACT3′
TGF-R	5′AGGGCCAGGACCTTGCTG3′
β-actin-F	5′-GTGGGGCGCCCCAGGCACCA-3′
β-actin-R	5′-CTCCTTAATGTCACGCACGATTTC-3′

S.2. ***In silico* studies**

S.2.1. Docking studies

The docking studies were performed utilizing MOE.14 software to explore the binding mode of the synthesized compounds towards VEGFR-2. The 3D crystal structures of the target macromolecules VEGFR-2 were downloaded from the protein databank, http://www.pdb.org (PDB ID; 2OH4 and 4ASD). Sorafenib was used as reference ligand. To prepare the target protein, water molecules were removed, and the valances of atoms were corrected through protonation of the whole molecule. Then energy minimization was carried out by applying CHARMM and MMFF94 force fields. After that, the active binding site was defined and prepared for docking. The validation process was performed by redocking the co-crystallized ligand. The designed compounds together with sorafenib were drawn using ChemBioDraw Ultra 14.0 and saved as MDL-SD format. The sketched compounds were constructed from fragment libraries in MOE program, protonated, followed by energy minimization then prepared for docking. Docking process was carried through Triangle matcher placement inserted in compute window, and the scoring function was London dG. Ten conformers (poses) for each molecule were generated using genetic algorithm searches. The free energies and binding modes of the designed molecules against VEGFR-2 were determined. The most ideal pose was selected according to its binding free energy as well as its binding mode with target molecule.

S.2.1.1. The predicted binding pattern of **14b** with the active site of VEGFR-2

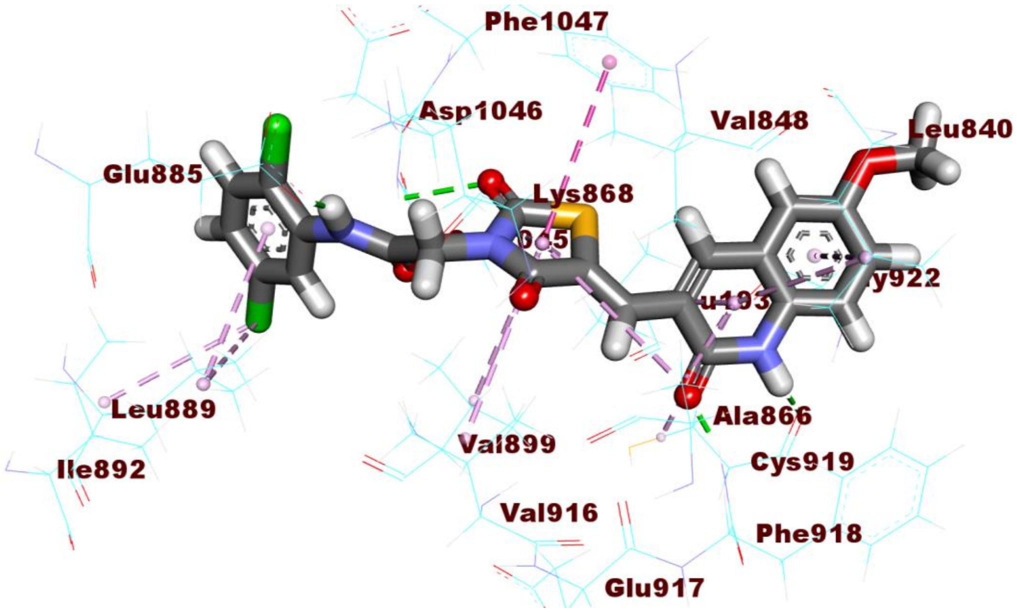

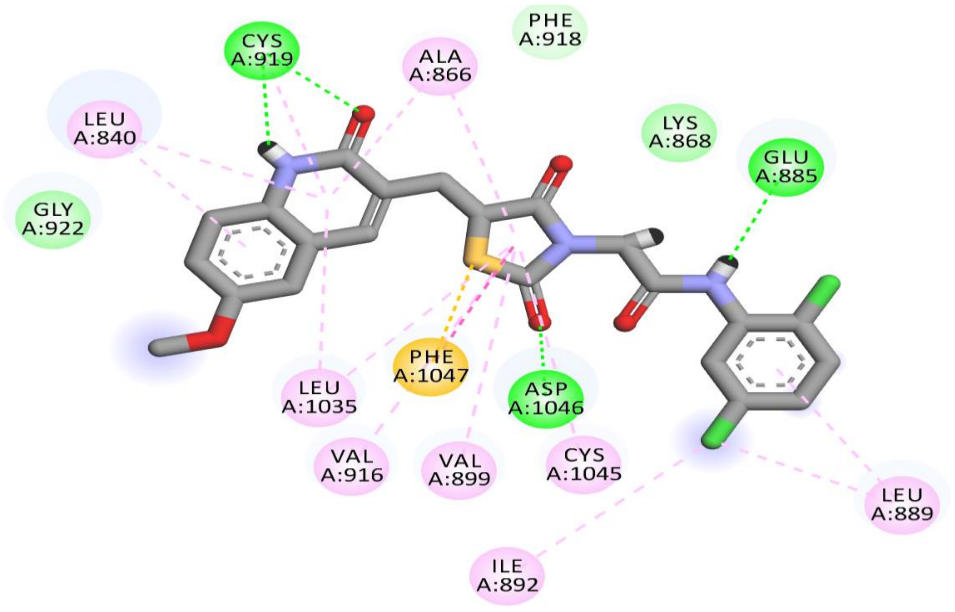

S2.1.2. The predicted binding pattern of **14c** with the active site of VEGFR-2

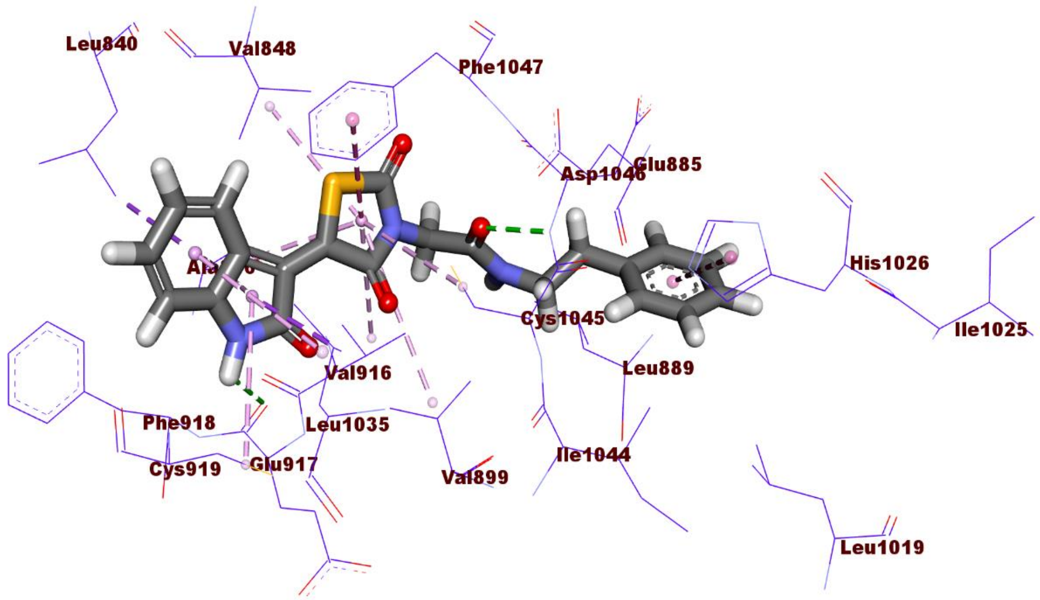

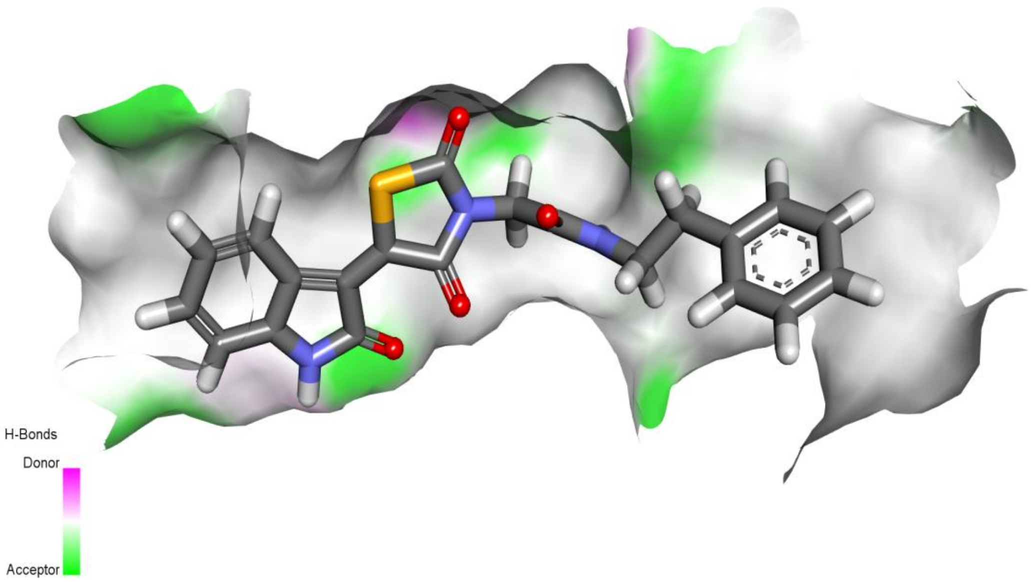

S.2.2. ADMET studies

ADMET descriptors (absorption, distribution, metabolism, excretion and toxicity) of the synthesized compounds were determined using Discovery studio 4.0. At first, the CHARMM force field was applied then the compounds were prepared and minimized according to the preparation of small molecule protocol. Then ADMET descriptors protocol was applied to carry out these studies.

S.2.3. Toxicity studies

The toxicity parameters of the synthesized compounds were calculated using Discovery studio 4.0. Sorafenib was used as a reference drug. At first, the CHARMM force field was applied then the compounds were prepared and minimized according to the preparation of small molecule protocol. Then different parameters were calculated from toxicity prediction (extensible) protocol.

S.2.4. Molecular dynamics simulation

Molecular dynamics simulation of the protein-ligand complexes was performed using GROMACS 2021 and Linux 5.4 package. The GROMOS96 54a7 forcefield was selected as the force field for proteins and the ligand topologies were generated from the PRODRG server. All the complexes were solvated using simple point charge (SPC) water molecules in a rectangular box. To make the simulation system electrically neutral, required number of Na^+ and Cl^- ions were added while 0.15 mol/L salt concentrations were set in all the systems. Using the steepest descent method, all the solvated systems were subjected to energy minimization for 5000 steps. Afterwards, NVT (constant number of particles, volume, and temperature) series, NPT (constant number of particles, pressure, and temperature) series, and the production run were conducted in the MD simulation. The NVT and the NPT series were conducted at a 300 K temperature and 1 atm pressure for the duration of 300 ps. V-rescale thermostat and Parrinello-Rahman barostat were selected of the performed simulation. Finally, the production run was performed at 300 K for a duration of 100 ns (nanoseconds). Thereafter, a comparative analysis was performed measuring root mean square deviation (RMSD), root mean square fluctuation (RMSF), radius of gyration (Rg), solvent accessible surface area (SASA) and hydrogen bonds to analyze their stability. The Xmgrace program was used to represent the analyses in the form of plots.

S.2.5. MM/PBSA

The g_mmpbsa package of GROMACS was utilized to calculate the MM/PBSA (Molecular Mechanics/Poisson Boltzmann Surface Area) binding free energies followed by final MD production run to get a detailed overview of the molecular interactions between the protein and ligand. The free solvation energy (polar and nonpolar solvation energies) and potential energy (electrostatic and Van der Waals interactions) of each protein-ligand complex were analyzed to determine the total ΔG_{bind} of the complex. The binding energies were calculated using the following equation in this method:

$$\Delta G_{binding} = G_{complex} - (G_{protein} + G_{ligand})$$

Here, the $\Delta G_{binding}$ = the total binding energy of the protein-ligand complex, $G_{protein}$ = the binding energy of free protein, and G_{ligand} = the binding energy of unbounded ligand.

S.3. Chemistry and material

Melting points were measured with a Stuart melting point apparatus and were uncorrected. Infrared (IR) spectra were recorded as KBr disks using an FT-IR Spectrum BX apparatus (Perkin Elmer, CT, USA). NMR spectra were recorded on a Bruker NMR spectrometer (Bruker, Reinstetten, Germany). ^{1}H spectra were run at 500 MHz and ^{13}C spectra were run at 125 MHz in deuterated dimethyl sulfoxide (DMSO-d_6). Chemical shifts are expressed in values (ppm) using the solvent peak as an internal standard. All coupling constant (J) values are given in Hz. The abbreviations used are as follows: s, singlet; d, doublet; m, multiplet. Analytical thin layer chromatography (TLC) on silica gel plates containing a UV indicator was employed routinely to follow the course of reactions and to check the purity of the products. All reagents and solvents were purified and dried by standard techniques. The microspheres were prepared with poly (D, L-lactide co-glycolide) PLGA (50:50, mol. wt 30,000–60,000), which was purchased from Sigma-Aldrich (St. Louis, USA). The emulsifier, low molecular weight polyvinyl alcohol (PVA) was obtained from Alfa Aesar (Karlsruhe, Germany). Dichloromethane (DCM) was purchased from Avonchem (United Kingdom). Dimethyl sulfoxide (DMSO) was obtained from Loba Chemie

(Mumbai, India). All ingredients used were of analytical grade. All cell lines have been purchased from the American Type Culture Collection (ATCC).

S.4. Spectral data

IR of compound 14a

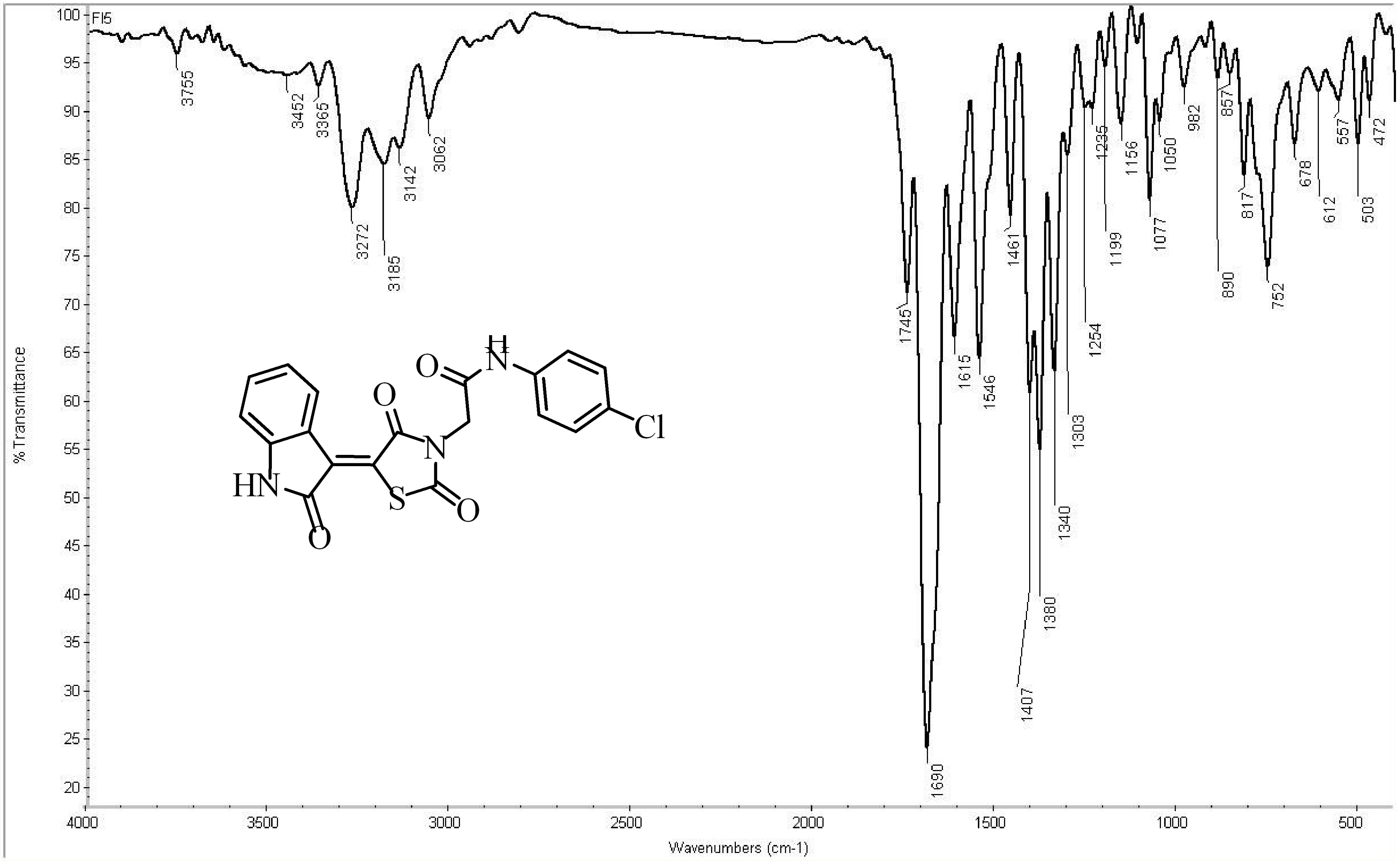

IR of compound 14b

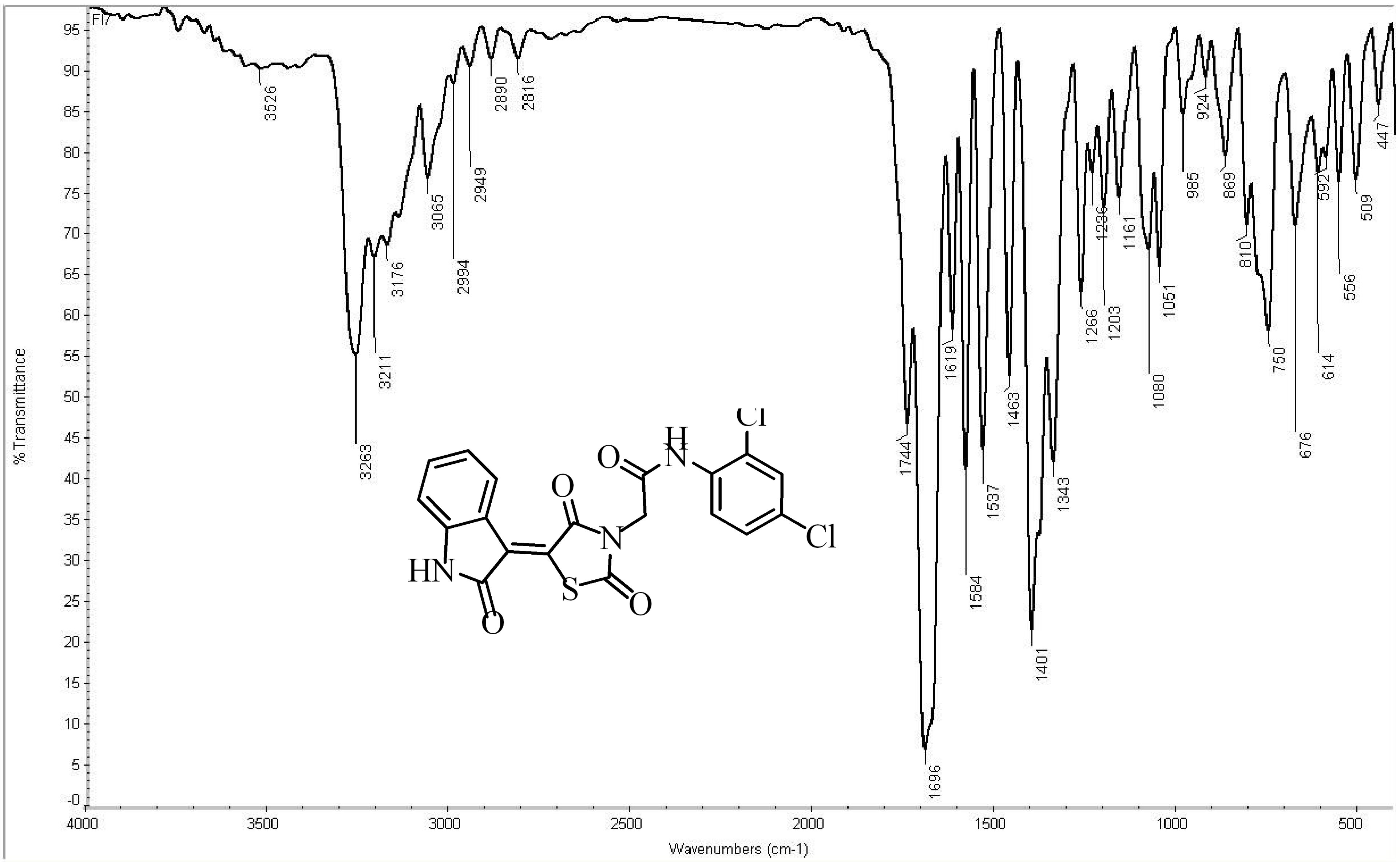

IR of compound 14c

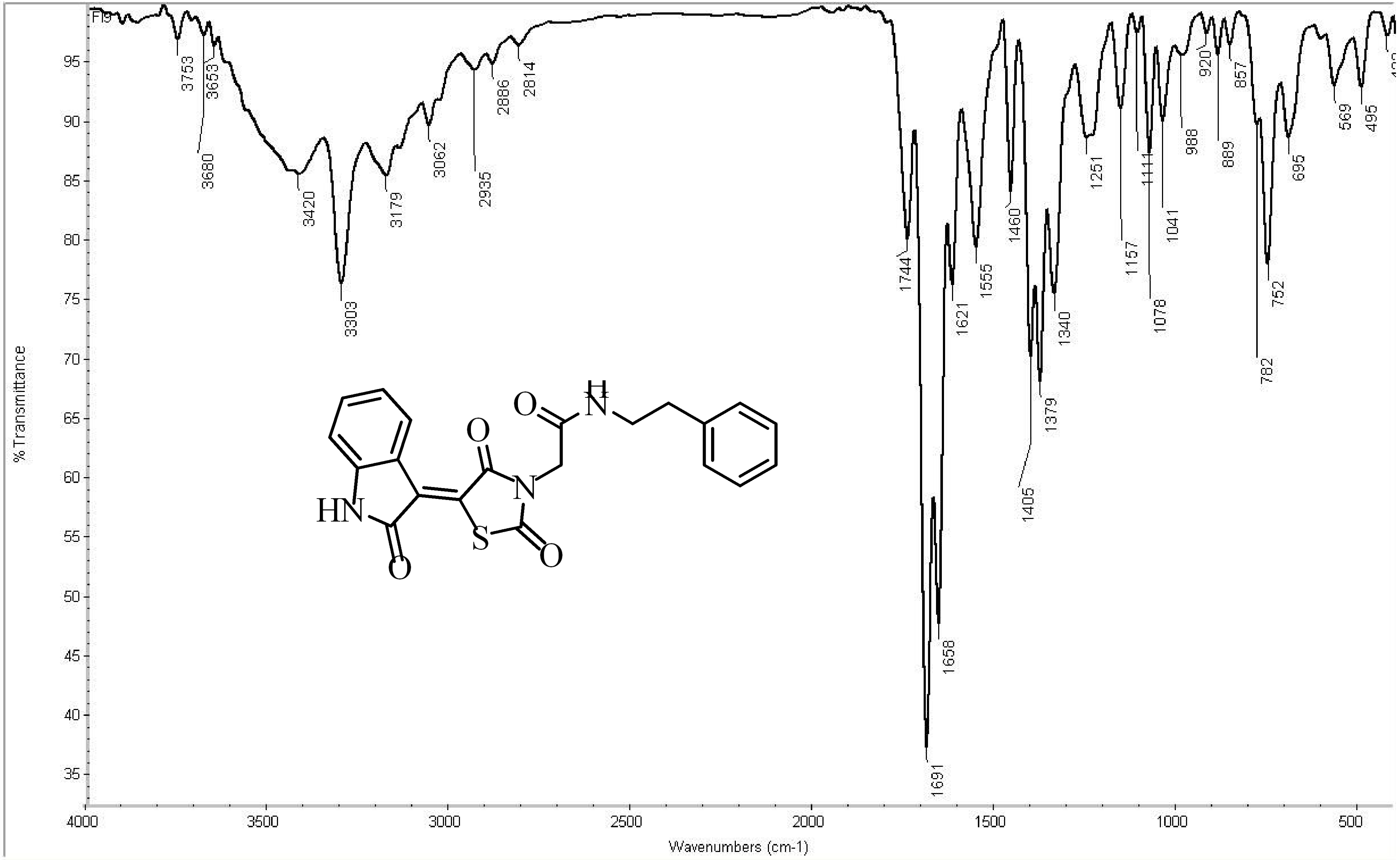

IR of compound 10a

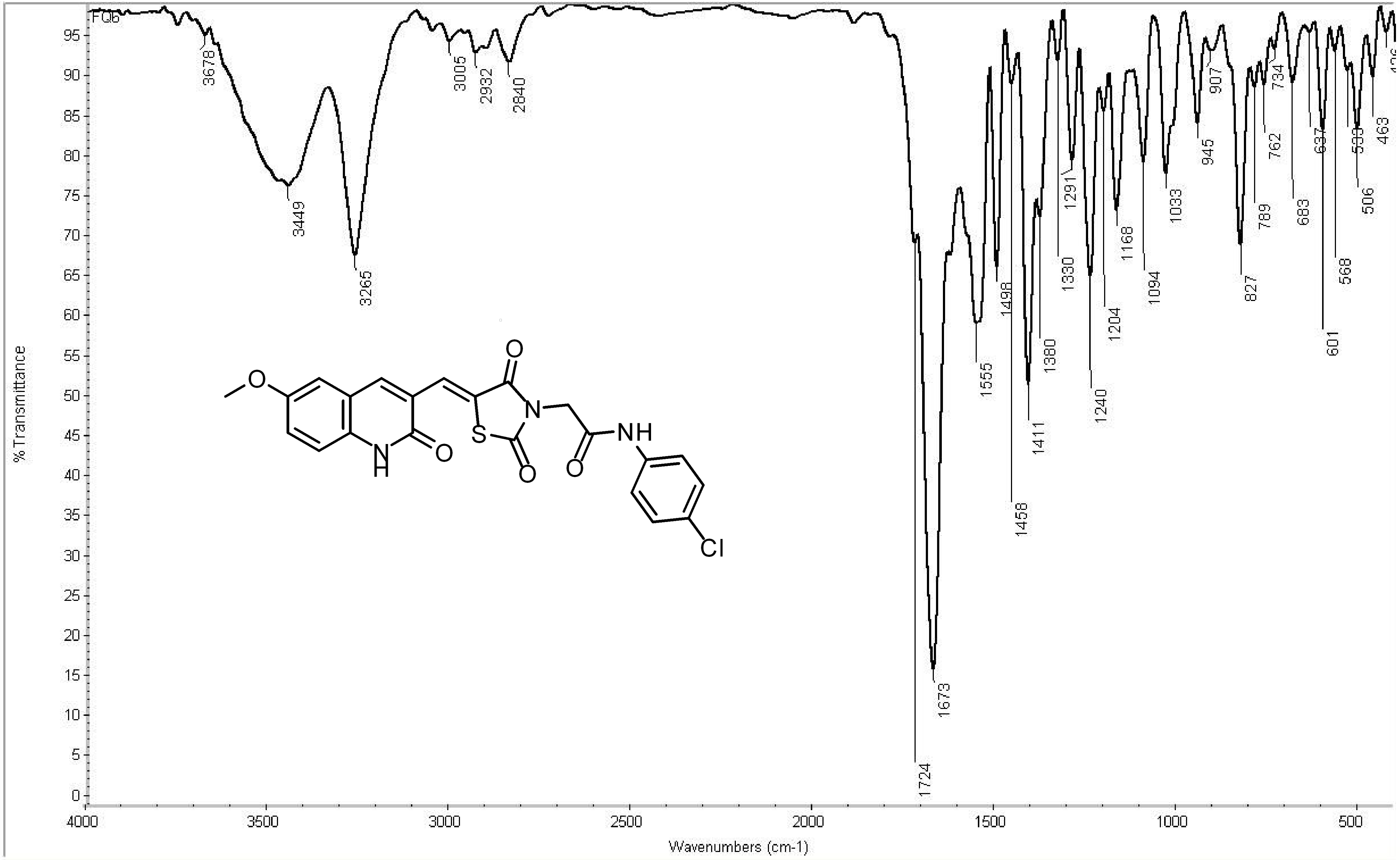

IR of compound 10b

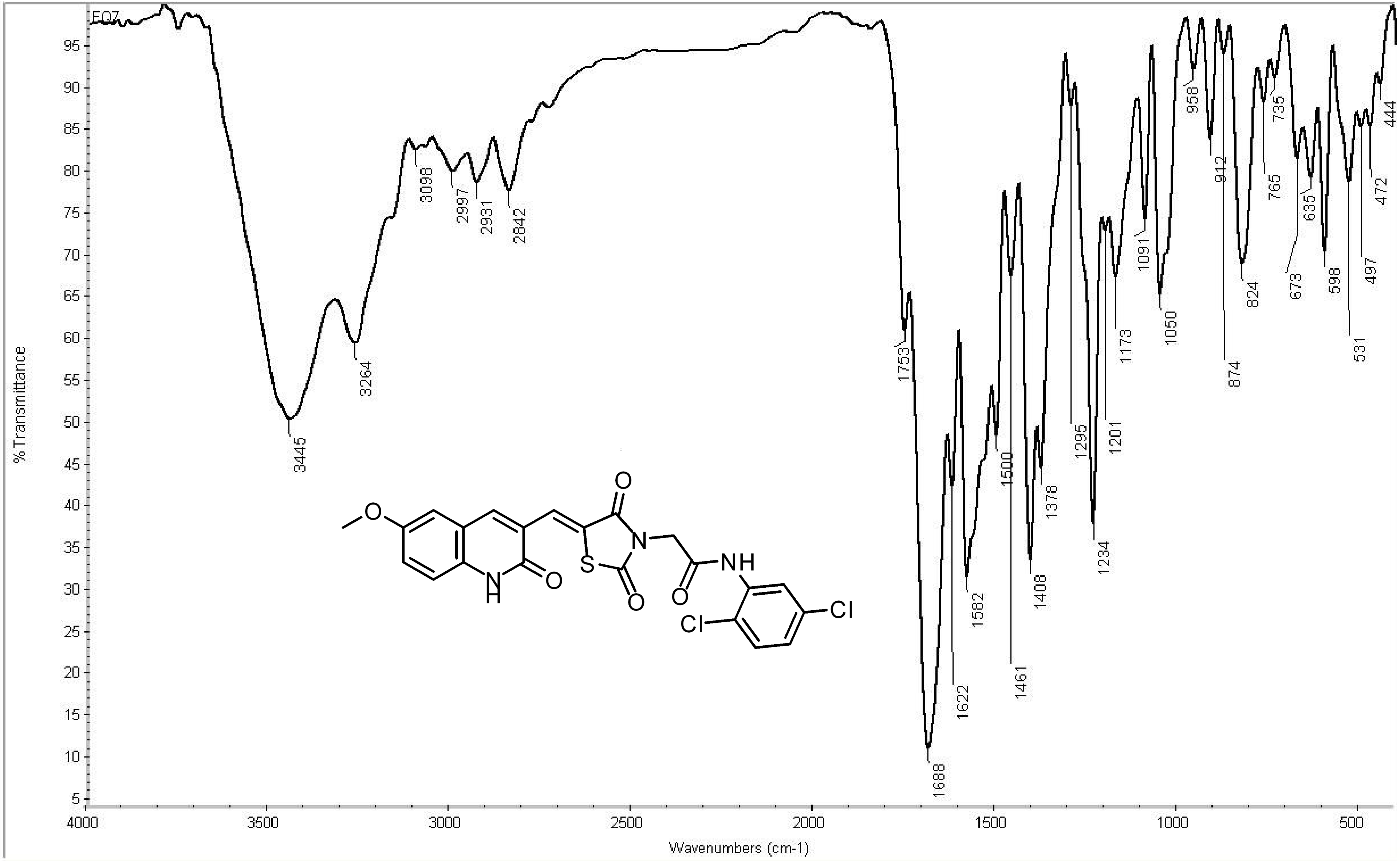

1H NMR of 14a

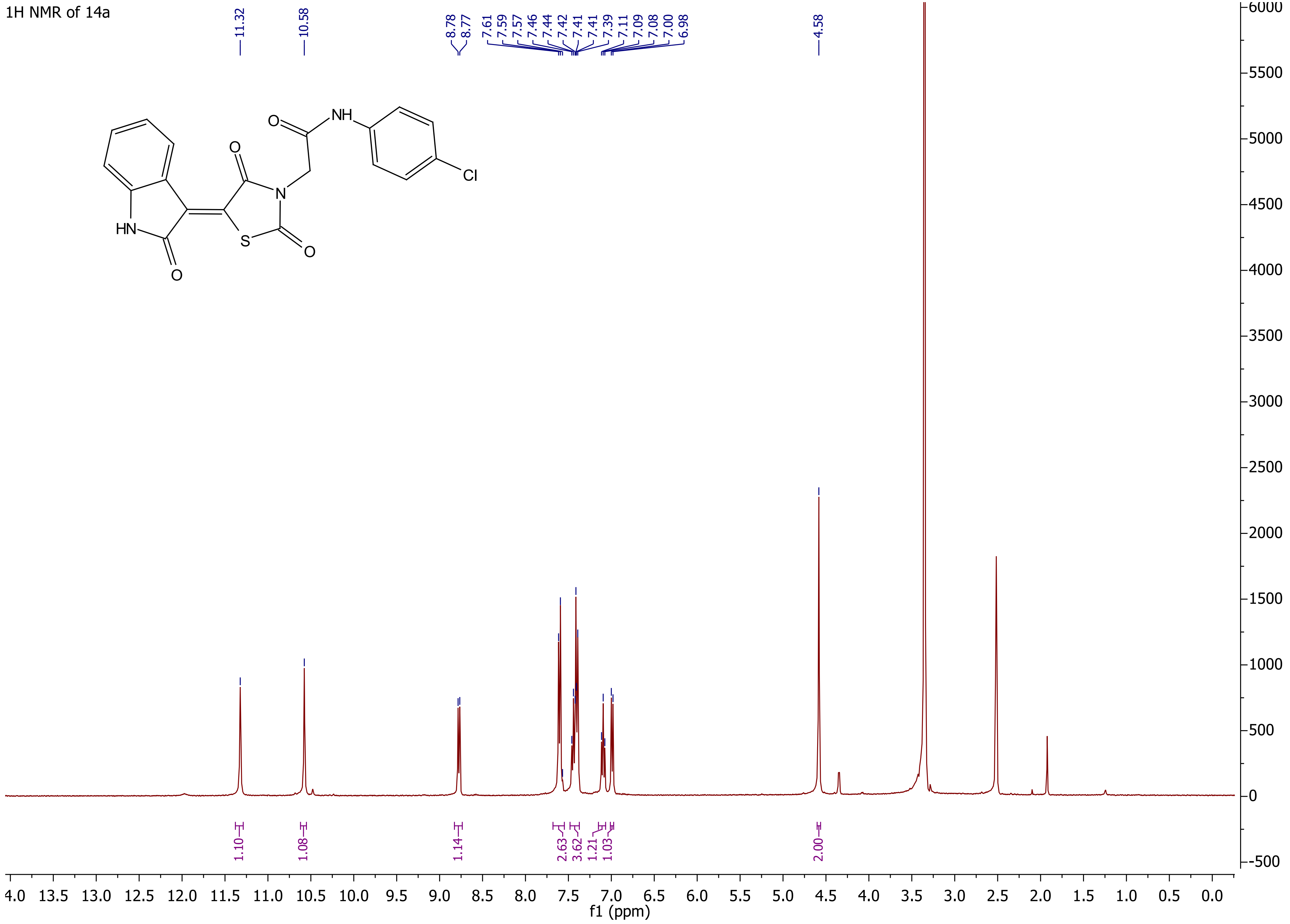
11.32
10.58
8.78
8.77
7.61
7.59
7.57
7.46
7.44
7.42
7.41
7.41
7.39
7.11
7.09
7.08
7.00
6.98
4.58
NH
O
O
N
Cl
HN
S
O
O
1.10
1.08
1.14
2.63
3.62
1.21
1.03
2.00
f1 (ppm)

1H NMR of 14a

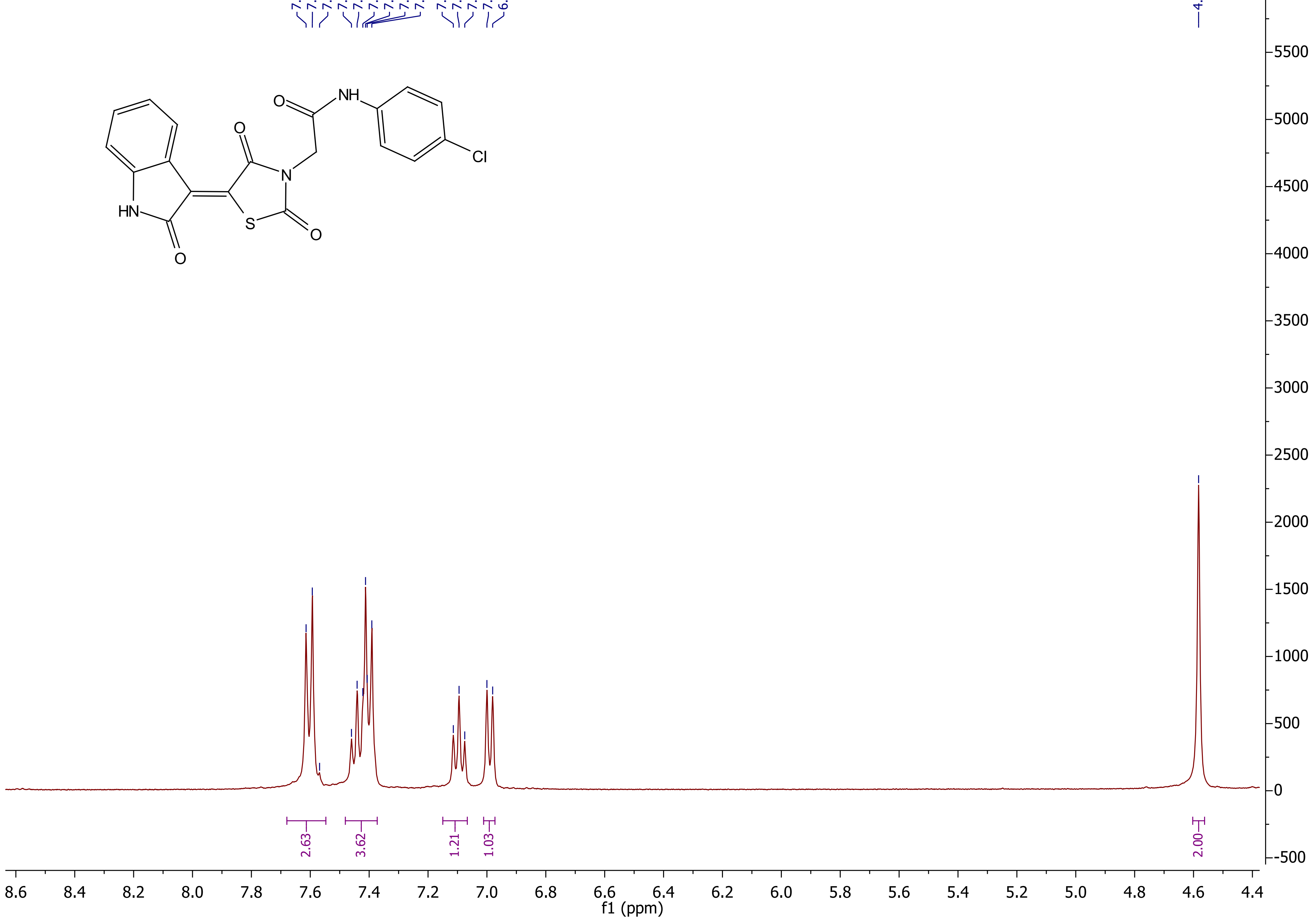

7.61
7.59
7.57
7.46
7.44
7.42
7.41
7.41
7.39
7.11
7.09
7.08
7.00
6.98
4.58
O
NH
Cl
O
N
HN
S
O
O
2.63
3.62
1.21
1.03
2.00
f1 (ppm)

1H NMR of 14a

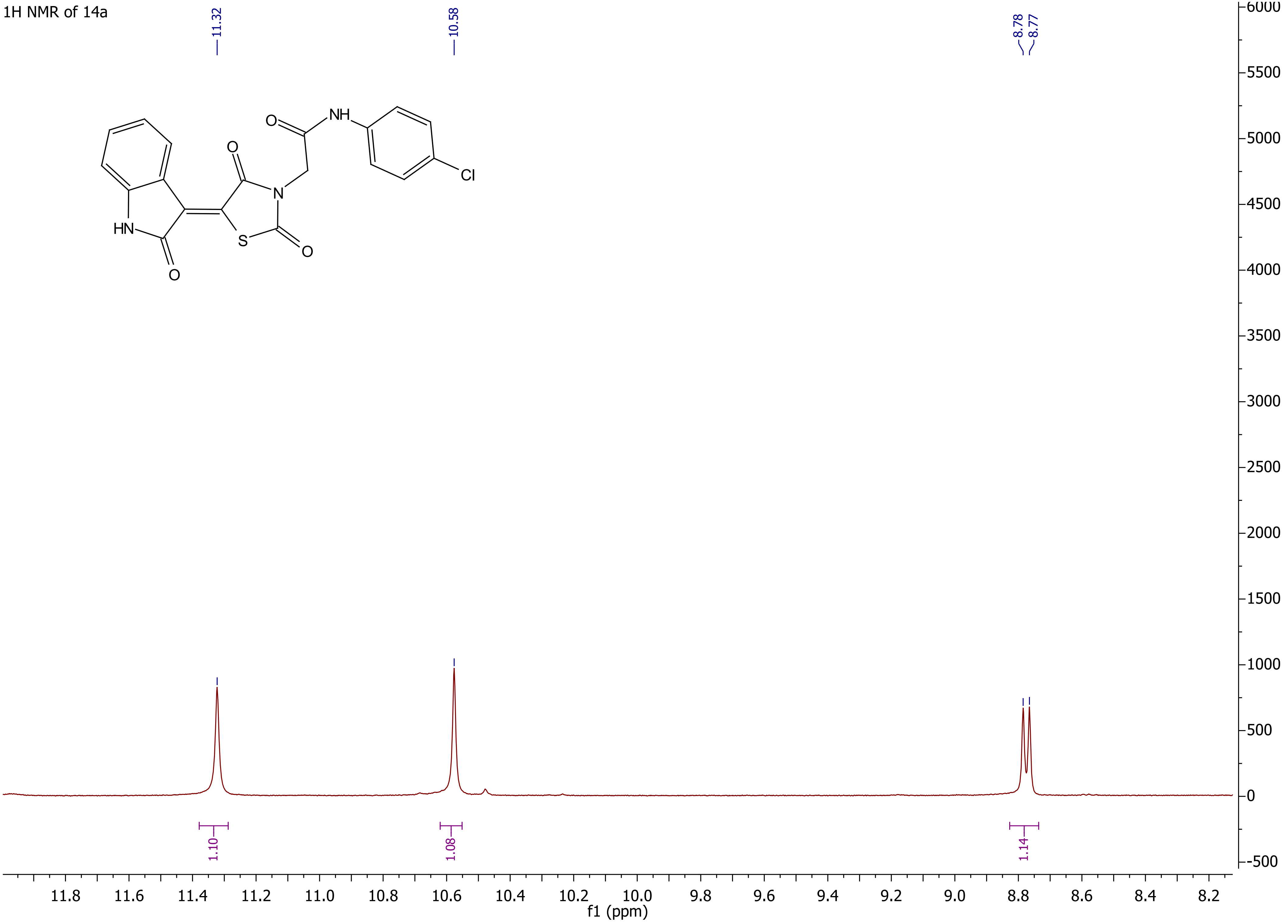
11.32
10.58
8.78
8.77
1.10
1.08
1.14
f1 (ppm)
11.8 11.6 11.4 11.2 11.0 10.8 10.6 10.4 10.2 10.0 9.8 9.6 9.4 9.2 9.0 8.8 8.6 8.4 8.2
6000 5500 5000 4500 4000 3500 3000 2500 2000 1500 1000 500 0 -500
O
NH
Cl
O
N
HN
S
O
O

1H NMR of 14b

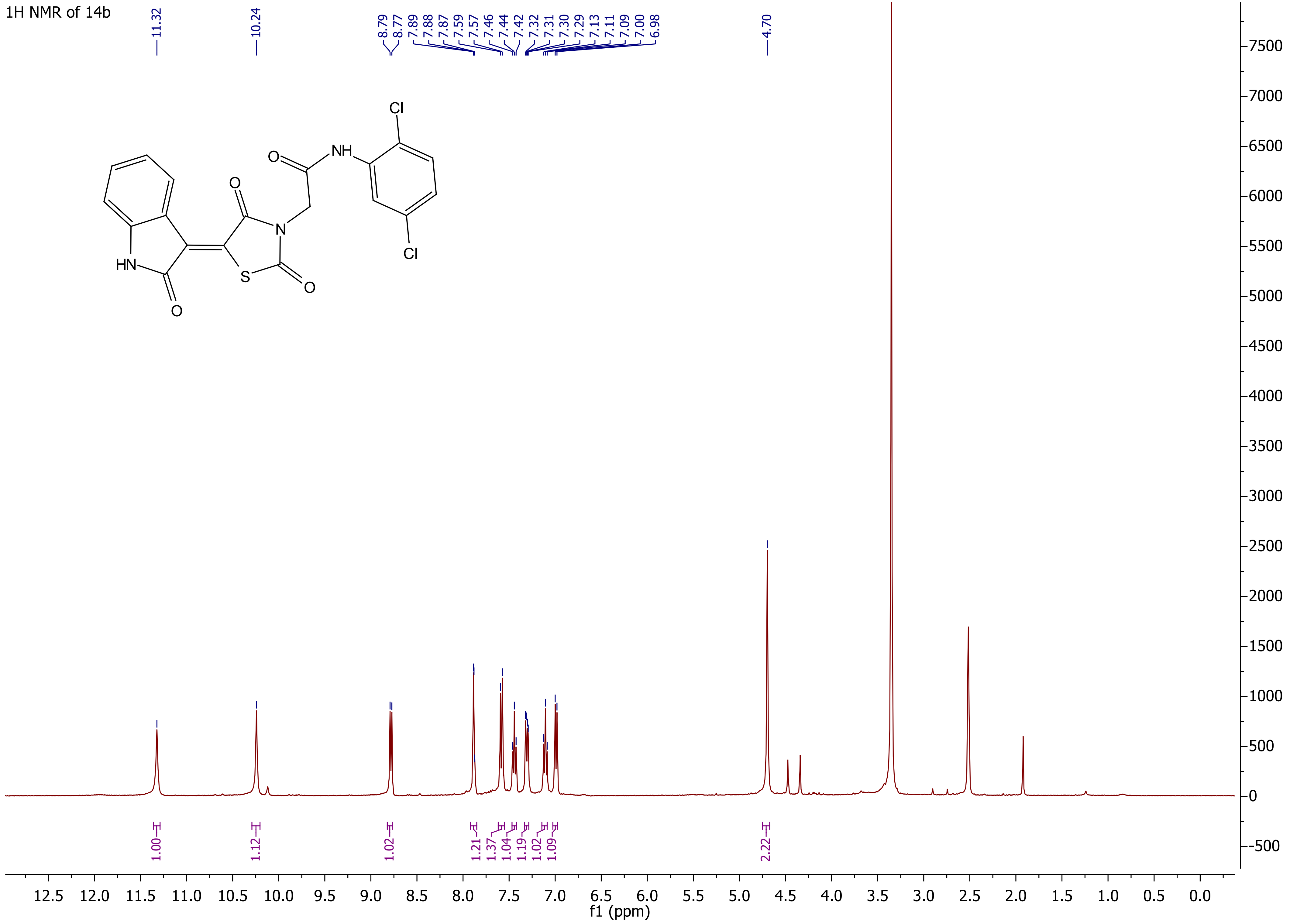
11.32
10.24
8.79
8.77
7.89
7.88
7.87
7.59
7.57
7.46
7.44
7.42
7.32
7.31
7.30
7.29
7.13
7.11
7.09
7.00
6.98
4.70
1.00
1.12
1.02
1.21
1.37
1.04
1.19
1.02
1.09
2.22
f1 (ppm)
Cl
NH
O
HN
N
S

1H NMR of 14b

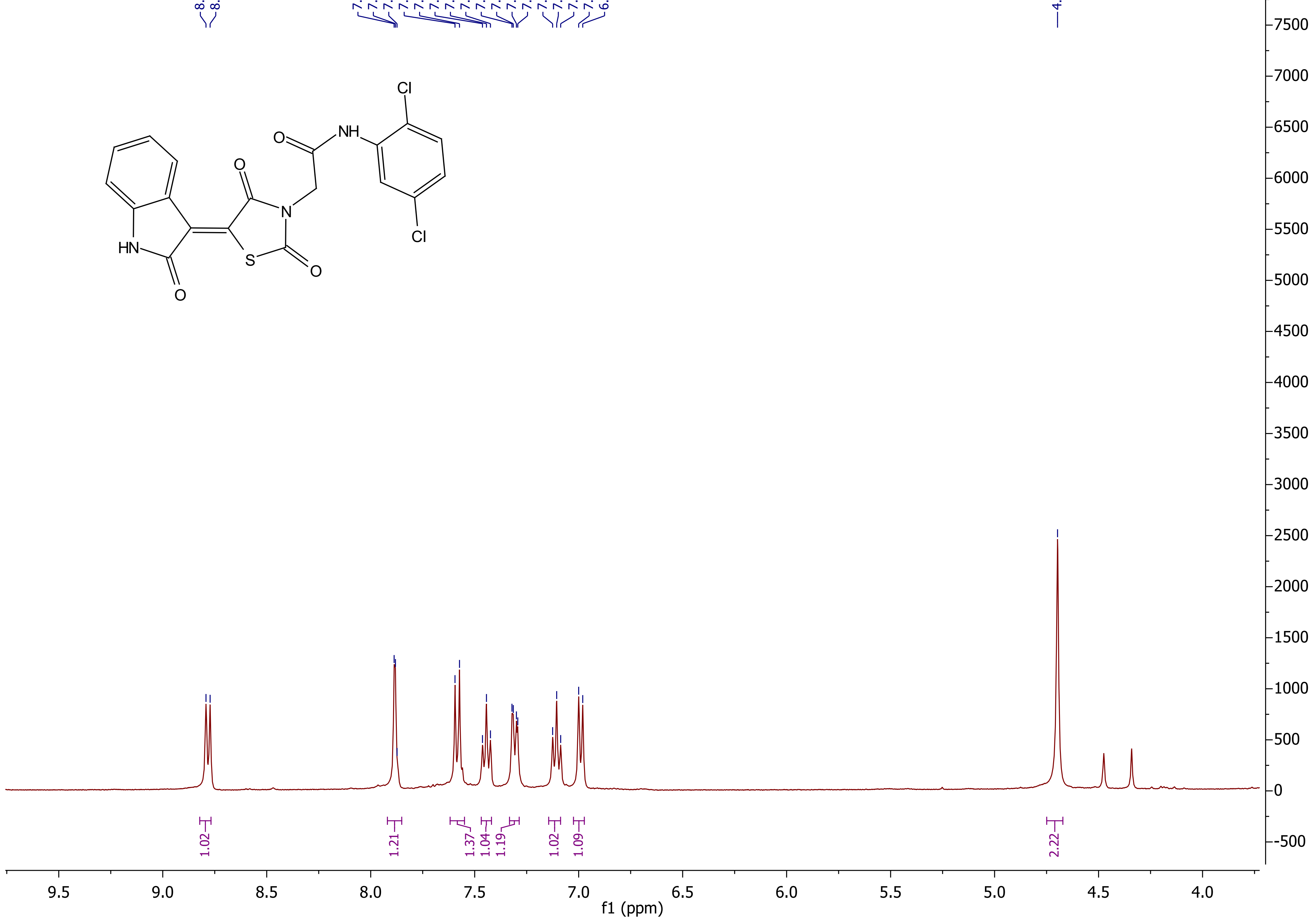
8.79
8.77
7.89
7.88
7.87
7.59
7.57
7.46
7.44
7.42
7.32
7.31
7.30
7.29
7.13
7.11
7.09
7.00
6.98
4.70
Cl
NH
O
O
N
Cl
HN
S
O
O
1.02
1.21
1.37
1.04
1.19
1.02
1.09
2.22
7500
7000
6500
6000
5500
5000
4500
4000
3500
3000
2500
2000
1500
1000
500
0
-500
9.5
9.0
8.5
8.0
7.5
7.0
6.5
6.0
5.5
5.0
4.5
4.0
f1 (ppm)

1H NMR of 14b

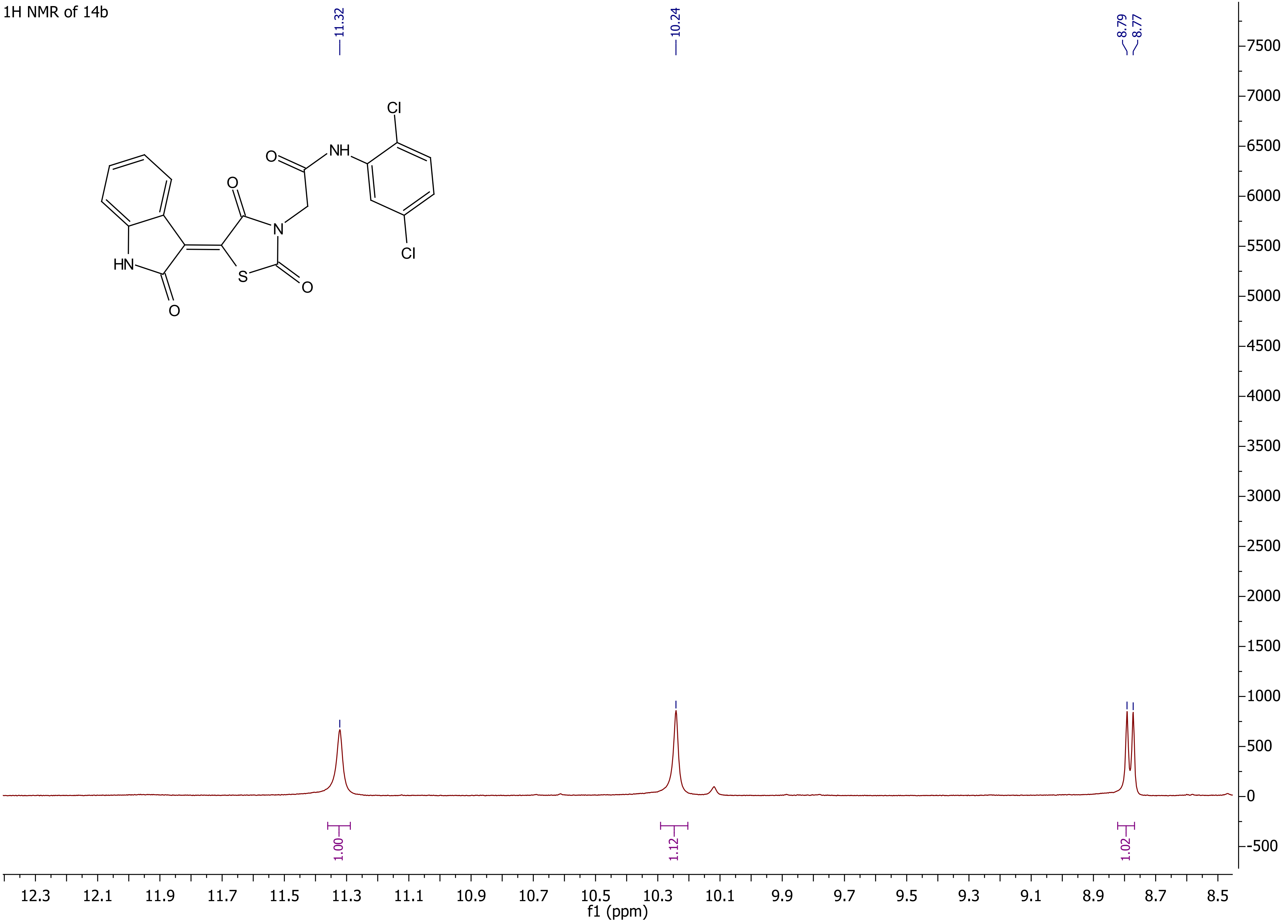

11.32
10.24
8.79
8.77
1.00
1.12
1.02
Cl
NH
O
O
N
S
HN
O
O
Cl
7500
7000
6500
6000
5500
5000
4500
4000
3500
3000
2500
2000
1500
1000
500
0
-500
12.3
12.1
11.9
11.7
11.5
11.3
11.1
10.9
10.7
10.5
10.3
10.1
9.9
9.7
9.5
9.3
9.1
8.9
8.7
8.5
f1 (ppm)

1H NMR of 14c

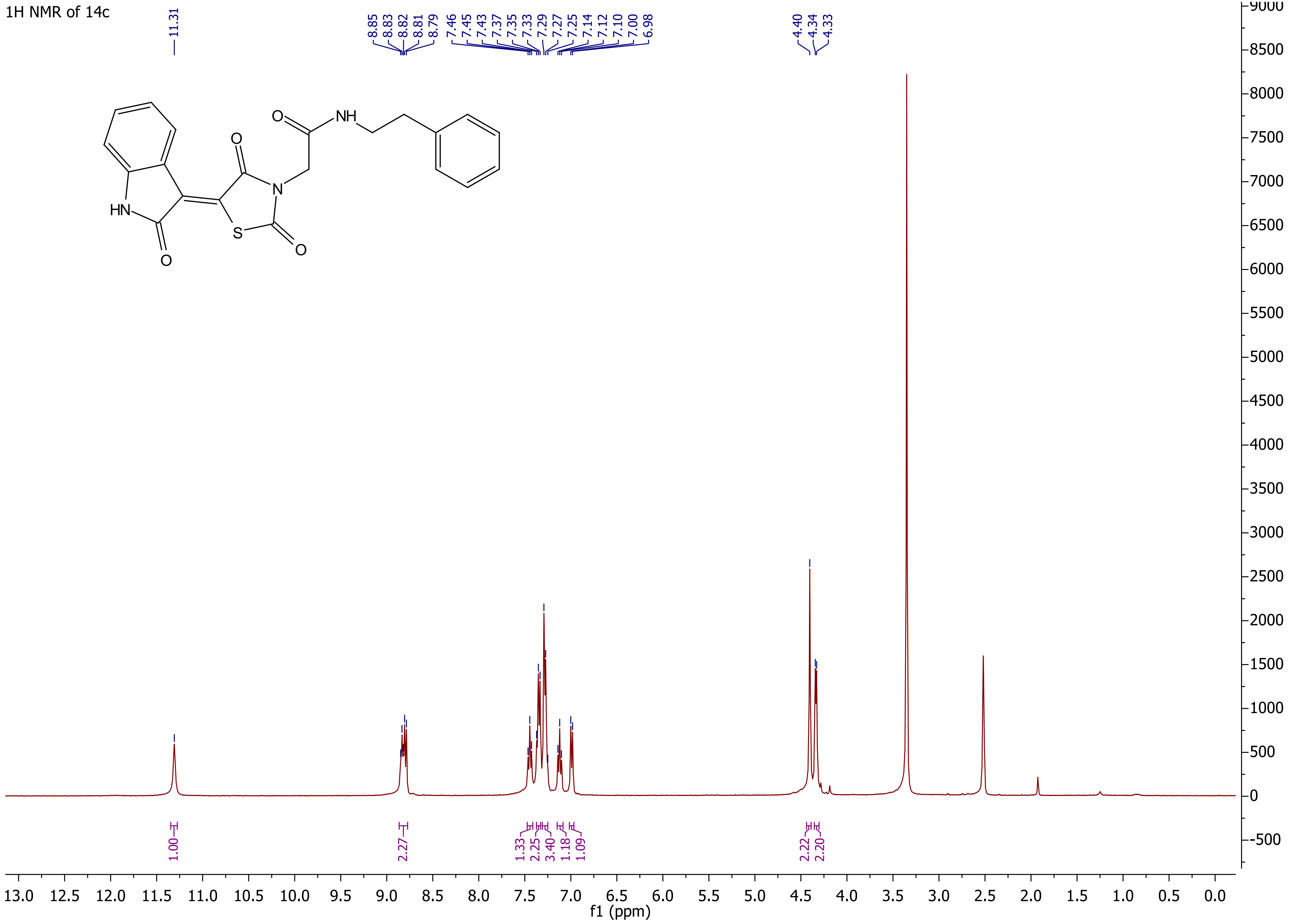

11.31
8.85
8.83
8.82
8.81
8.79
7.46
7.45
7.43
7.37
7.35
7.33
7.29
7.27
7.25
7.14
7.12
7.10
7.00
6.98
4.40
4.34
4.33
O
NH
O
N
HN
S
O
O
1.00
2.27
1.33
2.25
3.40
1.18
1.09
2.22
2.20
f1 (ppm)

1H NMR of 14c

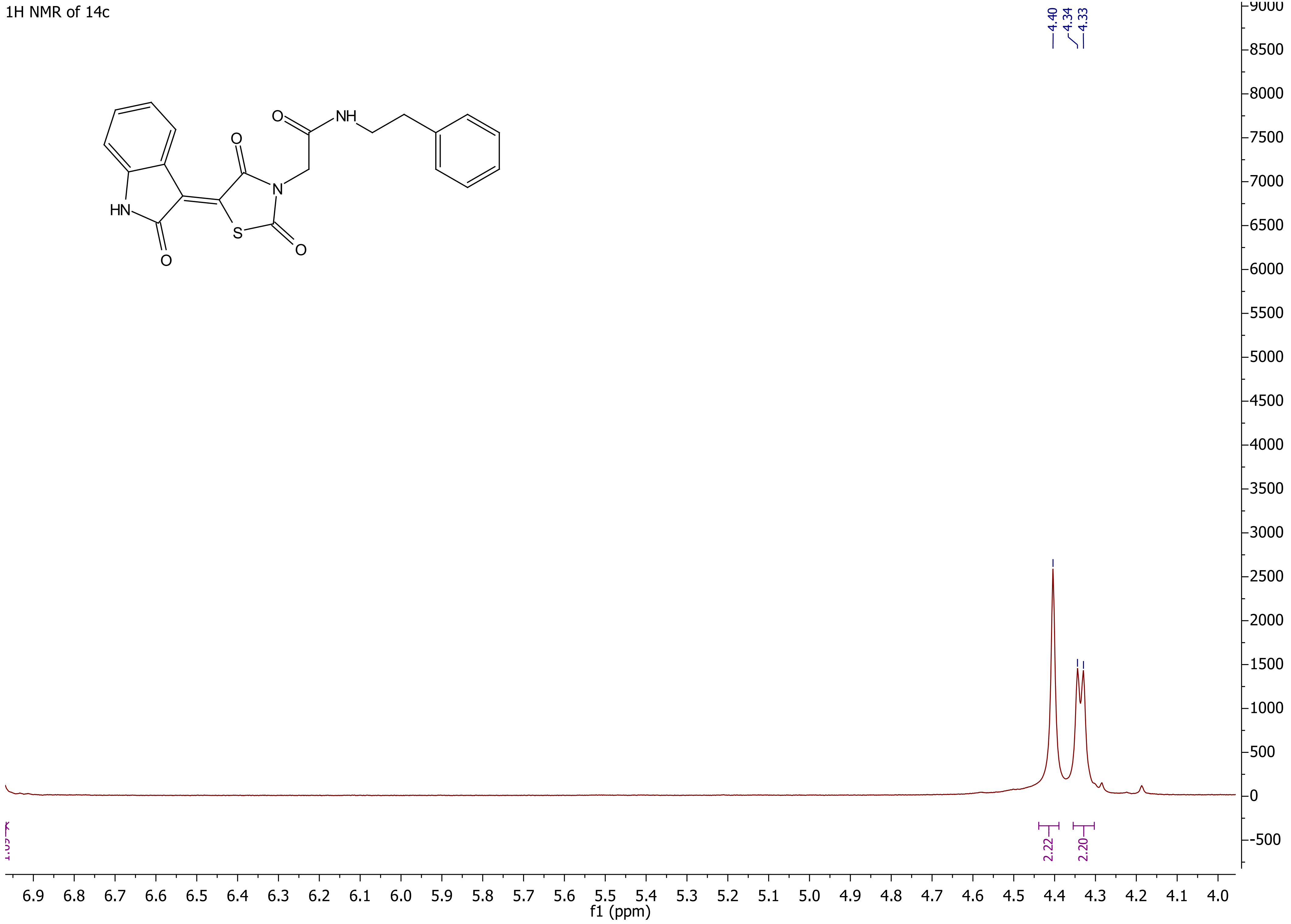

1H NMR of 14c

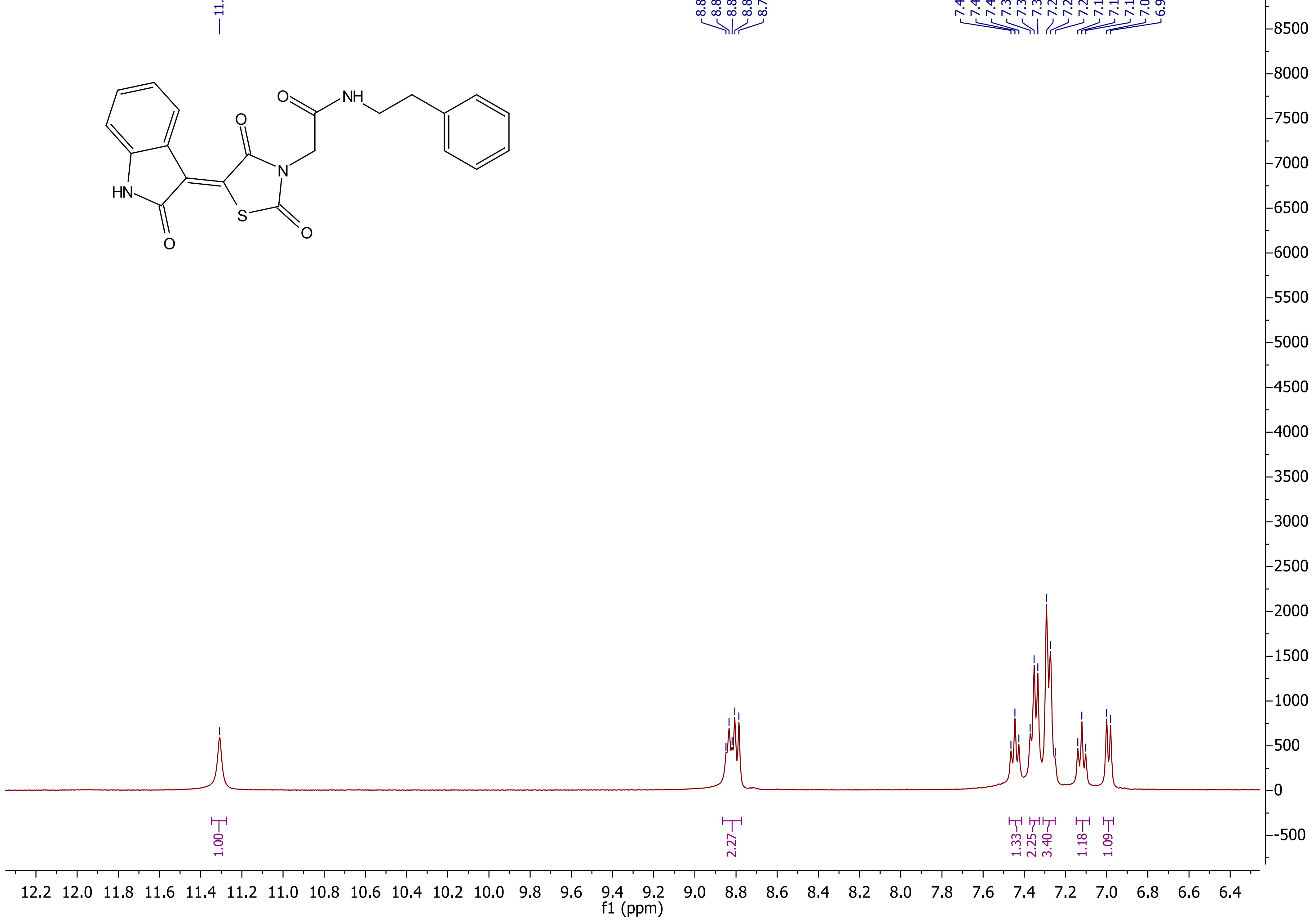

11.31
8.85
8.83
8.82
8.81
8.79
7.46
7.45
7.43
7.37
7.35
7.33
7.29
7.27
7.25
7.14
7.12
7.10
7.00
6.98
O
NH
O
N
HN
S
O
O
1.00
2.27
1.33
2.25
3.40
1.18
1.09
12.2 12.0 11.8 11.6 11.4 11.2 11.0 10.8 10.6 10.4 10.2 10.0 9.8 9.6 9.4 9.2 9.0 8.8 8.6 8.4 8.2 8.0 7.8 7.6 7.4 7.2 7.0 6.8 6.6 6.4
f1 (ppm)
9000
8500
8000
7500
7000
6500
6000
5500
5000
4500
4000
3500
3000
2500
2000
1500
1000
500
0
-500

13C NMR of 14a

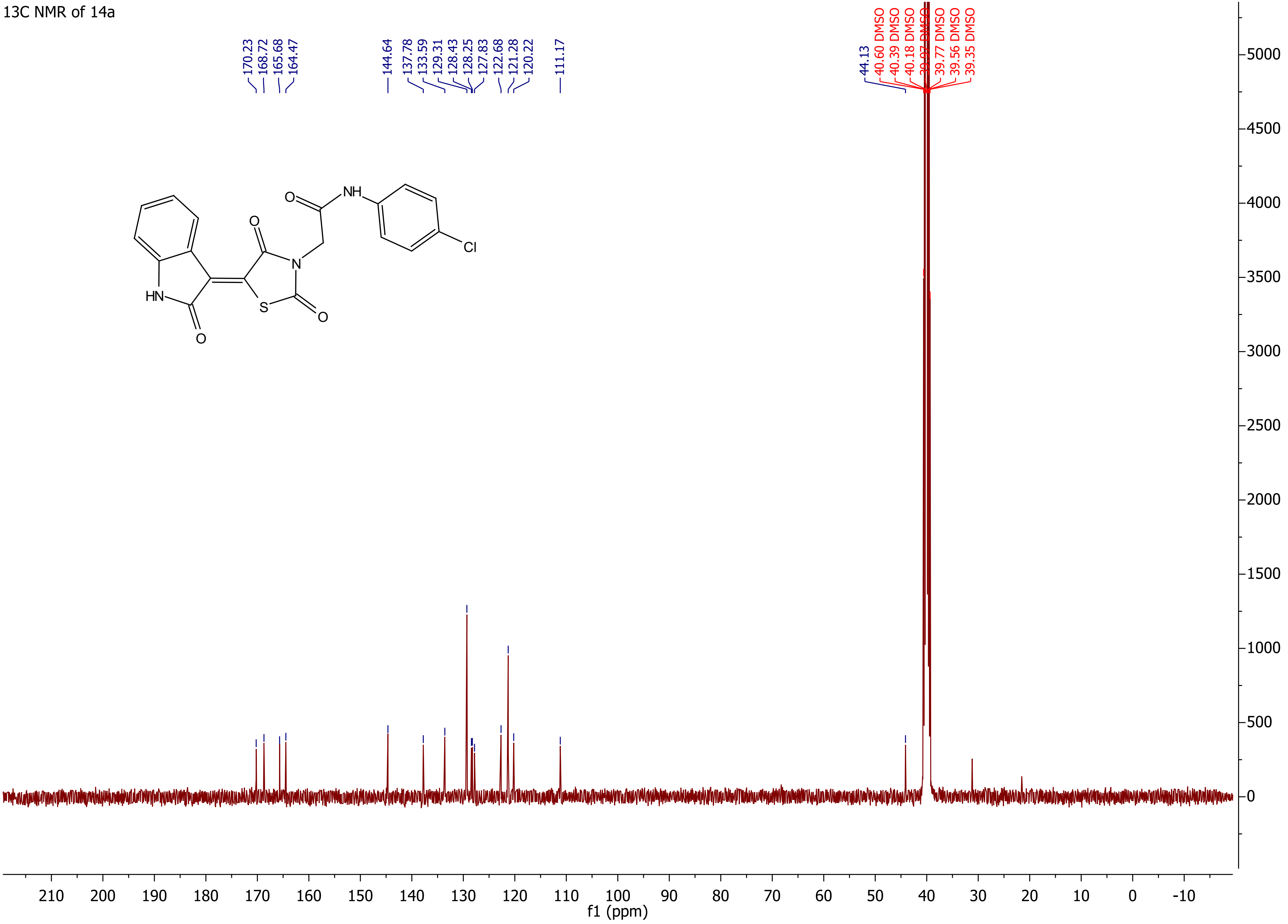

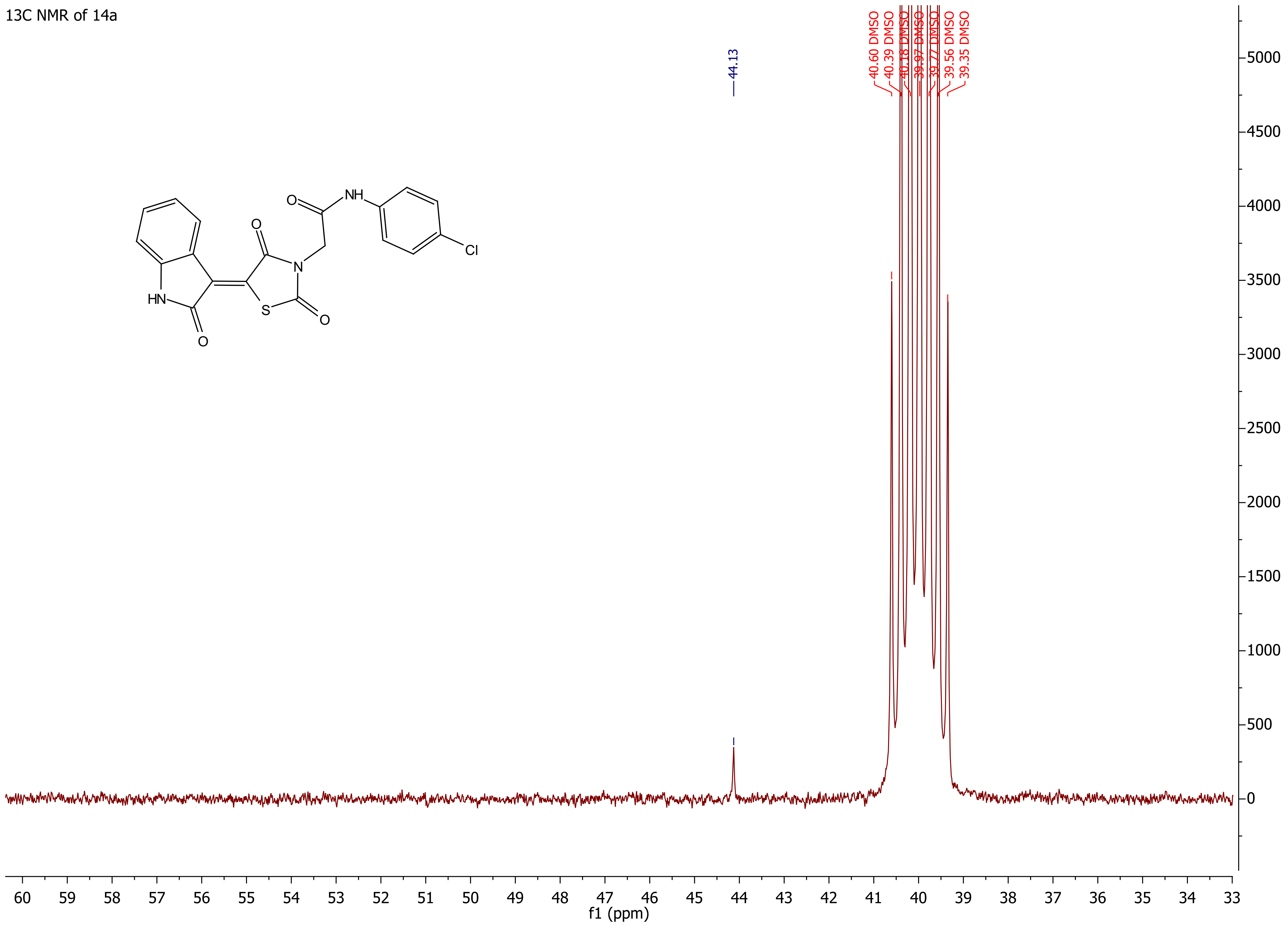
13C NMR of 14a
44.13
40.60 DMSO
40.39 DMSO
40.18 DMSO
39.97 DMSO
39.77 DMSO
39.56 DMSO
39.35 DMSO
f1 (ppm)

13C NMR of 14a

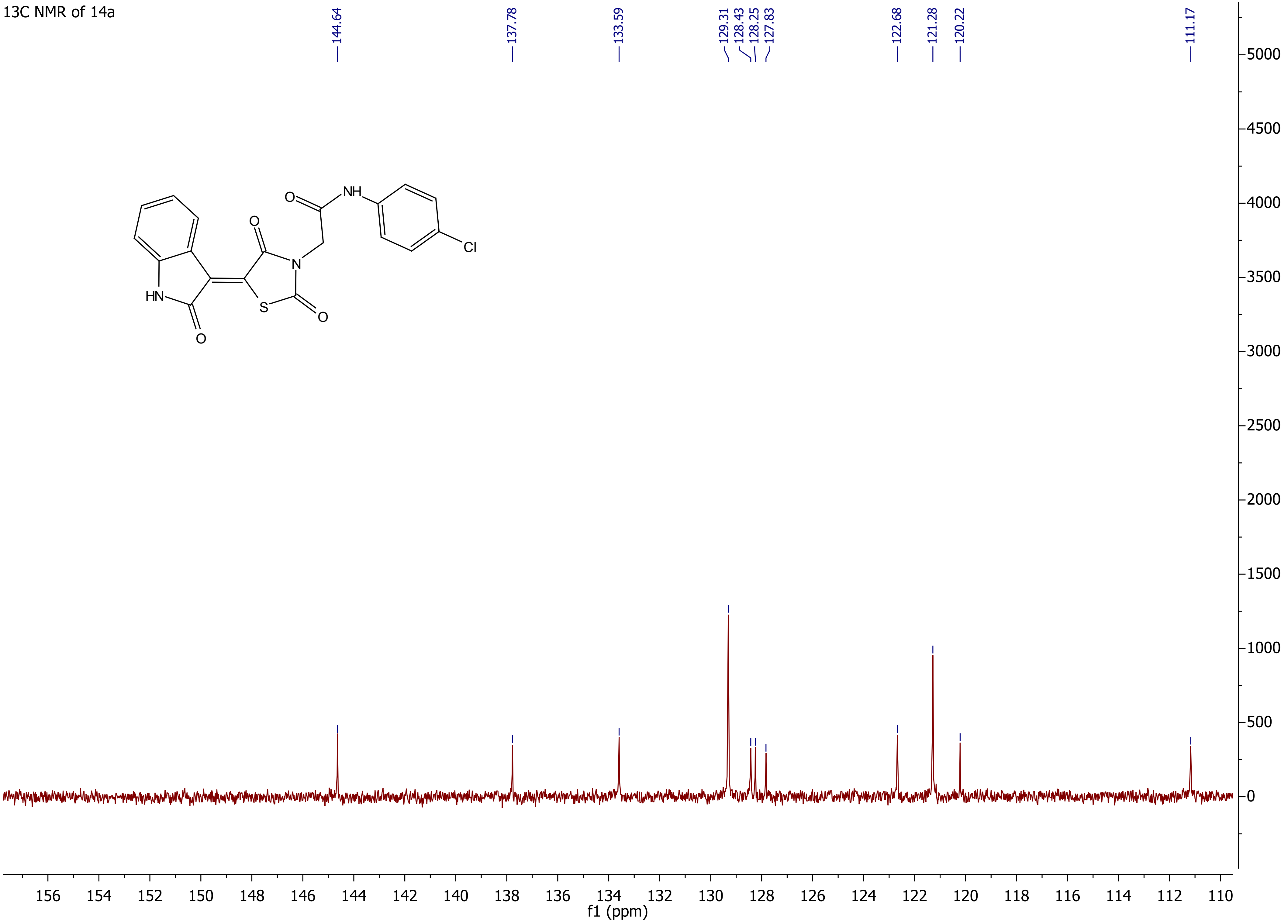

13C NMR of 14a

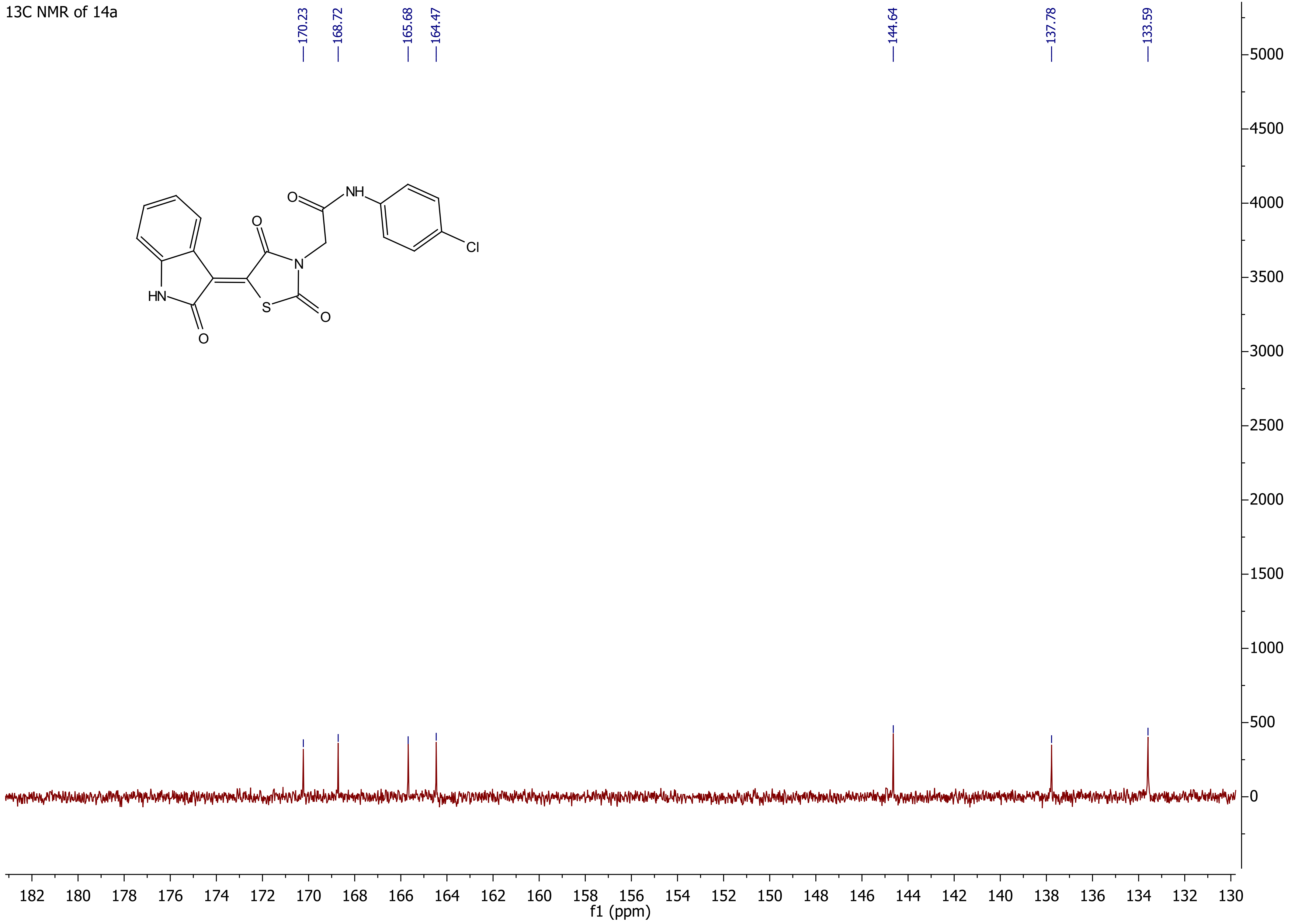

13C NMR of 14c

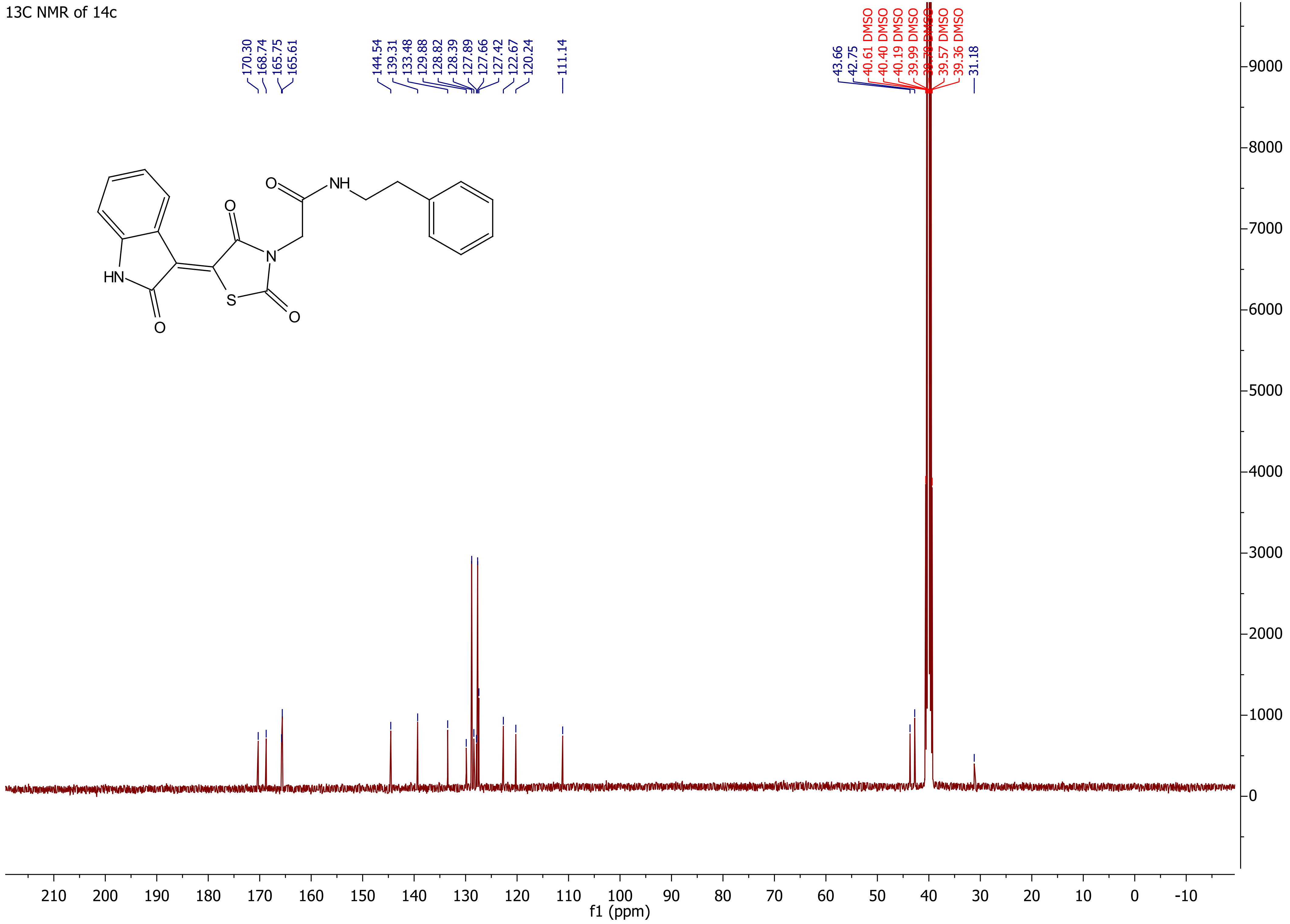

13C NMR of 14c

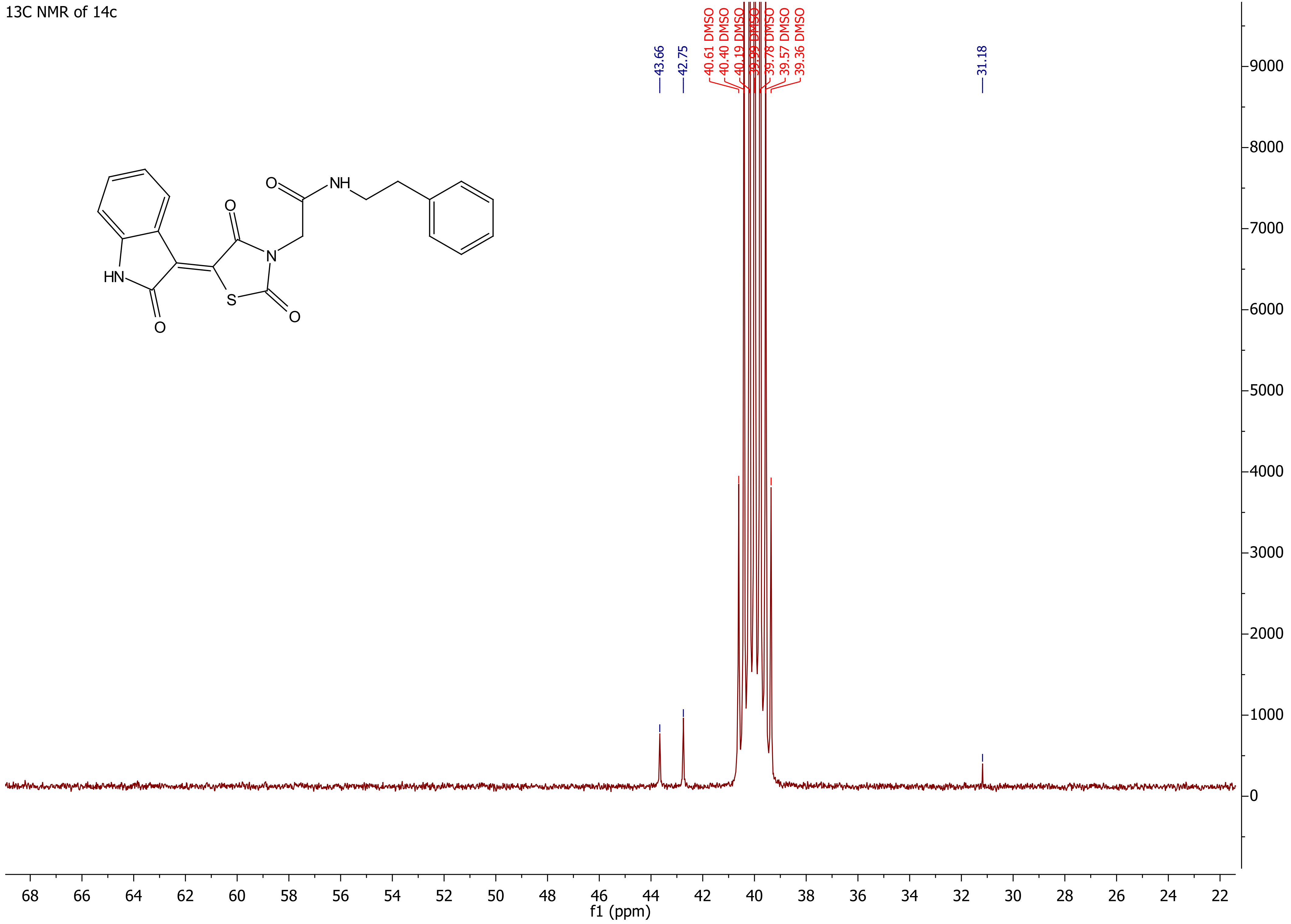

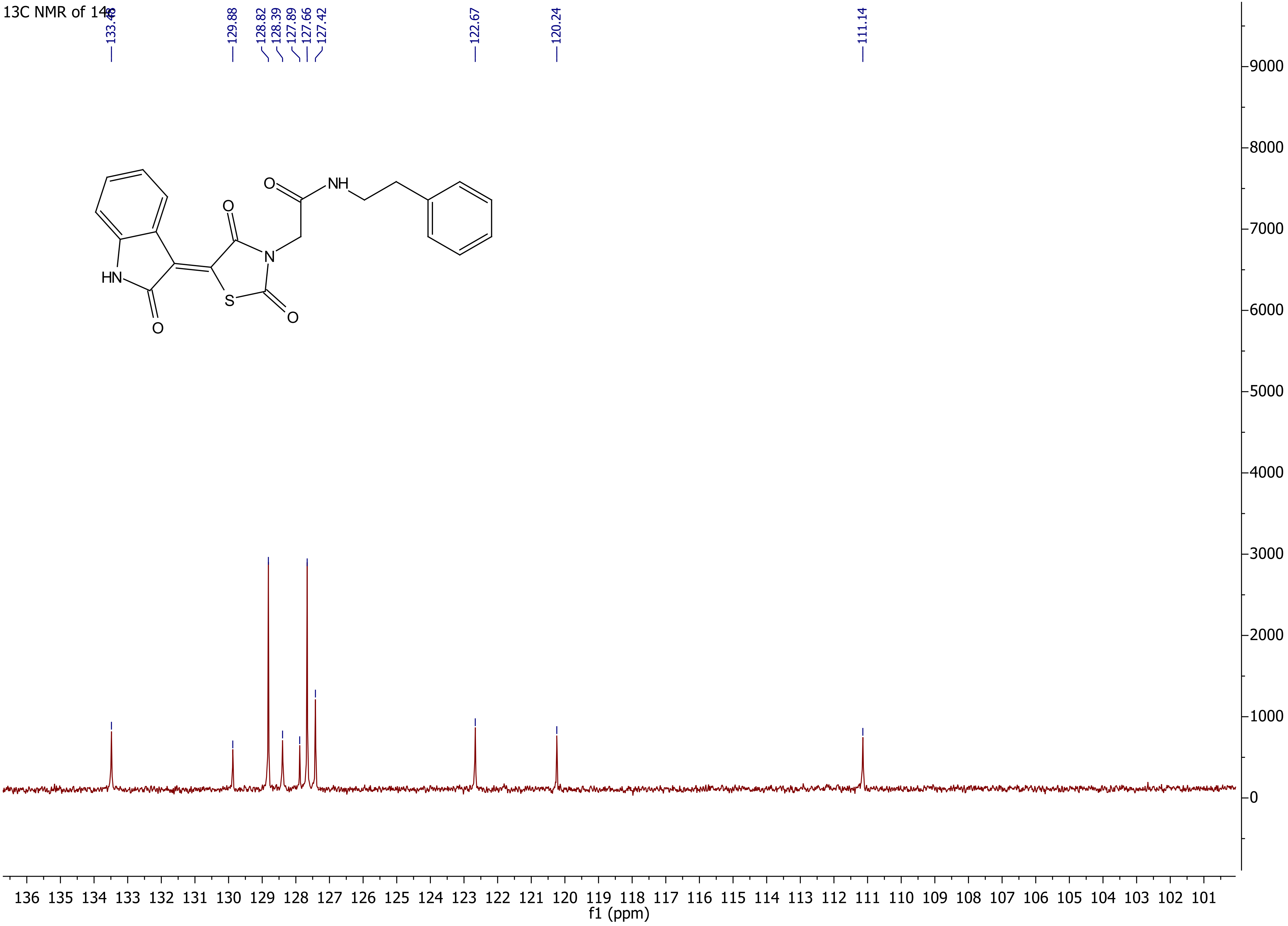

13C NMR of 14[illegible]
133.48
129.88
128.82
128.39
127.89
127.66
127.42
122.67
120.24
111.14
O
NH
O
O
N
HN
S
O
O
9000
8000
7000
6000
5000
4000
3000
2000
1000
0
136 135 134 133 132 131 130 129 128 127 126 125 124 123 122 121 120 119 118 117 116 115 114 113 112 111 110 109 108 107 106 105 104 103 102 101
f1 (ppm)

13C NMR of 14c

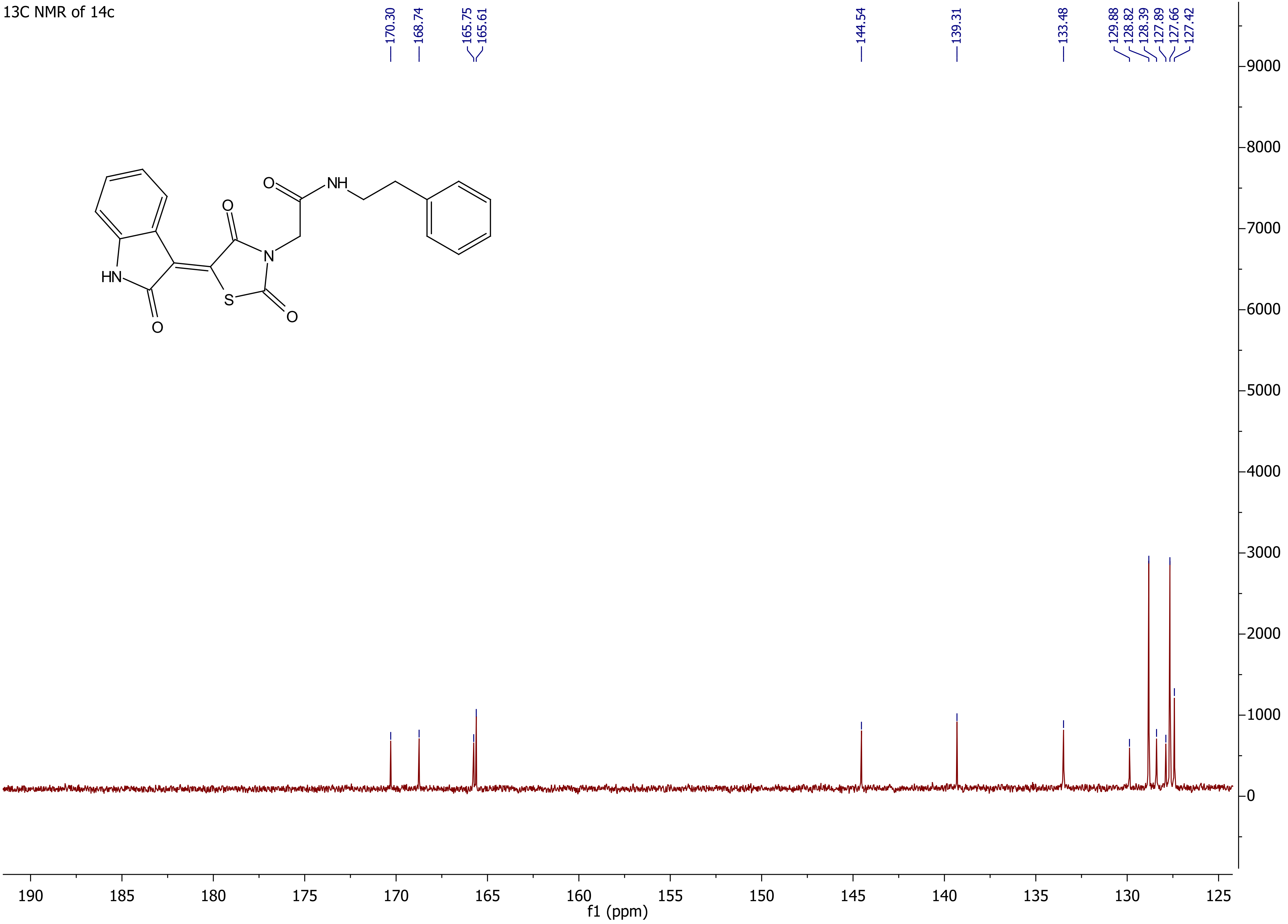

S.5. Raw data for biological testing

RAW DATA OF CYTOTOXICITY

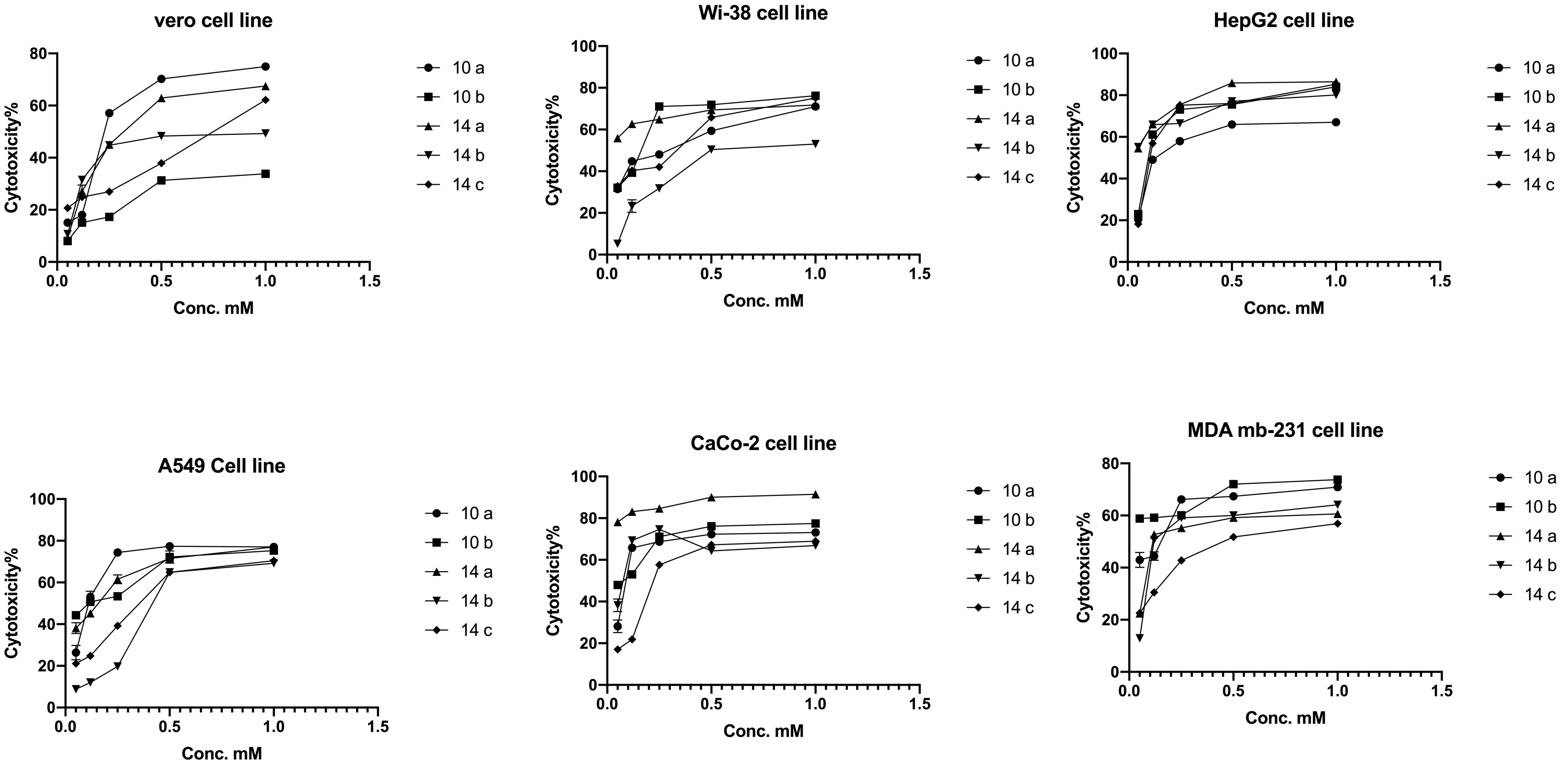
vero cell line
Cytotoxicity%
Conc. mM
10 a
10 b
14 a
14 b
14 c
Wi-38 cell line
Cytotoxicity%
Conc. mM
10 a
10 b
14 a
14 b
14 c
HepG2 cell line
Cytotoxicity%
Conc. mM
10 a
10 b
14 a
14 b
14 c
A549 Cell line
Cytotoxicity%
Conc. mM
10 a
10 b
14 a
14 b
14 c
CaCo-2 cell line
Cytotoxicity%
Conc. mM
10 a
10 b
14 a
14 b
14 c
MDA mb-231 cell line
Cytotoxicity%
Conc. mM
10 a
10 b
14 a
14 b
14 c

RAW DATA OF VEGFR-2 ASSAY

VEGFR-2 assay of compound 14a

Best-fit values	
LogIC50	1.961
HillSlope	-0.4675
IC50	91.51

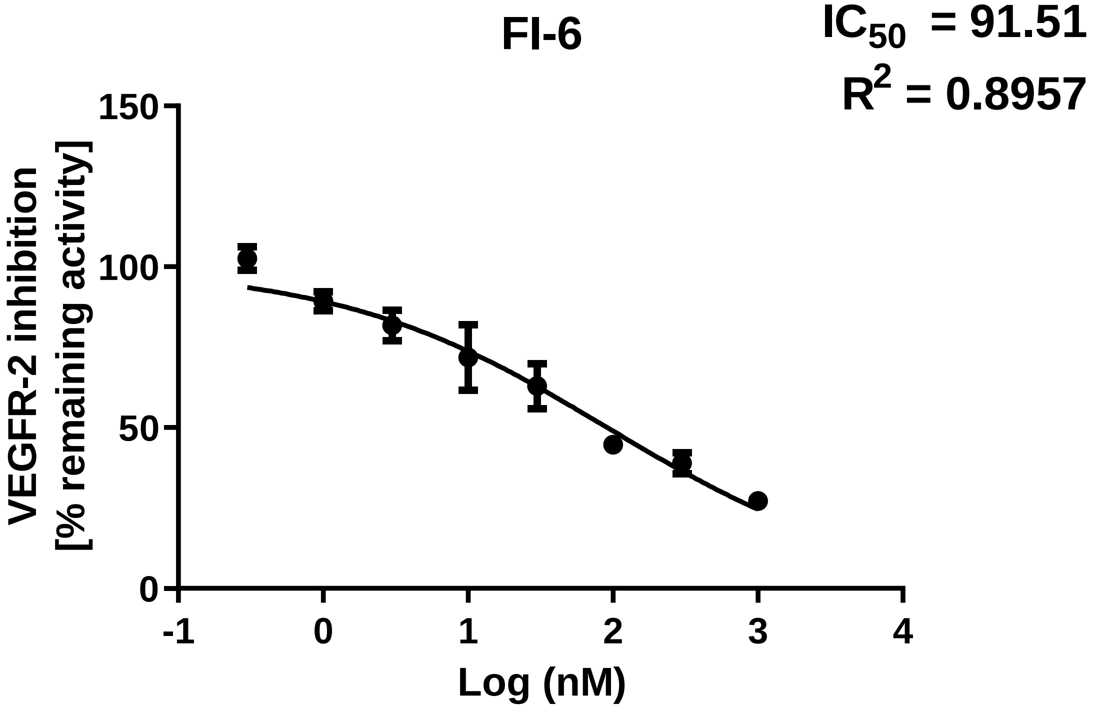

VEGFR-2 assay of compound 14b

Best-fit values	
LogIC50	1.934
HillSlope	-0.5620
IC50	85.85

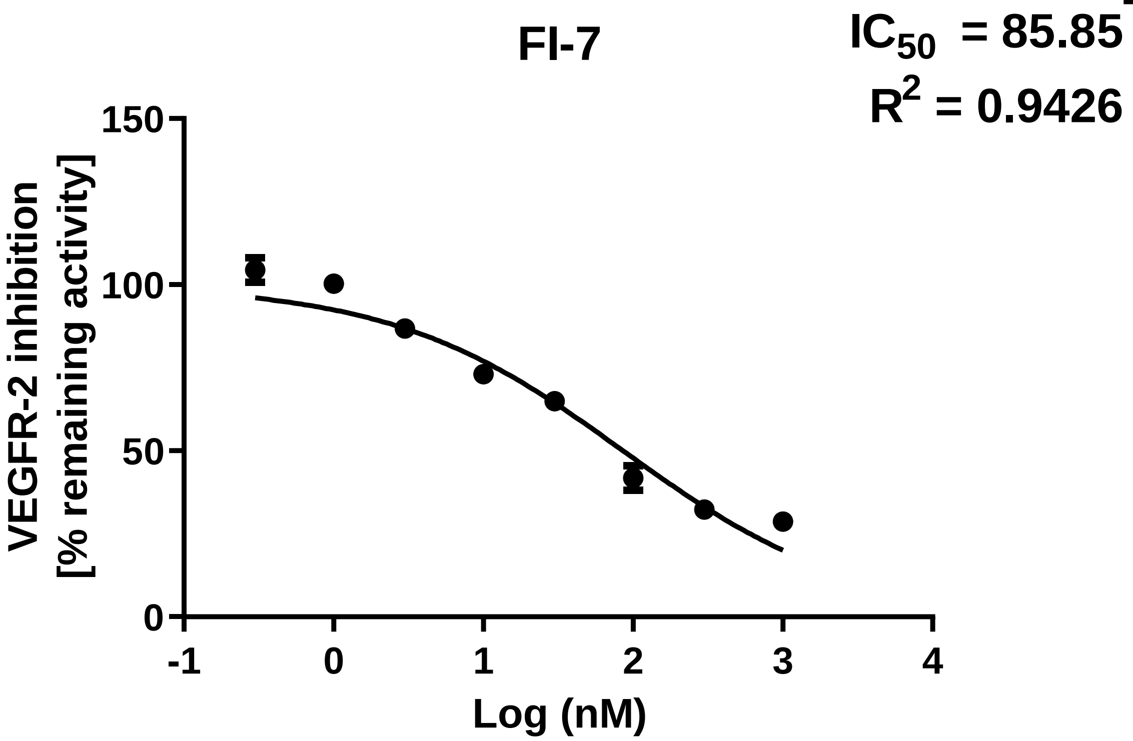

VEGFR-2 assay of compound 14c

Best-fit values	
LogIC50	1.911
HillSlope	-0.4220
IC50	81.46

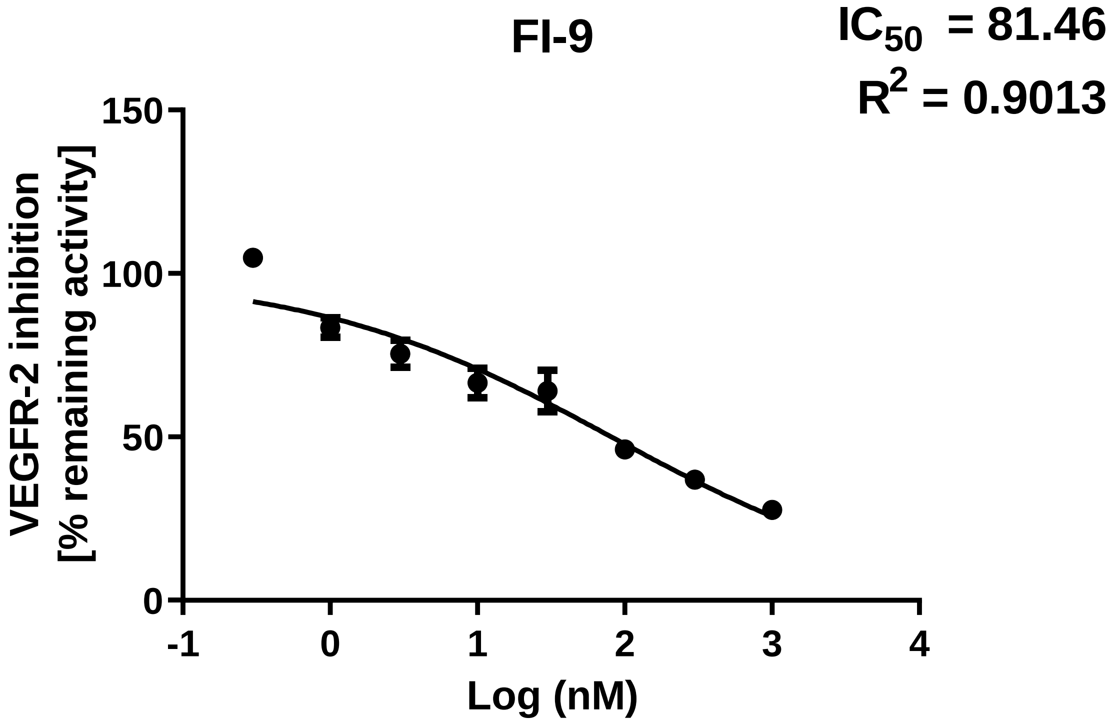

VEGFR-2 assay of compound 10a

Best-fit values	
LogIC50	1.814
HillSlope	-0.4587
IC50	65.16

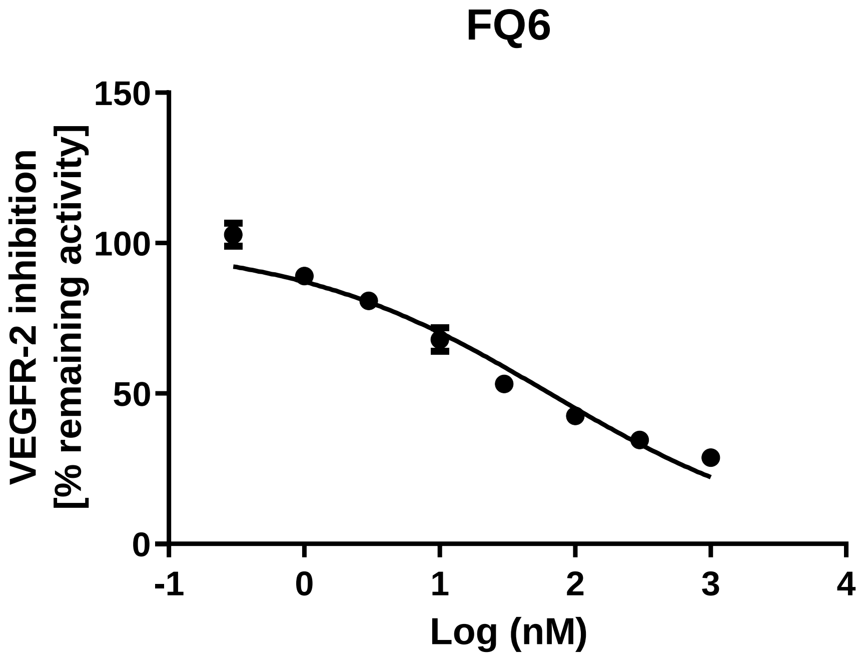

VEGFR-2 assay of compound 10b

Best-fit values	
LogIC50	2.216
HillSlope	-0.3514
IC50	164.5

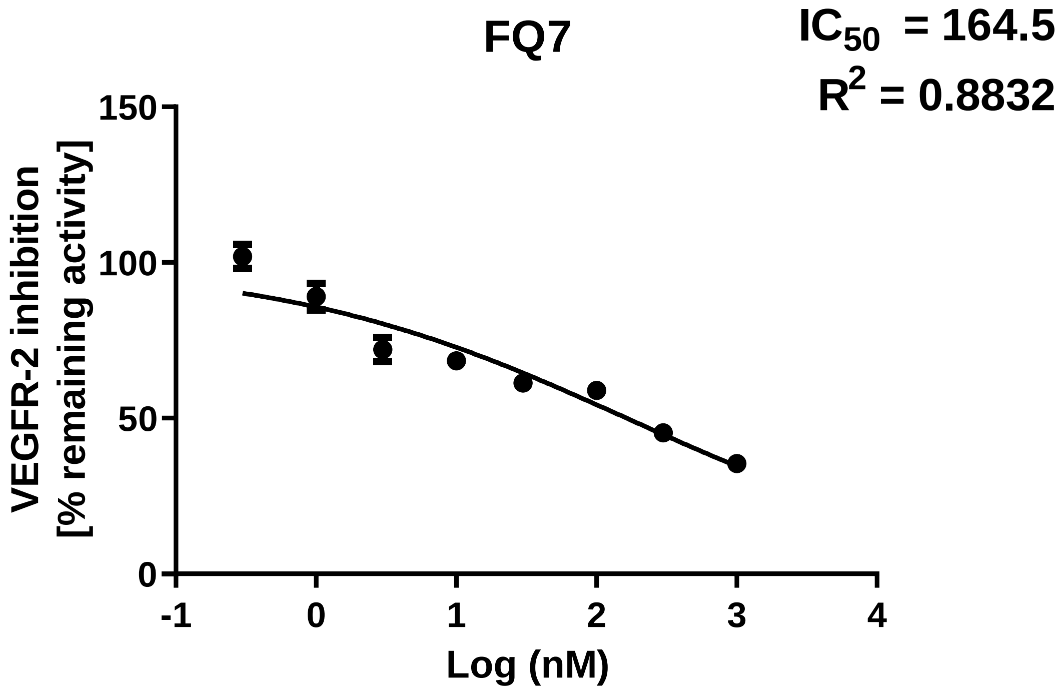

VEGFR-2 assay of sorafenib

Best-fit values	
LogIC50	1.730
HillSlope	-0.4752
IC50	53.65

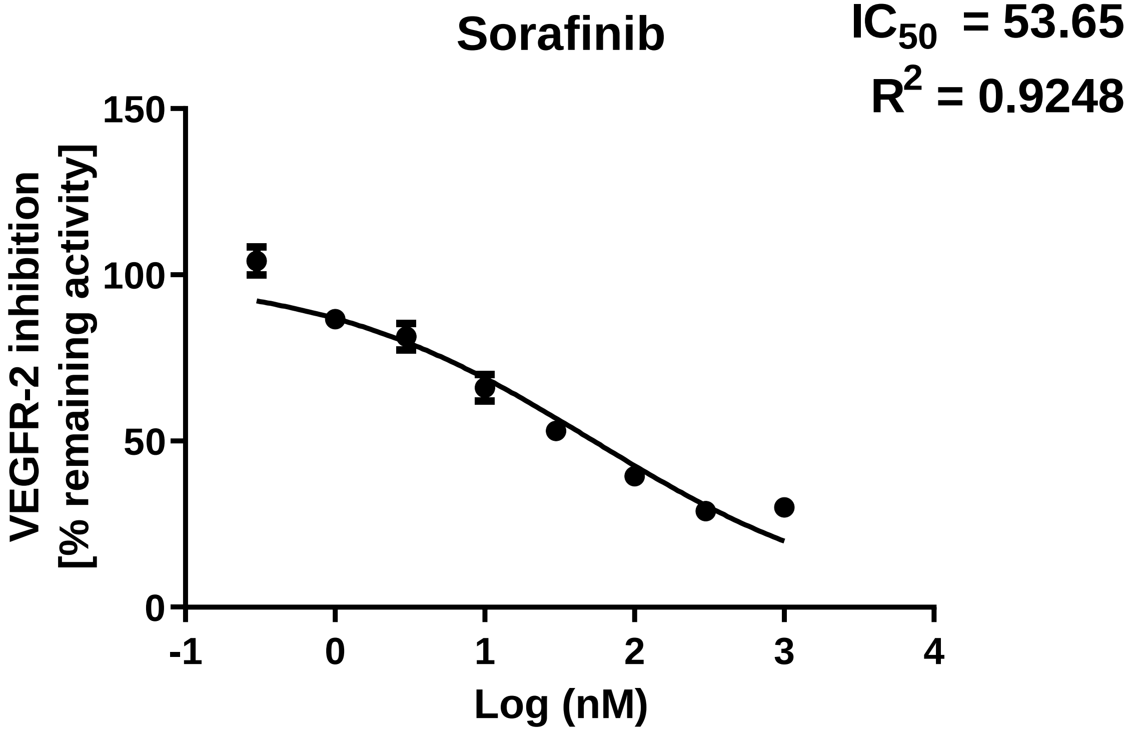

S.6. *In silico* cytotoxicity report

10a **TOPKAT_Ames_Mutagenicity**

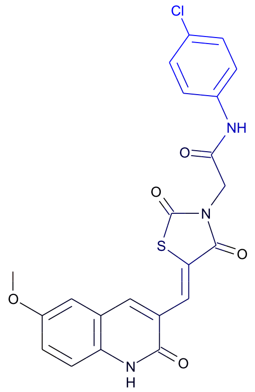

$C_{22}H_{16}ClN_3O_5S$
Molecular Weight: 469.89753
ALogP: 2.815
Rotatable Bonds: 5
Acceptors: 6
Donors: 2

Structural Similar Compounds

Name	Delavirdine	55256-55-8	Ochratoxin A
Structure	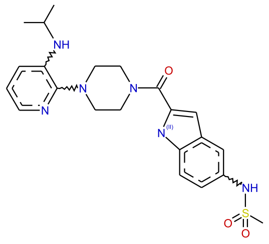	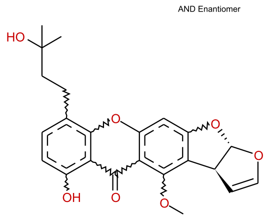	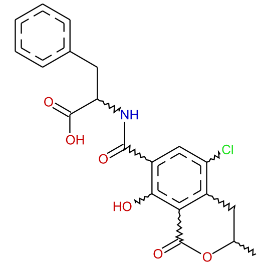
Actual Endpoint	Non-Mutagen	Mutagen	Non-Mutagen
Predicted Endpoint	Non-Mutagen	Mutagen	Non-Mutagen
Distance	0.605	0.608	0.614
Reference	Contrera, J.F., Matthews, E.J., Kruhlak, N.L., and Benz, R.D., Regulatory Toxicology and Pharmacology 2005, 313-323.	Kazius et. al., J. Med. Chem. (2005) 48, 312-320	EMIC

Model Prediction

Prediction: Non-Mutagen
Probability: 0.333
Enrichment: 0.596
Bayesian Score: -11.1
Mahalanobis Distance: 12.2
Mahalanobis Distance p-value: 0.000264

Prediction: Positive if the Bayesian score is above the estimated best cutoff value from minimizing the false positive and false negative rate.
Probability: The esimated probability that the sample is in the positive category. This assumes that the Bayesian score follows a normal distribution and is different from the prediction using a cutoff.
Enrichment: An estimate of enrichment, that is, the increased likelihood (versus random) of this sample being in the category.
Bayesian Score: The standard Laplacian-modified Bayesian score.
Mahalanobis Distance: The Mahalanobis distance (MD) is the distance to the center of the training data. The larger the MD, the less trustworthy the prediction.
Mahalanobis Distance p-value: The p-value gives the fraction of training data with an MD greater than or equal to the one for the given sample, assuming normally distributed data. The smaller the p-value, the less trustworthy the prediciton. For highly non-normal X properties (e.g., fingerprints), the MD p-value is wildly inaccurate.

Model Applicability

Unknown features are fingerprint features in the query molecule, but not found or appearing too infrequently in the training set.

1. All properties and OPS components are within expected ranges.

Feature Contribution

Top features for positive contribution

Fingerprint	Bit/Smiles	Feature Structure	Score	Mutagen in training set
SCFP_12	-577289847	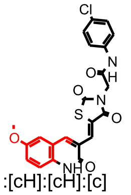 [*][c]1:[cH]:[cH]:[c](OC):[cH]:[c]:1C=[*]	0.399	9 out of 10

SCFP_12	-1971137145	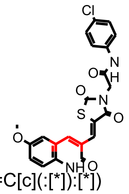 [*]C(=C[c](:[*]):[*])[*]	0.167	225 out of 333
SCFP_12	-496409612	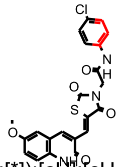 [*][c](:[*]):[cH]:[cH]:[*]	0.0771	2616 out of 4239

Top Features for negative contribution

**Fingerprint**	**Bit/Smiles**	**Feature Structure**	**Score**	**Mutagen in training set**
SCFP_12	-178170739	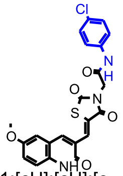 [*]N[c]1:[cH]:[cH]:[c](Cl):[cH]:[cH]:1	-1.57	1 out of 15
SCFP_12	-316886873	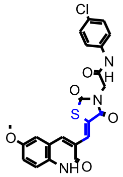 [*]\C=C\1/S[*][*]C1=[*]	-0.998	0 out of 3
SCFP_12	-1630519606	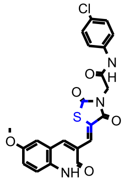 [*]=C1[*][*]C(=[*])S1	-0.998	0 out of 3

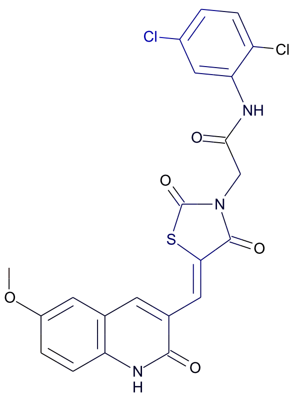

$C_{22}H_{15}Cl_2N_3O_5S$
Molecular Weight: 504.3426
ALogP: 3.479
Rotatable Bonds: 5
Acceptors: 6
Donors: 2

Model Prediction

Prediction: Non-Mutagen
Probability: 0.46
Enrichment: 0.824
Bayesian Score: -8.4
Mahalanobis Distance: 12.3
Mahalanobis Distance p-value: 0.000231

Prediction: Positive if the Bayesian score is above the estimated best cutoff value from minimizing the false positive and false negative rate.
Probability: The esimated probability that the sample is in the positive category. This assumes that the Bayesian score follows a normal distribution and is different from the prediction using a cutoff.
Enrichment: An estimate of enrichment, that is, the increased likelihood (versus random) of this sample being in the category.
Bayesian Score: The standard Laplacian-modified Bayesian score.
Mahalanobis Distance: The Mahalanobis distance (MD) is the distance to the center of the training data. The larger the MD, the less trustworthy the prediction.
Mahalanobis Distance p-value: The p-value gives the fraction of training data with an MD greater than or equal to the one for the given sample, assuming normally distributed data. The smaller the p-value, the less trustworthy the prediciton. For highly non-normal X properties (e.g., fingerprints), the MD p-value is wildly inaccurate.

Structural Similar Compounds

Name	55256-55-8	80266-02-0	83621-06-1
Structure	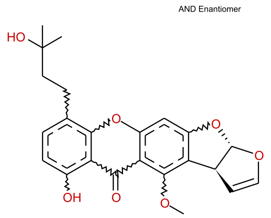	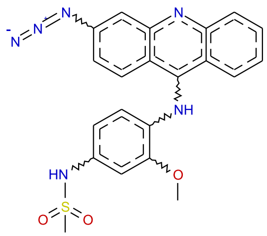	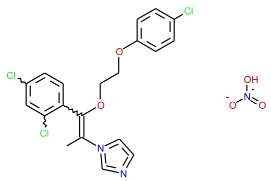
Actual Endpoint	Mutagen	Mutagen	Non-Mutagen
Predicted Endpoint	Mutagen	Mutagen	Non-Mutagen
Distance	0.633	0.636	0.637
Reference	Kazius et. al., J. Med. Chem. (2005) 48, 312-320	Kazius et. al., J. Med. Chem. (2005) 48, 312-320	Kazius et. al., J. Med. Chem. (2005) 48, 312-320

Model Applicability

Unknown features are fingerprint features in the query molecule, but not found or appearing too infrequently in the training set.

1. All properties and OPS components are within expected ranges.

Feature Contribution

Top features for positive contribution

Fingerprint	Bit/Smiles	Feature Structure	Score	Mutagen in training set
SCFP_12	1562412908	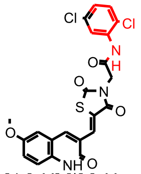 [*]N[c]1:[cH]:[*]:[cH]:[cH]:[c]:1Cl	0.442	5 out of 5

SCFP_12	-345817764	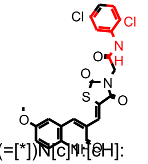 [*]C(=[*])N[c]1:[cH]:[c]([*]):[*]:[cH]:[c]:1Cl	0.442	5 out of 5
SCFP_12	-577289847	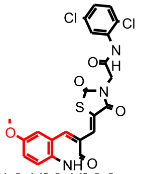 [*][c]1:[cH]:[cH]:[c](OC):[cH]:[c]:1C=[*]	0.399	9 out of 10

Top Features for negative contribution

Fingerprint	Bit/Smiles	Feature Structure	Score	Mutagen in training set
SCFP_12	-1630519606	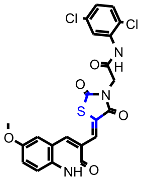 [*]=C1[*][*]C(=[*])S1	-0.998	0 out of 3
SCFP_12	-316886873	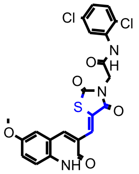 [*]\C=C\1/S[*][*]C1=[*]	-0.998	0 out of 3
SCFP_12	1907954607	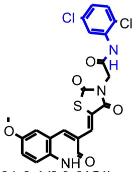 [*]N[c]1:[cH]:[c](Cl):[cH]:[cH]:[c]:1[*]	-0.762	0 out of 2

14a

TOPKAT_Ames_Mutagenicity

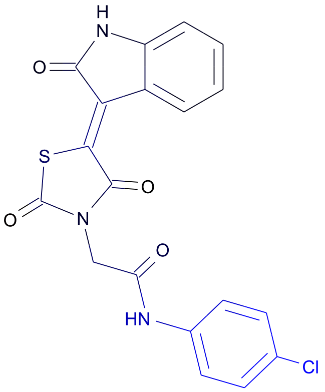

$C_{19}H_{12}ClN_3O_4S$
Molecular Weight: 413.83428
ALogP: 2.363
Rotatable Bonds: 3
Acceptors: 5
Donors: 2

Model Prediction

Prediction: Non-Mutagen
Probability: 0.35
Enrichment: 0.626
Bayesian Score: -10.7
Mahalanobis Distance: 11.5
Mahalanobis Distance p-value: 0.0055

Prediction: Positive if the Bayesian score is above the estimated best cutoff value from minimizing the false positive and false negative rate.
Probability: The esimated probability that the sample is in the positive category. This assumes that the Bayesian score follows a normal distribution and is different from the prediction using a cutoff.
Enrichment: An estimate of enrichment, that is, the increased likelihood (versus random) of this sample being in the category.
Bayesian Score: The standard Laplacian-modified Bayesian score.
Mahalanobis Distance: The Mahalanobis distance (MD) is the distance to the center of the training data. The larger the MD, the less trustworthy the prediction.
Mahalanobis Distance p-value: The p-value gives the fraction of training data with an MD greater than or equal to the one for the given sample, assuming normally distributed data. The smaller the p-value, the less trustworthy the prediciton. For highly non-normal X properties (e.g., fingerprints), the MD p-value is wildly inaccurate.

Structural Similar Compounds

Name	97919-22-7	98644-23-6	Chlorendic acid
Structure	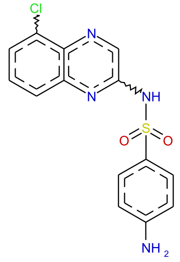	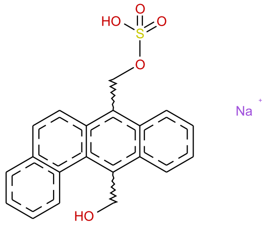	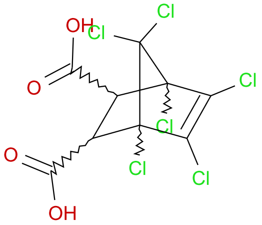
Actual Endpoint	Non-Mutagen	Mutagen	Non-Mutagen
Predicted Endpoint	Non-Mutagen	Mutagen	Non-Mutagen
Distance	0.581	0.600	0.602
Reference	Kazius et. al., J. Med. Chem. (2005) 48, 312-320	Kazius et. al., J. Med. Chem. (2005) 48, 312-320	Environ. Mol. Mut. 16(Suppl 18):1-14;1990

Model Applicability

Unknown features are fingerprint features in the query molecule, but not found or appearing too infrequently in the training set.

1. All properties and OPS components are within expected ranges.

Feature Contribution

Top features for positive contribution

Fingerprint	Bit/Smiles	Feature Structure	Score	Mutagen in training set
SCFP_12	-1379591900	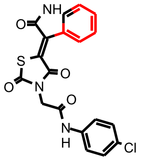 [*][c]1:[*]:[cH]:[cH]:[cH]:[cH]:1	0.108	1480 out of 2326

SCFP_12	-834984590	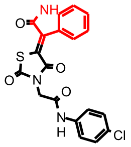 [*]=C1N[c]2:[cH]:[cH]:[cH]:[cH]:[c]:2C1=[*]	0.1	2 out of 3
SCFP_12	-496409612	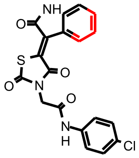 [*][c](:[*]):[cH]:[cH]:[*]	0.0771	2616 out of 4239

Top Features for negative contribution

Fingerprint	Bit/Smiles	Feature Structure	Score	Mutagen in training set
SCFP_12	-178170739	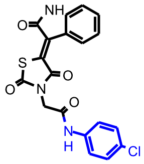 [*]N[c]1:[cH]:[cH]:[c](Cl):[cH]:[cH]:1	-1.57	1 out of 15
SCFP_12	-1630519606	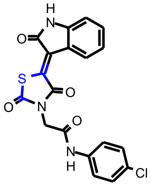 [*]=C1[*][*]C(=[*])S1	-0.998	0 out of 3
SCFP_12	-316886873	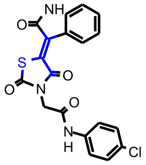 [*]\C=C\1/S[*][*]C1=[*]	-0.998	0 out of 3

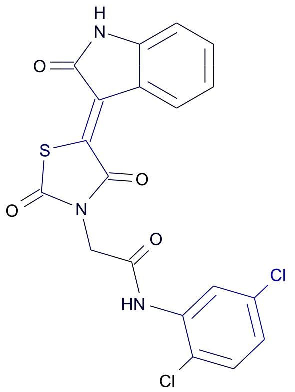

$C_{19}H_{11}Cl_2N_3O_4S$
Molecular Weight: 448.27933
ALogP: 3.028
Rotatable Bonds: 3
Acceptors: 5
Donors: 2

Model Prediction

Prediction: Non-Mutagen
Probability: 0.475
Enrichment: 0.851
Bayesian Score: -8.07
Mahalanobis Distance: 11.6
Mahalanobis Distance p-value: 0.0049

Prediction: Positive if the Bayesian score is above the estimated best cutoff value from minimizing the false positive and false negative rate.
Probability: The esimated probability that the sample is in the positive category. This assumes that the Bayesian score follows a normal distribution and is different from the prediction using a cutoff.
Enrichment: An estimate of enrichment, that is, the increased likelihood (versus random) of this sample being in the category.
Bayesian Score: The standard Laplacian-modified Bayesian score.
Mahalanobis Distance: The Mahalanobis distance (MD) is the distance to the center of the training data. The larger the MD, the less trustworthy the prediction.
Mahalanobis Distance p-value: The p-value gives the fraction of training data with an MD greater than or equal to the one for the given sample, assuming normally distributed data. The smaller the p-value, the less trustworthy the prediciton. For highly non-normal X properties (e.g., fingerprints), the MD p-value is wildly inaccurate.

Structural Similar Compounds

Name	98644-23-6	TETRABROMOPHTHALIC ACID	Chlorendic acid
Structure	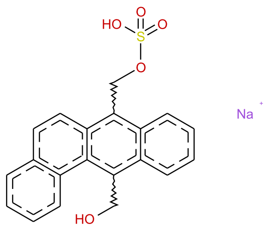	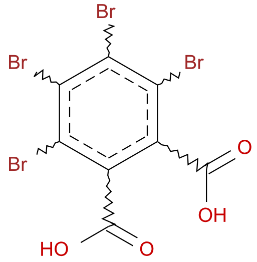	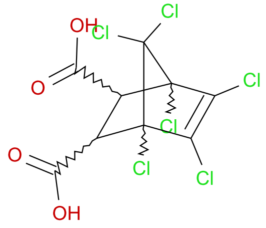
Actual Endpoint	Mutagen	Non-Mutagen	Non-Mutagen
Predicted Endpoint	Mutagen	Non-Mutagen	Non-Mutagen
Distance	0.614	0.617	0.620
Reference	Kazius et. al., J. Med. Chem. (2005) 48, 312-320	EMIC	Environ. Mol. Mut. 16(Suppl 18):1-14;1990

Model Applicability

Unknown features are fingerprint features in the query molecule, but not found or appearing too infrequently in the training set.

1. All properties and OPS components are within expected ranges.

Feature Contribution

Top features for positive contribution				
Fingerprint	Bit/Smiles	Feature Structure	Score	Mutagen in training set
SCFP_12	-345817764	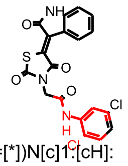 [*]C(=[*])N[c]1:[cH]:[c]([*]):[*]:[cH]:[c]:1Cl	0.442	5 out of 5

SCFP_12	1562412908	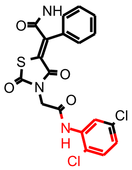 [*]N[c]1:[cH]:[*]:[cH]:[cH]:[c]:1Cl	0.442	5 out of 5
SCFP_12	-1490122748	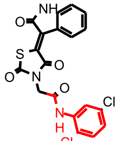 [*]C(=[*])N[c]1:[cH]: [c]([*]):[cH]:[cH]:[c]:1Cl	0.241	1 out of 1
**Top Features for negative contribution**				
**Fingerprint**	**Bit/Smiles**	**Feature Structure**	**Score**	**Mutagen in training set**
SCFP_12	-1630519606	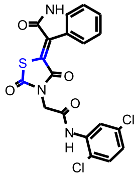 [*]=C1[*][*]C(=[*])S1	-0.998	0 out of 3
SCFP_12	-316886873	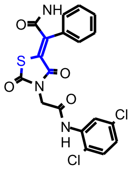 [*]\C=C\1/S[*][*]C1=[*]	-0.998	0 out of 3
SCFP_12	1907954607	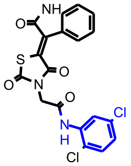 [*]N[c]1:[cH]:[c](Cl) :[cH]:[cH]:[c]:1[*]	-0.762	0 out of 2

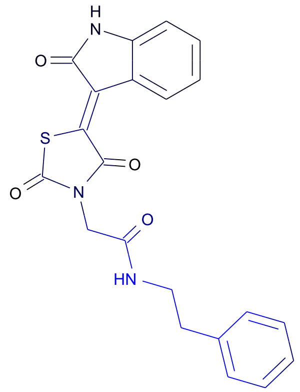

$C_{21}H_{17}N_3O_4S$
Molecular Weight: 407.44237
ALogP: 2.027
Rotatable Bonds: 5
Acceptors: 5
Donors: 2

Model Prediction

Prediction: Non-Mutagen
Probability: 0.251
Enrichment: 0.449
Bayesian Score: -12.9
Mahalanobis Distance: 12.9
Mahalanobis Distance p-value: 8.1e-006

Prediction: Positive if the Bayesian score is above the estimated best cutoff value from minimizing the false positive and false negative rate.
Probability: The esimated probability that the sample is in the positive category. This assumes that the Bayesian score follows a normal distribution and is different from the prediction using a cutoff.
Enrichment: An estimate of enrichment, that is, the increased likelihood (versus random) of this sample being in the category.
Bayesian Score: The standard Laplacian-modified Bayesian score.
Mahalanobis Distance: The Mahalanobis distance (MD) is the distance to the center of the training data. The larger the MD, the less trustworthy the prediction.
Mahalanobis Distance p-value: The p-value gives the fraction of training data with an MD greater than or equal to the one for the given sample, assuming normally distributed data. The smaller the p-value, the less trustworthy the prediciton. For highly non-normal X properties (e.g., fingerprints), the MD p-value is wildly inaccurate.

Structural Similar Compounds

Name	89784-39-4	PENICILLIN G POTASSIUM	4825-86-9
Structure	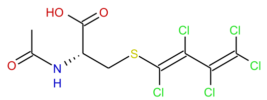	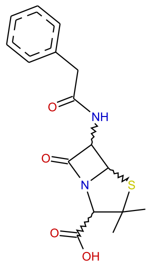	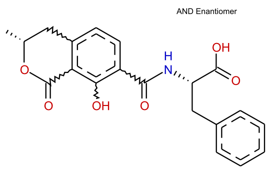
Actual Endpoint	Mutagen	Non-Mutagen	Non-Mutagen
Predicted Endpoint	Mutagen	Non-Mutagen	Non-Mutagen
Distance	0.587	0.591	0.592
Reference	Kazius et. al., J. Med. Chem. (2005) 48, 312-320	EMIC	Kazius et. al., J. Med. Chem. (2005) 48, 312-320

Model Applicability

Unknown features are fingerprint features in the query molecule, but not found or appearing too infrequently in the training set.

1. All properties and OPS components are within expected ranges.

Feature Contribution

Top features for positive contribution

Fingerprint	Bit/Smiles	Feature Structure	Score	Mutagen in training set
SCFP_12	-1379591900	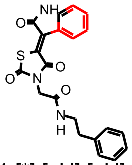 [*][c]1:[*]:[cH]:[cH]:[cH]:[cH]:1	0.108	1480 out of 2326

SCFP_12	-834984590	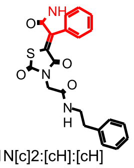 [*]=C1N[c]2:[cH]:[cH]:[cH]:[cH]:[c]:2C1=[*]	0.1	2 out of 3
SCFP_12	-496409612	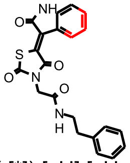 [*][c](:[*]):[cH]:[cH]:[*]	0.0771	2616 out of 4239

Top Features for negative contribution

Fingerprint	Bit/Smiles	Feature Structure	Score	Mutagen in training set
SCFP_12	-1849236245	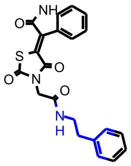 [*]NCC[c](:[cH]:[*]):[cH]:[*]	-1.61	0 out of 7
SCFP_12	647859032	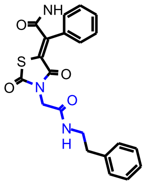 [*]CNC(=O)CN([*])[*]	-1.19	0 out of 4
SCFP_12	-1630519606	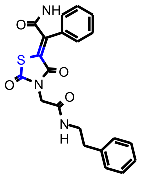 [*]=C1[*][*]C(=[*])S1	-0.998	0 out of 3

Sorafenib

TOPKAT_Ames_Mutagenicity

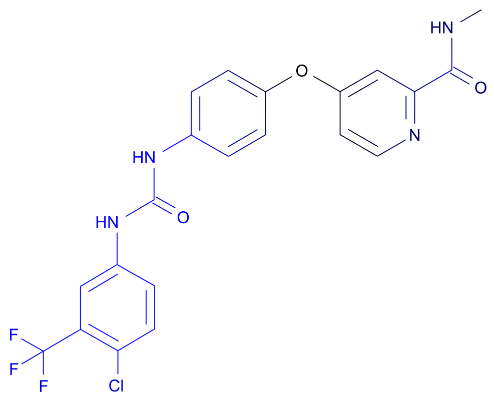

$C_{21}H_{16}ClF_3N_4O_3$

Molecular Weight: 464.82494

ALogP: 4.175

Rotatable Bonds: 6

Acceptors: 4

Donors: 3

Structural Similar Compounds

Name	GLYBURIDE	38914-96-4	93957-54-1
Structure	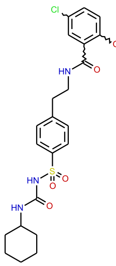	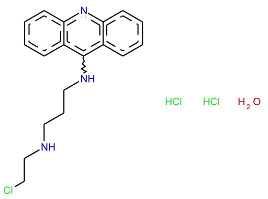	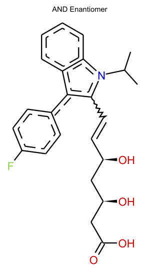
Actual Endpoint	Non-Mutagen	Mutagen	Non-Mutagen
Predicted Endpoint	Non-Mutagen	Mutagen	Non-Mutagen
Distance	0.590	0.592	0.600
Reference	PDR 1994	Kazius et. al., J. Med. Chem. (2005) 48, 312-320	US Environmental Protection Agency at http://www.epa.gov/NCCT/dsstox/sdf_isscan_external.html

Model Prediction

Prediction: Non-Mutagen

Probability: 0.0531

Enrichment: 0.0951

Bayesian Score: -19.7

Mahalanobis Distance: 13.1

Mahalanobis Distance p-value: 2.73e-006

Prediction: Positive if the Bayesian score is above the estimated best cutoff value from minimizing the false positive and false negative rate.
Probability: The esimated probability that the sample is in the positive category. This assumes that the Bayesian score follows a normal distribution and is different from the prediction using a cutoff.
Enrichment: An estimate of enrichment, that is, the increased likelihood (versus random) of this sample being in the category.
Bayesian Score: The standard Laplacian-modified Bayesian score.
Mahalanobis Distance: The Mahalanobis distance (MD) is the distance to the center of the training data. The larger the MD, the less trustworthy the prediction.
Mahalanobis Distance p-value: The p-value gives the fraction of training data with an MD greater than or equal to the one for the given sample, assuming normally distributed data. The smaller the p-value, the less trustworthy the prediciton. For highly non-normal X properties (e.g., fingerprints), the MD p-value is wildly inaccurate.

Model Applicability

Unknown features are fingerprint features in the query molecule, but not found or appearing too infrequently in the training set.

1. All properties and OPS components are within expected ranges.

Feature Contribution

Top features for positive contribution				
**Fingerprint**	**Bit/Smiles**	**Feature Structure**	**Score**	**Mutagen in training set**
SCFP_12	-347281112	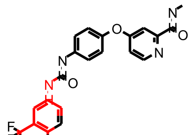 [*]Nc1:c:[cH]:[*]:[c]([*]):[c](:[cH]:1)C([*])([*])[*]	0.337	18 out of 22

SCFP_12	1208843554	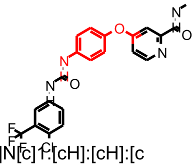<br>[*]N[c]1:[cH]:[cH]:[c<br>](O[c](:[*]):[*]):[c<br>H]:[cH]:1	0.337	6 out of 7
SCFP_12	-1943080297	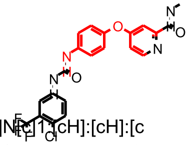<br>[*]N[c]1:[cH]:[cH]:[c<br>](O[c]2:[cH]:[cH]:[*<br>]:[c]([*]):[cH]:2):[<br>cH]:[cH]:1	0.304	5 out of 6
**Top Features for negative contribution**				
**Fingerprint**	**Bit/Smiles**	**Feature Structure**	**Score**	**Mutagen in training set**
SCFP_12	816802409	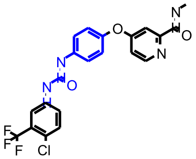<br>[*]NC(=O)N[c]1:[cH]:[<br>cH]:[c]([*]):[cH]:[c<br>H]:1	-1.82	0 out of 9
SCFP_12	-1903175541	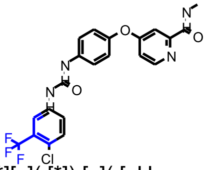<br>[*][c](:[*]):[c](:[cH<br>]:[*])C(F)(F)F	-1.51	3 out of 30
SCFP_12	-300280774	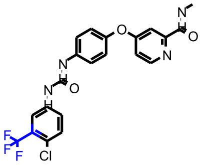<br>[*]:[c](:[*])C(F)(F)F	-1.51	3 out of 30

Sunitinib

TOPKAT_Ames_Mutagenicity

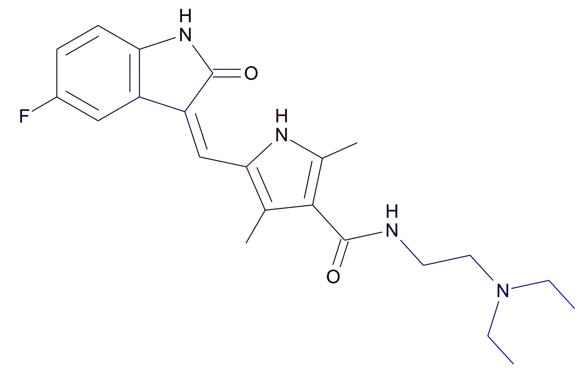

$C_{22}H_{27}FN_4O_2$
Molecular Weight: 398.47378
ALogP: 2.997
Rotatable Bonds: 7
Acceptors: 3
Donors: 3

Structural Similar Compounds

Name	38914-96-4	93957-54-1	IA 4 N-oxide
Structure	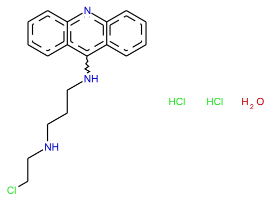	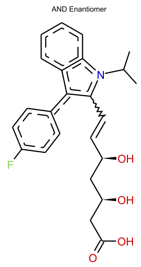	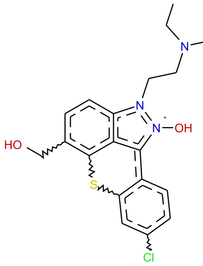
Actual Endpoint	Mutagen	Non-Mutagen	Mutagen
Predicted Endpoint	Mutagen	Non-Mutagen	Mutagen
Distance	0.597	0.602	0.610
Reference	Kazius et. al., J. Med. Chem. (2005) 48, 312-320	US Environmental Protection Agency at http://www.epa.gov/NCCT/dsstox/sdf_isscan_external.html	Contrera, J.F., Matthews, E.J., Kruhlak, N.L., and Benz, R.D., Regulatory Toxicology and Pharmacology 2005, 313-323.

Model Prediction

Prediction: Non-Mutagen
Probability: 0.623
Enrichment: 1.11
Bayesian Score: -4.39
Mahalanobis Distance: 14.8
Mahalanobis Distance p-value: 8.05e-012

Prediction: Positive if the Bayesian score is above the estimated best cutoff value from minimizing the false positive and false negative rate.
Probability: The esimated probability that the sample is in the positive category. This assumes that the Bayesian score follows a normal distribution and is different from the prediction using a cutoff.
Enrichment: An estimate of enrichment, that is, the increased likelihood (versus random) of this sample being in the category.
Bayesian Score: The standard Laplacian-modified Bayesian score.
Mahalanobis Distance: The Mahalanobis distance (MD) is the distance to the center of the training data. The larger the MD, the less trustworthy the prediction.
Mahalanobis Distance p-value: The p-value gives the fraction of training data with an MD greater than or equal to the one for the given sample, assuming normally distributed data. The smaller the p-value, the less trustworthy the prediciton. For highly non-normal X properties (e.g., fingerprints), the MD p-value is wildly inaccurate.

Model Applicability

Unknown features are fingerprint features in the query molecule, but not found or appearing too infrequently in the training set.

1. All properties and OPS components are within expected ranges.

Feature Contribution

Top features for positive contribution

Fingerprint	Bit/Smiles	Feature Structure	Score	Mutagen in training set
SCFP_12	1024473425	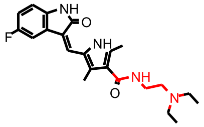 [*]N([*])CCNC(=[*])[*]	0.442	13 out of 14

SCFP_12	1232358329	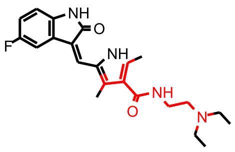<br>[*]N([*])CCNC(=O)[c]1:[c]([*]):[*]:[*]:[c]:1[*]	0.424	11 out of 12
SCFP_12	820030350	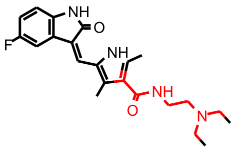<br>[*]CN(C[*])CCNC(=O)[c](:[*]):[*]	0.424	11 out of 12
**Top Features for negative contribution**				
**Fingerprint**	**Bit/Smiles**	**Feature Structure**	**Score**	**Mutagen in training set**
SCFP_12	-495096007	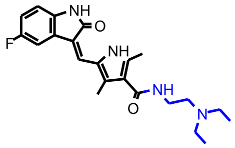<br>[*]NCCN(CC)CC	-0.762	0 out of 2
SCFP_12	1798334293	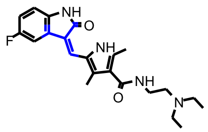<br>[*]C(=C1C(=[*])[*][*]:[c]1:[*])[*]	-0.525	5 out of 16
SCFP_12	32698025	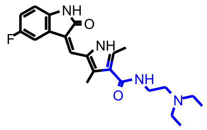<br>[*]:[c](:[*])C(=O)NCCN(CC)CC	-0.452	0 out of 1

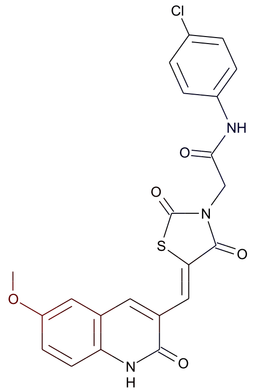

$C_{22}H_{16}ClN_3O_5S$
Molecular Weight: 469.89753
ALogP: 2.815
Rotatable Bonds: 5
Acceptors: 6
Donors: 2

Model Prediction

Prediction: Toxic
Probability: 0.619
Enrichment: 1.18
Bayesian Score: 1.83
Mahalanobis Distance: 12.2
Mahalanobis Distance p-value: 1.57e-005

Prediction: Positive if the Bayesian score is above the estimated best cutoff value from minimizing the false positive and false negative rate.
Probability: The esimated probability that the sample is in the positive category. This assumes that the Bayesian score follows a normal distribution and is different from the prediction using a cutoff.
Enrichment: An estimate of enrichment, that is, the increased likelihood (versus random) of this sample being in the category.
Bayesian Score: The standard Laplacian-modified Bayesian score.
Mahalanobis Distance: The Mahalanobis distance (MD) is the distance to the center of the training data. The larger the MD, the less trustworthy the prediction.
Mahalanobis Distance p-value: The p-value gives the fraction of training data with an MD greater than or equal to the one for the given sample, assuming normally distributed data. The smaller the p-value, the less trustworthy the prediciton. For highly non-normal X properties (e.g., fingerprints), the MD p-value is wildly inaccurate.

Structural Similar Compounds

Name	Ochratoxin a	Amsacrine	Acemetacin
Structure	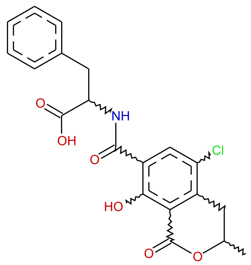	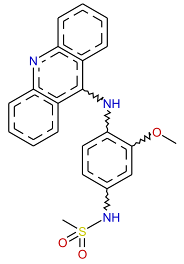	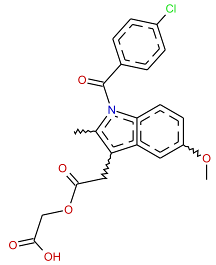
Actual Endpoint	Toxic	Toxic	Non-Toxic
Predicted Endpoint	Toxic	Toxic	Non-Toxic
Distance	0.608	0.621	0.648
Reference	Toxicol Appl Pharmacol 37(2):331-8; 1976	Fundam Appl Toxicol 7(2):214-20; 1986	Oyo Yakuri 22(6):777-786; 1981

Model Applicability

Unknown features are fingerprint features in the query molecule, but not found or appearing too infrequently in the training set.

1. All properties and OPS components are within expected ranges.

Feature Contribution

Top features for positive contribution				
**Fingerprint**	**Bit/Smiles**	**Feature Structure**	**Score**	**Toxic in training set**
SCFP_6	1237755852	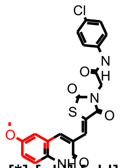 [*][c]1:[*]:[cH]:[cH]:[c](OC):[cH]:1	0.453	8 out of 9

SCFP_6	-1971137145	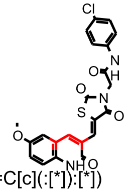 [*]C(=C[c](:[*]):[*])[*]	0.431	7 out of 8
SCFP_6	591469355	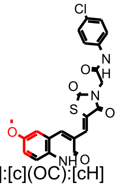 [*]:[cH]:[c](OC):[cH]:[*]	0.411	10 out of 12
**Top Features for negative contribution**				
**Fingerprint**	**Bit/Smiles**	**Feature Structure**	**Score**	**Toxic in training set**
SCFP_6	399659969	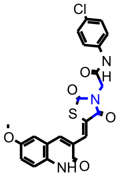 [*]CN1C(=[*])[*][*]C1=[*]	-0.526	3 out of 11
SCFP_6	1420330831	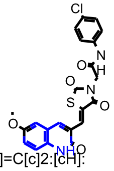 [*]=C1[*]=C[c]2:[cH]:[*]:[cH]:[cH]:[c]:2N1	-0.422	0 out of 1
SCFP_6	2097618059	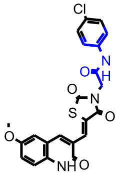 [*]CC(=O)N[c](:[cH]:[*]):[cH]:[*]	-0.422	0 out of 1

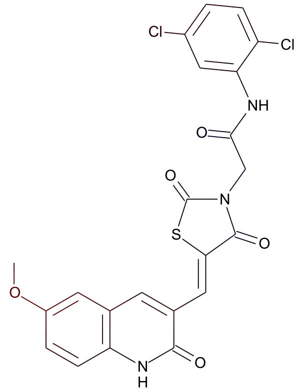

$C_{22}H_{15}Cl_2N_3O_5S$
Molecular Weight: 504.3426
ALogP: 3.479
Rotatable Bonds: 5
Acceptors: 6
Donors: 2

Model Prediction

Prediction: Toxic

Probability: 0.662

Enrichment: 1.26

Bayesian Score: 2.95

Mahalanobis Distance: 12.2

Mahalanobis Distance p-value: 1.58e-005

Prediction: Positive if the Bayesian score is above the estimated best cutoff value from minimizing the false positive and false negative rate.
Probability: The esimated probability that the sample is in the positive category. This assumes that the Bayesian score follows a normal distribution and is different from the prediction using a cutoff.
Enrichment: An estimate of enrichment, that is, the increased likelihood (versus random) of this sample being in the category.
Bayesian Score: The standard Laplacian-modified Bayesian score.
Mahalanobis Distance: The Mahalanobis distance (MD) is the distance to the center of the training data. The larger the MD, the less trustworthy the prediction.
Mahalanobis Distance p-value: The p-value gives the fraction of training data with an MD greater than or equal to the one for the given sample, assuming normally distributed data. The smaller the p-value, the less trustworthy the prediciton. For highly non-normal X properties (e.g., fingerprints), the MD p-value is wildly inaccurate.

Structural Similar Compounds

Name	Amsacrine	Ochratoxin a	Acemetacin
Structure	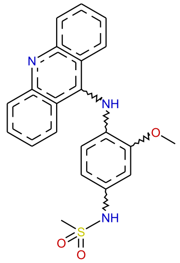	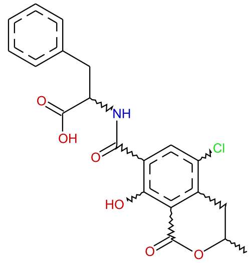	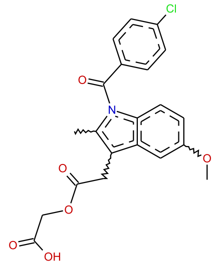
Actual Endpoint	Toxic	Toxic	Non-Toxic
Predicted Endpoint	Toxic	Toxic	Non-Toxic
Distance	0.626	0.633	0.648
Reference	Fundam Appl Toxicol 7(2):214-20; 1986	Toxicol Appl Pharmacol 37(2):331-8; 1976	Oyo Yakuri 22(6):777-786; 1981

Model Applicability

Unknown features are fingerprint features in the query molecule, but not found or appearing too infrequently in the training set.

1. All properties and OPS components are within expected ranges.

Feature Contribution

Top features for positive contribution				
**Fingerprint**	**Bit/Smiles**	**Feature Structure**	**Score**	**Toxic in training set**
SCFP_6	1237755852	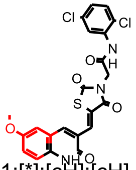 [*][c]1:[*]:[cH]:[cH]:[c](OC):[cH]:1	0.453	8 out of 9

SCFP_6	-1971137145	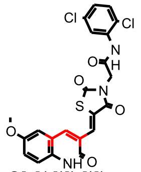 [*]C(=C[c](:[*]):[*])[*]	0.431	7 out of 8
SCFP_6	591469355	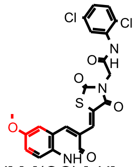 [*]:[cH]:[c](OC):[cH]:[*]	0.411	10 out of 12

Top Features for negative contribution

Fingerprint	Bit/Smiles	Feature Structure	Score	Toxic in training set
SCFP_6	399659969	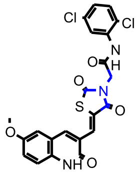 [*]CN1C(=[*])[*][*]C1=[*]	-0.526	3 out of 11
SCFP_6	2097618059	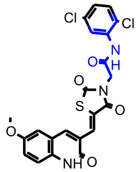 [*]CC(=O)N[c](:[cH]:[*]):[cH]:[*]	-0.422	0 out of 1
SCFP_6	1420330831	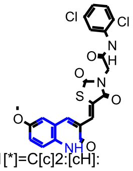 [*]=C1[*]=C[c]2:[cH]:[*]:[cH]:[cH]:[c]:2N1	-0.422	0 out of 1

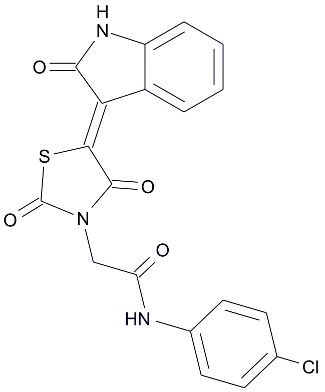

$C_{19}H_{12}ClN_3O_4S$
Molecular Weight: 413.83428
ALogP: 2.363
Rotatable Bonds: 3
Acceptors: 5
Donors: 2

Model Prediction

Prediction: Non-Toxic
Probability: 0.501
Enrichment: 0.953
Bayesian Score: -1.26
Mahalanobis Distance: 8.02
Mahalanobis Distance p-value: 0.595

Prediction: Positive if the Bayesian score is above the estimated best cutoff value from minimizing the false positive and false negative rate.
Probability: The esimated probability that the sample is in the positive category. This assumes that the Bayesian score follows a normal distribution and is different from the prediction using a cutoff.
Enrichment: An estimate of enrichment, that is, the increased likelihood (versus random) of this sample being in the category.
Bayesian Score: The standard Laplacian-modified Bayesian score.
Mahalanobis Distance: The Mahalanobis distance (MD) is the distance to the center of the training data. The larger the MD, the less trustworthy the prediction.
Mahalanobis Distance p-value: The p-value gives the fraction of training data with an MD greater than or equal to the one for the given sample, assuming normally distributed data. The smaller the p-value, the less trustworthy the prediciton. For highly non-normal X properties (e.g., fingerprints), the MD p-value is wildly inaccurate.

Structural Similar Compounds

Name	D&C Yellow 8	Sulfonylurea Gliclazide	Tiaramide .HCl (Free base form)
Structure	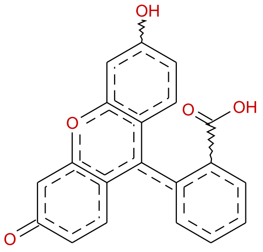	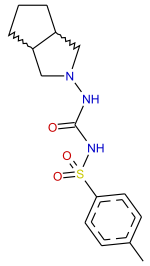	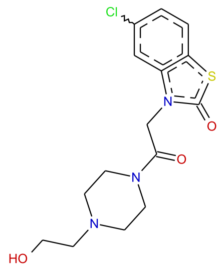
Actual Endpoint	Non-Toxic	Toxic	Toxic
Predicted Endpoint	Non-Toxic	Toxic	Toxic
Distance	0.612	0.620	0.623
Reference	Food Chem Toxicol 24:819-823; 1986	Yakuri to Chiryo 9:3551-3571; 1981	Arzneimittelforschung 23(4):504-8; 1973

Model Applicability

Unknown features are fingerprint features in the query molecule, but not found or appearing too infrequently in the training set.

1. All properties and OPS components are within expected ranges.

Feature Contribution

Top features for positive contribution				
**Fingerprint**	**Bit/Smiles**	**Feature Structure**	**Score**	**Toxic in training set**
SCFP_6	199205675	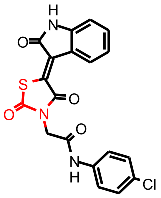 [*]N1[*][*]SC1=O	0.271	1 out of 1

SCFP_6	2102703671	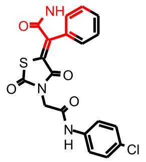 [*]C1=[*][c](:[*]):[c](NC1=O):[cH]:[*]	0.271	1 out of 1
SCFP_6	-587539325	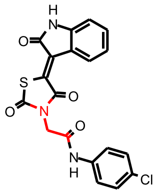 [*]N([*])CC(=[*])[*]	0.271	1 out of 1
**Top Features for negative contribution**				
**Fingerprint**	**Bit/Smiles**	**Feature Structure**	**Score**	**Toxic in training set**
SCFP_6	399659969	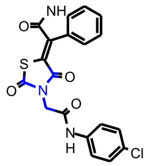 [*]CN1C(=[*])[*][*]C1 =[*]	-0.526	3 out of 11
SCFP_6	1420330831	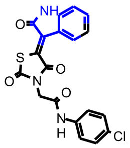 [*]=C1[*]=C[c]2:[cH]: [*]:[cH]:[cH]:[c]:2N 1	-0.422	0 out of 1
SCFP_6	2097618059	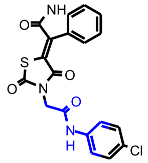 [*]CC(=O)N[c](:[cH]:[*]):[cH]:[*]	-0.422	0 out of 1

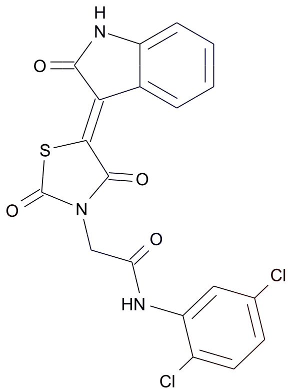

$C_{19}H_{11}Cl_2N_3O_4S$
Molecular Weight: 448.27933
ALogP: 3.028
Rotatable Bonds: 3
Acceptors: 5
Donors: 2

Model Prediction

Prediction: Non-Toxic
Probability: 0.51
Enrichment: 0.969
Bayesian Score: -1.03
Mahalanobis Distance: 8.01
Mahalanobis Distance p-value: 0.602

Prediction: Positive if the Bayesian score is above the estimated best cutoff value from minimizing the false positive and false negative rate.
Probability: The esimated probability that the sample is in the positive category. This assumes that the Bayesian score follows a normal distribution and is different from the prediction using a cutoff.
Enrichment: An estimate of enrichment, that is, the increased likelihood (versus random) of this sample being in the category.
Bayesian Score: The standard Laplacian-modified Bayesian score.
Mahalanobis Distance: The Mahalanobis distance (MD) is the distance to the center of the training data. The larger the MD, the less trustworthy the prediction.
Mahalanobis Distance p-value: The p-value gives the fraction of training data with an MD greater than or equal to the one for the given sample, assuming normally distributed data. The smaller the p-value, the less trustworthy the prediciton. For highly non-normal X properties (e.g., fingerprints), the MD p-value is wildly inaccurate.

Structural Similar Compounds

Name	D&C Yellow 8	Ochratoxin a	Amsacrine
Structure	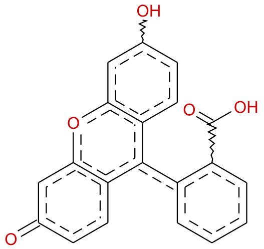	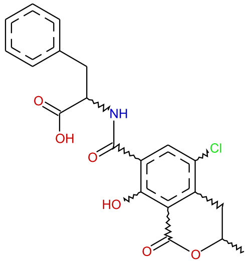	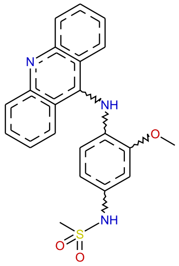
Actual Endpoint	Non-Toxic	Toxic	Toxic
Predicted Endpoint	Non-Toxic	Toxic	Toxic
Distance	0.628	0.645	0.649
Reference	Food Chem Toxicol 24:819-823; 1986	Toxicol Appl Pharmacol 37(2):331-8; 1976	Fundam Appl Toxicol 7(2):214-20; 1986

Model Applicability

Unknown features are fingerprint features in the query molecule, but not found or appearing too infrequently in the training set.

1. All properties and OPS components are within expected ranges.

Feature Contribution

Top features for positive contribution				
Fingerprint	Bit/Smiles	Feature Structure	Score	Toxic in training set
SCFP_6	2102703671	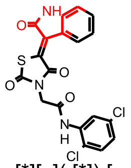 [*]C1=[*][c](:[*]):[c](NC1=O):[cH]:[*]	0.271	1 out of 1

SCFP_6	-534413823	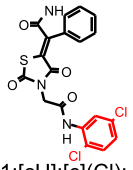 [*][c]1:[cH]:[c](Cl):[cH]:[cH]:[c]:1Cl	0.271	1 out of 1
SCFP_6	199205675	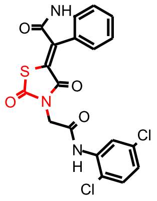 [*]N1[*][*]SC1=O	0.271	1 out of 1
**Top Features for negative contribution**				
**Fingerprint**	**Bit/Smiles**	**Feature Structure**	**Score**	**Toxic in training set**
SCFP_6	399659969	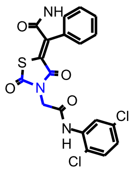 [*]CN1C(=[*])[*][*]C1=[*]	-0.526	3 out of 11
SCFP_6	2097618059	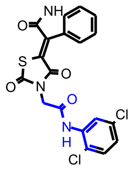 [*]CC(=O)N[c](:[cH]:[*]):[cH]:[*]	-0.422	0 out of 1
SCFP_6	1420330831	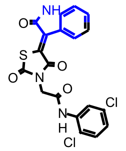 [*]=C1[*]=C[c]2:[cH]:[*]:[cH]:[cH]:[c]:2N1	-0.422	0 out of 1

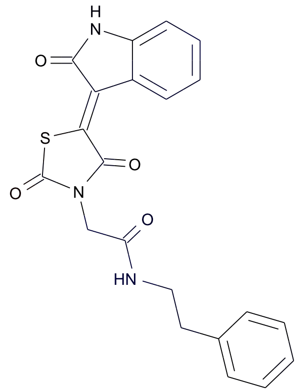

$C_{21}H_{17}N_3O_4S$

Molecular Weight: 407.44237

ALogP: 2.027

Rotatable Bonds: 5

Acceptors: 5

Donors: 2

Structural Similar Compounds

Name	Ochratoxin a	Tiaramide .HCl (Free base form)	Sulfonylurea Gliclazide
Structure	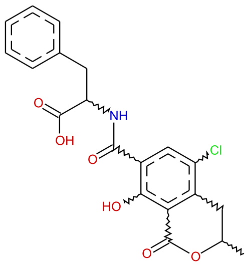	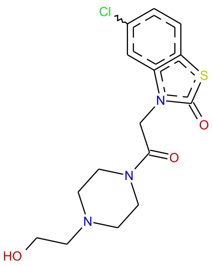	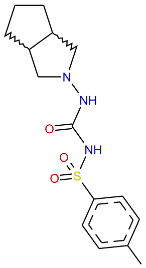
Actual Endpoint	Toxic	Toxic	Toxic
Predicted Endpoint	Toxic	Toxic	Toxic
Distance	0.610	0.624	0.635
Reference	Toxicol Appl Pharmacol 37(2):331-8; 1976	Arzneimittelforschung 23(4):504-8; 1973	Yakuri to Chiryo 9:3551-3571; 1981

Model Prediction

Prediction: Non-Toxic

Probability: 0.493

Enrichment: 0.937

Bayesian Score: -1.49

Mahalanobis Distance: 11.3

Mahalanobis Distance p-value: 0.000337

Prediction: Positive if the Bayesian score is above the estimated best cutoff value from minimizing the false positive and false negative rate.
Probability: The esimated probability that the sample is in the positive category. This assumes that the Bayesian score follows a normal distribution and is different from the prediction using a cutoff.
Enrichment: An estimate of enrichment, that is, the increased likelihood (versus random) of this sample being in the category.
Bayesian Score: The standard Laplacian-modified Bayesian score.
Mahalanobis Distance: The Mahalanobis distance (MD) is the distance to the center of the training data. The larger the MD, the less trustworthy the prediction.
Mahalanobis Distance p-value: The p-value gives the fraction of training data with an MD greater than or equal to the one for the given sample, assuming normally distributed data. The smaller the p-value, the less trustworthy the prediciton. For highly non-normal X properties (e.g., fingerprints), the MD p-value is wildly inaccurate.

Model Applicability

Unknown features are fingerprint features in the query molecule, but not found or appearing too infrequently in the training set.

1. All properties and OPS components are within expected ranges.

Feature Contribution

Top features for positive contribution

Fingerprint	Bit/Smiles	Feature Structure	Score	Toxic in training set
SCFP_6	2102703671	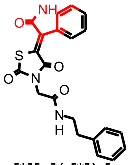 [*]C1=[*][c](:[*]):[c](NC1=O):[cH]:[*]	0.271	1 out of 1

SCFP_6	199205675	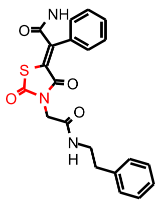 [*]N1[*][*]SC1=O	0.271	1 out of 1
SCFP_6	-587539325	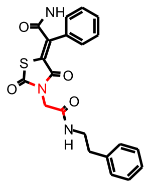 [*]N([*])CC(=[*])[*]	0.271	1 out of 1

Top Features for negative contribution

Fingerprint	Bit/Smiles	Feature Structure	Score	Toxic in training set
SCFP_6	2005026407	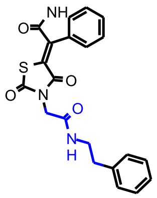 [*]CCNC(=O)C[*]	-0.718	0 out of 2
SCFP_6	399659969	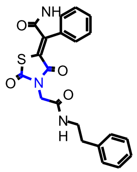 [*]CN1C(=[*])[*][*]C1 =[*]	-0.526	3 out of 11
SCFP_6	1420330831	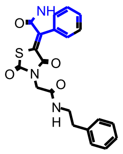 [*]=C1[*]=C[c]2:[cH]: [*]:[cH]:[cH]:[c]:2N 1	-0.422	0 out of 1

Sorafenib

TOPKAT_Developmental_Toxicity_Potential

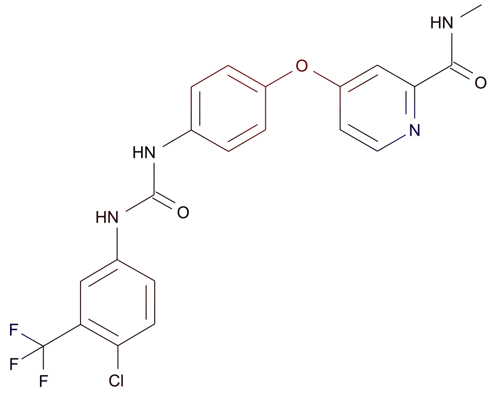

$C_{21}H_{16}ClF_3N_4O_3$

Molecular Weight: 464.82494

ALogP: 4.175

Rotatable Bonds: 6

Acceptors: 4

Donors: 3

Model Prediction

Prediction: Toxic

Probability: 0.592

Enrichment: 1.13

Bayesian Score: 1.15

Mahalanobis Distance: 12.6

Mahalanobis Distance p-value: 2.07e-006

Prediction: Positive if the Bayesian score is above the estimated best cutoff value from minimizing the false positive and false negative rate.
Probability: The esimated probability that the sample is in the positive category. This assumes that the Bayesian score follows a normal distribution and is different from the prediction using a cutoff.
Enrichment: An estimate of enrichment, that is, the increased likelihood (versus random) of this sample being in the category.
Bayesian Score: The standard Laplacian-modified Bayesian score.
Mahalanobis Distance: The Mahalanobis distance (MD) is the distance to the center of the training data. The larger the MD, the less trustworthy the prediction.
Mahalanobis Distance p-value: The p-value gives the fraction of training data with an MD greater than or equal to the one for the given sample, assuming normally distributed data. The smaller the p-value, the less trustworthy the prediciton. For highly non-normal X properties (e.g., fingerprints), the MD p-value is wildly inaccurate.

Structural Similar Compounds

Name	Chenodiol	Amsacrine	Ochratoxin a
Structure	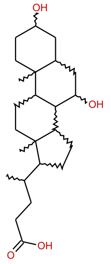	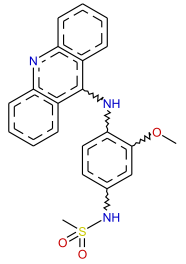	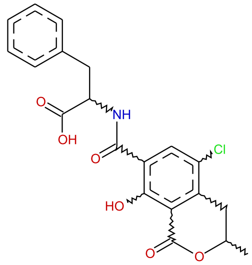
Actual Endpoint	Toxic	Toxic	Toxic
Predicted Endpoint	Toxic	Toxic	Toxic
Distance	0.631	0.637	0.644
Reference	Arch Int Pharm 246:149-158; 1980	Fundam Appl Toxicol 7(2):214-20; 1986	Toxicol Appl Pharmacol 37(2):331-8; 1976

Model Applicability

Unknown features are fingerprint features in the query molecule, but not found or appearing too infrequently in the training set.

1. All properties and OPS components are within expected ranges.

Feature Contribution

Top features for positive contribution

Fingerprint	Bit/Smiles	Feature Structure	Score	Toxic in training set
SCFP_6	1559190850	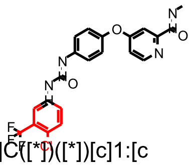 [*]C([*])([*])[c]1:[cH]:[*]:[cH]:[cH]:[c]:1Cl	0.441	3 out of 3

SCFP_6	-488587948	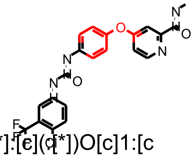<br>[*]:[c](c[*])O[c]1:[cH]:[cH]:[*]:[cH]:[cH]:1	0.381	2 out of 2
SCFP_6	-975241316	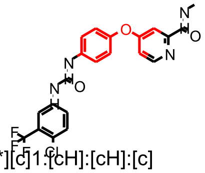<br>[*][c]1:[cH]:[cH]:[c](O[c](:[cH]:[*]):[cH]:[*]):[cH]:[cH]:1	0.381	2 out of 2

Top Features for negative contribution

Fingerprint	Bit/Smiles	Feature Structure	Score	Toxic in training set
SCFP_6	-1794974220	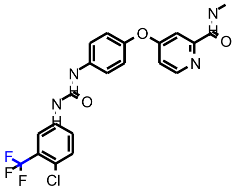<br>[*]C([*])([*])F	-0.55	2 out of 8
SCFP_6	-937094999	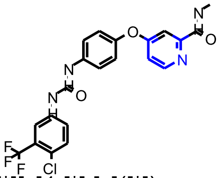<br>[*][c]1:[*]:[c]([*]):n:[cH]:[cH]:1	-0.358	3 out of 9
SCFP_6	-496201075	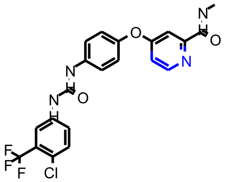<br>[*]:[cH]:[cH]:n:[*]	-0.289	8 out of 21

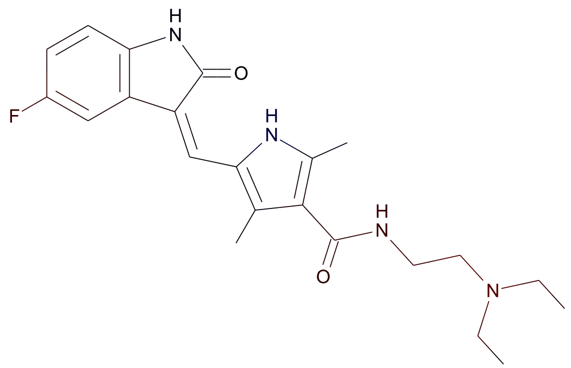

$C_{22}H_{27}FN_4O_2$

Molecular Weight: 398.47378

ALogP: 2.997

Rotatable Bonds: 7

Acceptors: 3

Donors: 3

Model Prediction

Prediction: Toxic

Probability: 0.635

Enrichment: 1.21

Bayesian Score: 2.26

Mahalanobis Distance: 13.3

Mahalanobis Distance p-value: 1.35e-007

Prediction: Positive if the Bayesian score is above the estimated best cutoff value from minimizing the false positive and false negative rate.
Probability: The esimated probability that the sample is in the positive category. This assumes that the Bayesian score follows a normal distribution and is different from the prediction using a cutoff.
Enrichment: An estimate of enrichment, that is, the increased likelihood (versus random) of this sample being in the category.
Bayesian Score: The standard Laplacian-modified Bayesian score.
Mahalanobis Distance: The Mahalanobis distance (MD) is the distance to the center of the training data. The larger the MD, the less trustworthy the prediction.
Mahalanobis Distance p-value: The p-value gives the fraction of training data with an MD greater than or equal to the one for the given sample, assuming normally distributed data. The smaller the p-value, the less trustworthy the prediciton. For highly non-normal X properties (e.g., fingerprints), the MD p-value is wildly inaccurate.

Structural Similar Compounds

Name	Domperidone	Clebopride Malate	Dobutamine .HCl (Free base form)
Structure	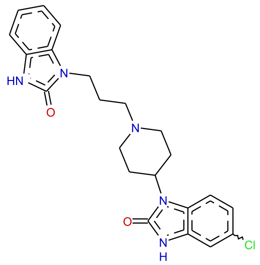	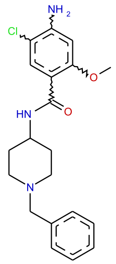	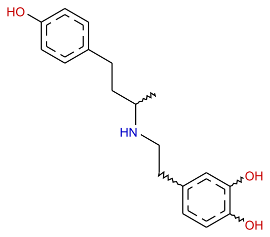
Actual Endpoint	Toxic	Non-Toxic	Toxic
Predicted Endpoint	Toxic	Non-Toxic	Toxic
Distance	0.619	0.627	0.678
Reference	Yakuri to Chiryo 8:4125-4136; 1980	Kiso to Rinsho 16:5649-5660; 1982	Yakuri to Chiryo 7:1707-1730; 1979

Model Applicability

Unknown features are fingerprint features in the query molecule, but not found or appearing too infrequently in the training set.

1. All properties and OPS components are within expected ranges.

Feature Contribution

Top features for positive contribution

Fingerprint	Bit/Smiles	Feature Structure	Score	Toxic in training set
SCFP_6	-1971137145	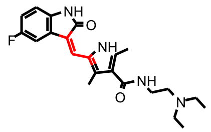 [*]C(=C[c](:[*]):[*])[*]	0.431	7 out of 8

SCFP_6	1725890097	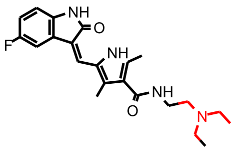<br>[*]CN(C[*])CC	0.381	2 out of 2
SCFP_6	-182283812	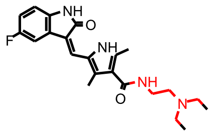<br>[*]CN(C[*])CCN[*]	0.381	2 out of 2

Top Features for negative contribution

Fingerprint	Bit/Smiles	Feature Structure	Score	Toxic in training set
SCFP_6	2109374332	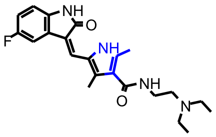<br>[*][c]1:[*]:[*]:[nH]:<br>[c]:1C	-0.446	3 out of 10
SCFP_6	1420330831	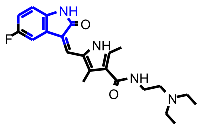<br>[*]=C1[*]=C[c]2:[cH]:<br>[*]:[cH]:[cH]:[c]:2N<br>1	-0.422	0 out of 1
SCFP_6	136686699	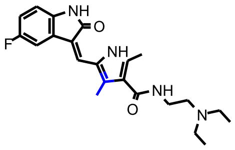<br>[*]:[c](:[*])C	-0.316	7 out of 19

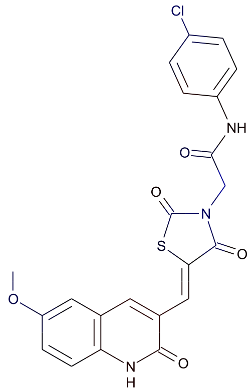

$C_{22}H_{16}ClN_3O_5S$

Molecular Weight: 469.89753

ALogP: 2.815

Rotatable Bonds: 5

Acceptors: 6

Donors: 2

Model Prediction

Prediction: Non-Carcinogen

Probability: 0.206

Enrichment: 0.642

Bayesian Score: -5.47

Mahalanobis Distance: 14

Mahalanobis Distance p-value: 1.65e-005

Prediction: Positive if the Bayesian score is above the estimated best cutoff value from minimizing the false positive and false negative rate.
Probability: The esimated probability that the sample is in the positive category. This assumes that the Bayesian score follows a normal distribution and is different from the prediction using a cutoff.
Enrichment: An estimate of enrichment, that is, the increased likelihood (versus random) of this sample being in the category.
Bayesian Score: The standard Laplacian-modified Bayesian score.
Mahalanobis Distance: The Mahalanobis distance (MD) is the distance to the center of the training data. The larger the MD, the less trustworthy the prediction.
Mahalanobis Distance p-value: The p-value gives the fraction of training data with an MD greater than or equal to the one for the given sample, assuming normally distributed data. The smaller the p-value, the less trustworthy the prediciton. For highly non-normal X properties (e.g., fingerprints), the MD p-value is wildly inaccurate.

Structural Similar Compounds

Name	Bicalutamide	Moricizine	Glipizide
Structure	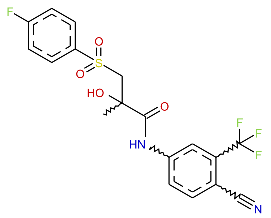	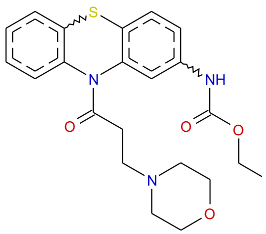	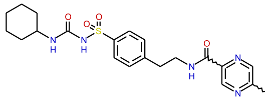
Actual Endpoint	Non-Carcinogen	Carcinogen	Non-Carcinogen
Predicted Endpoint	Non-Carcinogen	Carcinogen	Non-Carcinogen
Distance	0.579	0.638	0.679
Reference	US FDA (Centre for Drug Eval.& Res./Off. Testing & Res.) Sept. 1997	US FDA (Centre for Drug Eval.& Res./Off. Testing & Res.) Sept. 1997	US FDA (Centre for Drug Eval.& Res./Off. Testing & Res.) Sept. 1997

Model Applicability

Unknown features are fingerprint features in the query molecule, but not found or appearing too infrequently in the training set.

1. All properties and OPS components are within expected ranges.
2. Unknown ECFP_2 feature: 2131425032: [*]\C=C(\C=[*])/C(=[*])[*]
3. Unknown ECFP_2 feature: 1182722866: [*]C(=CC(=[*])[*])[*]
4. Unknown ECFP_2 feature: 1000552169: [*]\C=C\1/S[*][*]C1=[*]
5. Unknown ECFP_2 feature: 190445529: [*]N1[*][*]SC1=O

Feature Contribution

Top features for positive contribution

Fingerprint	Bit/Smiles	Feature Structure	Score	Carcinogen in training set
ECFP_6	738938915	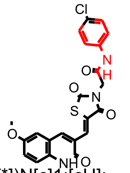 [*]C(=[*])N[c]1:[cH]:[cH]:[*]:[cH]:[cH]:1	0.617	2 out of 2

ECFP_6	464808839	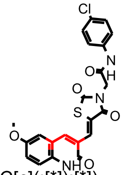 [*]C(=C[c](:[*]):[*]) [*]	0.524	8 out of 14
ECFP_6	-1925046727	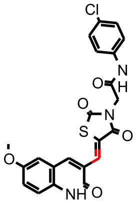 [*]C=[*]	0.391	11 out of 23
**Top Features for negative contribution**				
**Fingerprint**	**Bit/Smiles**	**Feature Structure**	**Score**	**Carcinogen in training set**
ECFP_6	-661097313	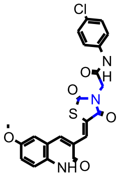 [*]CN1C(=[*])[*][*]C1 =[*]	-1.55	0 out of 12
ECFP_6	1731843802	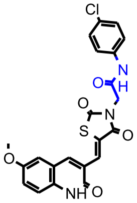 [*]CC(=O)N[*]	-0.657	0 out of 3
ECFP_6	912478223	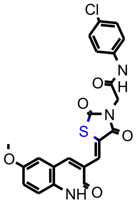 [*]S[*]	-0.638	1 out of 9

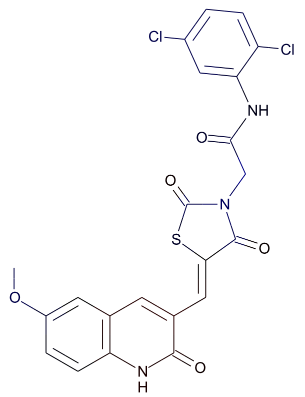

$C_{22}H_{15}Cl_2N_3O_5S$
Molecular Weight: 504.3426
ALogP: 3.479
Rotatable Bonds: 5
Acceptors: 6
Donors: 2

Structural Similar Compounds

Name	Bicalutamide	Fluticasone	Glimepride
Structure	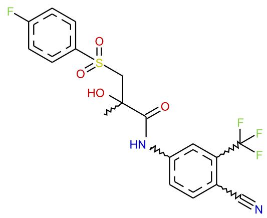	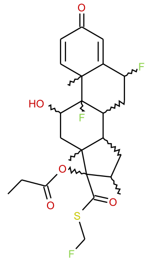	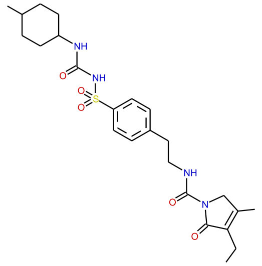
Actual Endpoint	Non-Carcinogen	Non-Carcinogen	Carcinogen
Predicted Endpoint	Non-Carcinogen	Non-Carcinogen	Carcinogen
Distance	0.634	0.657	0.671
Reference	US FDA (Centre for Drug Eval.& Res./Off. Testing & Res.) Sept. 1997	US FDA (Centre for Drug Eval.& Res./Off. Testing & Res.) Sept. 1997	US FDA (Centre for Drug Eval.& Res./Off. Testing & Res.) Sept. 1997

Model Prediction

Prediction: Non-Carcinogen
Probability: 0.205
Enrichment: 0.64
Bayesian Score: -6.8
Mahalanobis Distance: 13.6
Mahalanobis Distance p-value: 7.54e-005

Prediction: Positive if the Bayesian score is above the estimated best cutoff value from minimizing the false positive and false negative rate.
Probability: The esimated probability that the sample is in the positive category. This assumes that the Bayesian score follows a normal distribution and is different from the prediction using a cutoff.
Enrichment: An estimate of enrichment, that is, the increased likelihood (versus random) of this sample being in the category.
Bayesian Score: The standard Laplacian-modified Bayesian score.
Mahalanobis Distance: The Mahalanobis distance (MD) is the distance to the center of the training data. The larger the MD, the less trustworthy the prediction.
Mahalanobis Distance p-value: The p-value gives the fraction of training data with an MD greater than or equal to the one for the given sample, assuming normally distributed data. The smaller the p-value, the less trustworthy the prediciton. For highly non-normal X properties (e.g., fingerprints), the MD p-value is wildly inaccurate.

Model Applicability

Unknown features are fingerprint features in the query molecule, but not found or appearing too infrequently in the training set.

1. All properties and OPS components are within expected ranges.
2. Unknown ECFP_2 feature: 2131425032: [*]\C=C(\C=[*])/C(=[*])[*]
3. Unknown ECFP_2 feature: 1182722866: [*]C(=CC(=[*])[*])[*]
4. Unknown ECFP_2 feature: 1000552169: [*]\C=C\1/S[*][*]C1=[*]
5. Unknown ECFP_2 feature: 190445529: [*]N1[*][*]SC1=O

Feature Contribution

Top features for positive contribution				
**Fingerprint**	**Bit/Smiles**	**Feature Structure**	**Score**	**Carcinogen in training set**
ECFP_6	464808839	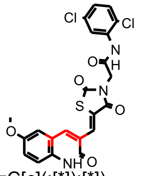 [*]C(=C[c](:[*]):[*]) [*]	0.524	8 out of 14

ECFP_6	-1925046727	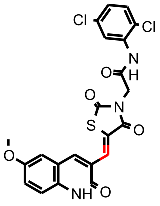 [*]C=[*]	0.391	11 out of 23
ECFP_6	-1699286547	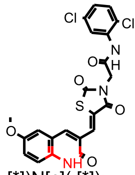 [*]C(=[*])N[c](:[*]):[*]	0.297	12 out of 28

Top Features for negative contribution

Fingerprint	Bit/Smiles	Feature Structure	Score	Carcinogen in training set
ECFP_6	-661097313	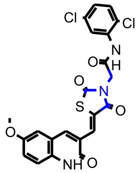 [*]CN1C(=[*])[*][*]C1=[*]	-1.55	0 out of 12
ECFP_6	1335691903	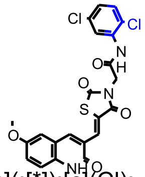 [*][c](:[*]):[c](Cl):[cH]:[*]	-0.669	3 out of 22
ECFP_6	1731843802	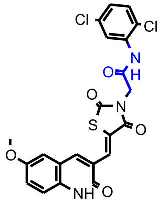 [*]CC(=O)N[*]	-0.657	0 out of 3

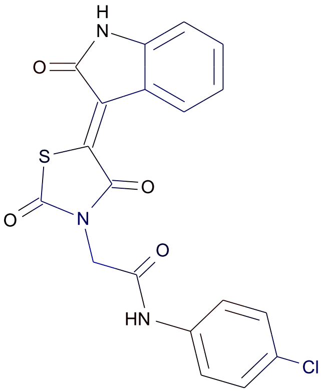

$C_{19}H_{12}ClN_3O_4S$
Molecular Weight: 413.83428
ALogP: 2.363
Rotatable Bonds: 3
Acceptors: 5
Donors: 2

Model Prediction

Prediction: Non-Carcinogen
Probability: 0.205
Enrichment: 0.639
Bayesian Score: -6.61
Mahalanobis Distance: 12.7
Mahalanobis Distance p-value: 0.00132

Prediction: Positive if the Bayesian score is above the estimated best cutoff value from minimizing the false positive and false negative rate.
Probability: The esimated probability that the sample is in the positive category. This assumes that the Bayesian score follows a normal distribution and is different from the prediction using a cutoff.
Enrichment: An estimate of enrichment, that is, the increased likelihood (versus random) of this sample being in the category.
Bayesian Score: The standard Laplacian-modified Bayesian score.
Mahalanobis Distance: The Mahalanobis distance (MD) is the distance to the center of the training data. The larger the MD, the less trustworthy the prediction.
Mahalanobis Distance p-value: The p-value gives the fraction of training data with an MD greater than or equal to the one for the given sample, assuming normally distributed data. The smaller the p-value, the less trustworthy the prediciton. For highly non-normal X properties (e.g., fingerprints), the MD p-value is wildly inaccurate.

Structural Similar Compounds

Name	Indapamide	Metolazone	Acetohexamide
Structure	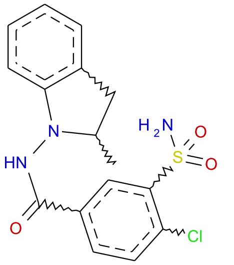	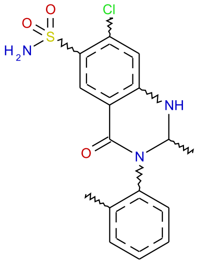	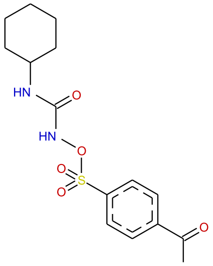
Actual Endpoint	Non-Carcinogen	Non-Carcinogen	Non-Carcinogen
Predicted Endpoint	Non-Carcinogen	Non-Carcinogen	Non-Carcinogen
Distance	0.578	0.580	0.647
Reference	US FDA (Centre for Drug Eval.& Res./Off. Testing & Res.) Sept. 1997	US FDA (Centre for Drug Eval.& Res./Off. Testing & Res.) Sept. 1997	US FDA (Centre for Drug Eval.& Res./Off. Testing & Res.) Sept. 1997

Model Applicability

Unknown features are fingerprint features in the query molecule, but not found or appearing too infrequently in the training set.

1. All properties and OPS components are within expected ranges.
2. Unknown ECFP_2 feature: -631778390: [*]C(=C1S[*][*]C1=[*])[*]
3. Unknown ECFP_2 feature: 190445529: [*]N1[*][*]SC1=O

Feature Contribution

Top features for positive contribution

Fingerprint	Bit/Smiles	Feature Structure	Score	Carcinogen in training set
ECFP_6	738938915	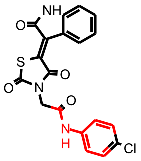 [*]C(=[*])N[c]1:[cH]:[cH]:[*]:[cH]:[cH]:1	0.617	2 out of 2

ECFP_6	-1699286547	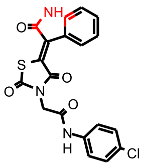 [*]C(=[*])N[c](:[*]):[*]	0.297	12 out of 28
ECFP_6	1298725959	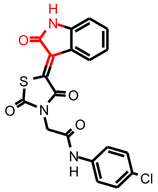 [*]NC(=O)C(=[*])[*]	0.279	4 out of 9

Top Features for negative contribution

Fingerprint	Bit/Smiles	Feature Structure	Score	Carcinogen in training set
ECFP_6	-661097313	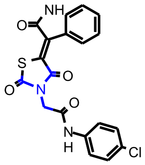 [*]CN1C(=[*])[*][*]C1=[*]	-1.55	0 out of 12
ECFP_6	1731843802	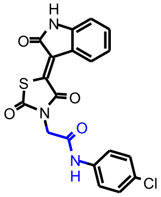 [*]CC(=O)N[*]	-0.657	0 out of 3
ECFP_6	912478223	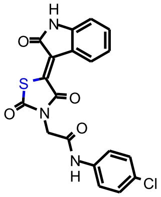 [*]S[*]	-0.638	1 out of 9

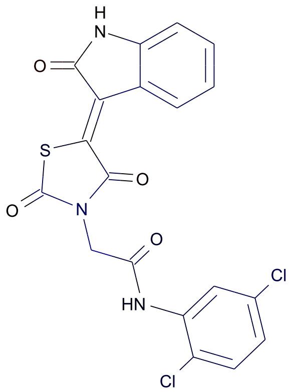

$C_{19}H_{11}Cl_2N_3O_4S$
Molecular Weight: 448.27933
ALogP: 3.028
Rotatable Bonds: 3
Acceptors: 5
Donors: 2

Structural Similar Compounds

Name	Indapamide	Metolazone	Bicalutamide
Structure	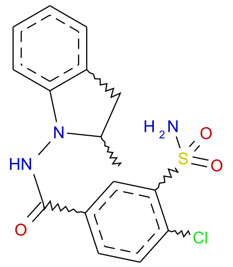	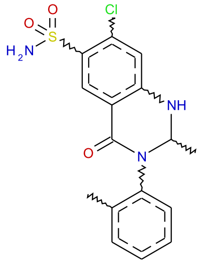	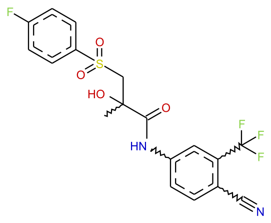
Actual Endpoint	Non-Carcinogen	Non-Carcinogen	Non-Carcinogen
Predicted Endpoint	Non-Carcinogen	Non-Carcinogen	Non-Carcinogen
Distance	0.609	0.619	0.652
Reference	US FDA (Centre for Drug Eval.& Res./Off. Testing & Res.) Sept. 1997	US FDA (Centre for Drug Eval.& Res./Off. Testing & Res.) Sept. 1997	US FDA (Centre for Drug Eval.& Res./Off. Testing & Res.) Sept. 1997

Model Prediction

Prediction: Non-Carcinogen
Probability: 0.207
Enrichment: 0.646
Bayesian Score: -7.61
Mahalanobis Distance: 11.3
Mahalanobis Distance p-value: 0.0557

Prediction: Positive if the Bayesian score is above the estimated best cutoff value from minimizing the false positive and false negative rate.
Probability: The esimated probability that the sample is in the positive category. This assumes that the Bayesian score follows a normal distribution and is different from the prediction using a cutoff.
Enrichment: An estimate of enrichment, that is, the increased likelihood (versus random) of this sample being in the category.
Bayesian Score: The standard Laplacian-modified Bayesian score.
Mahalanobis Distance: The Mahalanobis distance (MD) is the distance to the center of the training data. The larger the MD, the less trustworthy the prediction.
Mahalanobis Distance p-value: The p-value gives the fraction of training data with an MD greater than or equal to the one for the given sample, assuming normally distributed data. The smaller the p-value, the less trustworthy the prediciton. For highly non-normal X properties (e.g., fingerprints), the MD p-value is wildly inaccurate.

Model Applicability

Unknown features are fingerprint features in the query molecule, but not found or appearing too infrequently in the training set.

1. All properties and OPS components are within expected ranges.
2. Unknown ECFP_2 feature: -631778390: [*]C(=C1S[*][*]C1=[*])[*]
3. Unknown ECFP_2 feature: 190445529: [*]N1[*][*]SC1=O

Feature Contribution

Top features for positive contribution

Fingerprint	Bit/Smiles	Feature Structure	Score	Carcinogen in training set
ECFP_6	-1699286547	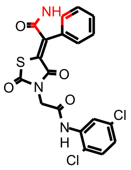 [*]C(=[*])N[c](:[*]):[*]	0.297	12 out of 28

ECFP_6	1298725959	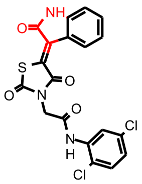 [*]NC(=O)C(=[*])[*]	0.279	4 out of 9
ECFP_6	2106656448	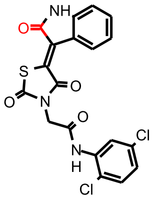 [*]C(=O)[*]	0.254	31 out of 77

Top Features for negative contribution

**Fingerprint**	**Bit/Smiles**	**Feature Structure**	**Score**	**Carcinogen in training set**
ECFP_6	-661097313	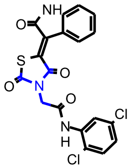 [*]CN1C(=[*])[*][*]C1 =[*]	-1.55	0 out of 12
ECFP_6	1335691903	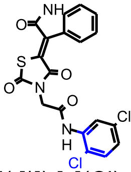 [*][c](:[*]):[c](Cl): [cH]:[*]	-0.669	3 out of 22
ECFP_6	1731843802	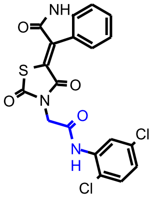 [*]CC(=O)N[*]	-0.657	0 out of 3

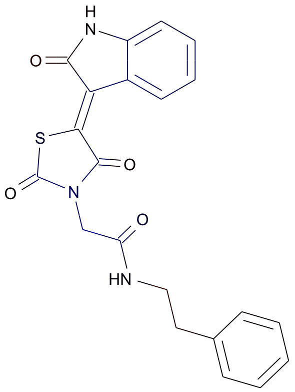

$C_{21}H_{17}N_3O_4S$
Molecular Weight: 407.44237
ALogP: 2.027
Rotatable Bonds: 5
Acceptors: 5
Donors: 2

Model Prediction

Prediction: Non-Carcinogen
Probability: 0.207
Enrichment: 0.646
Bayesian Score: -5.01
Mahalanobis Distance: 12
Mahalanobis Distance p-value: 0.0122

Prediction: Positive if the Bayesian score is above the estimated best cutoff value from minimizing the false positive and false negative rate.
Probability: The esimated probability that the sample is in the positive category. This assumes that the Bayesian score follows a normal distribution and is different from the prediction using a cutoff.
Enrichment: An estimate of enrichment, that is, the increased likelihood (versus random) of this sample being in the category.
Bayesian Score: The standard Laplacian-modified Bayesian score.
Mahalanobis Distance: The Mahalanobis distance (MD) is the distance to the center of the training data. The larger the MD, the less trustworthy the prediction.
Mahalanobis Distance p-value: The p-value gives the fraction of training data with an MD greater than or equal to the one for the given sample, assuming normally distributed data. The smaller the p-value, the less trustworthy the prediciton. For highly non-normal X properties (e.g., fingerprints), the MD p-value is wildly inaccurate.

Structural Similar Compounds

Name	Bicalutamide	Acetohexamide	Penicillin
Structure	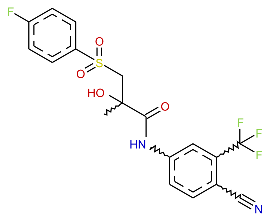	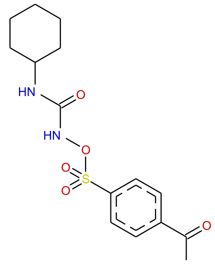	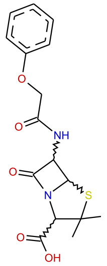
Actual Endpoint	Non-Carcinogen	Non-Carcinogen	Non-Carcinogen
Predicted Endpoint	Non-Carcinogen	Non-Carcinogen	Non-Carcinogen
Distance	0.587	0.589	0.604
Reference	US FDA (Centre for Drug Eval.& Res./Off. Testing & Res.) Sept. 1997	US FDA (Centre for Drug Eval.& Res./Off. Testing & Res.) Sept. 1997	US FDA (Centre for Drug Eval.& Res./Off. Testing & Res.) Sept. 1997

Model Applicability

Unknown features are fingerprint features in the query molecule, but not found or appearing too infrequently in the training set.

1. All properties and OPS components are within expected ranges.
2. Unknown ECFP_2 feature: -631778390: [*]C(=C1S[*][*]C1=[*])[*]
3. Unknown ECFP_2 feature: 190445529: [*]N1[*][*]SC1=O

Feature Contribution

Top features for positive contribution

Fingerprint	Bit/Smiles	Feature Structure	Score	Carcinogen in training set
ECFP_6	-1699286547	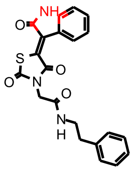 [*]C(=[*])N[c](:[*]):[*]	0.297	12 out of 28

ECFP_6	-1791034651	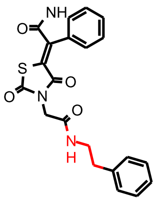 [*]CCN[*]	0.296	7 out of 16
ECFP_6	1298725959	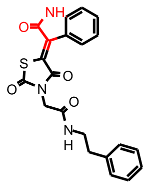 [*]NC(=O)C(=[*])[*]	0.279	4 out of 9

Top Features for negative contribution

Fingerprint	Bit/Smiles	Feature Structure	Score	Carcinogen in training set
ECFP_6	-661097313	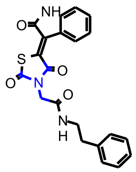 [*]CN1C(=[*])[*][*]C1=[*]	-1.55	0 out of 12
ECFP_6	1731843802	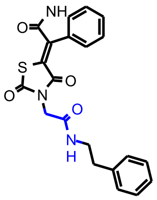 [*]CC(=O)N[*]	-0.657	0 out of 3
ECFP_6	912478223	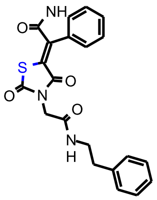 [*]S[*]	-0.638	1 out of 9

Sorafenib

TOPKAT_Mouse_Female_FDA_None_vs_Carcinogen

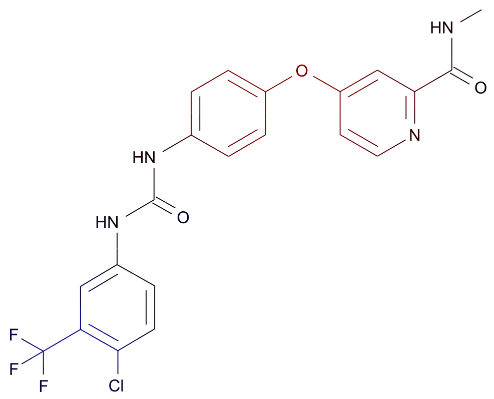

$C_{21}H_{16}ClF_3N_4O_3$
Molecular Weight: 464.82494
ALogP: 4.175
Rotatable Bonds: 6
Acceptors: 4
Donors: 3

Model Prediction

Prediction: Carcinogen
Probability: 0.257
Enrichment: 0.801
Bayesian Score: -0.321
Mahalanobis Distance: 14.9
Mahalanobis Distance p-value: 4.21e-007

Prediction: Positive if the Bayesian score is above the estimated best cutoff value from minimizing the false positive and false negative rate.
Probability: The esimated probability that the sample is in the positive category. This assumes that the Bayesian score follows a normal distribution and is different from the prediction using a cutoff.
Enrichment: An estimate of enrichment, that is, the increased likelihood (versus random) of this sample being in the category.
Bayesian Score: The standard Laplacian-modified Bayesian score.
Mahalanobis Distance: The Mahalanobis distance (MD) is the distance to the center of the training data. The larger the MD, the less trustworthy the prediction.
Mahalanobis Distance p-value: The p-value gives the fraction of training data with an MD greater than or equal to the one for the given sample, assuming normally distributed data. The smaller the p-value, the less trustworthy the prediciton. For highly non-normal X properties (e.g., fingerprints), the MD p-value is wildly inaccurate.

Structural Similar Compounds

Name	Glimepride	Glyburide	Fluvastatin
Structure	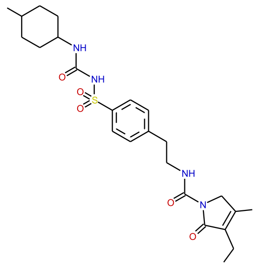	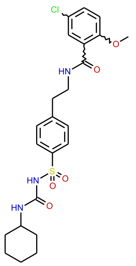	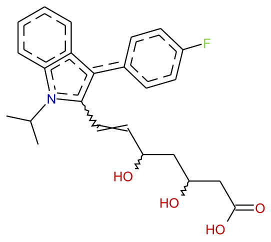
Actual Endpoint	Carcinogen	Non-Carcinogen	Non-Carcinogen
Predicted Endpoint	Carcinogen	Non-Carcinogen	Non-Carcinogen
Distance	0.605	0.615	0.625
Reference	US FDA (Centre for Drug Eval.& Res./Off. Testing & Res.) Sept. 1997	US FDA (Centre for Drug Eval.& Res./Off. Testing & Res.) Sept. 1997	US FDA (Centre for Drug Eval.& Res./Off. Testing & Res.) Sept. 1997

Model Applicability

Unknown features are fingerprint features in the query molecule, but not found or appearing too infrequently in the training set.

1. OPS PC20 out of range. Value: -3.3309. Training min, max, SD, explained variance: -3.1862, 4.4571, 1.28, 0.0167.

Feature Contribution

Top features for positive contribution

Fingerprint	Bit/Smiles	Feature Structure	Score	Carcinogen in training set
ECFP_6	738938915	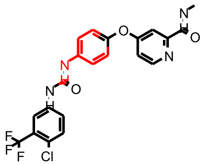 [*]C(=[*])N[c]1:[cH]:[cH]:[*]:[cH]:[cH]:1	0.617	2 out of 2

ECFP_6	1338334141	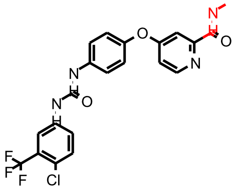 [*]C(=[*])NC	0.442	2 out of 3
ECFP_6	-834094296	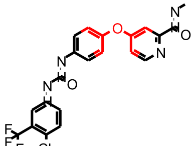 [*]:[cH]:[c](O[c](:[cH]:[*]):[cH]:[*]):[cH]:[*]	0.424	1 out of 1

Top Features for negative contribution

**Fingerprint**	**Bit/Smiles**	**Feature Structure**	**Score**	**Carcinogen in training set**
ECFP_6	1335691903	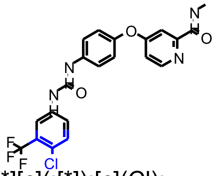 [*][c](:[*]):[c](Cl):[cH]:[*]	-0.669	3 out of 22
ECFP_6	1336678434	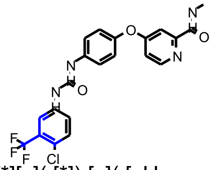 [*][c](:[*]):[c](:[cH]:[*])C([*])([*])[*]	-0.657	0 out of 3
ECFP_6	-1952889961	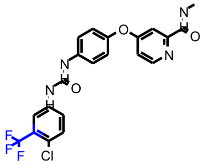 [*]:[c](:[*])C(F)(F)F	-0.657	0 out of 3

Sunitinib

TOPKAT_Mouse_Female_FDA_None_vs_Carcinogen

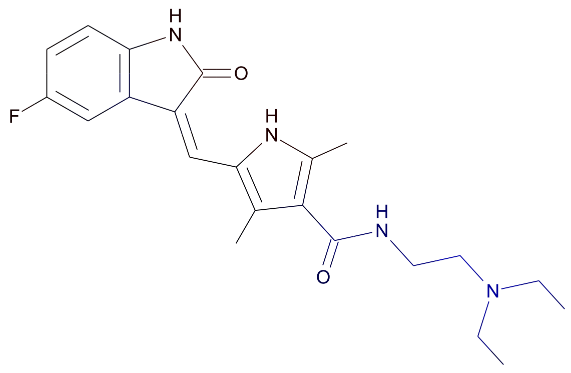

$C_{22}H_{27}FN_4O_2$
Molecular Weight: 398.47378
ALogP: 2.997
Rotatable Bonds: 7
Acceptors: 3
Donors: 3

Model Prediction

Prediction: Non-Carcinogen
Probability: 0.218
Enrichment: 0.679
Bayesian Score: -3.25
Mahalanobis Distance: 13.8
Mahalanobis Distance p-value: 3.24e-005

Prediction: Positive if the Bayesian score is above the estimated best cutoff value from minimizing the false positive and false negative rate.
Probability: The esimated probability that the sample is in the positive category. This assumes that the Bayesian score follows a normal distribution and is different from the prediction using a cutoff.
Enrichment: An estimate of enrichment, that is, the increased likelihood (versus random) of this sample being in the category.
Bayesian Score: The standard Laplacian-modified Bayesian score.
Mahalanobis Distance: The Mahalanobis distance (MD) is the distance to the center of the training data. The larger the MD, the less trustworthy the prediction.
Mahalanobis Distance p-value: The p-value gives the fraction of training data with an MD greater than or equal to the one for the given sample, assuming normally distributed data. The smaller the p-value, the less trustworthy the prediciton. For highly non-normal X properties (e.g., fingerprints), the MD p-value is wildly inaccurate.

Structural Similar Compounds

Name	Fluvastatin	Metoclopramide	Flecainide
Structure	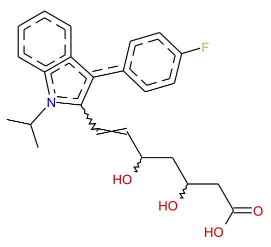	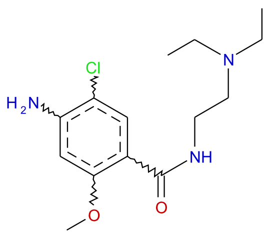	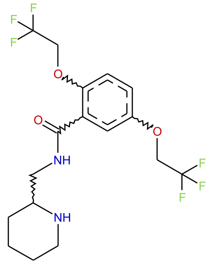
Actual Endpoint	Non-Carcinogen	Non-Carcinogen	Non-Carcinogen
Predicted Endpoint	Non-Carcinogen	Non-Carcinogen	Non-Carcinogen
Distance	0.610	0.643	0.666
Reference	US FDA (Centre for Drug Eval.& Res./Off. Testing & Res.) Sept. 1997	US FDA (Centre for Drug Eval.& Res./Off. Testing & Res.) Sept. 1997	US FDA (Centre for Drug Eval.& Res./Off. Testing & Res.) Sept. 1997

Model Applicability

Unknown features are fingerprint features in the query molecule, but not found or appearing too infrequently in the training set.

1. All properties and OPS components are within expected ranges.
2. Unknown ECFP_2 feature: -1658273810: [*]C(=[*])[c]1:[c]([*]):[*]:[*]:[c]:1[*]
3. Unknown ECFP_2 feature: 980271847: [*][c]1:[*]:[*]:[nH]:[c]:1C=[*]
4. Unknown ECFP_2 feature: 1182722866: [*]C(=CC(=[*])[*])[*]

Feature Contribution

Top features for positive contribution

Fingerprint	Bit/Smiles	Feature Structure	Score	Carcinogen in training set
ECFP_6	1791989338	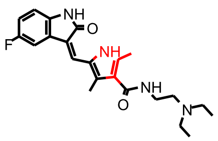 [*][c]1:[*]:[*]:[nH]:[c]:1C	0.424	1 out of 1

ECFP_6	-1925046727	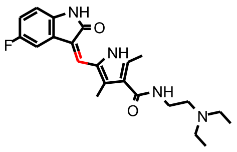 [*]C=[*]	0.391	11 out of 23
ECFP_6	-1699286547	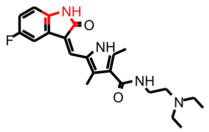 [*]C(=[*])N[c](:[*]):[*]	0.297	12 out of 28

Top Features for negative contribution

Fingerprint	Bit/Smiles	Feature Structure	Score	Carcinogen in training set
ECFP_6	1795893449	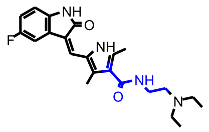 [*]CCNC(=O)[c](:[*]):[*]	-0.657	0 out of 3
ECFP_6	-628327667	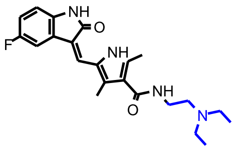 [*]CCN(CC)CC	-0.657	0 out of 3
ECFP_6	-659402940	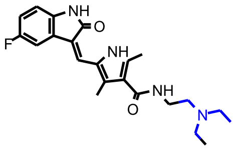 [*]CN(C[*])CC	-0.657	0 out of 3

Sorafenib

TOPKAT_Mouse_Female_FDA_Single_vs_Multiple

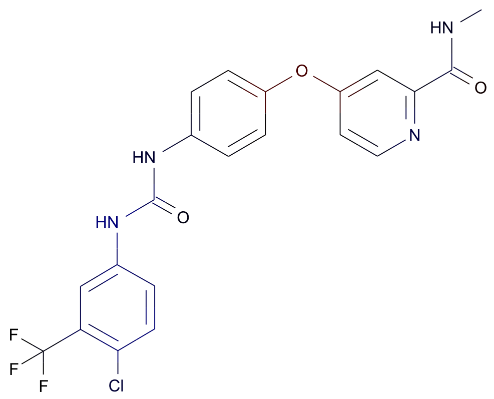

$C_{21}H_{16}ClF_3N_4O_3$
Molecular Weight: 464.82494
ALogP: 4.175
Rotatable Bonds: 6
Acceptors: 4
Donors: 3

Model Prediction

Prediction: Single-Carcinogen
Probability: 0.283
Enrichment: 0.691
Bayesian Score: -3.89
Mahalanobis Distance: 11.1
Mahalanobis Distance p-value: 0.00221

Prediction: Positive if the Bayesian score is above the estimated best cutoff value from minimizing the false positive and false negative rate.
Probability: The esimated probability that the sample is in the positive category. This assumes that the Bayesian score follows a normal distribution and is different from the prediction using a cutoff.
Enrichment: An estimate of enrichment, that is, the increased likelihood (versus random) of this sample being in the category.
Bayesian Score: The standard Laplacian-modified Bayesian score.
Mahalanobis Distance: The Mahalanobis distance (MD) is the distance to the center of the training data. The larger the MD, the less trustworthy the prediction.
Mahalanobis Distance p-value: The p-value gives the fraction of training data with an MD greater than or equal to the one for the given sample, assuming normally distributed data. The smaller the p-value, the less trustworthy the prediciton. For highly non-normal X properties (e.g., fingerprints), the MD p-value is wildly inaccurate.

Structural Similar Compounds

Name	Glimepride	Labetalol	Lansoprazole
Structure	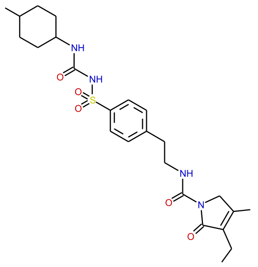	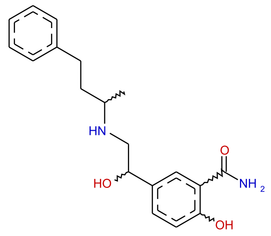	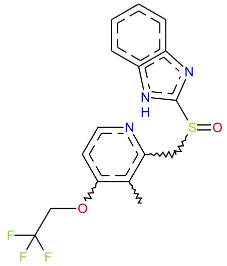
Actual Endpoint	Single-Carcinogen	Single-Carcinogen	Single-Carcinogen
Predicted Endpoint	Single-Carcinogen	Single-Carcinogen	Single-Carcinogen
Distance	0.599	0.808	0.820
Reference	US FDA (Centre for Drug Eval.& Res./Off. Testing & Res.) Sept. 1997	US FDA (Centre for Drug Eval.& Res./Off. Testing & Res.) Sept. 1997	US FDA (Centre for Drug Eval.& Res./Off. Testing & Res.) Sept. 1997

Model Applicability

Unknown features are fingerprint features in the query molecule, but not found or appearing too infrequently in the training set.

1. All properties and OPS components are within expected ranges.
2. Unknown ECFP_2 feature: 1336678434: [*][c](:[*]):[c](C([*])([*])[*]):c:[*]
3. Unknown ECFP_2 feature: -1952889961: [*]:[c](:[*])C(F)(F)F

Feature Contribution

Top features for positive contribution

Fingerprint	Bit/Smiles	Feature Structure	Score	Multiple-Carcinogen in training set
ECFP_4	-834094296	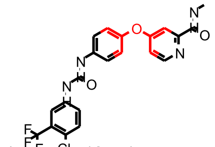 [*]:[cH]:[c](O[c](:[cH]:[*]):[cH]:[*]):[cH]:[*]	0.351	1 out of 1

ECFP_4	1407472008	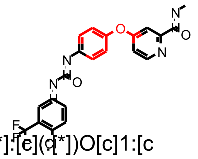 [*]:[c](:[*])O[c]1:[cH]:[cH]:[*]:[cH]:[cH]:1	0.351	1 out of 1
ECFP_4	143734695	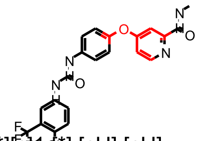 [*][c]1:[*]:[cH]:[cH]:[c](O[c](:[*]):[*]):[cH]:1	0.351	1 out of 1

Top Features for negative contribution

Fingerprint	Bit/Smiles	Feature Structure	Score	Multiple-Carcinogen in training set
ECFP_4	888054369	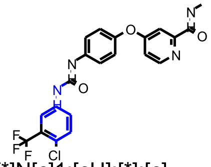 [*]N[c]1:[cH]:[*]:[c]([*]):[cH]:[cH]:1	-0.8	0 out of 3
ECFP_4	1335691903	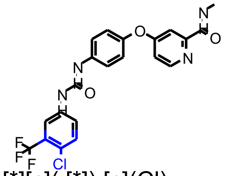 [*][c](:[*]):[c](Cl):[cH]:[*]	-0.8	0 out of 3
ECFP_4	1338334141	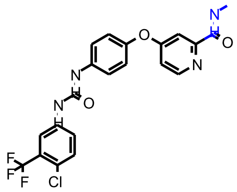 [*]C(=[*])NC	-0.597	0 out of 2

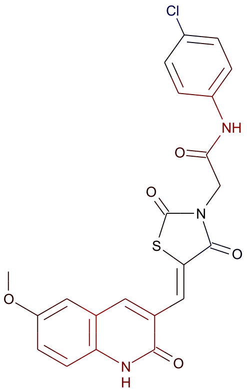

$C_{22}H_{16}ClN_3O_5S$
Molecular Weight: 469.89753
ALogP: 2.815
Rotatable Bonds: 5
Acceptors: 6
Donors: 2

Structural Similar Compounds

Name	Bicalutamide	Moricizine	Glipizide
Structure	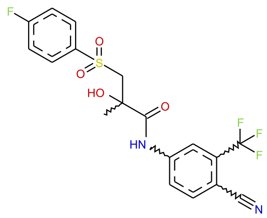	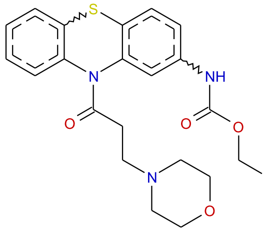	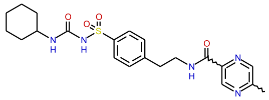
Actual Endpoint	Carcinogen	Non-Carcinogen	Non-Carcinogen
Predicted Endpoint	Carcinogen	Non-Carcinogen	Non-Carcinogen
Distance	0.536	0.632	0.681
Reference	US FDA (Centre for Drug Eval.& Res./Off. Testing & Res.) Sept. 1997	US FDA (Centre for Drug Eval.& Res./Off. Testing & Res.) Sept. 1997	US FDA (Centre for Drug Eval.& Res./Off. Testing & Res.) Sept. 1997

Model Prediction

Prediction: Carcinogen
Probability: 0.399
Enrichment: 1.36
Bayesian Score: 3
Mahalanobis Distance: 13.8
Mahalanobis Distance p-value: 1.72e-005

Prediction: Positive if the Bayesian score is above the estimated best cutoff value from minimizing the false positive and false negative rate.
Probability: The esimated probability that the sample is in the positive category. This assumes that the Bayesian score follows a normal distribution and is different from the prediction using a cutoff.
Enrichment: An estimate of enrichment, that is, the increased likelihood (versus random) of this sample being in the category.
Bayesian Score: The standard Laplacian-modified Bayesian score.
Mahalanobis Distance: The Mahalanobis distance (MD) is the distance to the center of the training data. The larger the MD, the less trustworthy the prediction.
Mahalanobis Distance p-value: The p-value gives the fraction of training data with an MD greater than or equal to the one for the given sample, assuming normally distributed data. The smaller the p-value, the less trustworthy the prediciton. For highly non-normal X properties (e.g., fingerprints), the MD p-value is wildly inaccurate.

Model Applicability

Unknown features are fingerprint features in the query molecule, but not found or appearing too infrequently in the training set.

1. All properties and OPS components are within expected ranges.

Feature Contribution

Top features for positive contribution

Fingerprint	Bit/Smiles	Feature Structure	Score	Carcinogen in training set
FCFP_6	-1757681964	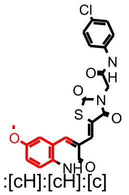 [*][c]1:[cH]:[cH]:[c](OC):[cH]:[c]:1C=[*]	0.676	2 out of 2

FCFP_6	-451043714	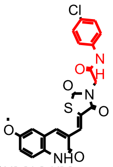 [*]CC(=O)N[c]1:[cH]:[cH]:[c]([*]):[cH]:[cH]:1	0.676	2 out of 2
FCFP_6	1175665944	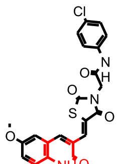 [*]C1=[*][c](:[*]):[c](NC1=O):[cH]:[*]	0.655	7 out of 12
**Top Features for negative contribution**				
**Fingerprint**	**Bit/Smiles**	**Feature Structure**	**Score**	**Carcinogen in training set**
FCFP_6	-1553874037	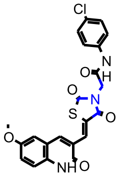 [*]CN1C(=[*])[*][*]C1=[*]	-0.45	5 out of 32
FCFP_6	551850122	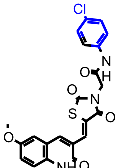 [*][c]1:[*]:[cH]:[c](Cl):[cH]:[cH]:1	-0.433	8 out of 49
FCFP_6	71476542	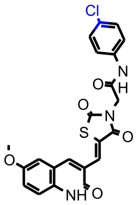 [*]:[c](:[*])Cl	-0.406	10 out of 59

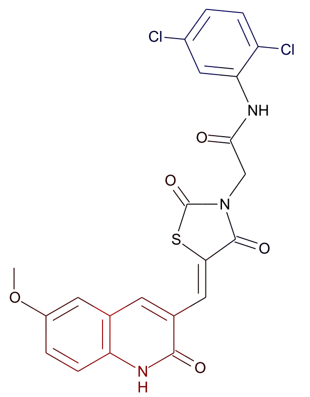

$C_{22}H_{15}Cl_2N_3O_5S$
Molecular Weight: 504.3426
ALogP: 3.479
Rotatable Bonds: 5
Acceptors: 6
Donors: 2

Model Prediction

Prediction: Carcinogen
Probability: 0.332
Enrichment: 1.13
Bayesian Score: 0.921
Mahalanobis Distance: 13.8
Mahalanobis Distance p-value: 1.31e-005

Prediction: Positive if the Bayesian score is above the estimated best cutoff value from minimizing the false positive and false negative rate.
Probability: The esimated probability that the sample is in the positive category. This assumes that the Bayesian score follows a normal distribution and is different from the prediction using a cutoff.
Enrichment: An estimate of enrichment, that is, the increased likelihood (versus random) of this sample being in the category.
Bayesian Score: The standard Laplacian-modified Bayesian score.
Mahalanobis Distance: The Mahalanobis distance (MD) is the distance to the center of the training data. The larger the MD, the less trustworthy the prediction.
Mahalanobis Distance p-value: The p-value gives the fraction of training data with an MD greater than or equal to the one for the given sample, assuming normally distributed data. The smaller the p-value, the less trustworthy the prediciton. For highly non-normal X properties (e.g., fingerprints), the MD p-value is wildly inaccurate.

Structural Similar Compounds

Name	Bicalutamide	Fluticasone	Moricizine
Structure	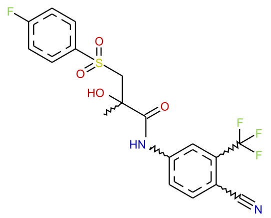	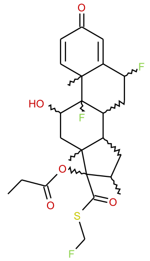	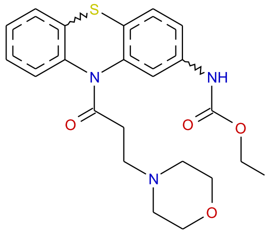
Actual Endpoint	Carcinogen	Non-Carcinogen	Non-Carcinogen
Predicted Endpoint	Carcinogen	Carcinogen	Non-Carcinogen
Distance	0.595	0.653	0.663
Reference	US FDA (Centre for Drug Eval.& Res./Off. Testing & Res.) Sept. 1997	US FDA (Centre for Drug Eval.& Res./Off. Testing & Res.) Sept. 1997	US FDA (Centre for Drug Eval.& Res./Off. Testing & Res.) Sept. 1997

Model Applicability

Unknown features are fingerprint features in the query molecule, but not found or appearing too infrequently in the training set.

1. All properties and OPS components are within expected ranges.

Feature Contribution

Top features for positive contribution

Fingerprint	Bit/Smiles	Feature Structure	Score	Carcinogen in training set
FCFP_6	-1757681964	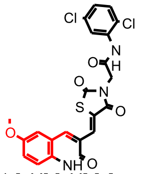 [*][c]1:[cH]:[cH]:[c](OC):[cH]:[c]:1C=[*]	0.676	2 out of 2

FCFP_6	1175665944	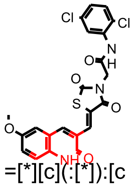 [*]C1=[*][c](:[*]):[c](NC1=O):[cH]:[*]	0.655	7 out of 12
FCFP_6	451847724	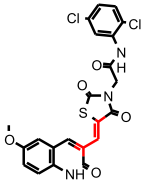 [*]C(=CC(=[*])[*])[*]	0.479	21 out of 48
**Top Features for negative contribution**				
**Fingerprint**	**Bit/Smiles**	**Feature Structure**	**Score**	**Carcinogen in training set**
FCFP_6	1783756416	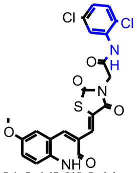 [*]N[c]1:[cH]:[*]:[cH]:[cH]:[c]:1Cl	-0.719	0 out of 4
FCFP_6	-1553874037	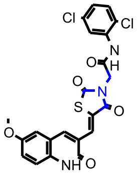 [*]CN1C(=[*])[*][*]C1=[*]	-0.45	5 out of 32
FCFP_6	551850122	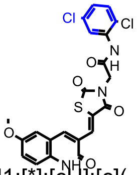 [*][c]1:[*]:[cH]:[c](Cl):[cH]:[cH]:1	-0.433	8 out of 49

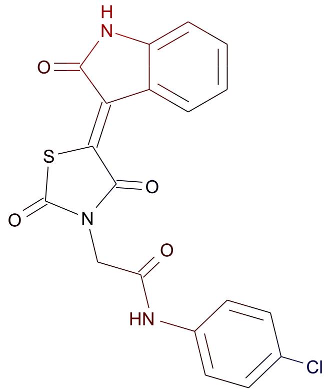

$C_{19}H_{12}ClN_3O_4S$
Molecular Weight: 413.83428
ALogP: 2.363
Rotatable Bonds: 3
Acceptors: 5
Donors: 2

Model Prediction

Prediction: Carcinogen
Probability: 0.364
Enrichment: 1.24
Bayesian Score: 1.95
Mahalanobis Distance: 10.6
Mahalanobis Distance p-value: 0.162

Prediction: Positive if the Bayesian score is above the estimated best cutoff value from minimizing the false positive and false negative rate.
Probability: The esimated probability that the sample is in the positive category. This assumes that the Bayesian score follows a normal distribution and is different from the prediction using a cutoff.
Enrichment: An estimate of enrichment, that is, the increased likelihood (versus random) of this sample being in the category.
Bayesian Score: The standard Laplacian-modified Bayesian score.
Mahalanobis Distance: The Mahalanobis distance (MD) is the distance to the center of the training data. The larger the MD, the less trustworthy the prediction.
Mahalanobis Distance p-value: The p-value gives the fraction of training data with an MD greater than or equal to the one for the given sample, assuming normally distributed data. The smaller the p-value, the less trustworthy the prediciton. For highly non-normal X properties (e.g., fingerprints), the MD p-value is wildly inaccurate.

Structural Similar Compounds

Name	Indapamide	Metolazone	Bicalutamide
Structure	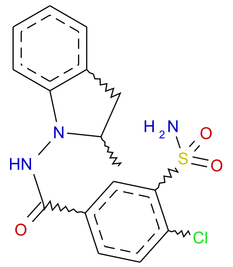	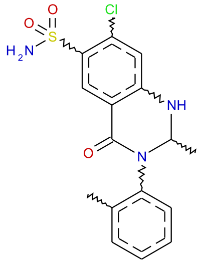	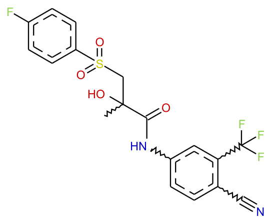
Actual Endpoint	Non-Carcinogen	Non-Carcinogen	Carcinogen
Predicted Endpoint	Non-Carcinogen	Non-Carcinogen	Carcinogen
Distance	0.554	0.555	0.594
Reference	US FDA (Centre for Drug Eval.& Res./Off. Testing & Res.) Sept. 1997	US FDA (Centre for Drug Eval.& Res./Off. Testing & Res.) Sept. 1997	US FDA (Centre for Drug Eval.& Res./Off. Testing & Res.) Sept. 1997

Model Applicability

Unknown features are fingerprint features in the query molecule, but not found or appearing too infrequently in the training set.

1. All properties and OPS components are within expected ranges.

Feature Contribution

Top features for positive contribution

Fingerprint	Bit/Smiles	Feature Structure	Score	Carcinogen in training set
FCFP_6	-451043714	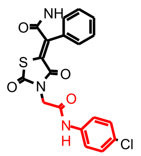 [*]CC(=O)N[c]1:[cH]:[cH]:[c]([*]):[cH]:[cH]:1	0.676	2 out of 2

FCFP_6	1175665944	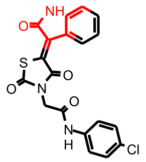 [*]C1=[*][c](:[*]):[c](NC1=O):[cH]:[*]	0.655	7 out of 12
FCFP_6	-1838187238	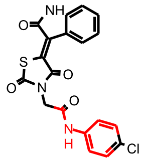 [*]C(=[*])N[c]1:[cH]:[cH]:[*]:[cH]:[cH]:1	0.565	4 out of 7
**Top Features for negative contribution**				
**Fingerprint**	**Bit/Smiles**	**Feature Structure**	**Score**	**Carcinogen in training set**
FCFP_6	-1553874037	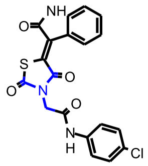 [*]CN1C(=[*])[*][*]C1=[*]	-0.45	5 out of 32
FCFP_6	551850122	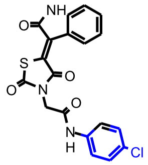 [*][c]1:[*]:[cH]:[c](Cl):[cH]:[cH]:1	-0.433	8 out of 49
FCFP_6	71476542	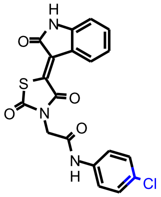 [*]:[c](:[*])Cl	-0.406	10 out of 59

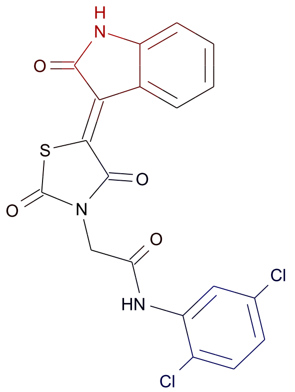

$C_{19}H_{11}Cl_2N_3O_4S$
Molecular Weight: 448.27933
ALogP: 3.028
Rotatable Bonds: 3
Acceptors: 5
Donors: 2

Model Prediction

Prediction: Carcinogen
Probability: 0.299
Enrichment: 1.02
Bayesian Score: -0.241
Mahalanobis Distance: 10.8
Mahalanobis Distance p-value: 0.112

Prediction: Positive if the Bayesian score is above the estimated best cutoff value from minimizing the false positive and false negative rate.
Probability: The esimated probability that the sample is in the positive category. This assumes that the Bayesian score follows a normal distribution and is different from the prediction using a cutoff.
Enrichment: An estimate of enrichment, that is, the increased likelihood (versus random) of this sample being in the category.
Bayesian Score: The standard Laplacian-modified Bayesian score.
Mahalanobis Distance: The Mahalanobis distance (MD) is the distance to the center of the training data. The larger the MD, the less trustworthy the prediction.
Mahalanobis Distance p-value: The p-value gives the fraction of training data with an MD greater than or equal to the one for the given sample, assuming normally distributed data. The smaller the p-value, the less trustworthy the prediciton. For highly non-normal X properties (e.g., fingerprints), the MD p-value is wildly inaccurate.

Structural Similar Compounds

Name	Metolazone	Bicalutamide	Indapamide
Structure	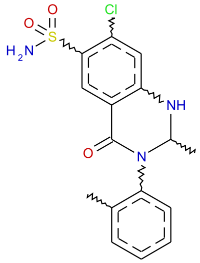	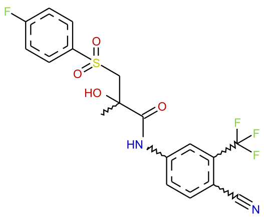	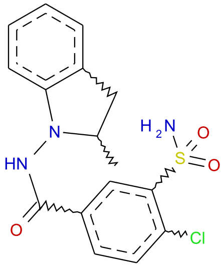
Actual Endpoint	Non-Carcinogen	Carcinogen	Non-Carcinogen
Predicted Endpoint	Non-Carcinogen	Carcinogen	Non-Carcinogen
Distance	0.597	0.599	0.599
Reference	US FDA (Centre for Drug Eval.& Res./Off. Testing & Res.) Sept. 1997	US FDA (Centre for Drug Eval.& Res./Off. Testing & Res.) Sept. 1997	US FDA (Centre for Drug Eval.& Res./Off. Testing & Res.) Sept. 1997

Model Applicability

Unknown features are fingerprint features in the query molecule, but not found or appearing too infrequently in the training set.

1. All properties and OPS components are within expected ranges.

Feature Contribution

Top features for positive contribution				
**Fingerprint**	**Bit/Smiles**	**Feature Structure**	**Score**	**Carcinogen in training set**
FCFP_6	1175665944	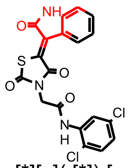 [*]C1=[*][c](:[*]):[c](NC1=O):[cH]:[*]	0.655	7 out of 12

FCFP_6	-1947166985	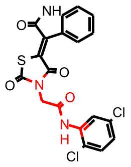 [*]N([*])CC(=O)N[c](:[*]):[*]	0.46	1 out of 1
FCFP_6	2036120522	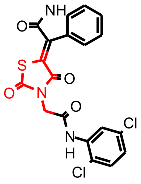 [*]CN1C(=O)SC(=[*])C1=[*]	0.46	1 out of 1

Top Features for negative contribution

Fingerprint	Bit/Smiles	Feature Structure	Score	Carcinogen in training set
FCFP_6	1783756416	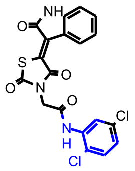 [*]N[c]1:[cH]:[*]:[cH]:[cH]:[c]:1Cl	-0.719	0 out of 4
FCFP_6	-1553874037	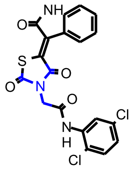 [*]CN1C(=[*])[*][*]C1=[*]	-0.45	5 out of 32
FCFP_6	551850122	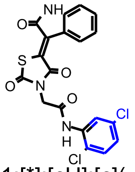 [*][c]1:[*]:[cH]:[c](Cl):[cH]:[cH]:1	-0.433	8 out of 49

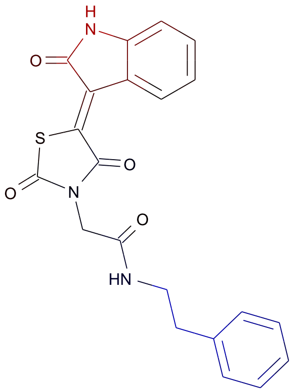

$C_{21}H_{17}N_3O_4S$
Molecular Weight: 407.44237
ALogP: 2.027
Rotatable Bonds: 5
Acceptors: 5
Donors: 2

Model Prediction

Prediction: Non-Carcinogen
Probability: 0.258
Enrichment: 0.878
Bayesian Score: -1.91
Mahalanobis Distance: 11.3
Mahalanobis Distance p-value: 0.0428

Prediction: Positive if the Bayesian score is above the estimated best cutoff value from minimizing the false positive and false negative rate.
Probability: The esimated probability that the sample is in the positive category. This assumes that the Bayesian score follows a normal distribution and is different from the prediction using a cutoff.
Enrichment: An estimate of enrichment, that is, the increased likelihood (versus random) of this sample being in the category.
Bayesian Score: The standard Laplacian-modified Bayesian score.
Mahalanobis Distance: The Mahalanobis distance (MD) is the distance to the center of the training data. The larger the MD, the less trustworthy the prediction.
Mahalanobis Distance p-value: The p-value gives the fraction of training data with an MD greater than or equal to the one for the given sample, assuming normally distributed data. The smaller the p-value, the less trustworthy the prediciton. For highly non-normal X properties (e.g., fingerprints), the MD p-value is wildly inaccurate.

Structural Similar Compounds

Name	Bicalutamide	Acetohexamide	Penicillin
Structure	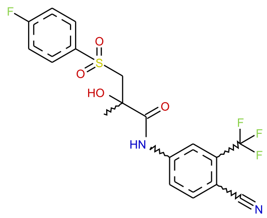	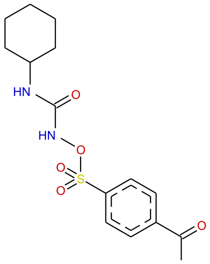	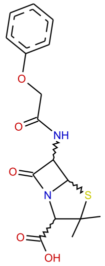
Actual Endpoint	Carcinogen	Non-Carcinogen	Non-Carcinogen
Predicted Endpoint	Carcinogen	Non-Carcinogen	Non-Carcinogen
Distance	0.554	0.571	0.594
Reference	US FDA (Centre for Drug Eval.& Res./Off. Testing & Res.) Sept. 1997	US FDA (Centre for Drug Eval.& Res./Off. Testing & Res.) Sept. 1997	US FDA (Centre for Drug Eval.& Res./Off. Testing & Res.) Sept. 1997

Model Applicability

Unknown features are fingerprint features in the query molecule, but not found or appearing too infrequently in the training set.

1. All properties and OPS components are within expected ranges.

Feature Contribution

Top features for positive contribution				
**Fingerprint**	**Bit/Smiles**	**Feature Structure**	**Score**	**Carcinogen in training set**
FCFP_6	1175665944	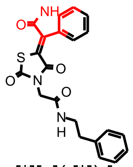 [*]C1=[*][c](:[*]):[c](NC1=O):[cH]:[*]	0.655	7 out of 12

FCFP_6	2036120522	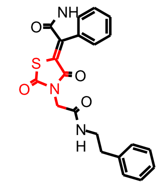 [*]CN1C(=O)SC(=[*])C1=[*]	0.46	1 out of 1
FCFP_6	566058135	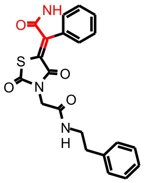 [*]NC(=O)C(=[*])[*]	0.447	17 out of 40
**Top Features for negative contribution**				
**Fingerprint**	**Bit/Smiles**	**Feature Structure**	**Score**	**Carcinogen in training set**
FCFP_6	1981711554	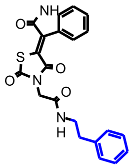 [*]CC[c]1:[cH]:[cH]:[cH]:[cH]:[cH]:1	-1.42	0 out of 12
FCFP_6	-497728148	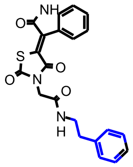 [*]CC[c]1:[cH]:[cH]:[*]:[cH]:[cH]:1	-0.96	2 out of 26
FCFP_6	-1553874037	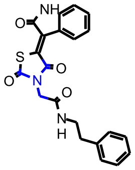 [*]CN1C(=[*])[*][*]C1=[*]	-0.45	5 out of 32

Sorafenib

TOPKAT_Mouse_Male_FDA_None_vs_Carcinogen

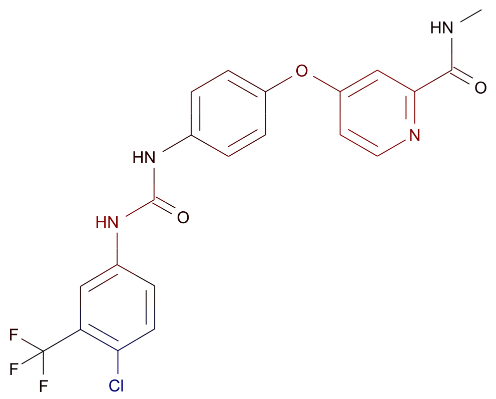

$C_{21}H_{16}ClF_3N_4O_3$
Molecular Weight: 464.82494
ALogP: 4.175
Rotatable Bonds: 6
Acceptors: 4
Donors: 3

Model Prediction

Prediction: Carcinogen
Probability: 0.444
Enrichment: 1.51
Bayesian Score: 4.21
Mahalanobis Distance: 20.3
Mahalanobis Distance p-value: 1.28e-019

Prediction: Positive if the Bayesian score is above the estimated best cutoff value from minimizing the false positive and false negative rate.
Probability: The esimated probability that the sample is in the positive category. This assumes that the Bayesian score follows a normal distribution and is different from the prediction using a cutoff.
Enrichment: An estimate of enrichment, that is, the increased likelihood (versus random) of this sample being in the category.
Bayesian Score: The standard Laplacian-modified Bayesian score.
Mahalanobis Distance: The Mahalanobis distance (MD) is the distance to the center of the training data. The larger the MD, the less trustworthy the prediction.
Mahalanobis Distance p-value: The p-value gives the fraction of training data with an MD greater than or equal to the one for the given sample, assuming normally distributed data. The smaller the p-value, the less trustworthy the prediciton. For highly non-normal X properties (e.g., fingerprints), the MD p-value is wildly inaccurate.

Structural Similar Compounds

Name	Glyburide	Glimepride	Fluvastatin
Structure	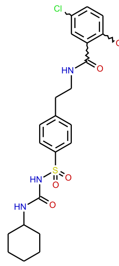	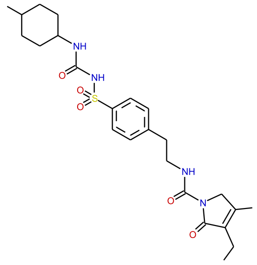	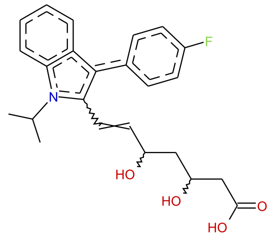
Actual Endpoint	Non-Carcinogen	Carcinogen	Non-Carcinogen
Predicted Endpoint	Non-Carcinogen	Carcinogen	Non-Carcinogen
Distance	0.594	0.599	0.603
Reference	US FDA (Centre for Drug Eval.& Res./Off. Testing & Res.) Sept. 1997	US FDA (Centre for Drug Eval.& Res./Off. Testing & Res.) Sept. 1997	US FDA (Centre for Drug Eval.& Res./Off. Testing & Res.) Sept. 1997

Model Applicability

Unknown features are fingerprint features in the query molecule, but not found or appearing too infrequently in the training set.

1. All properties and OPS components are within expected ranges.

Feature Contribution

Top features for positive contribution				
**Fingerprint**	**Bit/Smiles**	**Feature Structure**	**Score**	**Carcinogen in training set**
FCFP_6	71953198	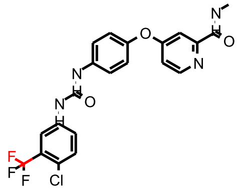 [*]C([*])([*])F	0.612	12 out of 23

FCFP_6	-1838187238	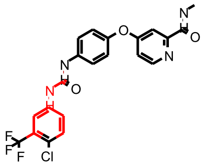<br>[*]C(=[*])N[c]1:[cH]:<br>[cH]:[*]:[cH]:[cH]:1	0.565	4 out of 7
FCFP_6	140656626	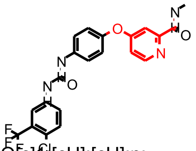<br>[*]O[c]1:[cH]:[cH]:n:<br>[c](:[cH]:1)C(=[*])[<br>*]	0.46	1 out of 1
**Top Features for negative contribution**				
**Fingerprint**	**Bit/Smiles**	**Feature Structure**	**Score**	**Carcinogen in training set**
FCFP_6	2104062943	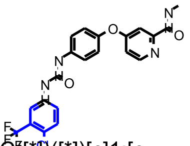<br>[*]C([*])([*])[c]1:[c<br>H]:[*]:[cH]:[cH]:[c]<br>:1Cl	-1.01	1 out of 17
FCFP_6	551850122	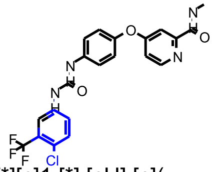<br>[*][c]1:[*]:[cH]:[c](<br>Cl):[cH]:[cH]:1	-0.433	8 out of 49
FCFP_6	71476542	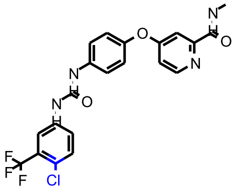<br>[*]:[c](:[*])Cl	-0.406	10 out of 59

Sunitinib

TOPKAT_Mouse_Male_FDA_None_vs_Carcinogen

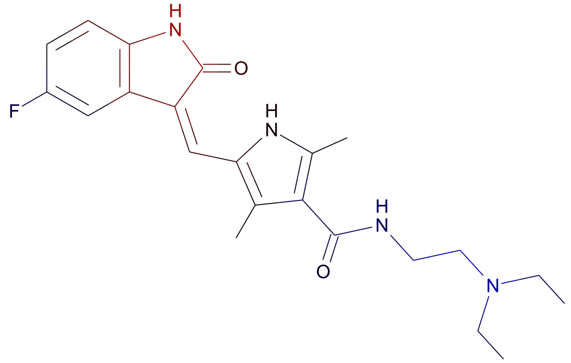

$C_{22}H_{27}FN_4O_2$
Molecular Weight: 398.47378
ALogP: 2.997
Rotatable Bonds: 7
Acceptors: 3
Donors: 3

Structural Similar Compounds

Name	Metoclopramide	Fluvastatin	Torsemide
Structure	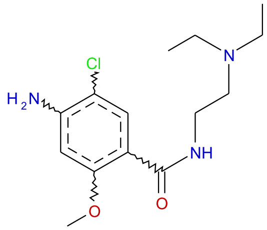	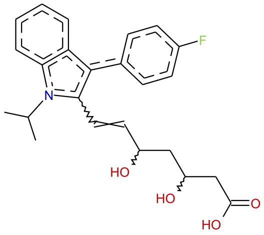	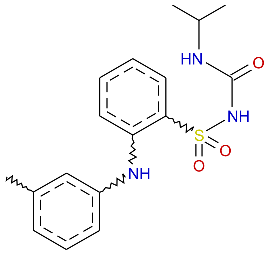
Actual Endpoint	Non-Carcinogen	Non-Carcinogen	Non-Carcinogen
Predicted Endpoint	Non-Carcinogen	Non-Carcinogen	Non-Carcinogen
Distance	0.590	0.603	0.650
Reference	US FDA (Centre for Drug Eval.& Res./Off. Testing & Res.) Sept. 1997	US FDA (Centre for Drug Eval.& Res./Off. Testing & Res.) Sept. 1997	US FDA (Centre for Drug Eval.& Res./Off. Testing & Res.) Sept. 1997

Model Prediction

Prediction: Non-Carcinogen
Probability: 0.247
Enrichment: 0.839
Bayesian Score: -2.43
Mahalanobis Distance: 14.2
Mahalanobis Distance p-value: 2.87e-006

Prediction: Positive if the Bayesian score is above the estimated best cutoff value from minimizing the false positive and false negative rate.
Probability: The esimated probability that the sample is in the positive category. This assumes that the Bayesian score follows a normal distribution and is different from the prediction using a cutoff.
Enrichment: An estimate of enrichment, that is, the increased likelihood (versus random) of this sample being in the category.
Bayesian Score: The standard Laplacian-modified Bayesian score.
Mahalanobis Distance: The Mahalanobis distance (MD) is the distance to the center of the training data. The larger the MD, the less trustworthy the prediction.
Mahalanobis Distance p-value: The p-value gives the fraction of training data with an MD greater than or equal to the one for the given sample, assuming normally distributed data. The smaller the p-value, the less trustworthy the prediciton. For highly non-normal X properties (e.g., fingerprints), the MD p-value is wildly inaccurate.

Model Applicability

Unknown features are fingerprint features in the query molecule, but not found or appearing too infrequently in the training set.

1. All properties and OPS components are within expected ranges.

Feature Contribution

Top features for positive contribution

Fingerprint	Bit/Smiles	Feature Structure	Score	Carcinogen in training set
FCFP_6	1175665944	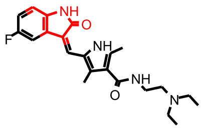 [*]C1=[*][c](:[*]):[c](NC1=O):[cH]:[*]	0.655	7 out of 12

FCFP_6	566058135	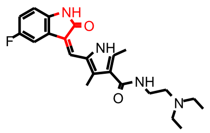 [*]NC(=O)C(=[*])[*]	0.447	17 out of 40
FCFP_6	1294255210	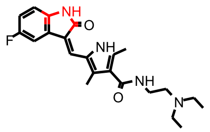 [*]C(=[*])N[c](:[*]): [*]	0.441	12 out of 28

Top Features for negative contribution

Fingerprint	Bit/Smiles	Feature Structure	Score	Carcinogen in training set
FCFP_6	172450560	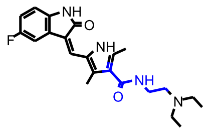 [*]CCNC(=O)[c](:[*]): [*]	-0.839	0 out of 5
FCFP_6	1851332093	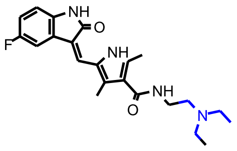 [*]CN(C[*])CC	-0.582	0 out of 3
FCFP_6	-587569116	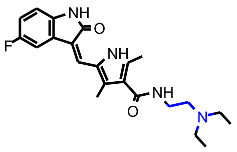 [*]CCN([*])[*]	-0.551	7 out of 49

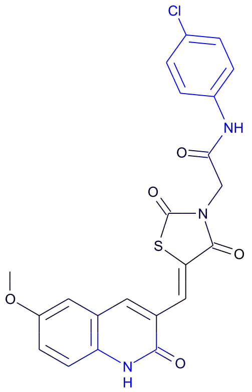

$C_{22}H_{16}ClN_3O_5S$
Molecular Weight: 469.89753
ALogP: 2.815
Rotatable Bonds: 5
Acceptors: 6
Donors: 2

Model Prediction

Prediction: Single-Carcinogen
Probability: 0.124
Enrichment: 0.41
Bayesian Score: -16.6
Mahalanobis Distance: 12.4
Mahalanobis Distance p-value: 0.000276

Prediction: Positive if the Bayesian score is above the estimated best cutoff value from minimizing the false positive and false negative rate.
Probability: The esimated probability that the sample is in the positive category. This assumes that the Bayesian score follows a normal distribution and is different from the prediction using a cutoff.
Enrichment: An estimate of enrichment, that is, the increased likelihood (versus random) of this sample being in the category.
Bayesian Score: The standard Laplacian-modified Bayesian score.
Mahalanobis Distance: The Mahalanobis distance (MD) is the distance to the center of the training data. The larger the MD, the less trustworthy the prediction.
Mahalanobis Distance p-value: The p-value gives the fraction of training data with an MD greater than or equal to the one for the given sample, assuming normally distributed data. The smaller the p-value, the less trustworthy the prediciton. For highly non-normal X properties (e.g., fingerprints), the MD p-value is wildly inaccurate.

Structural Similar Compounds

Name	Bicalutamide	Glimepride	Flunisolide
Structure	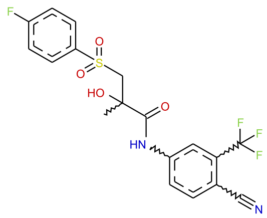	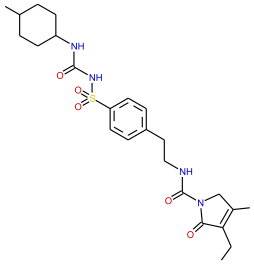	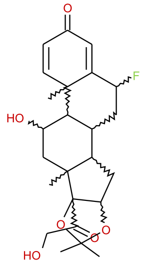
Actual Endpoint	Single-Carcinogen	Single-Carcinogen	Single-Carcinogen
Predicted Endpoint	Single-Carcinogen	Single-Carcinogen	Single-Carcinogen
Distance	0.588	0.743	0.763
Reference	US FDA (Centre for Drug Eval.& Res./Off. Testing & Res.) Sept. 1997	US FDA (Centre for Drug Eval.& Res./Off. Testing & Res.) Sept. 1997	US FDA (Centre for Drug Eval.& Res./Off. Testing & Res.) Sept. 1997

Model Applicability

Unknown features are fingerprint features in the query molecule, but not found or appearing too infrequently in the training set.

1. OPS PC5 out of range. Value: 5.3116. Training min, max, SD, explained variance: -3.5268, 3.8048, 1.733, 0.0560.

Feature Contribution

Top features for positive contribution

Fingerprint	Bit/Smiles	Feature Structure	Score	Multiple-Carcinogen in training set
FCFP_12	451847724	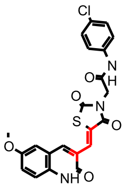 [*]C(=CC(=[*])[*])[*]	0.3	10 out of 21

FCFP_12	436886043	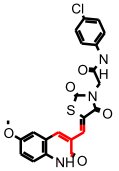 [*]\C=C(\C=[*])/C(=[*])[*]	0.27	7 out of 15
FCFP_12	565998553	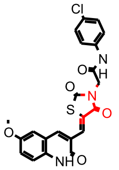 [*]N1[*][*]C(=[*])C1=O	0.194	6 out of 14
**Top Features for negative contribution**				
**Fingerprint**	**Bit/Smiles**	**Feature Structure**	**Score**	**Multiple-Carcinogen in training set**
FCFP_12	1294255210	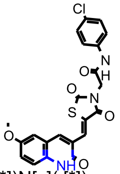 [*]C(=[*])N[c](:[*]):[*]	-1.63	0 out of 12
FCFP_12	1175665944	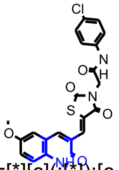 [*]C1=[*][c](:[*]):[c](NC1=O):[cH]:[*]	-1.22	0 out of 7
FCFP_12	590925877	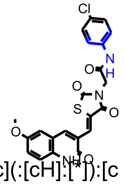 [*]N[c](:[cH]:[*]):[cH]:[*]	-0.998	1 out of 13

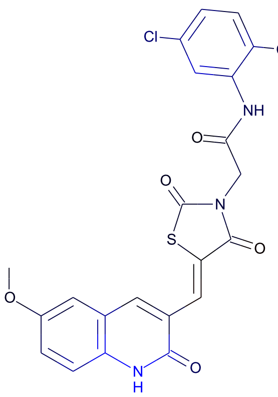

$C_{22}H_{15}Cl_2N_3O_5S$
Molecular Weight: 504.3426
ALogP: 3.479
Rotatable Bonds: 5
Acceptors: 6
Donors: 2

Model Prediction

Prediction: Single-Carcinogen
Probability: 0.142
Enrichment: 0.473
Bayesian Score: -14.3
Mahalanobis Distance: 12.6
Mahalanobis Distance p-value: 0.000217

Prediction: Positive if the Bayesian score is above the estimated best cutoff value from minimizing the false positive and false negative rate.
Probability: The esimated probability that the sample is in the positive category. This assumes that the Bayesian score follows a normal distribution and is different from the prediction using a cutoff.
Enrichment: An estimate of enrichment, that is, the increased likelihood (versus random) of this sample being in the category.
Bayesian Score: The standard Laplacian-modified Bayesian score.
Mahalanobis Distance: The Mahalanobis distance (MD) is the distance to the center of the training data. The larger the MD, the less trustworthy the prediction.
Mahalanobis Distance p-value: The p-value gives the fraction of training data with an MD greater than or equal to the one for the given sample, assuming normally distributed data. The smaller the p-value, the less trustworthy the prediciton. For highly non-normal X properties (e.g., fingerprints), the MD p-value is wildly inaccurate.

Structural Similar Compounds

Name	Bicalutamide	Glimepride	Flunisolide
Structure	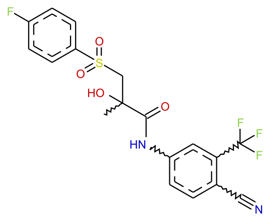	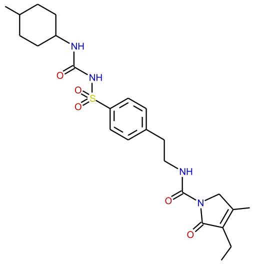	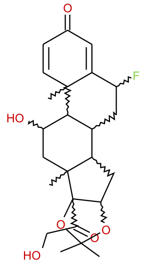
Actual Endpoint	Single-Carcinogen	Single-Carcinogen	Single-Carcinogen
Predicted Endpoint	Single-Carcinogen	Single-Carcinogen	Single-Carcinogen
Distance	0.638	0.728	0.812
Reference	US FDA (Centre for Drug Eval.& Res./Off. Testing & Res.) Sept. 1997	US FDA (Centre for Drug Eval.& Res./Off. Testing & Res.) Sept. 1997	US FDA (Centre for Drug Eval.& Res./Off. Testing & Res.) Sept. 1997

Model Applicability

Unknown features are fingerprint features in the query molecule, but not found or appearing too infrequently in the training set.

1. OPS PC5 out of range. Value: 5.3016. Training min, max, SD, explained variance: -3.5268, 3.8048, 1.733, 0.0560.

Feature Contribution

Top features for positive contribution

Fingerprint	Bit/Smiles	Feature Structure	Score	Multiple-Carcinogen in training set
FCFP_12	451847724	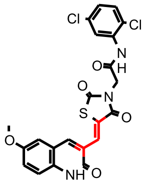 [*]C(=CC(=[*])[*])[*]	0.3	10 out of 21

FCFP_12	436886043	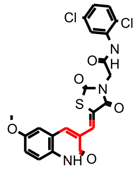 [*]\C=C(\C=[*])/C(=[*])[*]	0.27	7 out of 15
FCFP_12	565998553	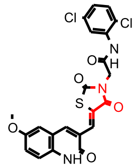 [*]N1[*][*]C(=[*])C1= O	0.194	6 out of 14
**Top Features for negative contribution**				
**Fingerprint**	**Bit/Smiles**	**Feature Structure**	**Score**	**Multiple-Carcinogen in training set**
FCFP_12	1294255210	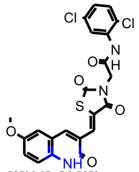 [*]C(=[*])N[c](:[*]): [*]	-1.63	0 out of 12
FCFP_12	1175665944	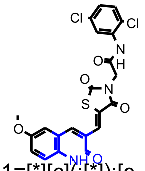 [*]C1=[*][c](:[*]):[c](NC1=O):[cH]:[*]	-1.22	0 out of 7
FCFP_12	590925877	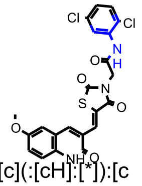 [*]N[c](:[cH]:[*]):[c H]:[*]	-0.998	1 out of 13

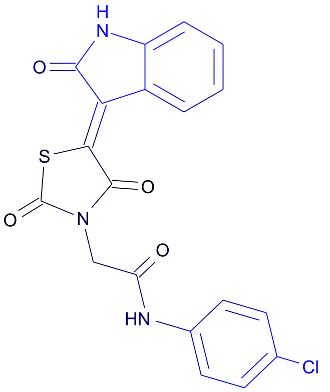

$C_{19}H_{12}ClN_3O_4S$
Molecular Weight: 413.83428
ALogP: 2.363
Rotatable Bonds: 3
Acceptors: 5
Donors: 2

Model Prediction

Prediction: Single-Carcinogen
Probability: 0.103
Enrichment: 0.342
Bayesian Score: -19.4
Mahalanobis Distance: 12.7
Mahalanobis Distance p-value: 0.000168

Prediction: Positive if the Bayesian score is above the estimated best cutoff value from minimizing the false positive and false negative rate.
Probability: The esimated probability that the sample is in the positive category. This assumes that the Bayesian score follows a normal distribution and is different from the prediction using a cutoff.
Enrichment: An estimate of enrichment, that is, the increased likelihood (versus random) of this sample being in the category.
Bayesian Score: The standard Laplacian-modified Bayesian score.
Mahalanobis Distance: The Mahalanobis distance (MD) is the distance to the center of the training data. The larger the MD, the less trustworthy the prediction.
Mahalanobis Distance p-value: The p-value gives the fraction of training data with an MD greater than or equal to the one for the given sample, assuming normally distributed data. The smaller the p-value, the less trustworthy the prediciton. For highly non-normal X properties (e.g., fingerprints), the MD p-value is wildly inaccurate.

Structural Similar Compounds

Name	Bicalutamide	Flunisolide	Sulfamethazine
Structure	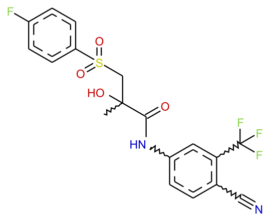	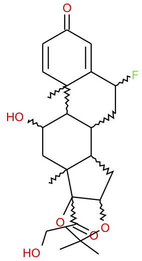	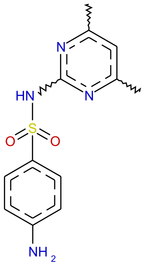
Actual Endpoint	Single-Carcinogen	Single-Carcinogen	Single-Carcinogen
Predicted Endpoint	Single-Carcinogen	Single-Carcinogen	Single-Carcinogen
Distance	0.646	0.707	0.735
Reference	US FDA (Centre for Drug Eval.& Res./Off. Testing & Res.) Sept. 1997	US FDA (Centre for Drug Eval.& Res./Off. Testing & Res.) Sept. 1997	US FDA (Centre for Drug Eval.& Res./Off. Testing & Res.) Sept. 1997

Model Applicability

Unknown features are fingerprint features in the query molecule, but not found or appearing too infrequently in the training set.

1. All properties and OPS components are within expected ranges.

Feature Contribution

Top features for positive contribution

Fingerprint	Bit/Smiles	Feature Structure	Score	Multiple-Carcinogen in training set
FCFP_12	565998553	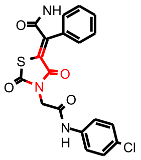 [*]N1[*][*]C(=[*])C1=O	0.194	6 out of 14

Top Features for negative contribution

Fingerprint	Bit/Smiles	Feature Structure	Score	Multiple-Carcinogen in training set
FCFP_12	1294255210	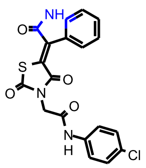 [*]C(=[*])N[c](:[*]):[*]	-1.63	0 out of 12
FCFP_12	1175665944	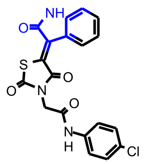 [*]C1=[*][c](:[*]):[c](NC1=O):[cH]:[*]	-1.22	0 out of 7
FCFP_12	590925877	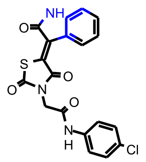 [*]N[c](:[cH]:[*]):[cH]:[*]	-0.998	1 out of 13

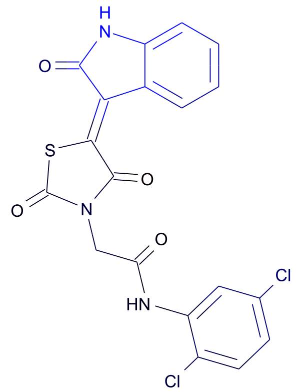

$C_{19}H_{11}Cl_2N_3O_4S$
Molecular Weight: 448.27933
ALogP: 3.028
Rotatable Bonds: 3
Acceptors: 5
Donors: 2

Model Prediction

Prediction: Single-Carcinogen
Probability: 0.119
Enrichment: 0.395
Bayesian Score: -17.2
Mahalanobis Distance: 13.2
Mahalanobis Distance p-value: 7.44e-005

Prediction: Positive if the Bayesian score is above the estimated best cutoff value from minimizing the false positive and false negative rate.
Probability: The esimated probability that the sample is in the positive category. This assumes that the Bayesian score follows a normal distribution and is different from the prediction using a cutoff.
Enrichment: An estimate of enrichment, that is, the increased likelihood (versus random) of this sample being in the category.
Bayesian Score: The standard Laplacian-modified Bayesian score.
Mahalanobis Distance: The Mahalanobis distance (MD) is the distance to the center of the training data. The larger the MD, the less trustworthy the prediction.
Mahalanobis Distance p-value: The p-value gives the fraction of training data with an MD greater than or equal to the one for the given sample, assuming normally distributed data. The smaller the p-value, the less trustworthy the prediciton. For highly non-normal X properties (e.g., fingerprints), the MD p-value is wildly inaccurate.

Structural Similar Compounds

Name	Bicalutamide	Flunisolide	Phenolphthalein
Structure	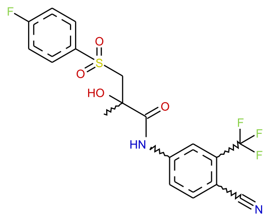	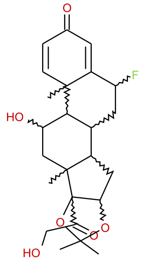	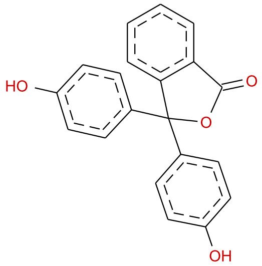
Actual Endpoint	Single-Carcinogen	Single-Carcinogen	Multiple-Carcinogen
Predicted Endpoint	Single-Carcinogen	Single-Carcinogen	Multiple-Carcinogen
Distance	0.645	0.730	0.753
Reference	US FDA (Centre for Drug Eval.& Res./Off. Testing & Res.) Sept. 1997	US FDA (Centre for Drug Eval.& Res./Off. Testing & Res.) Sept. 1997	US FDA (Centre for Drug Eval.& Res./Off. Testing & Res.) Sept. 1997

Model Applicability

Unknown features are fingerprint features in the query molecule, but not found or appearing too infrequently in the training set.

1. All properties and OPS components are within expected ranges.

Feature Contribution

Top features for positive contribution

Fingerprint	Bit/Smiles	Feature Structure	Score	Multiple-Carcinogen in training set
FCFP_12	565998553	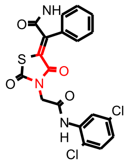 [*]N1[*][*]C(=[*])C1= O	0.194	6 out of 14

Top Features for negative contribution

Fingerprint	Bit/Smiles	Feature Structure	Score	Multiple-Carcinogen in training set
FCFP_12	1294255210	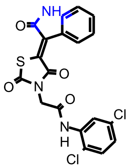 [*]C(=[*])N[c](:[*]):[*]	-1.63	0 out of 12
FCFP_12	1175665944	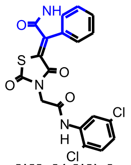 [*]C1=[*][c](:[*]):[c](NC1=O):[cH]:[*]	-1.22	0 out of 7
FCFP_12	590925877	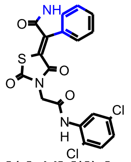 [*]N[c](:[cH]:[*]):[cH]:[*]	-0.998	1 out of 13

Sorafenib

TOPKAT_Mouse_Male_FDA_Single_vs_Multiple

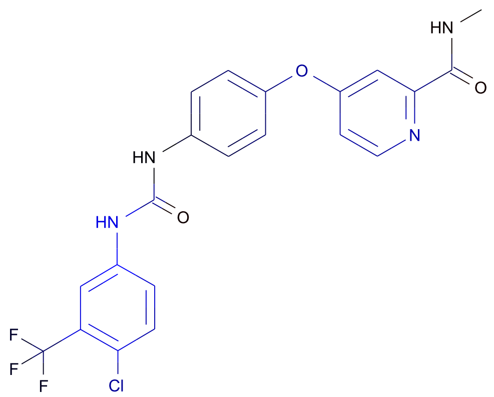

$C_{21}H_{16}ClF_3N_4O_3$
Molecular Weight: 464.82494
ALogP: 4.175
Rotatable Bonds: 6
Acceptors: 4
Donors: 3

Model Prediction

Prediction: Single-Carcinogen
Probability: 0.139
Enrichment: 0.461
Bayesian Score: -14.7
Mahalanobis Distance: 21.3
Mahalanobis Distance p-value: 4.93e-011

Prediction: Positive if the Bayesian score is above the estimated best cutoff value from minimizing the false positive and false negative rate.
Probability: The esimated probability that the sample is in the positive category. This assumes that the Bayesian score follows a normal distribution and is different from the prediction using a cutoff.
Enrichment: An estimate of enrichment, that is, the increased likelihood (versus random) of this sample being in the category.
Bayesian Score: The standard Laplacian-modified Bayesian score.
Mahalanobis Distance: The Mahalanobis distance (MD) is the distance to the center of the training data. The larger the MD, the less trustworthy the prediction.
Mahalanobis Distance p-value: The p-value gives the fraction of training data with an MD greater than or equal to the one for the given sample, assuming normally distributed data. The smaller the p-value, the less trustworthy the prediciton. For highly non-normal X properties (e.g., fingerprints), the MD p-value is wildly inaccurate.

Structural Similar Compounds

Name	Glimepride	Bicalutamide	Lansoprazole
Structure	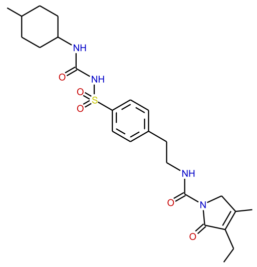	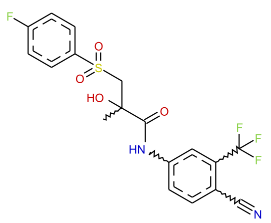	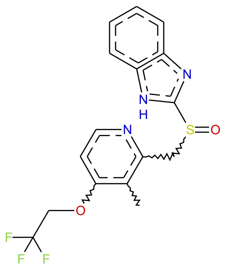
Actual Endpoint	Single-Carcinogen	Single-Carcinogen	Single-Carcinogen
Predicted Endpoint	Single-Carcinogen	Single-Carcinogen	Single-Carcinogen
Distance	0.626	0.700	0.866
Reference	US FDA (Centre for Drug Eval.& Res./Off. Testing & Res.) Sept. 1997	US FDA (Centre for Drug Eval.& Res./Off. Testing & Res.) Sept. 1997	US FDA (Centre for Drug Eval.& Res./Off. Testing & Res.) Sept. 1997

Model Applicability

Unknown features are fingerprint features in the query molecule, but not found or appearing too infrequently in the training set.

1. All properties and OPS components are within expected ranges.

Feature Contribution

Top features for positive contribution

Fingerprint	Bit/Smiles	Feature Structure	Score	Multiple-Carcinogen in training set
FCFP_12	1499521844	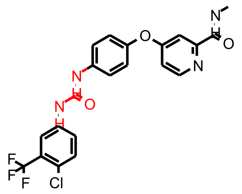 [*]NC(=O)N[*]	0.39	5 out of 9

FCFP_12	-904785030	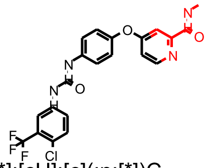<br>[*]:[cH]:[c](:n:[*])C<br>(=O)NC	0.174	1 out of 2
FCFP_12	-1549103449	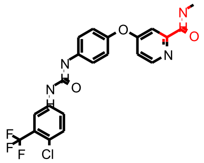<br>[*]NC(=O)[c](:[*]):[*<br>]	0.168	3 out of 7

Top Features for negative contribution

Fingerprint	Bit/Smiles	Feature Structure	Score	Multiple-Carcinogen in training set
FCFP_12	1294255210	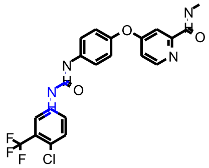<br>[*]C(=[*])N[c](:[*]):<br>[*]	-1.63	0 out of 12
FCFP_12	590925877	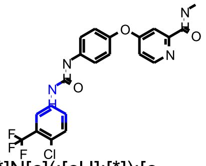<br>[*]N[c](:[cH]:[*]):[c<br>H]:[*]	-0.998	1 out of 13
FCFP_12	-1462709112	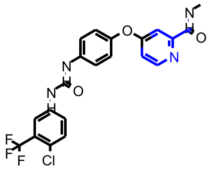<br>[*]C(=[*])[c]1:[cH]:[<br>*]:[cH]:[cH]:n:1	-0.994	0 out of 5

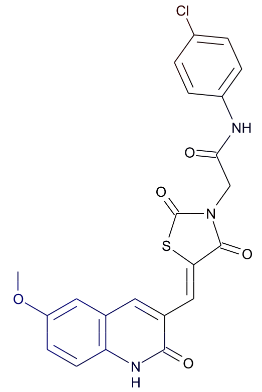

$C_{22}H_{16}ClN_3O_5S$
Molecular Weight: 469.89753
ALogP: 2.815
Rotatable Bonds: 5
Acceptors: 6
Donors: 2

Model Prediction

Prediction: Mild
Probability: 0.735
Enrichment: 1.07
Bayesian Score: -2.83
Mahalanobis Distance: 11.9
Mahalanobis Distance p-value: 0.000106

Prediction: Positive if the Bayesian score is above the estimated best cutoff value from minimizing the false positive and false negative rate.
Probability: The esimated probability that the sample is in the positive category. This assumes that the Bayesian score follows a normal distribution and is different from the prediction using a cutoff.
Enrichment: An estimate of enrichment, that is, the increased likelihood (versus random) of this sample being in the category.
Bayesian Score: The standard Laplacian-modified Bayesian score.
Mahalanobis Distance: The Mahalanobis distance (MD) is the distance to the center of the training data. The larger the MD, the less trustworthy the prediction.
Mahalanobis Distance p-value: The p-value gives the fraction of training data with an MD greater than or equal to the one for the given sample, assuming normally distributed data. The smaller the p-value, the less trustworthy the prediciton. For highly non-normal X properties (e.g., fingerprints), the MD p-value is wildly inaccurate.

Structural Similar Compounds

Name	5-NORBORNENE-2;3-DICARBOXYLIC ACID; 1;4;5;6;7;7-HEXACHLORO-	ANTHRAQUINONE; 1-AMINO-4-HYDROXY-2-PHENOXY-	4;4'-DIAMINO-1;1'-DIANTHRIMIDE
Structure	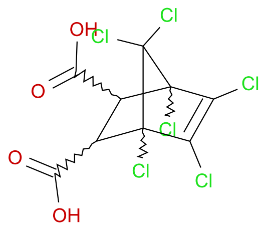	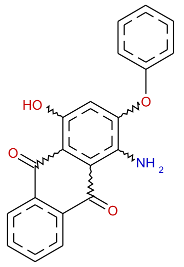	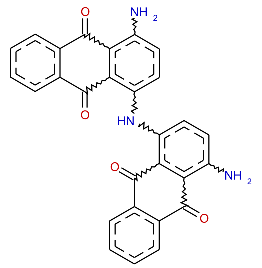
Actual Endpoint	Moderate_Severe	Mild	Mild
Predicted Endpoint	Moderate_Severe	Mild	Mild
Distance	0.742	0.745	0.751
Reference	28ZPAK-;92;72	28ZPAK 239;72	28ZPAK-;125;72

Model Applicability

Unknown features are fingerprint features in the query molecule, but not found or appearing too infrequently in the training set.

1. All properties and OPS components are within expected ranges.

Feature Contribution

Top features for positive contribution				
**Fingerprint**	**Bit/Smiles**	**Feature Structure**	**Score**	**Moderate_Severe in training set**
FCFP_10	-1508180856	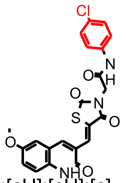 [*][c]1:[cH]:[cH]:[c](Cl):[cH]:[cH]:1	0.329	16 out of 17

FCFP_10	-745491832	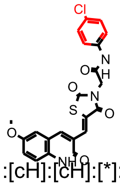 Cl[c]1:[cH]:[cH]:[*]:[cH]:[cH]:1	0.304	29 out of 32
FCFP_10	346218766	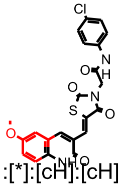 [*][c]1:[*]:[cH]:[cH]:[c](OC):[cH]:1	0.197	30 out of 37
**Top Features for negative contribution**				
**Fingerprint**	**Bit/Smiles**	**Feature Structure**	**Score**	**Moderate_Severe in training set**
FCFP_10	-1977641857	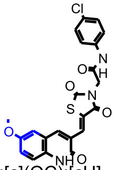 [*]:[cH]:[c](OC):[cH]:[*]	-0.78	4 out of 15
FCFP_10	723745966	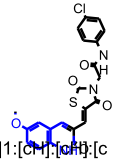 [*]O[c]1:[cH]:[cH]:[c]2NC(=[*])[*]=C[c]:2:[cH]:1	-0.507	0 out of 1
FCFP_10	-1757681964	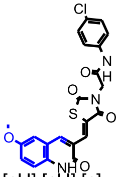 [*][c]1:[cH]:[cH]:[c](OC):[cH]:[c]:1C=[*]	-0.507	0 out of 1

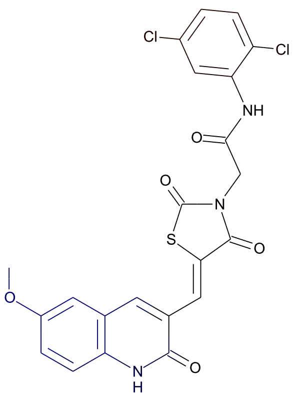

$C_{22}H_{15}Cl_2N_3O_5S$
Molecular Weight: 504.3426
ALogP: 3.479
Rotatable Bonds: 5
Acceptors: 6
Donors: 2

Structural Similar Compounds

Name	4;4'-DIAMINO-1;1'-DIANTHRIMIDE	5-NORBORNENE-2;3-DICARBOXYLIC ACID; 1;4;5;6;7;7-HEXACHLORO-	ANTHRAQUINONE; 1-AMINO-4-HYDROXY-2-PHENOXY-
Structure	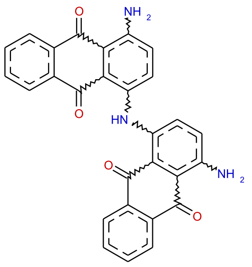	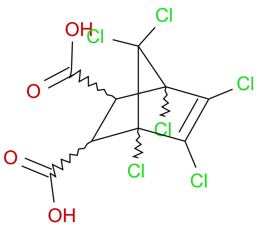	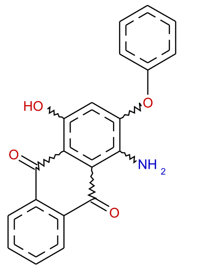
Actual Endpoint	Mild	Moderate_Severe	Mild
Predicted Endpoint	Mild	Moderate_Severe	Mild
Distance	0.747	0.790	0.793
Reference	28ZPAK-;125;72	28ZPAK-;92;72	28ZPAK 239;72

Model Prediction

Prediction: Mild
Probability: 0.74
Enrichment: 1.07
Bayesian Score: -2.74
Mahalanobis Distance: 12.1
Mahalanobis Distance p-value: 4.02e-005

Prediction: Positive if the Bayesian score is above the estimated best cutoff value from minimizing the false positive and false negative rate.
Probability: The esimated probability that the sample is in the positive category. This assumes that the Bayesian score follows a normal distribution and is different from the prediction using a cutoff.
Enrichment: An estimate of enrichment, that is, the increased likelihood (versus random) of this sample being in the category.
Bayesian Score: The standard Laplacian-modified Bayesian score.
Mahalanobis Distance: The Mahalanobis distance (MD) is the distance to the center of the training data. The larger the MD, the less trustworthy the prediction.
Mahalanobis Distance p-value: The p-value gives the fraction of training data with an MD greater than or equal to the one for the given sample, assuming normally distributed data. The smaller the p-value, the less trustworthy the prediciton. For highly non-normal X properties (e.g., fingerprints), the MD p-value is wildly inaccurate.

Model Applicability

Unknown features are fingerprint features in the query molecule, but not found or appearing too infrequently in the training set.

1. All properties and OPS components are within expected ranges.

Feature Contribution

Top features for positive contribution

Fingerprint	Bit/Smiles	Feature Structure	Score	Moderate_Severe in training set
FCFP_10	-745491832	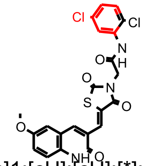 Cl[c]1:[cH]:[cH]:[*]:[cH]:[cH]:1	0.304	29 out of 32

FCFP_10	346218766	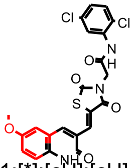 [*][c]1:[*]:[cH]:[cH]:[c](OC):[cH]:1	0.197	30 out of 37
FCFP_10	562194858	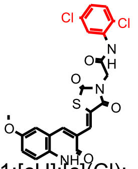 [*][c]1:[cH]:[c](Cl):[cH]:[cH]:[c]:1Cl	0.186	1 out of 1

Top Features for negative contribution

Fingerprint	Bit/Smiles	Feature Structure	Score	Moderate_Severe in training set
FCFP_10	-1977641857	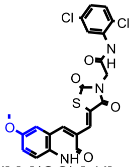 [*]:[cH]:[c](OC):[cH]:[*]	-0.78	4 out of 15
FCFP_10	-1757681964	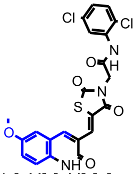 [*][c]1:[cH]:[cH]:[c](OC):[cH]:[c]:1C=[*]	-0.507	0 out of 1
FCFP_10	723745966	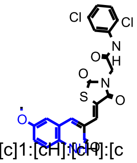 [*]O[c]1:[cH]:[cH]:[c]2NC(=[*])[*]=C[c]:2:[cH]:1	-0.507	0 out of 1

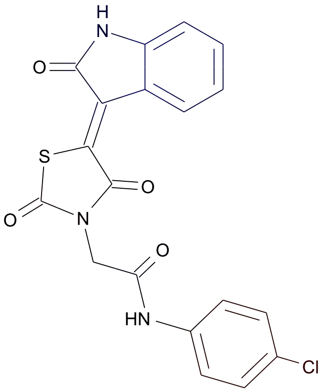

$C_{19}H_{12}ClN_3O_4S$
Molecular Weight: 413.83428
ALogP: 2.363
Rotatable Bonds: 3
Acceptors: 5
Donors: 2

Model Prediction

Prediction: Mild
Probability: 0.787
Enrichment: 1.14
Bayesian Score: -1.47
Mahalanobis Distance: 11.2
Mahalanobis Distance p-value: 0.00241

Prediction: Positive if the Bayesian score is above the estimated best cutoff value from minimizing the false positive and false negative rate.
Probability: The esimated probability that the sample is in the positive category. This assumes that the Bayesian score follows a normal distribution and is different from the prediction using a cutoff.
Enrichment: An estimate of enrichment, that is, the increased likelihood (versus random) of this sample being in the category.
Bayesian Score: The standard Laplacian-modified Bayesian score.
Mahalanobis Distance: The Mahalanobis distance (MD) is the distance to the center of the training data. The larger the MD, the less trustworthy the prediction.
Mahalanobis Distance p-value: The p-value gives the fraction of training data with an MD greater than or equal to the one for the given sample, assuming normally distributed data. The smaller the p-value, the less trustworthy the prediciton. For highly non-normal X properties (e.g., fingerprints), the MD p-value is wildly inaccurate.

Structural Similar Compounds

Name	1-AMINO-4-BENZOYLAMINO-ANTHRAQUINONE	5-NORBORNENE-2;3-DICARBOXYLIC ACID; 1;4;5;6;7;7-HEXACHLORO-	ANTHRAQUINONE; 1-AMINO-4-HYDROXY-2-PHENOXY-
Structure	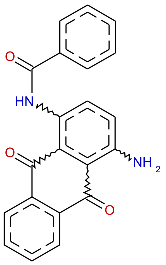	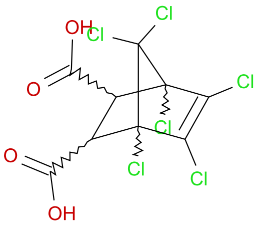	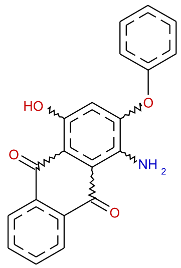
Actual Endpoint	Mild	Moderate_Severe	Mild
Predicted Endpoint	Mild	Moderate_Severe	Mild
Distance	0.624	0.630	0.645
Reference	28ZPAK-;124;72	28ZPAK-;92;72	28ZPAK 239;72

Model Applicability

Unknown features are fingerprint features in the query molecule, but not found or appearing too infrequently in the training set.

1. All properties and OPS components are within expected ranges.

Feature Contribution

Top features for positive contribution

Fingerprint	Bit/Smiles	Feature Structure	Score	Moderate_Severe in training set
FCFP_10	-1508180856	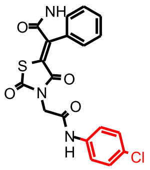 [*][c]1:[cH]:[cH]:[c](Cl):[cH]:[cH]:1	0.329	16 out of 17

FCFP_10	-745491832	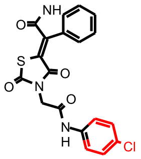 Cl[c]1:[cH]:[cH]:[*]:[cH]:[cH]:1	0.304	29 out of 32
FCFP_10	-175681259	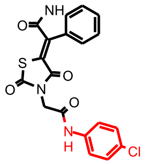 [*]N[c]1:[cH]:[cH]:[c](Cl):[cH]:[cH]:1	0.186	1 out of 1

Top Features for negative contribution

Fingerprint	Bit/Smiles	Feature Structure	Score	Moderate_Severe in training set
FCFP_10	1011367537	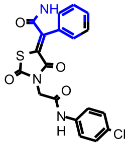 [*]=C1[*]=C[c]2:[cH]:[*]:[cH]:[cH]:[c]:2N1	-0.329	4 out of 9
FCFP_10	-773983804	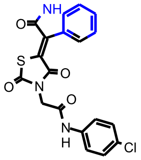 [*]N[c]1:[cH]:[*]:[c]([*]):[cH]:[cH]:1	-0.294	50 out of 102
FCFP_10	-1698724694	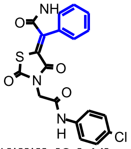 [*]=C1[*][*][c]2:[cH]:[cH]:[cH]:[cH]:[c]1:2	-0.284	53 out of 107

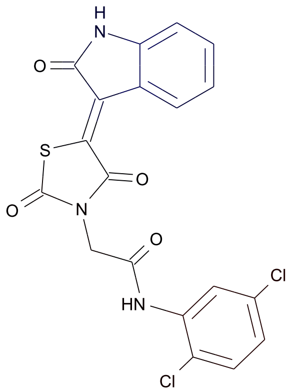

$C_{19}H_{11}Cl_2N_3O_4S$
Molecular Weight: 448.27933
ALogP: 3.028
Rotatable Bonds: 3
Acceptors: 5
Donors: 2

Model Prediction

Prediction: Mild
Probability: 0.793
Enrichment: 1.15
Bayesian Score: -1.28
Mahalanobis Distance: 11.3
Mahalanobis Distance p-value: 0.00136

Prediction: Positive if the Bayesian score is above the estimated best cutoff value from minimizing the false positive and false negative rate.
Probability: The esimated probability that the sample is in the positive category. This assumes that the Bayesian score follows a normal distribution and is different from the prediction using a cutoff.
Enrichment: An estimate of enrichment, that is, the increased likelihood (versus random) of this sample being in the category.
Bayesian Score: The standard Laplacian-modified Bayesian score.
Mahalanobis Distance: The Mahalanobis distance (MD) is the distance to the center of the training data. The larger the MD, the less trustworthy the prediction.
Mahalanobis Distance p-value: The p-value gives the fraction of training data with an MD greater than or equal to the one for the given sample, assuming normally distributed data. The smaller the p-value, the less trustworthy the prediciton. For highly non-normal X properties (e.g., fingerprints), the MD p-value is wildly inaccurate.

Structural Similar Compounds

Name	5-NORBORNENE-2;3-DICARBOXYLIC ACID; 1;4;5;6;7;7-HEXACHLORO-	1-AMINO-4-BENZOYLAMINO-ANTHRAQUINONE	ANTHRAQUINONE; 1-AMINO-4-HYDROXY-2-PHENOXY-
Structure	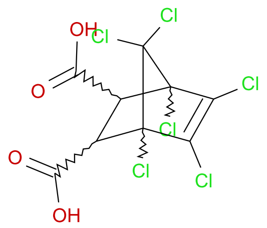	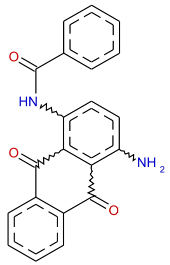	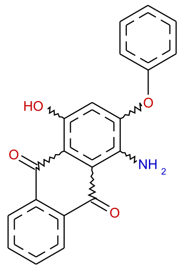
Actual Endpoint	Moderate_Severe	Mild	Mild
Predicted Endpoint	Moderate_Severe	Mild	Mild
Distance	0.652	0.658	0.669
Reference	28ZPAK-;92;72	28ZPAK-;124;72	28ZPAK 239;72

Model Applicability

Unknown features are fingerprint features in the query molecule, but not found or appearing too infrequently in the training set.

1. All properties and OPS components are within expected ranges.

Feature Contribution

Top features for positive contribution				
**Fingerprint**	**Bit/Smiles**	**Feature Structure**	**Score**	**Moderate_Severe in training set**
FCFP_10	-745491832	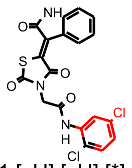 Cl[c]1:[cH]:[cH]:[*]:[cH]:[cH]:1	0.304	29 out of 32

FCFP_10	562194858	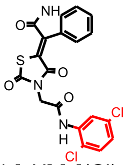 [*][c]1:[cH]:[c](Cl):[cH]:[cH]:[c]:1Cl	0.186	1 out of 1
FCFP_10	3	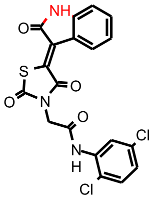 [*]N[*]	0.165	383 out of 491

Top Features for negative contribution

Fingerprint	Bit/Smiles	Feature Structure	Score	Moderate_Severe in training set
FCFP_10	1011367537	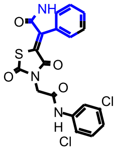 [*]=C1[*]=C[c]2:[cH]:[*]:[cH]:[cH]:[c]:2N1	-0.329	4 out of 9
FCFP_10	-773983804	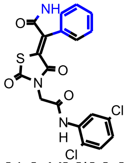 [*]N[c]1:[cH]:[*]:[c]([*]):[cH]:[cH]:1	-0.294	50 out of 102
FCFP_10	-1698724694	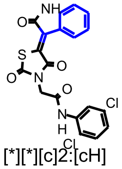 [*]=C1[*][*][c]2:[cH]:[cH]:[cH]:[cH]:[c]1:2	-0.284	53 out of 107

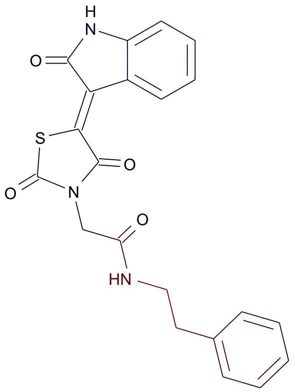

$C_{21}H_{17}N_3O_4S$
Molecular Weight: 407.44237
ALogP: 2.027
Rotatable Bonds: 5
Acceptors: 5
Donors: 2

Model Prediction

Prediction: Moderate_Severe
Probability: 0.831
Enrichment: 1.21
Bayesian Score: 0.573
Mahalanobis Distance: 11
Mahalanobis Distance p-value: 0.00418

Prediction: Positive if the Bayesian score is above the estimated best cutoff value from minimizing the false positive and false negative rate.
Probability: The esimated probability that the sample is in the positive category. This assumes that the Bayesian score follows a normal distribution and is different from the prediction using a cutoff.
Enrichment: An estimate of enrichment, that is, the increased likelihood (versus random) of this sample being in the category.
Bayesian Score: The standard Laplacian-modified Bayesian score.
Mahalanobis Distance: The Mahalanobis distance (MD) is the distance to the center of the training data. The larger the MD, the less trustworthy the prediction.
Mahalanobis Distance p-value: The p-value gives the fraction of training data with an MD greater than or equal to the one for the given sample, assuming normally distributed data. The smaller the p-value, the less trustworthy the prediciton. For highly non-normal X properties (e.g., fingerprints), the MD p-value is wildly inaccurate.

Structural Similar Compounds

Name	1-AMINO-4-BENZOYLAMINO-ANTHRAQUINONE	5-NORBORNENE-2;3-DICARBOXYLIC ACID; 1;4;5;6;7;7-HEXACHLORO-	ANTHRAQUINONE; 1-AMINO-4-HYDROXY-2-PHENOXY-
Structure	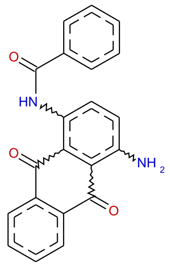	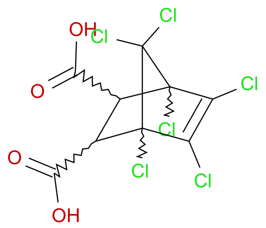	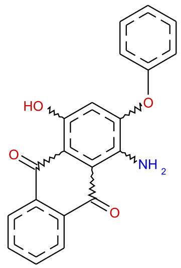
Actual Endpoint	Mild	Moderate_Severe	Mild
Predicted Endpoint	Mild	Moderate_Severe	Mild
Distance	0.637	0.648	0.668
Reference	28ZPAK-;124;72	28ZPAK-;92;72	28ZPAK 239;72

Model Applicability

Unknown features are fingerprint features in the query molecule, but not found or appearing too infrequently in the training set.

1. All properties and OPS components are within expected ranges.

Feature Contribution

Top features for positive contribution				
**Fingerprint**	**Bit/Smiles**	**Feature Structure**	**Score**	**Moderate_Severe in training set**
FCFP_10	-497728148	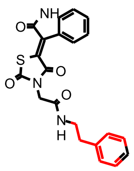 [*]CC[c]1:[cH]:[cH]:[*]:[cH]:[cH]:1	0.356	24 out of 25

FCFP_10	-547731249	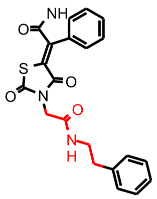 [*]CCNC(=O)C[*]	0.294	3 out of 3
FCFP_10	-1272709286	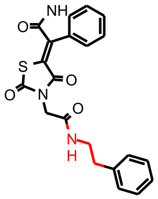 [*]CCN[*]	0.285	234 out of 266

Top Features for negative contribution

**Fingerprint**	**Bit/Smiles**	**Feature Structure**	**Score**	**Moderate_Severe in training set**
FCFP_10	1011367537	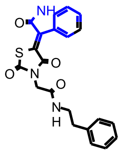 [*]=C1[*]=C[c]2:[cH]:[*]:[cH]:[cH]:[c]:2N1	-0.329	4 out of 9
FCFP_10	-773983804	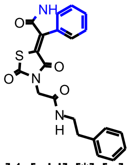 [*]N[c]1:[cH]:[*]:[c]([*]):[cH]:[cH]:1	-0.294	50 out of 102
FCFP_10	-1698724694	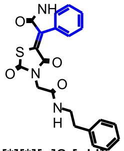 [*]=C1[*][*][c]2:[cH]:[cH]:[cH]:[cH]:[c]1:2	-0.284	53 out of 107

Sorafenib

TOPKAT_Ocular_Irritancy_Mild_vs_Moderate_Severe

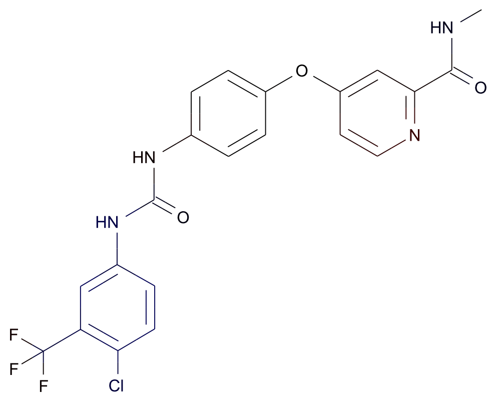

$C_{21}H_{16}ClF_3N_4O_3$
Molecular Weight: 464.82494
ALogP: 4.175
Rotatable Bonds: 6
Acceptors: 4
Donors: 3

Model Prediction

Prediction: Mild
Probability: 0.776
Enrichment: 1.13
Bayesian Score: -1.8
Mahalanobis Distance: 8.95
Mahalanobis Distance p-value: 0.537

Prediction: Positive if the Bayesian score is above the estimated best cutoff value from minimizing the false positive and false negative rate.
Probability: The esimated probability that the sample is in the positive category. This assumes that the Bayesian score follows a normal distribution and is different from the prediction using a cutoff.
Enrichment: An estimate of enrichment, that is, the increased likelihood (versus random) of this sample being in the category.
Bayesian Score: The standard Laplacian-modified Bayesian score.
Mahalanobis Distance: The Mahalanobis distance (MD) is the distance to the center of the training data. The larger the MD, the less trustworthy the prediction.
Mahalanobis Distance p-value: The p-value gives the fraction of training data with an MD greater than or equal to the one for the given sample, assuming normally distributed data. The smaller the p-value, the less trustworthy the prediciton. For highly non-normal X properties (e.g., fingerprints), the MD p-value is wildly inaccurate.

Structural Similar Compounds

Name	4;4'-DIAMINO-1;1'-DIANTHRIMIDE	5-NORBORNENE-2;3-DICARBOXYLIC ACID; 1;4;5;6;7;7-HEXACHLORO-	METHANE;TRIS(4-AMINOPHENYL)-
Structure	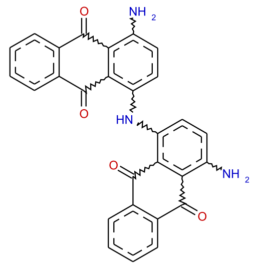	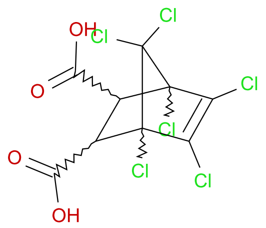	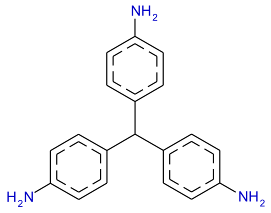
Actual Endpoint	Mild	Moderate_Severe	Moderate_Severe
Predicted Endpoint	Mild	Moderate_Severe	Moderate_Severe
Distance	0.799	0.816	0.827
Reference	28ZPAK-;125;72	28ZPAK-;92;72	28ZPAK-;73;72

Model Applicability

Unknown features are fingerprint features in the query molecule, but not found or appearing too infrequently in the training set.

1. All properties and OPS components are within expected ranges.

Feature Contribution

Top features for positive contribution				
**Fingerprint**	**Bit/Smiles**	**Feature Structure**	**Score**	**Moderate_Severe in training set**
FCFP_10	-1695756380	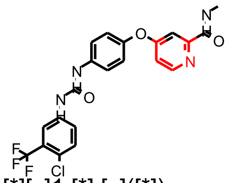 [*][c]1:[*]:[c]([*]):n:[cH]:[cH]:1	0.285	10 out of 11

FCFP_10	-124655670	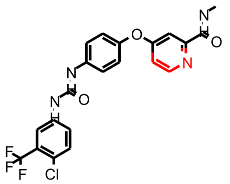 [*]:[cH]:[cH]:n:[*]	0.259	14 out of 16
FCFP_10	-885550502	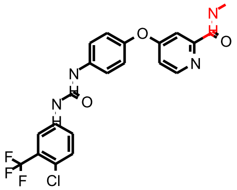 [*]CNC(=[*])[*]	0.239	54 out of 64

Top Features for negative contribution

Fingerprint	Bit/Smiles	Feature Structure	Score	Moderate_Severe in training set
FCFP_10	2104062943	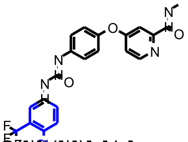 [*]C([*])([*])[c]1:[cH]:[*]:[cH]:[cH]:[c]:1Cl	-0.745	7 out of 24
FCFP_10	-174293376	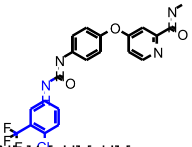 [*]N[c]1:[cH]:[cH]:[c](Cl):[c](:[cH]:1)C([*])([*])[*]	-0.507	0 out of 1
FCFP_10	-1549103449	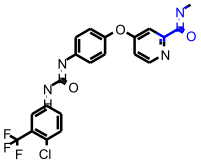 [*]NC(=O)[c](:[*]):[*]	-0.504	2 out of 6

Sunitinib

TOPKAT_Ocular_Irritancy_Mild_vs_Moderate_Severe

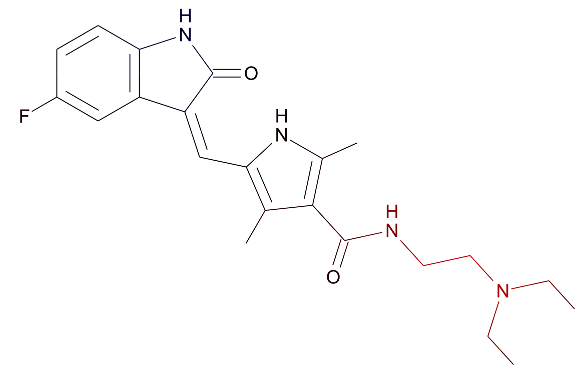

$C_{22}H_{27}FN_4O_2$

Molecular Weight: 398.47378

ALogP: 2.997

Rotatable Bonds: 7

Acceptors: 3

Donors: 3

Model Prediction

Prediction: Moderate_Severe

Probability: 0.893

Enrichment: 1.3

Bayesian Score: 3.24

Mahalanobis Distance: 11.1

Mahalanobis Distance p-value: 0.00304

Prediction: Positive if the Bayesian score is above the estimated best cutoff value from minimizing the false positive and false negative rate.
Probability: The esimated probability that the sample is in the positive category. This assumes that the Bayesian score follows a normal distribution and is different from the prediction using a cutoff.
Enrichment: An estimate of enrichment, that is, the increased likelihood (versus random) of this sample being in the category.
Bayesian Score: The standard Laplacian-modified Bayesian score.
Mahalanobis Distance: The Mahalanobis distance (MD) is the distance to the center of the training data. The larger the MD, the less trustworthy the prediction.
Mahalanobis Distance p-value: The p-value gives the fraction of training data with an MD greater than or equal to the one for the given sample, assuming normally distributed data. The smaller the p-value, the less trustworthy the prediciton. For highly non-normal X properties (e.g., fingerprints), the MD p-value is wildly inaccurate.

Structural Similar Compounds

Name	METHANE;TRIS(4-AMINOPHENYL)-	ANTHRAQUINONE; 1-((2-HYDROXYETHYL)AMINO)-4-(METHYLAMINO)-	1-AMINO-4-BENZOYLAMINO-ANTHRAQUINONE
Structure	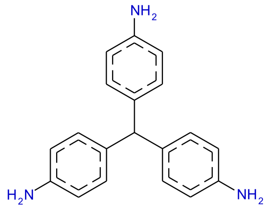	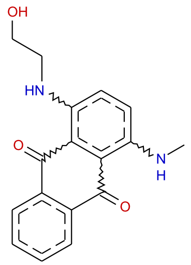	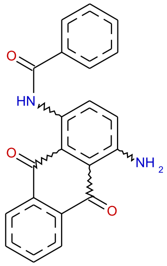
Actual Endpoint	Moderate_Severe	Mild	Mild
Predicted Endpoint	Moderate_Severe	Mild	Mild
Distance	0.713	0.770	0.786
Reference	28ZPAK-;73;72	28ZPAK 245;72	28ZPAK-;124;72

Model Applicability

Unknown features are fingerprint features in the query molecule, but not found or appearing too infrequently in the training set.

1. All properties and OPS components are within expected ranges.
2. Unknown FCFP_2 feature: 203707511: [*][c]1:[*]:[*]:[nH]:[c]:1C=[*]

Feature Contribution

Top features for positive contribution

Fingerprint	Bit/Smiles	Feature Structure	Score	Moderate_Severe in training set
FCFP_10	1851332093	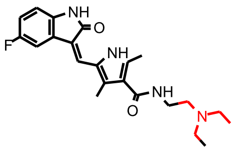 [*]CN(C[*])CC	0.376	12 out of 12

FCFP_10	-371808660	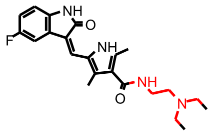 [*]CN(C[*])CCN[*]	0.338	18 out of 19
FCFP_10	-587569116	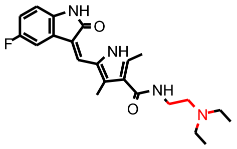 [*]CCN([*])[*]	0.335	66 out of 71

Top Features for negative contribution

**Fingerprint**	**Bit/Smiles**	**Feature Structure**	**Score**	**Moderate_Severe in training set**
FCFP_10	-1549103449	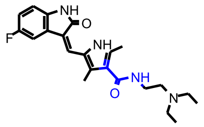 [*]NC(=O)[c](:[*]):[*]	-0.504	2 out of 6
FCFP_10	1011367537	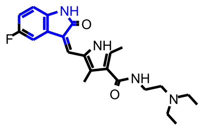 [*]=C1[*]=C[c]2:[cH]:[*]:[cH]:[cH]:[c]:2N 1	-0.329	4 out of 9
FCFP_10	-773983804	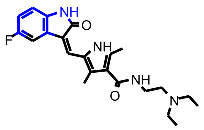 [*]N[c]1:[cH]:[*]:[c]([*]):[cH]:[cH]:1	-0.294	50 out of 102

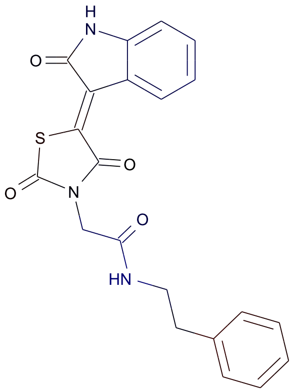

$C_{21}H_{17}N_3O_4S$
Molecular Weight: 407.44237
ALogP: 2.027
Rotatable Bonds: 5
Acceptors: 5
Donors: 2

Structural Similar Compounds

Name	5-NORBORNENE-2;3-DICARBOXYLIC ACID; 1;4;5;6;7;7-HEXACHLORO-	2-NAPHTHALENESULFONIC ACID;5-AMINO-6-ETHOXY-	PHENOL; 4;4'-SULFONYLDI-
Structure	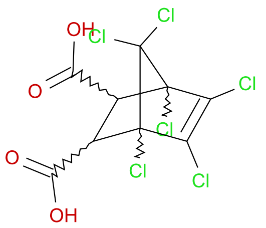	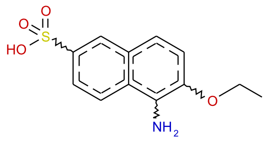	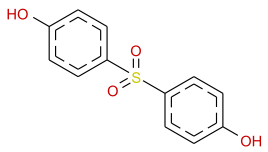
Actual Endpoint	Severe	Moderate	Moderate
Predicted Endpoint	Severe	Moderate	Moderate
Distance	0.643	0.754	0.794
Reference	28ZPAK-;92;72	28ZPAK-;191;72	BIOFX* 601-05501;74

Model Prediction

Prediction: Moderate
Probability: 0.557
Enrichment: 0.898
Bayesian Score: -3.1
Mahalanobis Distance: 10.1
Mahalanobis Distance p-value: 0.0339

Prediction: Positive if the Bayesian score is above the estimated best cutoff value from minimizing the false positive and false negative rate.
Probability: The esimated probability that the sample is in the positive category. This assumes that the Bayesian score follows a normal distribution and is different from the prediction using a cutoff.
Enrichment: An estimate of enrichment, that is, the increased likelihood (versus random) of this sample being in the category.
Bayesian Score: The standard Laplacian-modified Bayesian score.
Mahalanobis Distance: The Mahalanobis distance (MD) is the distance to the center of the training data. The larger the MD, the less trustworthy the prediction.
Mahalanobis Distance p-value: The p-value gives the fraction of training data with an MD greater than or equal to the one for the given sample, assuming normally distributed data. The smaller the p-value, the less trustworthy the prediciton. For highly non-normal X properties (e.g., fingerprints), the MD p-value is wildly inaccurate.

Model Applicability

Unknown features are fingerprint features in the query molecule, but not found or appearing too infrequently in the training set.

1. All properties and OPS components are within expected ranges.

Feature Contribution

Top features for positive contribution

Fingerprint	Bit/Smiles	Feature Structure	Score	Severe in training set
SCFP_12	-1640858361	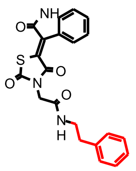 [*]CC[c]1:[cH]:[cH]:[cH]:[cH]:[cH]:1	0.376	4 out of 4

SCFP_12	-1272709286	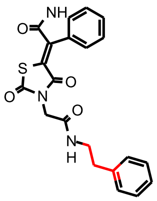 [*]CC[c](:[*]):[*]	0.231	24 out of 31
SCFP_12	2102703671	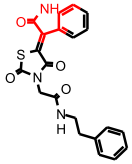 [*]=C1C(=O)N[c](:[cH]:[*]):[c]1:[*]	0.218	1 out of 1

Top Features for negative contribution

Fingerprint	Bit/Smiles	Feature Structure	Score	Severe in training set
SCFP_12	2005026407	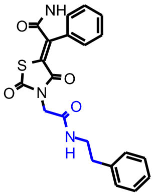 [*]CCNC(=O)C[*]	-0.796	0 out of 2
SCFP_12	1655488245	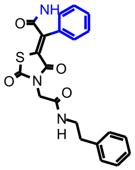 [*]1[*][c]2:[cH]:[cH]:[cH]:[cH]:[c]:2N1	-0.796	0 out of 2
SCFP_12	-587569116	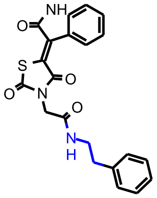 [*]CCN[*]	-0.619	11 out of 35

Sunitinib

TOPKAT_Ocular_Irritancy_Moderate_vs_Severe

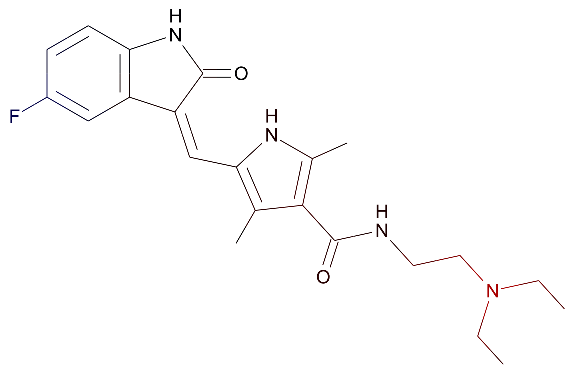

$C_{22}H_{27}FN_4O_2$
Molecular Weight: 398.47378
ALogP: 2.997
Rotatable Bonds: 7
Acceptors: 3
Donors: 3

Model Prediction

Prediction: Severe
Probability: 0.714
Enrichment: 1.15
Bayesian Score: 0.743
Mahalanobis Distance: 11.7
Mahalanobis Distance p-value: 7.58e-005

Prediction: Positive if the Bayesian score is above the estimated best cutoff value from minimizing the false positive and false negative rate.
Probability: The esimated probability that the sample is in the positive category. This assumes that the Bayesian score follows a normal distribution and is different from the prediction using a cutoff.
Enrichment: An estimate of enrichment, that is, the increased likelihood (versus random) of this sample being in the category.
Bayesian Score: The standard Laplacian-modified Bayesian score.
Mahalanobis Distance: The Mahalanobis distance (MD) is the distance to the center of the training data. The larger the MD, the less trustworthy the prediction.
Mahalanobis Distance p-value: The p-value gives the fraction of training data with an MD greater than or equal to the one for the given sample, assuming normally distributed data. The smaller the p-value, the less trustworthy the prediciton. For highly non-normal X properties (e.g., fingerprints), the MD p-value is wildly inaccurate.

Structural Similar Compounds

Name	METHANE;TRIS(4-AMINOPHENYL)-	5-NORBORNENE-2;3-DICARBOXYLIC ACID; 1;4;5;6;7;7-HEXACHLORO-	FLUORENE-9;9-(BIS)PROPYLAMINE
Structure	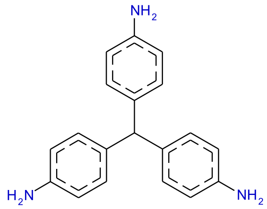	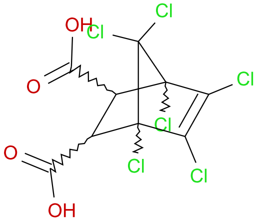	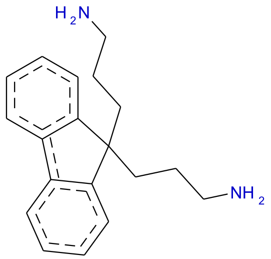
Actual Endpoint	Moderate	Severe	Severe
Predicted Endpoint	Moderate	Severe	Severe
Distance	0.726	0.793	0.804
Reference	28ZPAK-;73;72	28ZPAK-;92;72	IHFCAY 6;1;67

Model Applicability

Unknown features are fingerprint features in the query molecule, but not found or appearing too infrequently in the training set.

1. All properties and OPS components are within expected ranges.

Feature Contribution

Top features for positive contribution				
Fingerprint	Bit/Smiles	Feature Structure	Score	Severe in training set
SCFP_12	1725890097	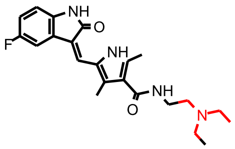 [*]CN(C[*])CC	0.449	12 out of 12

SCFP_12	2088704928	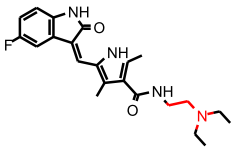 [*]CCN([*])[*]	0.342	110 out of 128
SCFP_12	5	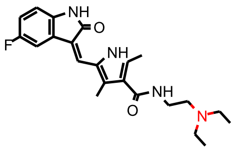 [*]N([*])[*]	0.337	129 out of 151
**Top Features for negative contribution**				
**Fingerprint**	**Bit/Smiles**	**Feature Structure**	**Score**	**Severe in training set**
SCFP_12	-587569116	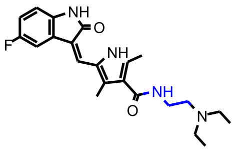 [*]CCN[*]	-0.619	11 out of 35
SCFP_12	-1381307546	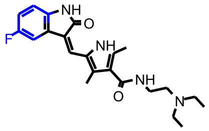 [*][c]1:[*]:[cH]:[c](F):[cH]:[cH]:1	-0.345	1 out of 3
SCFP_12	-1794884847	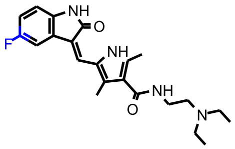 [*]:[c](:[*])F	-0.345	1 out of 3

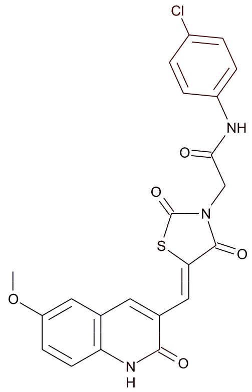

$C_{22}H_{16}ClN_3O_5S$
Molecular Weight: 469.89753
ALogP: 2.815
Rotatable Bonds: 5
Acceptors: 6
Donors: 2

Model Prediction

Prediction: Irritant
Probability: 1
Enrichment: 1.18
Bayesian Score: 2.29
Mahalanobis Distance: 11.7
Mahalanobis Distance p-value: 0.000301

Prediction: Positive if the Bayesian score is above the estimated best cutoff value from minimizing the false positive and false negative rate.
Probability: The esimated probability that the sample is in the positive category. This assumes that the Bayesian score follows a normal distribution and is different from the prediction using a cutoff.
Enrichment: An estimate of enrichment, that is, the increased likelihood (versus random) of this sample being in the category.
Bayesian Score: The standard Laplacian-modified Bayesian score.
Mahalanobis Distance: The Mahalanobis distance (MD) is the distance to the center of the training data. The larger the MD, the less trustworthy the prediction.
Mahalanobis Distance p-value: The p-value gives the fraction of training data with an MD greater than or equal to the one for the given sample, assuming normally distributed data. The smaller the p-value, the less trustworthy the prediciton. For highly non-normal X properties (e.g., fingerprints), the MD p-value is wildly inaccurate.

Structural Similar Compounds

Name	5-NORBORNENE-2;3-DICARBOXYLIC ACID; 1;4;5;6;7;7-HEXACHLORO-	ANTHRAQUINONE; 1-AMINO-4-HYDROXY-2-PHENOXY-	4;4'-DIAMINO-1;1'-DIANTHRIMIDE
Structure	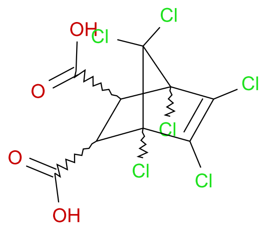	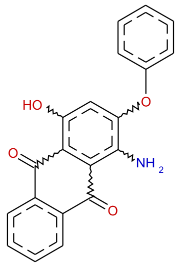	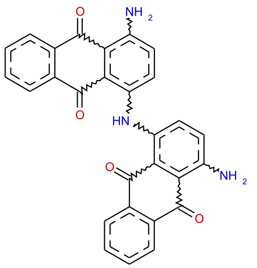
Actual Endpoint	Irritant	Irritant	Irritant
Predicted Endpoint	Irritant	Irritant	Irritant
Distance	0.731	0.732	0.746
Reference	28ZPAK-;92;72	28ZPAK 239;72	28ZPAK-;125;72

Model Applicability

Unknown features are fingerprint features in the query molecule, but not found or appearing too infrequently in the training set.

1. All properties and OPS components are within expected ranges.

Feature Contribution

Top features for positive contribution				
Fingerprint	Bit/Smiles	Feature Structure	Score	Irritant in training set
FCFP_12	-1508180856	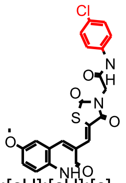 [*][c]1:[cH]:[cH]:[c](Cl):[cH]:[cH]:1	0.2	17 out of 17

FCFP_12	1175665944	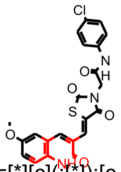 [*]C1=[*][c](:[*]):[c](NC1=O):[cH]:[*]	0.198	14 out of 14
FCFP_12	-745491832	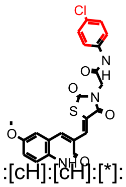 Cl[c]1:[cH]:[cH]:[*]:[cH]:[cH]:1	0.177	32 out of 33
**Top Features for negative contribution**				
**Fingerprint**	**Bit/Smiles**	**Feature Structure**	**Score**	**Irritant in training set**
FCFP_12	-1060187936	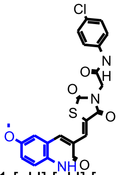 [*]N[c]1:[cH]:[cH]:[c](OC):[cH]:[c]:1[*]	-0.344	2 out of 4
FCFP_12	-1757681964	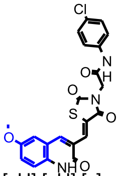 [*][c]1:[cH]:[cH]:[c](OC):[cH]:[c]:1C=[*]	-0.268	1 out of 2
FCFP_12	451371068	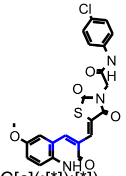 [*]C(=C[c](:[*]):[*])[*]	-0.167	6 out of 9

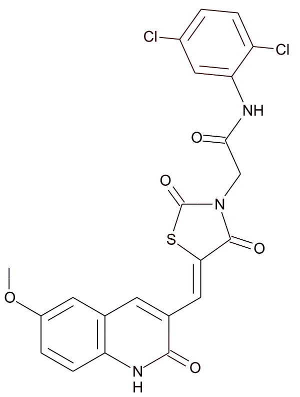

$C_{22}H_{15}Cl_2N_3O_5S$
Molecular Weight: 504.3426
ALogP: 3.479
Rotatable Bonds: 5
Acceptors: 6
Donors: 2

Model Prediction

Prediction: Irritant
Probability: 1
Enrichment: 1.18
Bayesian Score: 1.99
Mahalanobis Distance: 11.9
Mahalanobis Distance p-value: 0.000107

Prediction: Positive if the Bayesian score is above the estimated best cutoff value from minimizing the false positive and false negative rate.
Probability: The esimated probability that the sample is in the positive category. This assumes that the Bayesian score follows a normal distribution and is different from the prediction using a cutoff.
Enrichment: An estimate of enrichment, that is, the increased likelihood (versus random) of this sample being in the category.
Bayesian Score: The standard Laplacian-modified Bayesian score.
Mahalanobis Distance: The Mahalanobis distance (MD) is the distance to the center of the training data. The larger the MD, the less trustworthy the prediction.
Mahalanobis Distance p-value: The p-value gives the fraction of training data with an MD greater than or equal to the one for the given sample, assuming normally distributed data. The smaller the p-value, the less trustworthy the prediciton. For highly non-normal X properties (e.g., fingerprints), the MD p-value is wildly inaccurate.

Structural Similar Compounds

Name	4;4'-DIAMINO-1;1'-DIANTHRIMIDE	5-NORBORNENE-2;3-DICARBOXYLIC ACID; 1;4;5;6;7;7-HEXACHLORO-	BENZANILIDE;2';2'''-DITHIOBIS-
Structure	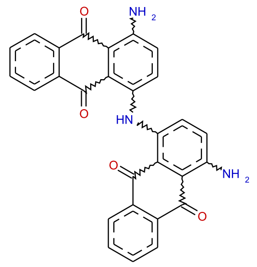	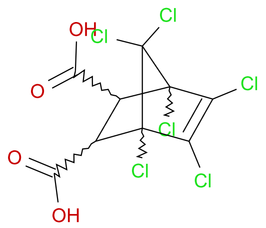	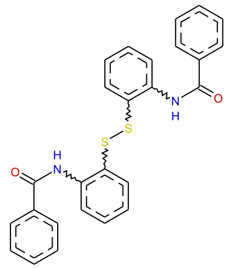
Actual Endpoint	Irritant	Irritant	Non-Irritant
Predicted Endpoint	Irritant	Irritant	Non-Irritant
Distance	0.744	0.774	0.775
Reference	28ZPAK-;125;72	28ZPAK-;92;72	28ZPAK-;173;72

Model Applicability

Unknown features are fingerprint features in the query molecule, but not found or appearing too infrequently in the training set.

1. All properties and OPS components are within expected ranges.

Feature Contribution

Top features for positive contribution				
**Fingerprint**	**Bit/Smiles**	**Feature Structure**	**Score**	**Irritant in training set**
FCFP_12	1175665944	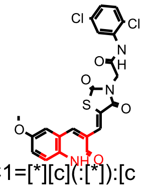 [*]C1=[*][c](:[*]):[c](NC1=O):[cH]:[*]	0.198	14 out of 14

FCFP_12	-770645118	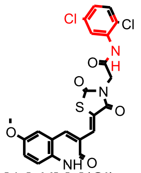 [*]N[c]1:[cH]:[c](Cl):[cH]:[*]:[c]:1[*]	0.184	7 out of 7
FCFP_12	-745491832	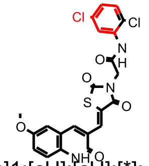 Cl[c]1:[cH]:[cH]:[*]:[cH]:[cH]:1	0.177	32 out of 33
**Top Features for negative contribution**				
**Fingerprint**	**Bit/Smiles**	**Feature Structure**	**Score**	**Irritant in training set**
FCFP_12	-1060187936	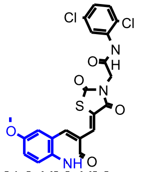 [*]N[c]1:[cH]:[cH]:[c](OC):[cH]:[c]:1[*]	-0.344	2 out of 4
FCFP_12	-1757681964	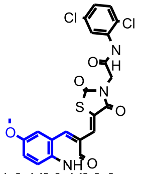 [*][c]1:[cH]:[cH]:[c](OC):[cH]:[c]:1C=[*]	-0.268	1 out of 2
FCFP_12	451371068	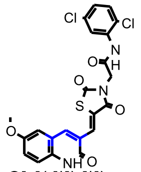 [*]C(=C[c](:[*]):[*])[*]	-0.167	6 out of 9

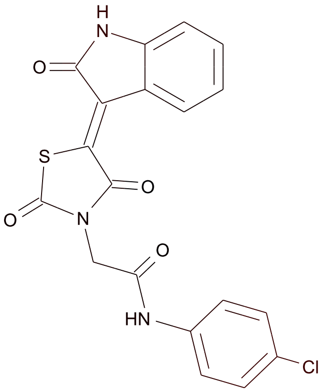

$C_{19}H_{12}ClN_3O_4S$
Molecular Weight: 413.83428
ALogP: 2.363
Rotatable Bonds: 3
Acceptors: 5
Donors: 2

Model Prediction

Prediction: Irritant
Probability: 1
Enrichment: 1.18
Bayesian Score: 2.71
Mahalanobis Distance: 8.42
Mahalanobis Distance p-value: 0.8

Prediction: Positive if the Bayesian score is above the estimated best cutoff value from minimizing the false positive and false negative rate.
Probability: The esimated probability that the sample is in the positive category. This assumes that the Bayesian score follows a normal distribution and is different from the prediction using a cutoff.
Enrichment: An estimate of enrichment, that is, the increased likelihood (versus random) of this sample being in the category.
Bayesian Score: The standard Laplacian-modified Bayesian score.
Mahalanobis Distance: The Mahalanobis distance (MD) is the distance to the center of the training data. The larger the MD, the less trustworthy the prediction.
Mahalanobis Distance p-value: The p-value gives the fraction of training data with an MD greater than or equal to the one for the given sample, assuming normally distributed data. The smaller the p-value, the less trustworthy the prediciton. For highly non-normal X properties (e.g., fingerprints), the MD p-value is wildly inaccurate.

Structural Similar Compounds

Name	1-AMINO-4-BENZOYLAMINO-ANTHRAQUINONE	5-NORBORNENE-2;3-DICARBOXYLIC ACID; 1;4;5;6;7;7-HEXACHLORO-	ANTHRAQUINONE; 1-AMINO-4-HYDROXY-2-PHENOXY-
Structure	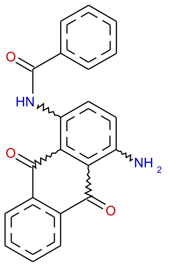	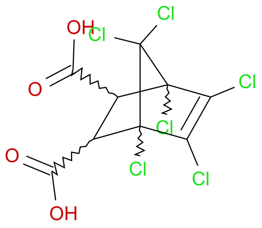	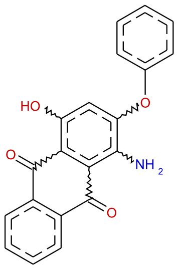
Actual Endpoint	Irritant	Irritant	Irritant
Predicted Endpoint	Irritant	Irritant	Irritant
Distance	0.621	0.628	0.635
Reference	28ZPAK-;124;72	28ZPAK-;92;72	28ZPAK 239;72

Model Applicability

Unknown features are fingerprint features in the query molecule, but not found or appearing too infrequently in the training set.

1. All properties and OPS components are within expected ranges.

Feature Contribution

Top features for positive contribution				
Fingerprint	Bit/Smiles	Feature Structure	Score	Irritant in training set
FCFP_12	-1508180856	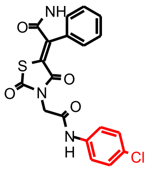 [*][c]1:[cH]:[cH]:[c](Cl):[cH]:[cH]:1	0.2	17 out of 17

FCFP_12	1175665944	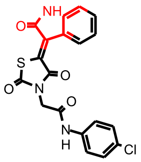 [*]C1=[*][c](:[*]):[c](NC1=O):[cH]:[*]	0.198	14 out of 14
FCFP_12	-745491832	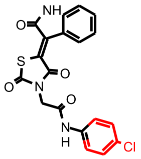 Cl[c]1:[cH]:[cH]:[*]:[cH]:[cH]:1	0.177	32 out of 33

Top Features for negative contribution

Fingerprint	Bit/Smiles	Feature Structure	Score	Irritant in training set
FCFP_12	-1698724694	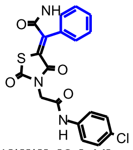 [*]=C1[*][*][c]2:[cH]:[cH]:[cH]:[cH]:[c]1:2	-0.0964	107 out of 146
FCFP_12	565998553	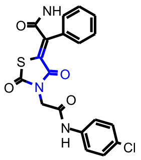 [*]N1[*][*]C(=[*])C1=O	-0.0662	198 out of 262
FCFP_12	-1678275541	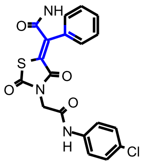 [*]C(=C1C(=[*])[*][*]:[c]1:[*])[*]	-0.0561	3 out of 4

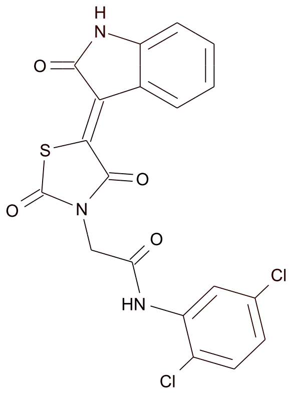

$C_{19}H_{11}Cl_2N_3O_4S$
Molecular Weight: 448.27933
ALogP: 3.028
Rotatable Bonds: 3
Acceptors: 5
Donors: 2

Model Prediction

Prediction: Irritant
Probability: 1
Enrichment: 1.18
Bayesian Score: 2.4
Mahalanobis Distance: 8.64
Mahalanobis Distance p-value: 0.699

Prediction: Positive if the Bayesian score is above the estimated best cutoff value from minimizing the false positive and false negative rate.
Probability: The esimated probability that the sample is in the positive category. This assumes that the Bayesian score follows a normal distribution and is different from the prediction using a cutoff.
Enrichment: An estimate of enrichment, that is, the increased likelihood (versus random) of this sample being in the category.
Bayesian Score: The standard Laplacian-modified Bayesian score.
Mahalanobis Distance: The Mahalanobis distance (MD) is the distance to the center of the training data. The larger the MD, the less trustworthy the prediction.
Mahalanobis Distance p-value: The p-value gives the fraction of training data with an MD greater than or equal to the one for the given sample, assuming normally distributed data. The smaller the p-value, the less trustworthy the prediciton. For highly non-normal X properties (e.g., fingerprints), the MD p-value is wildly inaccurate.

Structural Similar Compounds

Name	5-NORBORNENE-2;3-DICARBOXYLIC ACID; 1;4;5;6;7;7-HEXACHLORO-	1-AMINO-4-BENZOYLAMINO-ANTHRAQUINONE	ANTHRAQUINONE; 1-AMINO-4-HYDROXY-2-PHENOXY-
Structure	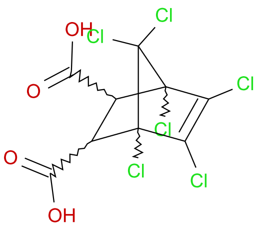	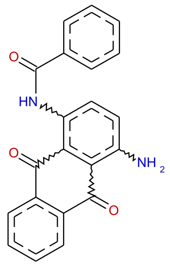	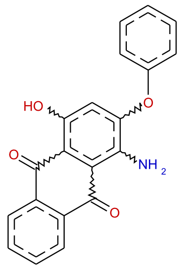
Actual Endpoint	Irritant	Irritant	Irritant
Predicted Endpoint	Irritant	Irritant	Irritant
Distance	0.646	0.652	0.660
Reference	28ZPAK-;92;72	28ZPAK-;124;72	28ZPAK 239;72

Model Applicability

Unknown features are fingerprint features in the query molecule, but not found or appearing too infrequently in the training set.

1. All properties and OPS components are within expected ranges.

Feature Contribution

Top features for positive contribution

Fingerprint	Bit/Smiles	Feature Structure	Score	Irritant in training set
FCFP_12	1175665944	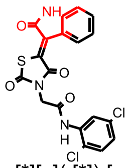 [*]C1=[*][c](:[*]):[c](NC1=O):[cH]:[*]	0.198	14 out of 14

FCFP_12	-770645118	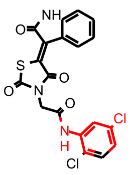 [*]N[c]1:[cH]:[c](Cl):[cH]:[*]:[c]:1[*]	0.184	7 out of 7
FCFP_12	-745491832	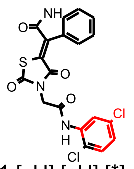 Cl[c]1:[cH]:[cH]:[*]:[cH]:[cH]:1	0.177	32 out of 33

Top Features for negative contribution

Fingerprint	Bit/Smiles	Feature Structure	Score	Irritant in training set
FCFP_12	-1698724694	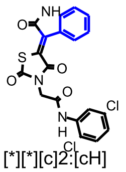 [*]=C1[*][*][c]2:[cH]:[cH]:[cH]:[cH]:[c]1:2	-0.0964	107 out of 146
FCFP_12	565998553	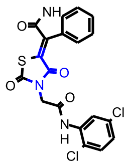 [*]N1[*][*]C(=[*])C1=O	-0.0662	198 out of 262
FCFP_12	-1678275541	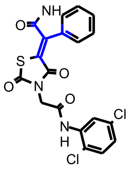 [*]C(=C1C(=[*])[*][*]:[c]1:[*])[*]	-0.0561	3 out of 4

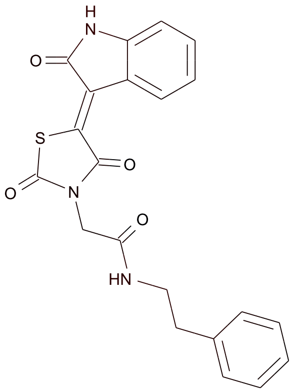

$C_{21}H_{17}N_3O_4S$
Molecular Weight: 407.44237
ALogP: 2.027
Rotatable Bonds: 5
Acceptors: 5
Donors: 2

Model Prediction

Prediction: Irritant
Probability: 1
Enrichment: 1.18
Bayesian Score: 2.12
Mahalanobis Distance: 8.94
Mahalanobis Distance p-value: 0.546

Prediction: Positive if the Bayesian score is above the estimated best cutoff value from minimizing the false positive and false negative rate.
Probability: The esimated probability that the sample is in the positive category. This assumes that the Bayesian score follows a normal distribution and is different from the prediction using a cutoff.
Enrichment: An estimate of enrichment, that is, the increased likelihood (versus random) of this sample being in the category.
Bayesian Score: The standard Laplacian-modified Bayesian score.
Mahalanobis Distance: The Mahalanobis distance (MD) is the distance to the center of the training data. The larger the MD, the less trustworthy the prediction.
Mahalanobis Distance p-value: The p-value gives the fraction of training data with an MD greater than or equal to the one for the given sample, assuming normally distributed data. The smaller the p-value, the less trustworthy the prediciton. For highly non-normal X properties (e.g., fingerprints), the MD p-value is wildly inaccurate.

Structural Similar Compounds

Name	1-AMINO-4-BENZOYLAMINO-ANTHRAQUINONE	5-NORBORNENE-2;3-DICARBOXYLIC ACID; 1;4;5;6;7;7-HEXACHLORO-	ANTHRAQUINONE; 1-AMINO-4-HYDROXY-2-PHENOXY-
Structure	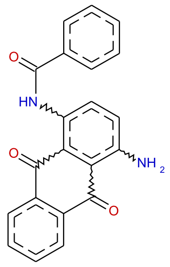	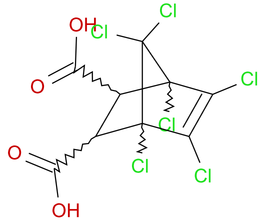	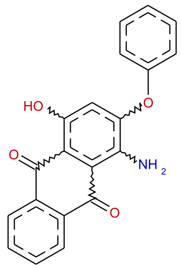
Actual Endpoint	Irritant	Irritant	Irritant
Predicted Endpoint	Irritant	Irritant	Irritant
Distance	0.629	0.641	0.653
Reference	28ZPAK-;124;72	28ZPAK-;92;72	28ZPAK 239;72

Model Applicability

Unknown features are fingerprint features in the query molecule, but not found or appearing too infrequently in the training set.

1. All properties and OPS components are within expected ranges.

Feature Contribution

Top features for positive contribution				
**Fingerprint**	**Bit/Smiles**	**Feature Structure**	**Score**	**Irritant in training set**
FCFP_12	1175665944	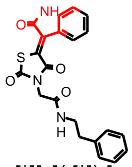 [*]C1=[*][c](:[*]):[c](NC1=O):[cH]:[*]	0.198	14 out of 14

FCFP_12	-885550502	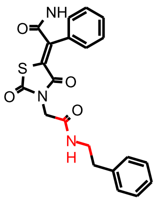 [*]CNC(=[*])[*]	0.18	64 out of 66
FCFP_12	436915834	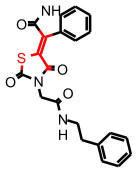 [*]\C=C\1/S[*][*]C1=[*]	0.167	4 out of 4

Top Features for negative contribution

Fingerprint	Bit/Smiles	Feature Structure	Score	Irritant in training set
FCFP_12	1981711554	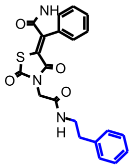 [*]CC[c]1:[cH]:[cH]:[cH]:[cH]:[cH]:1	-0.103	5 out of 7
FCFP_12	-1698724694	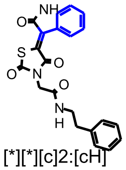 [*]=C1[*][*][c]2:[cH]:[cH]:[cH]:[cH]:[c]1:2	-0.0964	107 out of 146
FCFP_12	565998553	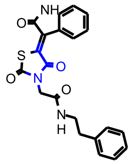 [*]N1[*][*]C(=[*])C1=O	-0.0662	198 out of 262

Sorafenib

TOPKAT_Ocular_Irritancy_None_vs_Irritant

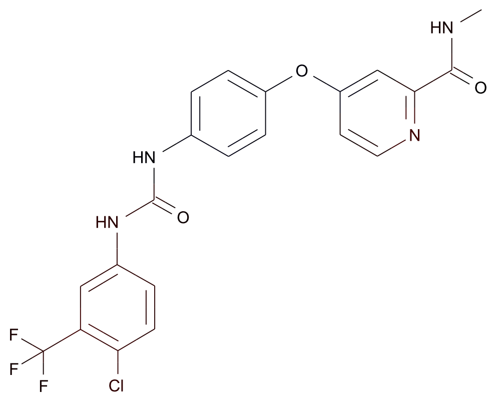

$C_{21}H_{16}ClF_3N_4O_3$
Molecular Weight: 464.82494
ALogP: 4.175
Rotatable Bonds: 6
Acceptors: 4
Donors: 3

Structural Similar Compounds

Name	BENZANILIDE;2';2'''-DITHIOBIS-	4;4'-DIAMINO-1;1'-DIANTHRIMIDE	5-NORBORNENE-2;3-DICARBOXYLIC ACID; 1;4;5;6;7;7-HEXACHLORO-
Structure	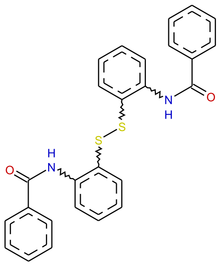	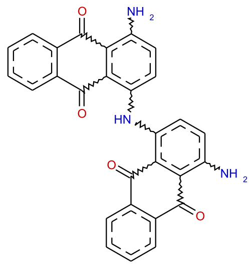	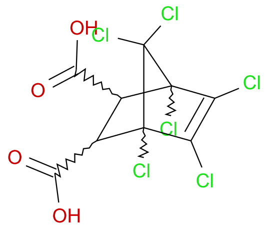
Actual Endpoint	Non-Irritant	Irritant	Irritant
Predicted Endpoint	Non-Irritant	Irritant	Irritant
Distance	0.743	0.791	0.801
Reference	28ZPAK-;173;72	28ZPAK-;125;72	28ZPAK-;92;72

Model Prediction

Prediction: Irritant
Probability: 1
Enrichment: 1.18
Bayesian Score: 3.04
Mahalanobis Distance: 6.28
Mahalanobis Distance p-value: 1

Prediction: Positive if the Bayesian score is above the estimated best cutoff value from minimizing the false positive and false negative rate.
Probability: The esimated probability that the sample is in the positive category. This assumes that the Bayesian score follows a normal distribution and is different from the prediction using a cutoff.
Enrichment: An estimate of enrichment, that is, the increased likelihood (versus random) of this sample being in the category.
Bayesian Score: The standard Laplacian-modified Bayesian score.
Mahalanobis Distance: The Mahalanobis distance (MD) is the distance to the center of the training data. The larger the MD, the less trustworthy the prediction.
Mahalanobis Distance p-value: The p-value gives the fraction of training data with an MD greater than or equal to the one for the given sample, assuming normally distributed data. The smaller the p-value, the less trustworthy the prediciton. For highly non-normal X properties (e.g., fingerprints), the MD p-value is wildly inaccurate.

Model Applicability

Unknown features are fingerprint features in the query molecule, but not found or appearing too infrequently in the training set.

1. All properties and OPS components are within expected ranges.

Feature Contribution

Top features for positive contribution				
**Fingerprint**	**Bit/Smiles**	**Feature Structure**	**Score**	**Irritant in training set**
FCFP_12	1747237384	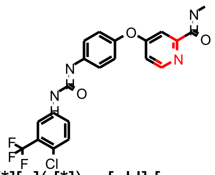 [*][c](:[*]):n:[cH]:[*]	0.208	44 out of 44

FCFP_12	-124655670	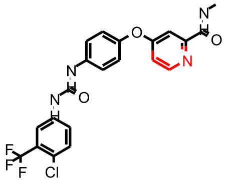 [*]:[cH]:[cH]:n:[*]	0.2	16 out of 16
FCFP_12	-1539132615	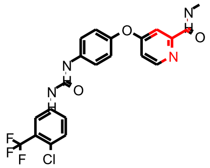 [*]C(=[*])[c](:[cH]:[*]):n:[*]	0.197	13 out of 13

Top Features for negative contribution

**Fingerprint**	**Bit/Smiles**	**Feature Structure**	**Score**	**Irritant in training set**
FCFP_12	-747629521	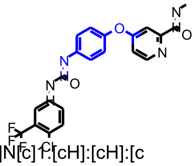 [*]N[c]1:[cH]:[cH]:[c](O[c](:[*]):[*]):[cH]:[cH]:1	-0.268	1 out of 2
FCFP_12	702861189	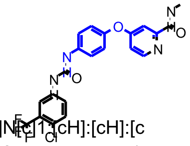 [*]N[c]1:[cH]:[cH]:[c](O[c]2:[cH]:[cH]:[*]:[c]([*]):[cH]:2):[cH]:[cH]:1	-0.268	1 out of 2
FCFP_12	-1597477966	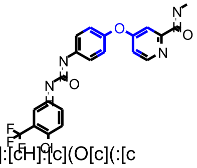 [*]:[cH]:[c](O[c](:[cH]:[*]):[cH]:[*]):[cH]:[*]	0	9 out of 11

Sunitinib

TOPKAT_Ocular_Irritancy_None_vs_Irritant

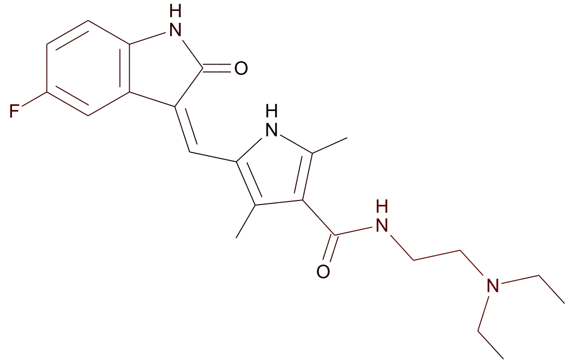

$C_{22}H_{27}FN_4O_2$

Molecular Weight: 398.47378

ALogP: 2.997

Rotatable Bonds: 7

Acceptors: 3

Donors: 3

Model Prediction

Prediction: Irritant

Probability: 1

Enrichment: 1.18

Bayesian Score: 4.17

Mahalanobis Distance: 8.04

Mahalanobis Distance p-value: 0.916

Prediction: Positive if the Bayesian score is above the estimated best cutoff value from minimizing the false positive and false negative rate.
Probability: The esimated probability that the sample is in the positive category. This assumes that the Bayesian score follows a normal distribution and is different from the prediction using a cutoff.
Enrichment: An estimate of enrichment, that is, the increased likelihood (versus random) of this sample being in the category.
Bayesian Score: The standard Laplacian-modified Bayesian score.
Mahalanobis Distance: The Mahalanobis distance (MD) is the distance to the center of the training data. The larger the MD, the less trustworthy the prediction.
Mahalanobis Distance p-value: The p-value gives the fraction of training data with an MD greater than or equal to the one for the given sample, assuming normally distributed data. The smaller the p-value, the less trustworthy the prediciton. For highly non-normal X properties (e.g., fingerprints), the MD p-value is wildly inaccurate.

Structural Similar Compounds

Name	METHANE;TRIS(4-AMINOPHENYL)-	ANTHRAQUINONE; 1-((2-HYDROXYETHYL)AMINO)-4-(METHYLAMINO)-	PHENOL;4-(3-CARBAZOLYLAMINO)-
Structure	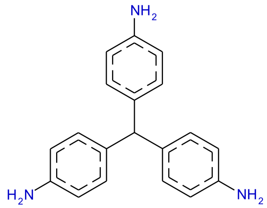	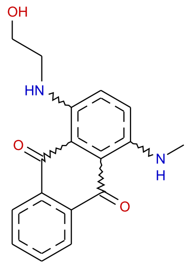	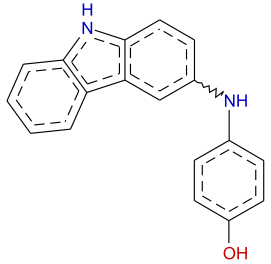
Actual Endpoint	Irritant	Irritant	Irritant
Predicted Endpoint	Irritant	Irritant	Irritant
Distance	0.704	0.757	0.773
Reference	28ZPAK-;73;72	28ZPAK 245;72	28ZPAK-;143;72

Model Applicability

Unknown features are fingerprint features in the query molecule, but not found or appearing too infrequently in the training set.

1. All properties and OPS components are within expected ranges.
2. Unknown FCFP_2 feature: 203707511: [*][c]1:[*]:[*]:[nH]:[c]:1C=[*]

Feature Contribution

Top features for positive contribution				
**Fingerprint**	**Bit/Smiles**	**Feature Structure**	**Score**	**Irritant in training set**
FCFP_12	1175665944	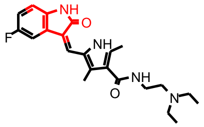 [*]C1=[*][c](:[*]):[c](NC1=O):[cH]:[*]	0.198	14 out of 14

FCFP_12	1851332093	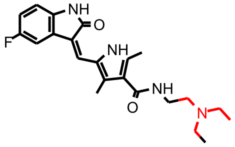<br>[*]CN(C[*])CC	0.195	12 out of 12
FCFP_12	-885550502	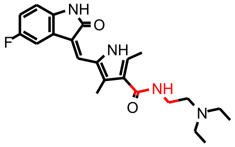<br>[*]CNC(=[*])[*]	0.18	64 out of 66

Top Features for negative contribution

Fingerprint	Bit/Smiles	Feature Structure	Score	Irritant in training set
FCFP_12	451371068	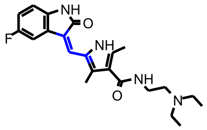<br>[*]C(=C[c](:[*]):[*])<br>[*]	-0.167	6 out of 9
FCFP_12	-1678275541	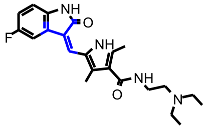<br>[*]C(=C1C(=[*])[*][*]<br>:[c]1:[*])[*]	-0.0561	3 out of 4
FCFP_12	0	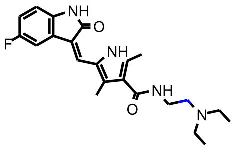<br>[*]C=[*]	0	1184 out of 1397

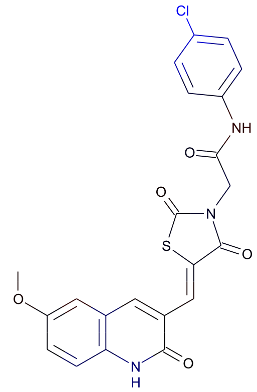

$C_{22}H_{16}ClN_3O_5S$
Molecular Weight: 469.89753
ALogP: 2.815
Rotatable Bonds: 5
Acceptors: 6
Donors: 2

Structural Similar Compounds

Name	Bicalutamide	Carbenicillin	Polythiazide
Structure	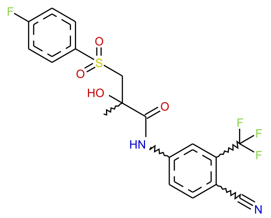	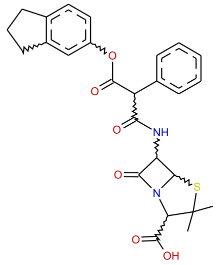	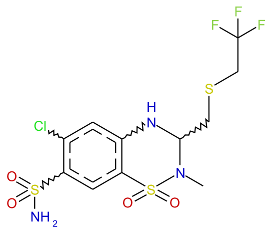
Actual Endpoint	Carcinogen	Non-Carcinogen	Non-Carcinogen
Predicted Endpoint	Carcinogen	Non-Carcinogen	Non-Carcinogen
Distance	0.599	0.641	0.644
Reference	US FDA (Centre for Drug Eval.& Res./Off. Testing & Res.) Sept. 1997	US FDA (Centre for Drug Eval.& Res./Off. Testing & Res.) Sept. 1997	US FDA (Centre for Drug Eval.& Res./Off. Testing & Res.) Sept. 1997

Model Prediction

Prediction: Non-Carcinogen
Probability: 0.21
Enrichment: 0.652
Bayesian Score: -6.08
Mahalanobis Distance: 11.2
Mahalanobis Distance p-value: 0.0452

Prediction: Positive if the Bayesian score is above the estimated best cutoff value from minimizing the false positive and false negative rate.
Probability: The esimated probability that the sample is in the positive category. This assumes that the Bayesian score follows a normal distribution and is different from the prediction using a cutoff.
Enrichment: An estimate of enrichment, that is, the increased likelihood (versus random) of this sample being in the category.
Bayesian Score: The standard Laplacian-modified Bayesian score.
Mahalanobis Distance: The Mahalanobis distance (MD) is the distance to the center of the training data. The larger the MD, the less trustworthy the prediction.
Mahalanobis Distance p-value: The p-value gives the fraction of training data with an MD greater than or equal to the one for the given sample, assuming normally distributed data. The smaller the p-value, the less trustworthy the prediciton. For highly non-normal X properties (e.g., fingerprints), the MD p-value is wildly inaccurate.

Model Applicability

Unknown features are fingerprint features in the query molecule, but not found or appearing too infrequently in the training set.

1. All properties and OPS components are within expected ranges.
2. Unknown ECFP_2 feature: 2131425032: [*]\C=C(\C=[*])/C(=[*])[*]
3. Unknown ECFP_2 feature: 1182722866: [*]C(=CC(=[*])[*])[*]
4. Unknown ECFP_2 feature: 1000552169: [*]\C=C\1/S[*][*]C1=[*]
5. Unknown ECFP_2 feature: 190445529: [*]N1[*][*]SC1=O

Feature Contribution

Top features for positive contribution				
**Fingerprint**	**Bit/Smiles**	**Feature Structure**	**Score**	**Carcinogen in training set**
ECFP_12	-177077903	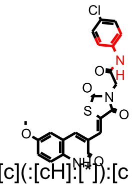 [*]N[c](:[cH]:[*]):[cH]:[*]	0.529	6 out of 10

ECFP_12	-1236483485	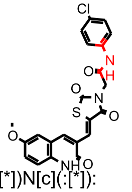 [*]C(=[*])N[c](:[*]):[*]	0.46	9 out of 17
ECFP_12	888054369	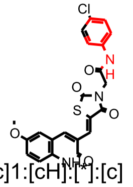 [*]N[c]1:[cH]:[*]:[c]([*]):[cH]:[cH]:1	0.454	5 out of 9
**Top Features for negative contribution**				
**Fingerprint**	**Bit/Smiles**	**Feature Structure**	**Score**	**Carcinogen in training set**
ECFP_12	1335833675	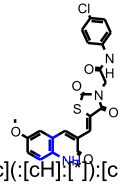 [*]N[c](:[cH]:[*]):[c]([*]):[*]	-1.25	0 out of 8
ECFP_12	99947387	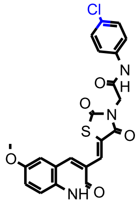 [*]:[c](:[*])Cl	-0.817	8 out of 62
ECFP_12	1854732111	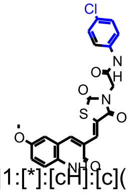 [*][c]1:[*]:[cH]:[c](Cl):[cH]:[cH]:1	-0.816	4 out of 33

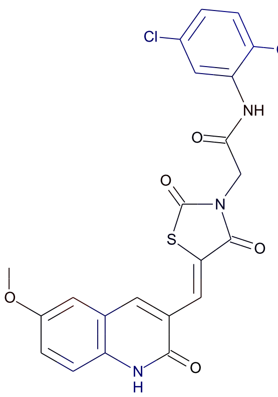

$C_{22}H_{15}Cl_2N_3O_5S$
Molecular Weight: 504.3426
ALogP: 3.479
Rotatable Bonds: 5
Acceptors: 6
Donors: 2

Model Prediction

Prediction: Non-Carcinogen
Probability: 0.206
Enrichment: 0.639
Bayesian Score: -6.52
Mahalanobis Distance: 11.4
Mahalanobis Distance p-value: 0.0271

Prediction: Positive if the Bayesian score is above the estimated best cutoff value from minimizing the false positive and false negative rate.
Probability: The esimated probability that the sample is in the positive category. This assumes that the Bayesian score follows a normal distribution and is different from the prediction using a cutoff.
Enrichment: An estimate of enrichment, that is, the increased likelihood (versus random) of this sample being in the category.
Bayesian Score: The standard Laplacian-modified Bayesian score.
Mahalanobis Distance: The Mahalanobis distance (MD) is the distance to the center of the training data. The larger the MD, the less trustworthy the prediction.
Mahalanobis Distance p-value: The p-value gives the fraction of training data with an MD greater than or equal to the one for the given sample, assuming normally distributed data. The smaller the p-value, the less trustworthy the prediciton. For highly non-normal X properties (e.g., fingerprints), the MD p-value is wildly inaccurate.

Structural Similar Compounds

Name	Carbenicillin	Bicalutamide	Glimepride
Structure	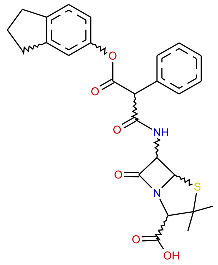	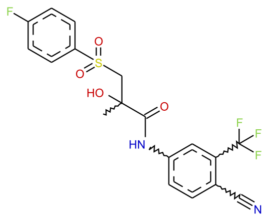	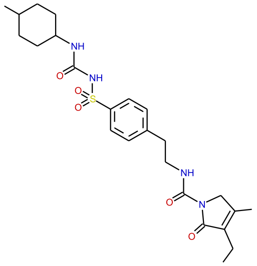
Actual Endpoint	Non-Carcinogen	Carcinogen	Non-Carcinogen
Predicted Endpoint	Non-Carcinogen	Carcinogen	Non-Carcinogen
Distance	0.620	0.651	0.687
Reference	US FDA (Centre for Drug Eval.& Res./Off. Testing & Res.) Sept. 1997	US FDA (Centre for Drug Eval.& Res./Off. Testing & Res.) Sept. 1997	US FDA (Centre for Drug Eval.& Res./Off. Testing & Res.) Sept. 1997

Model Applicability

Unknown features are fingerprint features in the query molecule, but not found or appearing too infrequently in the training set.

1. All properties and OPS components are within expected ranges.
2. Unknown ECFP_2 feature: 2131425032: [*]\C=C(\C=[*])/C(=[*])[*]
3. Unknown ECFP_2 feature: 1182722866: [*]C(=CC(=[*])[*])[*]
4. Unknown ECFP_2 feature: 1000552169: [*]\C=C\1/S[*][*]C1=[*]
5. Unknown ECFP_2 feature: 190445529: [*]N1[*][*]SC1=O

Feature Contribution

Top features for positive contribution				
**Fingerprint**	**Bit/Smiles**	**Feature Structure**	**Score**	**Carcinogen in training set**
ECFP_12	-1236483485	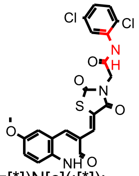 [*]C(=[*])N[c](:[*]):[*]	0.46	9 out of 17

ECFP_12	-1925046727	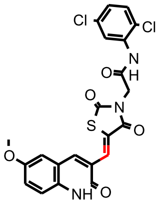 [*]C=[*]	0.407	16 out of 33
ECFP_12	1336666212	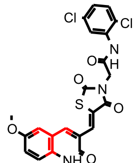 [*][c](:[*]):[c](C=[*]):[cH]:[*]	0.288	2 out of 4

Top Features for negative contribution

Fingerprint	Bit/Smiles	Feature Structure	Score	Carcinogen in training set
ECFP_12	1335833675	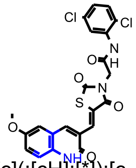 [*]N[c](:[cH]:[*]):[c]([*]):[*]	-1.25	0 out of 8
ECFP_12	1335691903	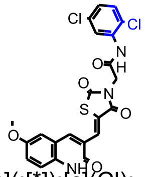 [*][c](:[*]):[c](Cl): [cH]:[*]	-1.11	2 out of 26
ECFP_12	99947387	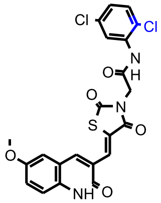 [*]:[c](:[*])Cl	-0.817	8 out of 62

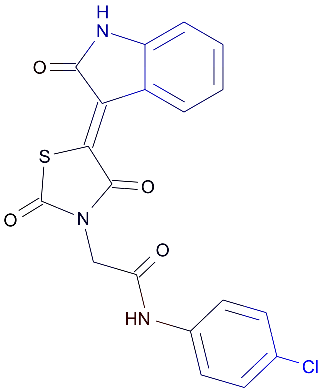

$C_{19}H_{12}ClN_3O_4S$
Molecular Weight: 413.83428
ALogP: 2.363
Rotatable Bonds: 3
Acceptors: 5
Donors: 2

Model Prediction

Prediction: Non-Carcinogen
Probability: 0.187
Enrichment: 0.581
Bayesian Score: -9.02
Mahalanobis Distance: 8.83
Mahalanobis Distance p-value: 0.872

Prediction: Positive if the Bayesian score is above the estimated best cutoff value from minimizing the false positive and false negative rate.
Probability: The esimated probability that the sample is in the positive category. This assumes that the Bayesian score follows a normal distribution and is different from the prediction using a cutoff.
Enrichment: An estimate of enrichment, that is, the increased likelihood (versus random) of this sample being in the category.
Bayesian Score: The standard Laplacian-modified Bayesian score.
Mahalanobis Distance: The Mahalanobis distance (MD) is the distance to the center of the training data. The larger the MD, the less trustworthy the prediction.
Mahalanobis Distance p-value: The p-value gives the fraction of training data with an MD greater than or equal to the one for the given sample, assuming normally distributed data. The smaller the p-value, the less trustworthy the prediciton. For highly non-normal X properties (e.g., fingerprints), the MD p-value is wildly inaccurate.

Structural Similar Compounds

Name	Indapamide	Metolazone	Doxefazepam
Structure	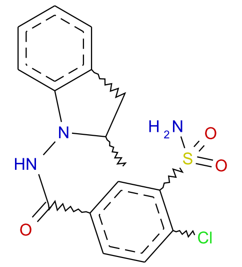	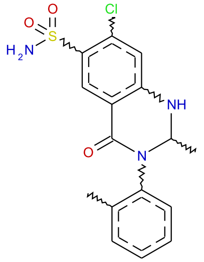	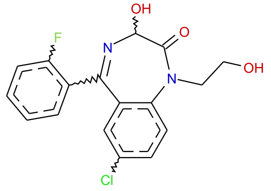
Actual Endpoint	Non-Carcinogen	Non-Carcinogen	Carcinogen
Predicted Endpoint	Non-Carcinogen	Non-Carcinogen	Carcinogen
Distance	0.594	0.601	0.651
Reference	US FDA (Centre for Drug Eval.& Res./Off. Testing & Res.) Sept. 1997	US FDA (Centre for Drug Eval.& Res./Off. Testing & Res.) Sept. 1997	US FDA (Centre for Drug Eval.& Res./Off. Testing & Res.) Sept. 1997

Model Applicability

Unknown features are fingerprint features in the query molecule, but not found or appearing too infrequently in the training set.

1. All properties and OPS components are within expected ranges.
2. Unknown ECFP_2 feature: -631778390: [*]C(=C1S[*][*]C1=[*])[*]
3. Unknown ECFP_2 feature: 190445529: [*]N1[*][*]SC1=O

Feature Contribution

Top features for positive contribution

Fingerprint	Bit/Smiles	Feature Structure	Score	Carcinogen in training set
ECFP_12	-177077903	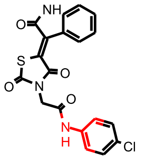 [*]N[c](:[cH]:[*]):[cH]:[*]	0.529	6 out of 10

ECFP_12	-1236483485	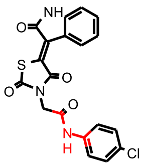 [*]C(=[*])N[c](:[*]):[*]	0.46	9 out of 17
ECFP_12	888054369	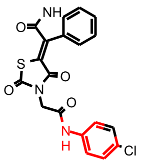 [*]N[c]1:[cH]:[*]:[c]([*]):[cH]:[cH]:1	0.454	5 out of 9
**Top Features for negative contribution**				
**Fingerprint**	**Bit/Smiles**	**Feature Structure**	**Score**	**Carcinogen in training set**
ECFP_12	1335833675	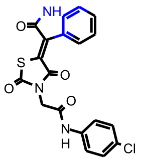 [*]N[c](:[cH]:[*]):[c]([*]):[*]	-1.25	0 out of 8
ECFP_12	99947387	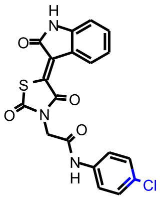 [*]:[c](:[*])Cl	-0.817	8 out of 62
ECFP_12	1854732111	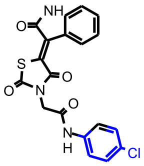 [*][c]1:[*]:[cH]:[c](Cl):[cH]:[cH]:1	-0.816	4 out of 33

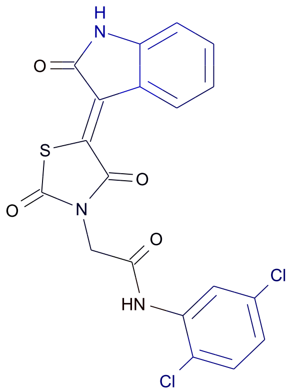

$C_{19}H_{11}Cl_2N_3O_4S$
Molecular Weight: 448.27933
ALogP: 3.028
Rotatable Bonds: 3
Acceptors: 5
Donors: 2

Model Prediction

Prediction: Non-Carcinogen
Probability: 0.183
Enrichment: 0.568
Bayesian Score: -9.76
Mahalanobis Distance: 8.95
Mahalanobis Distance p-value: 0.836

Prediction: Positive if the Bayesian score is above the estimated best cutoff value from minimizing the false positive and false negative rate.
Probability: The esimated probability that the sample is in the positive category. This assumes that the Bayesian score follows a normal distribution and is different from the prediction using a cutoff.
Enrichment: An estimate of enrichment, that is, the increased likelihood (versus random) of this sample being in the category.
Bayesian Score: The standard Laplacian-modified Bayesian score.
Mahalanobis Distance: The Mahalanobis distance (MD) is the distance to the center of the training data. The larger the MD, the less trustworthy the prediction.
Mahalanobis Distance p-value: The p-value gives the fraction of training data with an MD greater than or equal to the one for the given sample, assuming normally distributed data. The smaller the p-value, the less trustworthy the prediciton. For highly non-normal X properties (e.g., fingerprints), the MD p-value is wildly inaccurate.

Structural Similar Compounds

Name	Indapamide	Metolazone	Bicalutamide
Structure	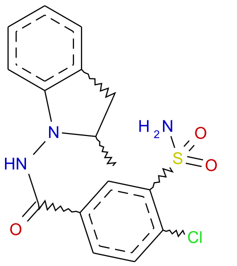	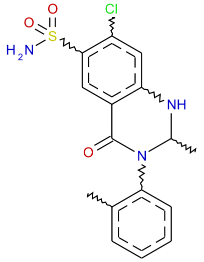	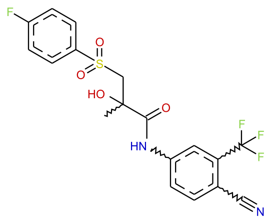
Actual Endpoint	Non-Carcinogen	Non-Carcinogen	Carcinogen
Predicted Endpoint	Non-Carcinogen	Non-Carcinogen	Carcinogen
Distance	0.629	0.641	0.662
Reference	US FDA (Centre for Drug Eval.& Res./Off. Testing & Res.) Sept. 1997	US FDA (Centre for Drug Eval.& Res./Off. Testing & Res.) Sept. 1997	US FDA (Centre for Drug Eval.& Res./Off. Testing & Res.) Sept. 1997

Model Applicability

Unknown features are fingerprint features in the query molecule, but not found or appearing too infrequently in the training set.

1. All properties and OPS components are within expected ranges.
2. Unknown ECFP_2 feature: -631778390: [*]C(=C1S[*][*]C1=[*])[*]
3. Unknown ECFP_2 feature: 190445529: [*]N1[*][*]SC1=O

Feature Contribution

Top features for positive contribution

Fingerprint	Bit/Smiles	Feature Structure	Score	Carcinogen in training set
ECFP_12	-1236483485	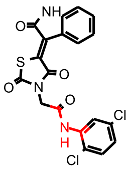 [*]C(=[*])N[c](:[*]):[*]	0.46	9 out of 17

ECFP_12	2106656448	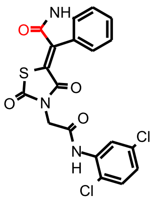 [*]C(=O)[*]	0.141	30 out of 83
ECFP_12	1335108269	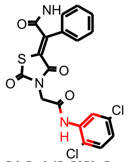 [*]N[c](:[cH]:[*]):[c]([*]):[*]	0.119	4 out of 11

Top Features for negative contribution

Fingerprint	Bit/Smiles	Feature Structure	Score	Carcinogen in training set
ECFP_12	1335833675	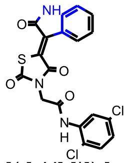 [*]N[c](:[cH]:[*]):[c]([*]):[*]	-1.25	0 out of 8
ECFP_12	1335691903	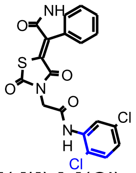 [*][c](:[*]):[c](Cl): [cH]:[*]	-1.11	2 out of 26
ECFP_12	99947387	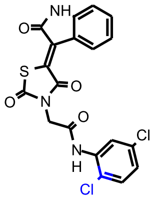 [*]:[c](:[*])Cl	-0.817	8 out of 62

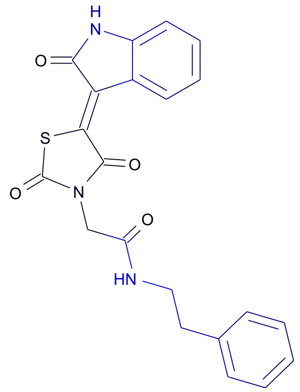

$C_{21}H_{17}N_3O_4S$
Molecular Weight: 407.44237
ALogP: 2.027
Rotatable Bonds: 5
Acceptors: 5
Donors: 2

Model Prediction

Prediction: Non-Carcinogen
Probability: 0.177
Enrichment: 0.551
Bayesian Score: -10.9
Mahalanobis Distance: 9.75
Mahalanobis Distance p-value: 0.486

Prediction: Positive if the Bayesian score is above the estimated best cutoff value from minimizing the false positive and false negative rate.
Probability: The esimated probability that the sample is in the positive category. This assumes that the Bayesian score follows a normal distribution and is different from the prediction using a cutoff.
Enrichment: An estimate of enrichment, that is, the increased likelihood (versus random) of this sample being in the category.
Bayesian Score: The standard Laplacian-modified Bayesian score.
Mahalanobis Distance: The Mahalanobis distance (MD) is the distance to the center of the training data. The larger the MD, the less trustworthy the prediction.
Mahalanobis Distance p-value: The p-value gives the fraction of training data with an MD greater than or equal to the one for the given sample, assuming normally distributed data. The smaller the p-value, the less trustworthy the prediciton. For highly non-normal X properties (e.g., fingerprints), the MD p-value is wildly inaccurate.

Structural Similar Compounds

Name	Bicalutamide	Acetohexamide	Penicillin
Structure	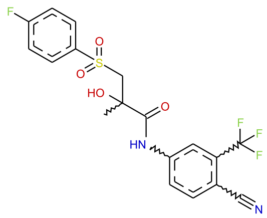	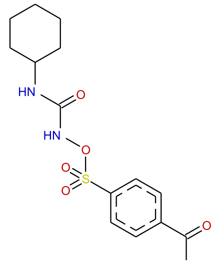	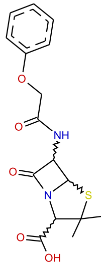
Actual Endpoint	Carcinogen	Non-Carcinogen	Non-Carcinogen
Predicted Endpoint	Carcinogen	Non-Carcinogen	Non-Carcinogen
Distance	0.598	0.601	0.629
Reference	US FDA (Centre for Drug Eval.& Res./Off. Testing & Res.) Sept. 1997	US FDA (Centre for Drug Eval.& Res./Off. Testing & Res.) Sept. 1997	US FDA (Centre for Drug Eval.& Res./Off. Testing & Res.) Sept. 1997

Model Applicability

Unknown features are fingerprint features in the query molecule, but not found or appearing too infrequently in the training set.

1. All properties and OPS components are within expected ranges.
2. Unknown ECFP_2 feature: -631778390: [*]C(=C1S[*][*]C1=[*])[*]
3. Unknown ECFP_2 feature: 190445529: [*]N1[*][*]SC1=O

Feature Contribution

Top features for positive contribution

Fingerprint	Bit/Smiles	Feature Structure	Score	Carcinogen in training set
ECFP_12	-1650219925	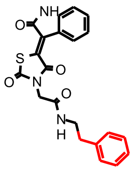 [*]C[c]1:[cH]:[cH]:[cH]:[cH]:[cH]:1	0.208	6 out of 15

ECFP_12	1095683433	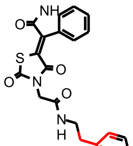 [*]C[c]1:[cH]:[*]:[cH]:[cH]:[cH]:1	0.154	6 out of 16
ECFP_12	2106656448	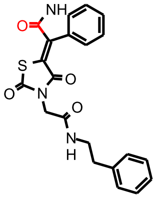 [*]C(=O)[*]	0.141	30 out of 83

Top Features for negative contribution

Fingerprint	Bit/Smiles	Feature Structure	Score	Carcinogen in training set
ECFP_12	1335833675	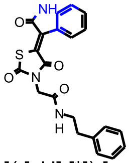 [*]N[c](:[cH]:[*]):[c]([*]):[*]	-1.25	0 out of 8
ECFP_12	497523368	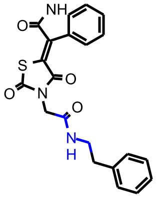 [*]CNC(=[*])[*]	-0.989	1 out of 14
ECFP_12	-104952638	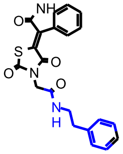 [*]C(=[*])NCC[c]1:[cH]:[cH]:[*]:[cH]:[cH] :1	-0.811	0 out of 4

Sorafenib

TOPKAT_Rat_Female_FDA_None_vs_Carcinogen

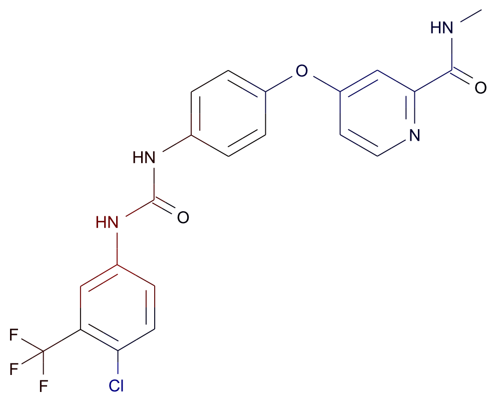

$C_{21}H_{16}ClF_3N_4O_3$
Molecular Weight: 464.82494
ALogP: 4.175
Rotatable Bonds: 6
Acceptors: 4
Donors: 3

Model Prediction

Prediction: Non-Carcinogen
Probability: 0.236
Enrichment: 0.734
Bayesian Score: -3.76
Mahalanobis Distance: 12.2
Mahalanobis Distance p-value: 0.00229

Prediction: Positive if the Bayesian score is above the estimated best cutoff value from minimizing the false positive and false negative rate.
Probability: The esimated probability that the sample is in the positive category. This assumes that the Bayesian score follows a normal distribution and is different from the prediction using a cutoff.
Enrichment: An estimate of enrichment, that is, the increased likelihood (versus random) of this sample being in the category.
Bayesian Score: The standard Laplacian-modified Bayesian score.
Mahalanobis Distance: The Mahalanobis distance (MD) is the distance to the center of the training data. The larger the MD, the less trustworthy the prediction.
Mahalanobis Distance p-value: The p-value gives the fraction of training data with an MD greater than or equal to the one for the given sample, assuming normally distributed data. The smaller the p-value, the less trustworthy the prediciton. For highly non-normal X properties (e.g., fingerprints), the MD p-value is wildly inaccurate.

Structural Similar Compounds

Name	Glimepride	Glyburide	Fluvastatin
Structure	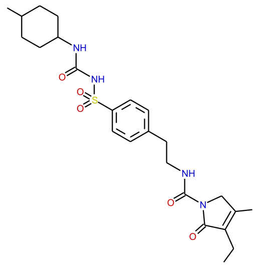	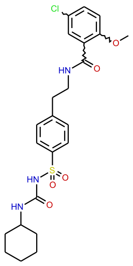	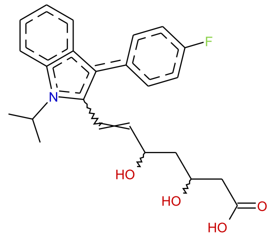
Actual Endpoint	Non-Carcinogen	Non-Carcinogen	Non-Carcinogen
Predicted Endpoint	Non-Carcinogen	Non-Carcinogen	Non-Carcinogen
Distance	0.620	0.635	0.635
Reference	US FDA (Centre for Drug Eval.& Res./Off. Testing & Res.) Sept. 1997	US FDA (Centre for Drug Eval.& Res./Off. Testing & Res.) Sept. 1997	US FDA (Centre for Drug Eval.& Res./Off. Testing & Res.) Sept. 1997

Model Applicability

Unknown features are fingerprint features in the query molecule, but not found or appearing too infrequently in the training set.

1. All properties and OPS components are within expected ranges.

Feature Contribution

Top features for positive contribution

Fingerprint	Bit/Smiles	Feature Structure	Score	Carcinogen in training set
ECFP_12	-970385855	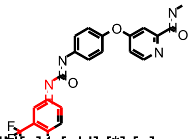 [*]N[c]1:[cH]:[*]:[c]([*]):[c](:[cH]:1)C([*])([*])[*]	0.613	2 out of 2

ECFP_12	-177077903	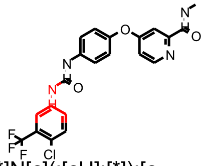 [*]N[c](:[cH]:[*]):[cH]:[*]	0.529	6 out of 10
ECFP_12	-1236483485	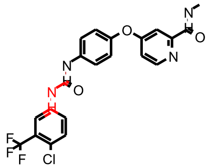 [*]C(=[*])N[c](:[*]):[*]	0.46	9 out of 17
**Top Features for negative contribution**				
**Fingerprint**	**Bit/Smiles**	**Feature Structure**	**Score**	**Carcinogen in training set**
ECFP_12	1335691903	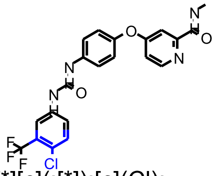 [*][c](:[*]):[c](Cl):[cH]:[*]	-1.11	2 out of 26
ECFP_12	99947387	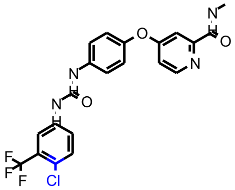 [*]:[c](:[*])Cl	-0.817	8 out of 62
ECFP_12	1413420509	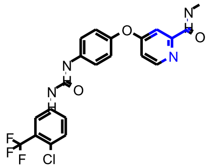 [*]C(=[*])[c](:[cH]:[*]):n:[*]	-0.661	0 out of 3

Sunitinib

TOPKAT_Rat_Female_FDA_None_vs_Carcinogen

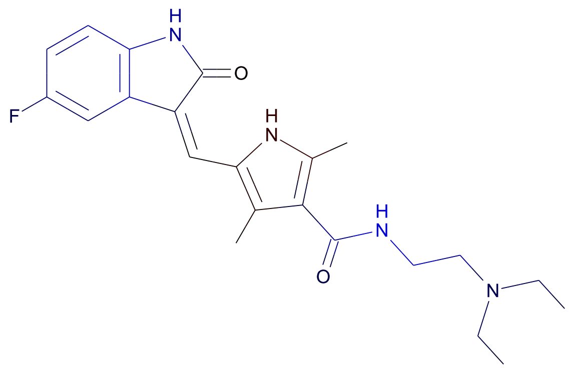

$C_{22}H_{27}FN_4O_2$

Molecular Weight: 398.47378

ALogP: 2.997

Rotatable Bonds: 7

Acceptors: 3

Donors: 3

Model Prediction

Prediction: Non-Carcinogen

Probability: 0.188

Enrichment: 0.584

Bayesian Score: -8.84

Mahalanobis Distance: 11.3

Mahalanobis Distance p-value: 0.0371

Prediction: Positive if the Bayesian score is above the estimated best cutoff value from minimizing the false positive and false negative rate.
Probability: The esimated probability that the sample is in the positive category. This assumes that the Bayesian score follows a normal distribution and is different from the prediction using a cutoff.
Enrichment: An estimate of enrichment, that is, the increased likelihood (versus random) of this sample being in the category.
Bayesian Score: The standard Laplacian-modified Bayesian score.
Mahalanobis Distance: The Mahalanobis distance (MD) is the distance to the center of the training data. The larger the MD, the less trustworthy the prediction.
Mahalanobis Distance p-value: The p-value gives the fraction of training data with an MD greater than or equal to the one for the given sample, assuming normally distributed data. The smaller the p-value, the less trustworthy the prediciton. For highly non-normal X properties (e.g., fingerprints), the MD p-value is wildly inaccurate.

Structural Similar Compounds

Name	Fluvastatin	Metoclopramide	Torsemide
Structure	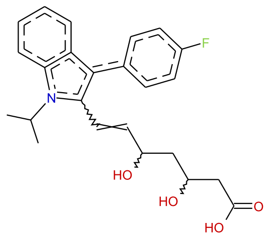	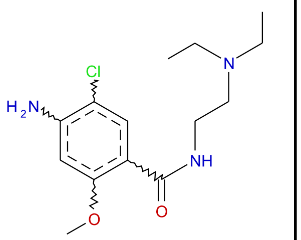	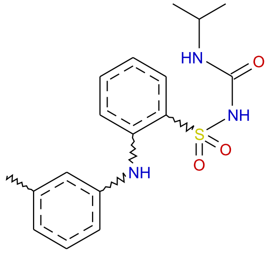
Actual Endpoint	Non-Carcinogen	Non-Carcinogen	Carcinogen
Predicted Endpoint	Non-Carcinogen	Non-Carcinogen	Carcinogen
Distance	0.629	0.681	0.684
Reference	US FDA (Centre for Drug Eval.& Res./Off. Testing & Res.) Sept. 1997	US FDA (Centre for Drug Eval.& Res./Off. Testing & Res.) Sept. 1997	US FDA (Centre for Drug Eval.& Res./Off. Testing & Res.) Sept. 1997

Model Applicability

Unknown features are fingerprint features in the query molecule, but not found or appearing too infrequently in the training set.

1. All properties and OPS components are within expected ranges.
2. Unknown ECFP_2 feature: 980271847: [*][c]1:[*]:[*]:[nH]:[c]:1C=[*]
3. Unknown ECFP_2 feature: 1791989338: [*][c]1:[*]:[*]:[nH]:[c]:1C
4. Unknown ECFP_2 feature: 1182722866: [*]C(=CC(=[*])[*])[*]

Feature Contribution

Top features for positive contribution

Fingerprint	Bit/Smiles	Feature Structure	Score	Carcinogen in training set
ECFP_12	558201926	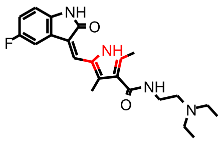 [*][c]1:[*]:[*]:[c]([*]):[nH]:1	0.539	5 out of 8

ECFP_12	-1658273810	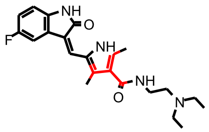 [*]C(=[*])[c]1:[c]([*]):[*]:[*]:[c]:1[*]	0.421	1 out of 1
ECFP_12	-152683720	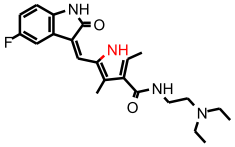 [*]:[nH]:[*]	0.412	9 out of 18
**Top Features for negative contribution**				
**Fingerprint**	**Bit/Smiles**	**Feature Structure**	**Score**	**Carcinogen in training set**
ECFP_12	1335833675	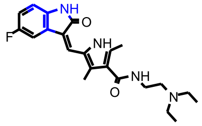 [*]N[c](:[cH]:[*]):[c]([*]):[*]	-1.25	0 out of 8
ECFP_12	497523368	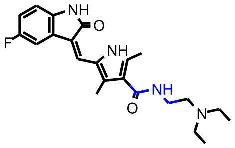 [*]CNC(=[*])[*]	-0.989	1 out of 14
ECFP_12	2083628577	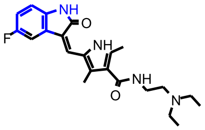 [*]N[c]1:[cH]:[cH]:[c]([*]):[*]:[c]:1[*]	-0.811	0 out of 4

TOPKAT_Rat_Male_FDA_None_vs_Carcinogen

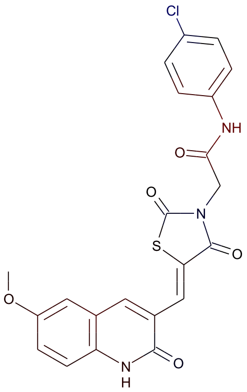

$C_{22}H_{16}ClN_3O_5S$
Molecular Weight: 469.89753
ALogP: 2.815
Rotatable Bonds: 5
Acceptors: 6
Donors: 2

Model Prediction

Prediction: Non-Carcinogen
Probability: 0.341
Enrichment: 1.02
Bayesian Score: -0.539
Mahalanobis Distance: 12
Mahalanobis Distance p-value: 0.0309

Prediction: Positive if the Bayesian score is above the estimated best cutoff value from minimizing the false positive and false negative rate.
Probability: The esimated probability that the sample is in the positive category. This assumes that the Bayesian score follows a normal distribution and is different from the prediction using a cutoff.
Enrichment: An estimate of enrichment, that is, the increased likelihood (versus random) of this sample being in the category.
Bayesian Score: The standard Laplacian-modified Bayesian score.
Mahalanobis Distance: The Mahalanobis distance (MD) is the distance to the center of the training data. The larger the MD, the less trustworthy the prediction.
Mahalanobis Distance p-value: The p-value gives the fraction of training data with an MD greater than or equal to the one for the given sample, assuming normally distributed data. The smaller the p-value, the less trustworthy the prediciton. For highly non-normal X properties (e.g., fingerprints), the MD p-value is wildly inaccurate.

Structural Similar Compounds

Name	Bicalutamide	Carbenicillin	Polythiazide
Structure	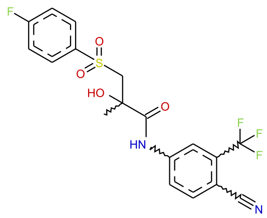	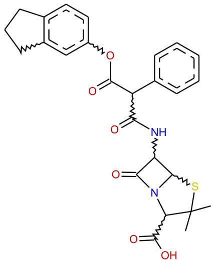	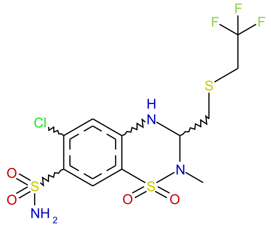
Actual Endpoint	Carcinogen	Non-Carcinogen	Non-Carcinogen
Predicted Endpoint	Carcinogen	Non-Carcinogen	Non-Carcinogen
Distance	0.572	0.627	0.632
Reference	US FDA (Centre for Drug Eval.& Res./Off. Testing & Res.) Sept. 1997	US FDA (Centre for Drug Eval.& Res./Off. Testing & Res.) Sept. 1997	US FDA (Centre for Drug Eval.& Res./Off. Testing & Res.) Sept. 1997

Model Applicability

Unknown features are fingerprint features in the query molecule, but not found or appearing too infrequently in the training set.

1. OPS PC7 out of range. Value: 5.8284. Training min, max, SD, explained variance: -5.7463, 5.4773, 2.041, 0.0388.

Feature Contribution

Top features for positive contribution				
**Fingerprint**	**Bit/Smiles**	**Feature Structure**	**Score**	**Carcinogen in training set**
SCFP_6	-347048986	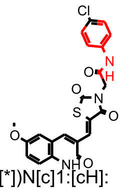 [*]C(=[*])N[c]1:[cH]:[cH]:[*]:[cH]:[cH]:1	0.615	5 out of 7

SCFP_6	814408713	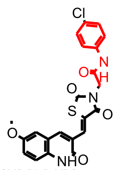 [*]CC(=O)N[c]1:[cH]:[cH]:[c]([*]):[cH]:[c H]:1	0.603	2 out of 2
SCFP_6	2097618059	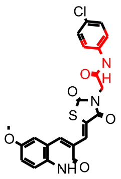 [*]CC(=O)N[c](:[cH]:[*]):[cH]:[*]	0.437	7 out of 13
**Top Features for negative contribution**				
**Fingerprint**	**Bit/Smiles**	**Feature Structure**	**Score**	**Carcinogen in training set**
SCFP_6	399659969	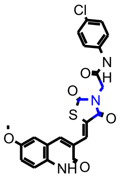 [*]CN1C(=[*])[*][*]C1 =[*]	-0.578	1 out of 8
SCFP_6	2102703671	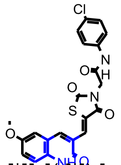 [*]C1=[*][c](:[*]):[c](NC1=O):[cH]:[*]	-0.496	0 out of 2
SCFP_6	1905487031	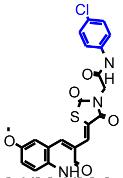 [*][c]1:[cH]:[cH]:[c] (Cl):[cH]:[cH]:1	-0.48	2 out of 12

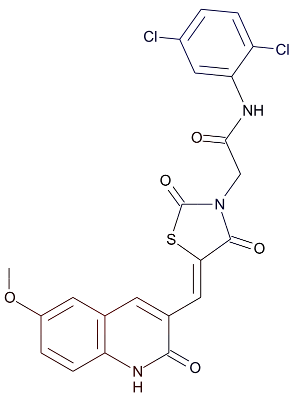

$C_{22}H_{15}Cl_2N_3O_5S$
Molecular Weight: 504.3426
ALogP: 3.479
Rotatable Bonds: 5
Acceptors: 6
Donors: 2

Model Prediction

Prediction: Non-Carcinogen
Probability: 0.297
Enrichment: 0.89
Bayesian Score: -2.23
Mahalanobis Distance: 12.1
Mahalanobis Distance p-value: 0.0246

Prediction: Positive if the Bayesian score is above the estimated best cutoff value from minimizing the false positive and false negative rate.
Probability: The esimated probability that the sample is in the positive category. This assumes that the Bayesian score follows a normal distribution and is different from the prediction using a cutoff.
Enrichment: An estimate of enrichment, that is, the increased likelihood (versus random) of this sample being in the category.
Bayesian Score: The standard Laplacian-modified Bayesian score.
Mahalanobis Distance: The Mahalanobis distance (MD) is the distance to the center of the training data. The larger the MD, the less trustworthy the prediction.
Mahalanobis Distance p-value: The p-value gives the fraction of training data with an MD greater than or equal to the one for the given sample, assuming normally distributed data. The smaller the p-value, the less trustworthy the prediciton. For highly non-normal X properties (e.g., fingerprints), the MD p-value is wildly inaccurate.

Structural Similar Compounds

Name	Carbenicillin	Bicalutamide	Moricizine
Structure	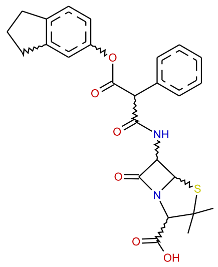	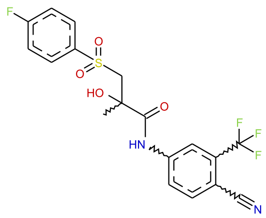	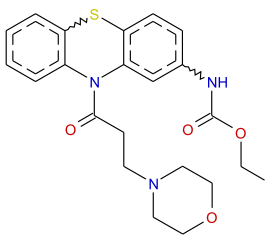
Actual Endpoint	Non-Carcinogen	Carcinogen	Carcinogen
Predicted Endpoint	Non-Carcinogen	Carcinogen	Carcinogen
Distance	0.607	0.625	0.672
Reference	US FDA (Centre for Drug Eval.& Res./Off. Testing & Res.) Sept. 1997	US FDA (Centre for Drug Eval.& Res./Off. Testing & Res.) Sept. 1997	US FDA (Centre for Drug Eval.& Res./Off. Testing & Res.) Sept. 1997

Model Applicability

Unknown features are fingerprint features in the query molecule, but not found or appearing too infrequently in the training set.

1. OPS PC7 out of range. Value: 5.8042. Training min, max, SD, explained variance: -5.7463, 5.4773, 2.041, 0.0388.

Feature Contribution

Top features for positive contribution

Fingerprint	Bit/Smiles	Feature Structure	Score	Carcinogen in training set
SCFP_6	2097618059	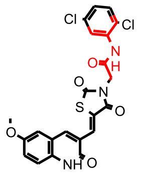 [*]CC(=O)N[c](:[cH]:[*]):[cH]:[*]	0.437	7 out of 13

SCFP_6	-1971137145	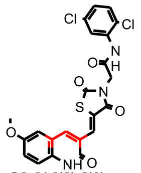<br>[*]C(=C[c](:[*]):[*])<br>[*]	0.434	5 out of 9
SCFP_6	392579710	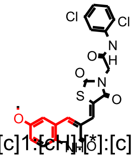<br>[*]O[c]1:[cH]:[*]:[c]<br>([*]):[c](C=[*]):[cH<br>]:1	0.425	2 out of 3
**Top Features for negative contribution**				
**Fingerprint**	**Bit/Smiles**	**Feature Structure**	**Score**	**Carcinogen in training set**
SCFP_6	399659969	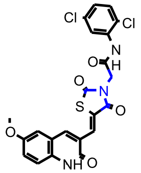<br>[*]CN1C(=[*])[*][*]C1<br>=[*]	-0.578	1 out of 8
SCFP_6	2102703671	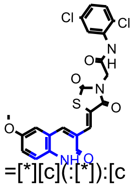<br>[*]C1=[*][c](:[*]):[c<br>](NC1=O):[cH]:[*]	-0.496	0 out of 2
SCFP_6	-345817764	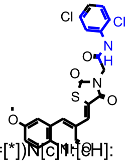<br>[*]C(=[*])N[c]1:[cH]:<br>[c]([*]):[*]:[cH]:[c<br>]:1Cl	-0.496	0 out of 2

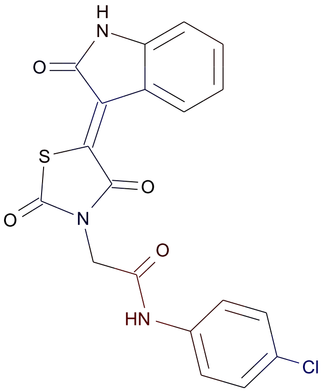

$C_{19}H_{12}ClN_3O_4S$

Molecular Weight: 413.83428

ALogP: 2.363

Rotatable Bonds: 3

Acceptors: 5

Donors: 2

Structural Similar Compounds

Name	Indapamide	Metolazone	Doxefazepam
Structure	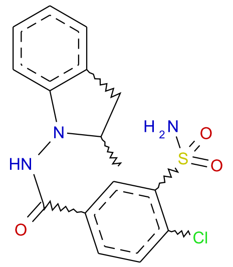	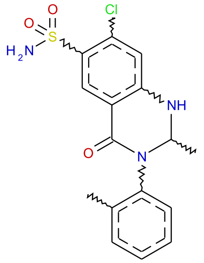	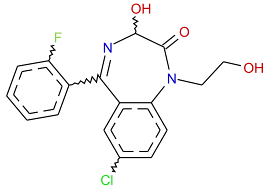
Actual Endpoint	Non-Carcinogen	Non-Carcinogen	Carcinogen
Predicted Endpoint	Non-Carcinogen	Non-Carcinogen	Carcinogen
Distance	0.564	0.571	0.605
Reference	US FDA (Centre for Drug Eval.& Res./Off. Testing & Res.) Sept. 1997	US FDA (Centre for Drug Eval.& Res./Off. Testing & Res.) Sept. 1997	US FDA (Centre for Drug Eval.& Res./Off. Testing & Res.) Sept. 1997

Model Prediction

Prediction: Non-Carcinogen

Probability: 0.312

Enrichment: 0.935

Bayesian Score: -1.62

Mahalanobis Distance: 9.5

Mahalanobis Distance p-value: 0.845

Prediction: Positive if the Bayesian score is above the estimated best cutoff value from minimizing the false positive and false negative rate.
Probability: The esimated probability that the sample is in the positive category. This assumes that the Bayesian score follows a normal distribution and is different from the prediction using a cutoff.
Enrichment: An estimate of enrichment, that is, the increased likelihood (versus random) of this sample being in the category.
Bayesian Score: The standard Laplacian-modified Bayesian score.
Mahalanobis Distance: The Mahalanobis distance (MD) is the distance to the center of the training data. The larger the MD, the less trustworthy the prediction.
Mahalanobis Distance p-value: The p-value gives the fraction of training data with an MD greater than or equal to the one for the given sample, assuming normally distributed data. The smaller the p-value, the less trustworthy the prediciton. For highly non-normal X properties (e.g., fingerprints), the MD p-value is wildly inaccurate.

Model Applicability

Unknown features are fingerprint features in the query molecule, but not found or appearing too infrequently in the training set.

1. All properties and OPS components are within expected ranges.

Feature Contribution

Top features for positive contribution				
**Fingerprint**	**Bit/Smiles**	**Feature Structure**	**Score**	**Carcinogen in training set**
SCFP_6	-347048986	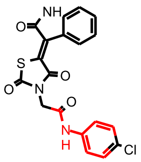 [*]C(=[*])N[c]1:[cH]:[cH]:[*]:[cH]:[cH]:1	0.615	5 out of 7

SCFP_6	814408713	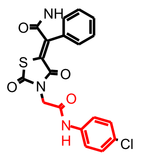 [*]CC(=O)N[c]1:[cH]:[cH]:[c]([*]):[cH]:[cH]:1	0.603	2 out of 2
SCFP_6	2097618059	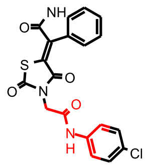 [*]CC(=O)N[c](:[cH]:[*]):[cH]:[*]	0.437	7 out of 13

Top Features for negative contribution

**Fingerprint**	**Bit/Smiles**	**Feature Structure**	**Score**	**Carcinogen in training set**
SCFP_6	1798334293	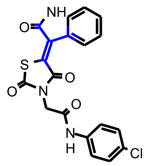 [*]C(=C1C(=[*])[*][*]:[c]1:[*])[*]	-0.674	0 out of 3
SCFP_6	399659969	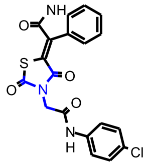 [*]CN1C(=[*])[*][*]C1=[*]	-0.578	1 out of 8
SCFP_6	2102703671	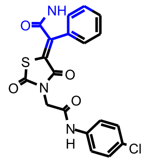 [*]C1=[*][c](:[*]):[c](NC1=O):[cH]:[*]	-0.496	0 out of 2

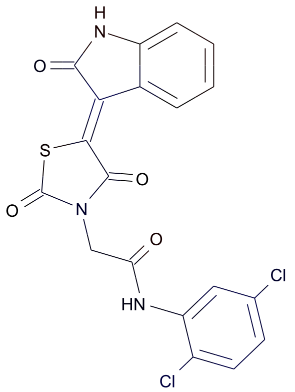

$C_{19}H_{11}Cl_2N_3O_4S$
Molecular Weight: 448.27933
ALogP: 3.028
Rotatable Bonds: 3
Acceptors: 5
Donors: 2

Model Prediction

Prediction: Non-Carcinogen
Probability: 0.28
Enrichment: 0.838
Bayesian Score: -2.98
Mahalanobis Distance: 9.53
Mahalanobis Distance p-value: 0.835

Prediction: Positive if the Bayesian score is above the estimated best cutoff value from minimizing the false positive and false negative rate.
Probability: The esimated probability that the sample is in the positive category. This assumes that the Bayesian score follows a normal distribution and is different from the prediction using a cutoff.
Enrichment: An estimate of enrichment, that is, the increased likelihood (versus random) of this sample being in the category.
Bayesian Score: The standard Laplacian-modified Bayesian score.
Mahalanobis Distance: The Mahalanobis distance (MD) is the distance to the center of the training data. The larger the MD, the less trustworthy the prediction.
Mahalanobis Distance p-value: The p-value gives the fraction of training data with an MD greater than or equal to the one for the given sample, assuming normally distributed data. The smaller the p-value, the less trustworthy the prediciton. For highly non-normal X properties (e.g., fingerprints), the MD p-value is wildly inaccurate.

Structural Similar Compounds

Name	Indapamide	Metolazone	Bicalutamide
Structure	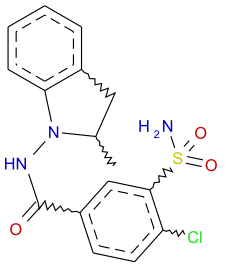	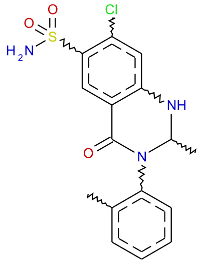	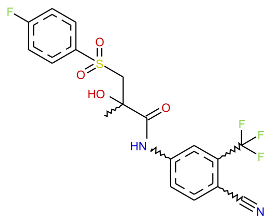
Actual Endpoint	Non-Carcinogen	Non-Carcinogen	Carcinogen
Predicted Endpoint	Non-Carcinogen	Non-Carcinogen	Carcinogen
Distance	0.611	0.620	0.637
Reference	US FDA (Centre for Drug Eval.& Res./Off. Testing & Res.) Sept. 1997	US FDA (Centre for Drug Eval.& Res./Off. Testing & Res.) Sept. 1997	US FDA (Centre for Drug Eval.& Res./Off. Testing & Res.) Sept. 1997

Model Applicability

Unknown features are fingerprint features in the query molecule, but not found or appearing too infrequently in the training set.

1. All properties and OPS components are within expected ranges.

Feature Contribution

Top features for positive contribution

Fingerprint	Bit/Smiles	Feature Structure	Score	Carcinogen in training set
SCFP_6	2097618059	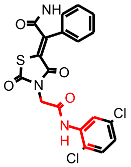 [*]CC(=O)N[c](:[cH]:[*]):[cH]:[*]	0.437	7 out of 13

SCFP_6	1655488245	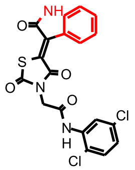 [*]1[*][c]2:[cH]:[cH]:[cH]:[cH]:[c]:2N1	0.252	4 out of 9
SCFP_6	-1375926917	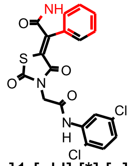 [*]N[c]1:[cH]:[*]:[c]([*]):[cH]:[cH]:1	0.251	11 out of 26
**Top Features for negative contribution**				
**Fingerprint**	**Bit/Smiles**	**Feature Structure**	**Score**	**Carcinogen in training set**
SCFP_6	1798334293	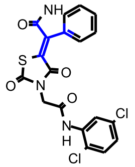 [*]C(=C1C(=[*])[*][*]:[c]1:[*])[*]	-0.674	0 out of 3
SCFP_6	399659969	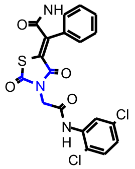 [*]CN1C(=[*])[*][*]C1=[*]	-0.578	1 out of 8
SCFP_6	2102703671	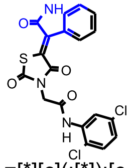 [*]C1=[*][c](:[*]):[c](NC1=O):[cH]:[*]	-0.496	0 out of 2

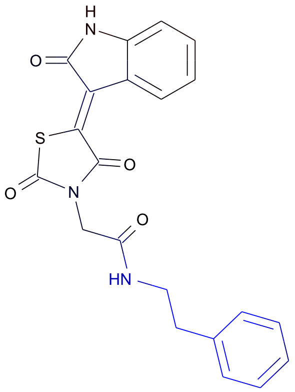

$C_{21}H_{17}N_3O_4S$

Molecular Weight: 407.44237

ALogP: 2.027

Rotatable Bonds: 5

Acceptors: 5

Donors: 2

Model Prediction

Prediction: Non-Carcinogen

Probability: 0.186

Enrichment: 0.556

Bayesian Score: -8.37

Mahalanobis Distance: 13.3

Mahalanobis Distance p-value: 0.000591

Prediction: Positive if the Bayesian score is above the estimated best cutoff value from minimizing the false positive and false negative rate.
Probability: The esimated probability that the sample is in the positive category. This assumes that the Bayesian score follows a normal distribution and is different from the prediction using a cutoff.
Enrichment: An estimate of enrichment, that is, the increased likelihood (versus random) of this sample being in the category.
Bayesian Score: The standard Laplacian-modified Bayesian score.
Mahalanobis Distance: The Mahalanobis distance (MD) is the distance to the center of the training data. The larger the MD, the less trustworthy the prediction.
Mahalanobis Distance p-value: The p-value gives the fraction of training data with an MD greater than or equal to the one for the given sample, assuming normally distributed data. The smaller the p-value, the less trustworthy the prediciton. For highly non-normal X properties (e.g., fingerprints), the MD p-value is wildly inaccurate.

Structural Similar Compounds

Name	Bicalutamide	Acetohexamide	Indapamide
Structure	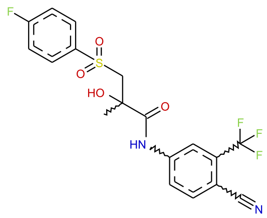	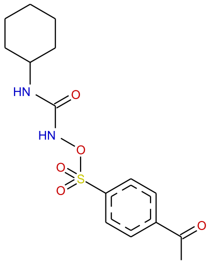	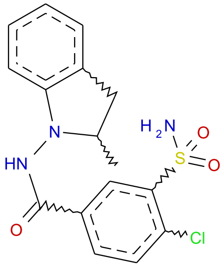
Actual Endpoint	Carcinogen	Non-Carcinogen	Non-Carcinogen
Predicted Endpoint	Carcinogen	Non-Carcinogen	Non-Carcinogen
Distance	0.567	0.574	0.615
Reference	US FDA (Centre for Drug Eval.& Res./Off. Testing & Res.) Sept. 1997	US FDA (Centre for Drug Eval.& Res./Off. Testing & Res.) Sept. 1997	US FDA (Centre for Drug Eval.& Res./Off. Testing & Res.) Sept. 1997

Model Applicability

Unknown features are fingerprint features in the query molecule, but not found or appearing too infrequently in the training set.

1. OPS PC29 out of range. Value: -3.5892. Training min, max, SD, explained variance: -3.1746, 3.7825, 1.007, 0.0095.

Feature Contribution

Top features for positive contribution				
**Fingerprint**	**Bit/Smiles**	**Feature Structure**	**Score**	**Carcinogen in training set**
SCFP_6	1655488245	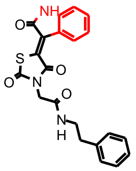 [*]1[*][c]2:[cH]:[cH]:[cH]:[cH]:[c]:2N1	0.252	4 out of 9

SCFP_6	-1375926917	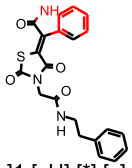 [*]N[c]1:[cH]:[*]:[c]([*]):[cH]:[cH]:1	0.251	11 out of 26
SCFP_6	1631845520	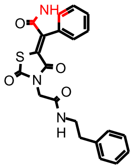 [*]C(=[*])N[c](:[*]):[*]	0.216	9 out of 22
**Top Features for negative contribution**				
**Fingerprint**	**Bit/Smiles**	**Feature Structure**	**Score**	**Carcinogen in training set**
SCFP_6	-1211866396	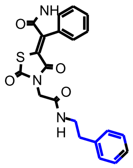 [*]CC[c]1:[cH]:[cH]:[*]:[cH]:[cH]:1	-1.1	2 out of 25
SCFP_6	-1849236245	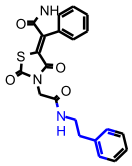 [*]NCC[c](:[cH]:[*]):[cH]:[*]	-0.825	0 out of 4
SCFP_6	-1640858361	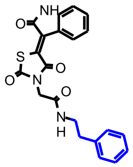 [*]CC[c]1:[cH]:[cH]:[cH]:[cH]:[cH]:1	-0.817	1 out of 11

Sorafenib

TOPKAT_Rat_Male_FDA_None_vs_Carcinogen

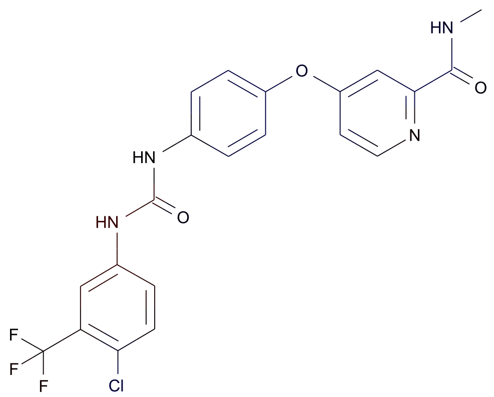

$C_{21}H_{16}ClF_3N_4O_3$
Molecular Weight: 464.82494
ALogP: 4.175
Rotatable Bonds: 6
Acceptors: 4
Donors: 3

Structural Similar Compounds

Name	Glyburide	Glimepride	Fluvastatin
Structure	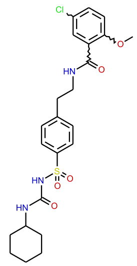	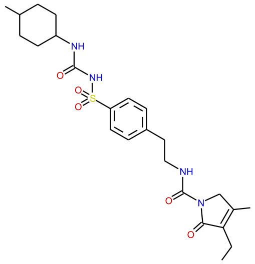	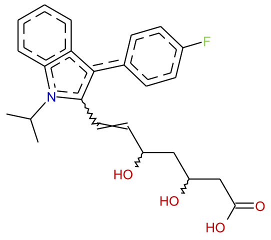
Actual Endpoint	Non-Carcinogen	Non-Carcinogen	Carcinogen
Predicted Endpoint	Non-Carcinogen	Non-Carcinogen	Carcinogen
Distance	0.593	0.600	0.615
Reference	US FDA (Centre for Drug Eval.& Res./Off. Testing & Res.) Sept. 1997	US FDA (Centre for Drug Eval.& Res./Off. Testing & Res.) Sept. 1997	US FDA (Centre for Drug Eval.& Res./Off. Testing & Res.) Sept. 1997

Model Prediction

Prediction: Non-Carcinogen
Probability: 0.293
Enrichment: 0.878
Bayesian Score: -2.4
Mahalanobis Distance: 17.6
Mahalanobis Distance p-value: 1.1e-012

Prediction: Positive if the Bayesian score is above the estimated best cutoff value from minimizing the false positive and false negative rate.
Probability: The esimated probability that the sample is in the positive category. This assumes that the Bayesian score follows a normal distribution and is different from the prediction using a cutoff.
Enrichment: An estimate of enrichment, that is, the increased likelihood (versus random) of this sample being in the category.
Bayesian Score: The standard Laplacian-modified Bayesian score.
Mahalanobis Distance: The Mahalanobis distance (MD) is the distance to the center of the training data. The larger the MD, the less trustworthy the prediction.
Mahalanobis Distance p-value: The p-value gives the fraction of training data with an MD greater than or equal to the one for the given sample, assuming normally distributed data. The smaller the p-value, the less trustworthy the prediciton. For highly non-normal X properties (e.g., fingerprints), the MD p-value is wildly inaccurate.

Model Applicability

Unknown features are fingerprint features in the query molecule, but not found or appearing too infrequently in the training set.

1. All properties and OPS components are within expected ranges.

Feature Contribution

Top features for positive contribution

Fingerprint	Bit/Smiles	Feature Structure	Score	Carcinogen in training set
SCFP_6	-347048986	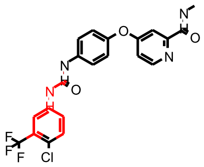 [*]C(=[*])N[c]1:[cH]:[cH]:[*]:[cH]:[cH]:1	0.615	5 out of 7

SCFP_6	-754059116	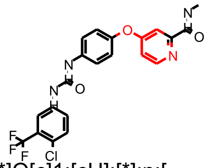 [*]O[c]1:[cH]:[*]:n:[cH]:[cH]:1	0.415	1 out of 1
SCFP_6	-531283893	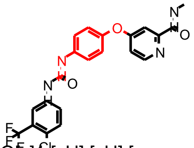 [*]O[c]1:[cH]:[cH]:[c](NC(=[*])[*]):[cH]:[cH]:1	0.273	2 out of 4
**Top Features for negative contribution**				
**Fingerprint**	**Bit/Smiles**	**Feature Structure**	**Score**	**Carcinogen in training set**
SCFP_6	-827073191	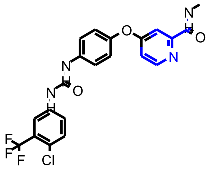 [*]C(=[*])[c]1:[cH]:[*]:[cH]:[cH]:n:1	-0.674	0 out of 3
SCFP_6	-975241316	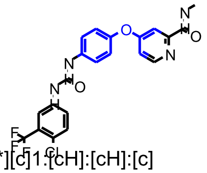 [*][c]1:[cH]:[cH]:[c](O[c](:[cH]:[*]):[cH]:[*]):[cH]:[cH]:1	-0.496	0 out of 2
SCFP_6	-488587948	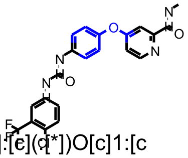 [*]:[c](:[*])O[c]1:[cH]:[cH]:[*]:[cH]:[cH]:1	-0.496	0 out of 2

Sunitinib

TOPKAT_Rat_Male_FDA_None_vs_Carcinogen

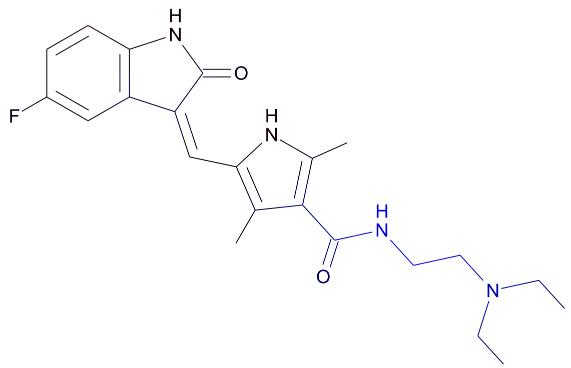

$C_{22}H_{27}FN_4O_2$
Molecular Weight: 398.47378
ALogP: 2.997
Rotatable Bonds: 7
Acceptors: 3
Donors: 3

Structural Similar Compounds

Name	Fluvastatin	Metoclopramide	Flecainide
Structure	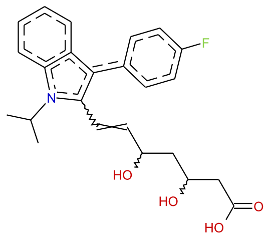	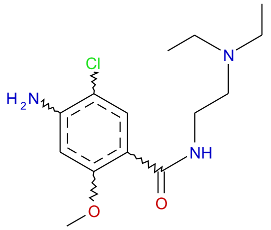	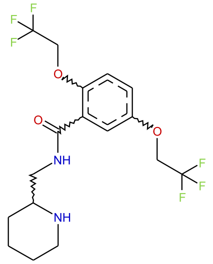
Actual Endpoint	Carcinogen	Non-Carcinogen	Non-Carcinogen
Predicted Endpoint	Carcinogen	Non-Carcinogen	Non-Carcinogen
Distance	0.599	0.634	0.643
Reference	US FDA (Centre for Drug Eval.& Res./Off. Testing & Res.) Sept. 1997	US FDA (Centre for Drug Eval.& Res./Off. Testing & Res.) Sept. 1997	US FDA (Centre for Drug Eval.& Res./Off. Testing & Res.) Sept. 1997

Model Prediction

Prediction: Non-Carcinogen
Probability: 0.217
Enrichment: 0.648
Bayesian Score: -6.26
Mahalanobis Distance: 15.7
Mahalanobis Distance p-value: 2.18e-008

Prediction: Positive if the Bayesian score is above the estimated best cutoff value from minimizing the false positive and false negative rate.
Probability: The esimated probability that the sample is in the positive category. This assumes that the Bayesian score follows a normal distribution and is different from the prediction using a cutoff.
Enrichment: An estimate of enrichment, that is, the increased likelihood (versus random) of this sample being in the category.
Bayesian Score: The standard Laplacian-modified Bayesian score.
Mahalanobis Distance: The Mahalanobis distance (MD) is the distance to the center of the training data. The larger the MD, the less trustworthy the prediction.
Mahalanobis Distance p-value: The p-value gives the fraction of training data with an MD greater than or equal to the one for the given sample, assuming normally distributed data. The smaller the p-value, the less trustworthy the prediciton. For highly non-normal X properties (e.g., fingerprints), the MD p-value is wildly inaccurate.

Model Applicability

Unknown features are fingerprint features in the query molecule, but not found or appearing too infrequently in the training set.

1. OPS PC28 out of range. Value: 3.8517. Training min, max, SD, explained variance: -3.142, 3.4791, 1.048, 0.0102.

Feature Contribution

Top features for positive contribution				
**Fingerprint**	**Bit/Smiles**	**Feature Structure**	**Score**	**Carcinogen in training set**
SCFP_6	-1971137145	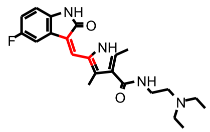 [*]C(=C[c](:[*]):[*])[*]	0.434	5 out of 9

SCFP_6	136686699	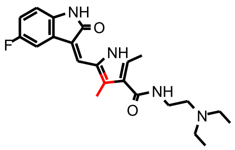 [*]:[c](:[*])C	0.287	17 out of 39
SCFP_6	-1375926917	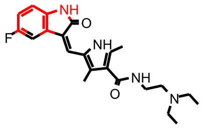 [*]N[c]1:[cH]:[*]:[c]([*]):[cH]:[cH]:1	0.251	11 out of 26

Top Features for negative contribution

Fingerprint	Bit/Smiles	Feature Structure	Score	Carcinogen in training set
SCFP_6	1323614814	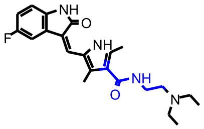 [*]CCNC(=O)[c](:[*]):[*]	-0.825	0 out of 4
SCFP_6	1798334293	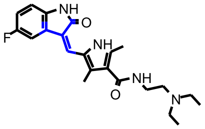 [*]C(=C1C(=[*])[*][*]:[c]1:[*])[*]	-0.674	0 out of 3
SCFP_6	-182283812	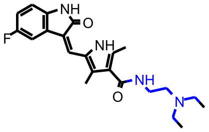 [*]CN(C[*])CCN[*]	-0.674	0 out of 3

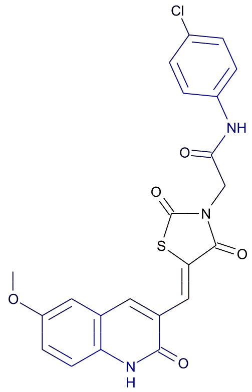

$C_{22}H_{16}ClN_3O_5S$
Molecular Weight: 469.89753
ALogP: 2.815
Rotatable Bonds: 5
Acceptors: 6
Donors: 2

Model Prediction

Prediction: Non-Irritant
Probability: 0.0465
Enrichment: 0.0505
Bayesian Score: -6.3
Mahalanobis Distance: 13.4
Mahalanobis Distance p-value: 5.13e-009

Prediction: Positive if the Bayesian score is above the estimated best cutoff value from minimizing the false positive and false negative rate.
Probability: The esimated probability that the sample is in the positive category. This assumes that the Bayesian score follows a normal distribution and is different from the prediction using a cutoff.
Enrichment: An estimate of enrichment, that is, the increased likelihood (versus random) of this sample being in the category.
Bayesian Score: The standard Laplacian-modified Bayesian score.
Mahalanobis Distance: The Mahalanobis distance (MD) is the distance to the center of the training data. The larger the MD, the less trustworthy the prediction.
Mahalanobis Distance p-value: The p-value gives the fraction of training data with an MD greater than or equal to the one for the given sample, assuming normally distributed data. The smaller the p-value, the less trustworthy the prediciton. For highly non-normal X properties (e.g., fingerprints), the MD p-value is wildly inaccurate.

Structural Similar Compounds

Name	2-Anthracenesulfonic acid, 1-amino-9,10-dihydro-9,10-dioxo-4-(2,4,6 -trimethylanilino)-, monosodium salt	Pregna-1,4-diene-3,20-dione, 21-(acetyloxy)-11-hydroxy-6-methyl-17- (1-oxopropoxy)-, (6-alpha,11-beta)-	Benzenesulfonic acid, 2,2'-(4,4'-biphenylylenedivinylene)di-, disod ium salt
Structure	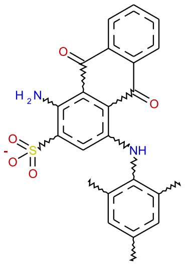	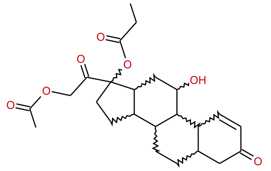	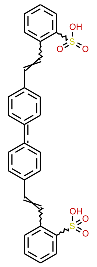
Actual Endpoint	Irritant	Irritant	Irritant
Predicted Endpoint	Non-Irritant	Irritant	Non-Irritant
Distance	0.649	0.777	0.802
Reference	85JCAE "Prehled Prumyslove Toxikologie; Organicke Latky," Marhold, J., Prague , Czechoslovakia, Avicenum, 1986 Volume(issue)/page/year: -,1327,1986	YACHDS Yakuri to Chiryo. Pharmacology and Therapeutics. (Raifu Saiensu Shup pan K.K., 2-5-13, Yaesu, Chuo-ku, Tokyo 104, Japan) V.1-1972- Volume(issue) /page/year: 19,3103,1991	MVCRB3 MVC-Report. (Stockholm, Sweden) No.1-2, 1972-73. Discontinued. Volu me(issue)/page/year: 2,193,1973

Model Applicability

Unknown features are fingerprint features in the query molecule, but not found or appearing too infrequently in the training set.

1. All properties and OPS components are within expected ranges.

Feature Contribution

Top features for positive contribution				
**Fingerprint**	**Bit/Smiles**	**Feature Structure**	**Score**	**Irritant in training set**

FCFP_12	-1986158408	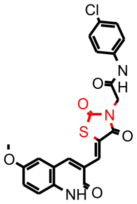 [*]N1[*][*]SC1=O	0.0821	13 out of 13
FCFP_12	436886043	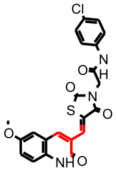 [*]\C=C(\C=[*])/C(=[*])[*]	0.0804	129 out of 130
FCFP_12	1383817444	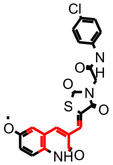 [*]=CC1=C[c](:[cH]:[*]):[c](:[*])[*]C1=[*]	0.0772	7 out of 7

Top Features for negative contribution

Fingerprint	Bit/Smiles	Feature Structure	Score	Irritant in training set
FCFP_12	1175665944	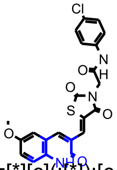 [*]C1=[*][c](:[*]):[c](NC1=O):[cH]:[*]	-1.02	2 out of 8
FCFP_12	-1838187238	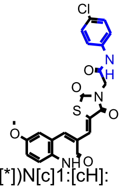 [*]C(=[*])N[c]1:[cH]:[cH]:[*]:[cH]:[cH]:1	-0.692	5 out of 12

FCFP_12	-451043714	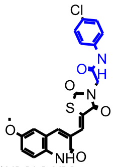 [*]CC(=O)N[c]1:[cH]:[cH]:[c]([*]):[cH]:[cH]:1	-0.65	0 out of 1

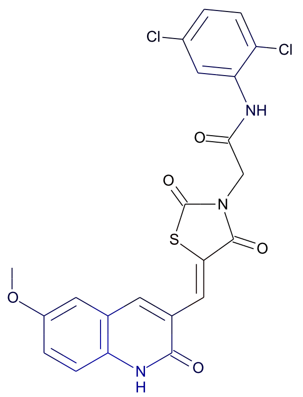

$C_{22}H_{15}Cl_2N_3O_5S$
Molecular Weight: 504.3426
ALogP: 3.479
Rotatable Bonds: 5
Acceptors: 6
Donors: 2

Model Prediction

Prediction: Non-Irritant
Probability: 0.148
Enrichment: 0.161
Bayesian Score: -5.62
Mahalanobis Distance: 13.5
Mahalanobis Distance p-value: 4.1e-009

Prediction: Positive if the Bayesian score is above the estimated best cutoff value from minimizing the false positive and false negative rate.
Probability: The esimated probability that the sample is in the positive category. This assumes that the Bayesian score follows a normal distribution and is different from the prediction using a cutoff.
Enrichment: An estimate of enrichment, that is, the increased likelihood (versus random) of this sample being in the category.
Bayesian Score: The standard Laplacian-modified Bayesian score.
Mahalanobis Distance: The Mahalanobis distance (MD) is the distance to the center of the training data. The larger the MD, the less trustworthy the prediction.
Mahalanobis Distance p-value: The p-value gives the fraction of training data with an MD greater than or equal to the one for the given sample, assuming normally distributed data. The smaller the p-value, the less trustworthy the prediciton. For highly non-normal X properties (e.g., fingerprints), the MD p-value is wildly inaccurate.

Structural Similar Compounds

Name	2-Anthracenesulfonic acid, 1-amino-9,10-dihydro-9,10-dioxo-4-(2,4,6 -trimethylanilino)-, monosodium salt	Benzenesulfonic acid, 2,2'-(4,4'-biphenylylenedivinylene)di-, disod ium salt	Pregna-1,4-diene-3,20-dione, 21-(acetyloxy)-11-hydroxy-6-methyl-17- (1-oxopropoxy)-, (6-alpha,11-beta)-
Structure	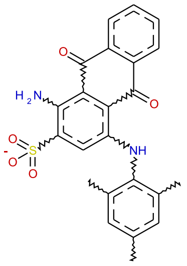	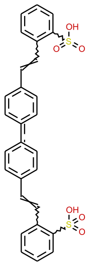	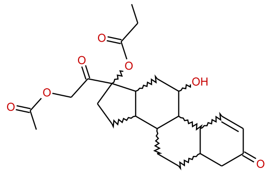
Actual Endpoint	Irritant	Irritant	Irritant
Predicted Endpoint	Non-Irritant	Non-Irritant	Irritant
Distance	0.718	0.728	0.820
Reference	85JCAE "Prehled Prumyslove Toxikologie; Organicke Latky," Marhold, J., Prague , Czechoslovakia, Avicenum, 1986 Volume(issue)/page/year: -,1327,1986	MVCRB3 MVC-Report. (Stockholm, Sweden) No.1-2, 1972-73. Discontinued. Volu me(issue)/page/year: 2,193,1973	YACHDS Yakuri to Chiryo. Pharmacology and Therapeutics. (Raifu Saiensu Shup pan K.K., 2-5-13, Yaesu, Chuo-ku, Tokyo 104, Japan) V.1-1972- Volume(issue) /page/year: 19,3103,1991

Model Applicability

Unknown features are fingerprint features in the query molecule, but not found or appearing too infrequently in the training set.

1. All properties and OPS components are within expected ranges.

Feature Contribution

Top features for positive contribution				
**Fingerprint**	**Bit/Smiles**	**Feature Structure**	**Score**	**Irritant in training set**

FCFP_12	-1986158408	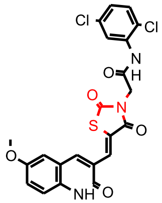 [*]N1[*][*]SC1=O	0.0821	13 out of 13
FCFP_12	436886043	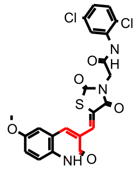 [*]\C=C(\C=[*])/C(=[*])[*]	0.0804	129 out of 130
FCFP_12	1383817444	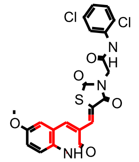 [*]=CC1=C[c](:[cH]:[*]):[c](:[*])[*]C1=[*]	0.0772	7 out of 7
**Top Features for negative contribution**				
**Fingerprint**	**Bit/Smiles**	**Feature Structure**	**Score**	**Irritant in training set**
FCFP_12	1175665944	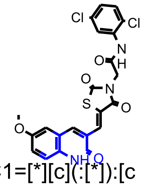 [*]C1=[*][c](:[*]):[c](NC1=O):[cH]:[*]	-1.02	2 out of 8
FCFP_12	-1757681964	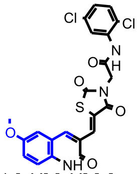 [*][c]1:[cH]:[cH]:[c](OC):[cH]:[c]:1C=[*]	-0.627	1 out of 3

FCFP_12	1783756416	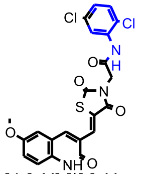<br>[*]N[c]1:[cH]:[*]:[cH<br>]:[cH]:[c]:1Cl	-0.509	4 out of 8

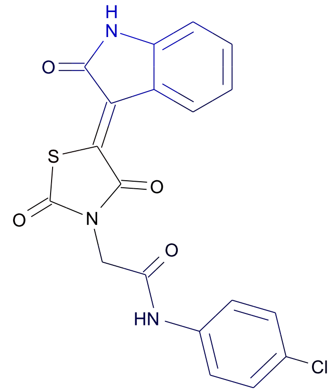

$C_{19}H_{12}ClN_3O_4S$
Molecular Weight: 413.83428
ALogP: 2.363
Rotatable Bonds: 3
Acceptors: 5
Donors: 2

Model Prediction

Prediction: Non-Irritant
Probability: 0.0408
Enrichment: 0.0443
Bayesian Score: -6.37
Mahalanobis Distance: 11.4
Mahalanobis Distance p-value: 0.000388

Prediction: Positive if the Bayesian score is above the estimated best cutoff value from minimizing the false positive and false negative rate.
Probability: The esimated probability that the sample is in the positive category. This assumes that the Bayesian score follows a normal distribution and is different from the prediction using a cutoff.
Enrichment: An estimate of enrichment, that is, the increased likelihood (versus random) of this sample being in the category.
Bayesian Score: The standard Laplacian-modified Bayesian score.
Mahalanobis Distance: The Mahalanobis distance (MD) is the distance to the center of the training data. The larger the MD, the less trustworthy the prediction.
Mahalanobis Distance p-value: The p-value gives the fraction of training data with an MD greater than or equal to the one for the given sample, assuming normally distributed data. The smaller the p-value, the less trustworthy the prediciton. For highly non-normal X properties (e.g., fingerprints), the MD p-value is wildly inaccurate.

Structural Similar Compounds

Name	2-Anthracenesulfonic acid, 1-amino-9,10-dihydro-9,10-dioxo-4-(2,4,6 -trimethylanilino)-, monosodium salt	Benzenesulfonic acid, 2-anilino-5-nitro-	5-Norbornene-2,3-dicarboxylic acid, 1,4,5,6,7,7-hexachloro-
Structure	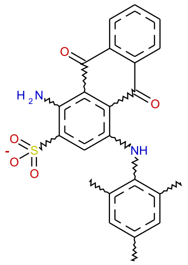	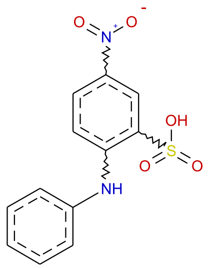	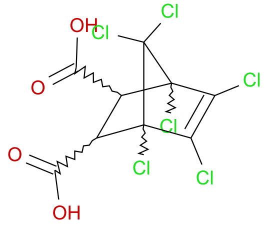
Actual Endpoint	Irritant	Irritant	Irritant
Predicted Endpoint	Non-Irritant	Non-Irritant	Irritant
Distance	0.683	0.763	0.773
Reference	85JCAE "Prehled Prumyslove Toxikologie; Organicke Latky," Marhold, J., Prague , Czechoslovakia, Avicenum, 1986 Volume(issue)/page/year: -,1327,1986	85JCAE "Prehled Prumyslove Toxikologie; Organicke Latky," Marhold, J., Prague , Czechoslovakia, Avicenum, 1986 Volume(issue)/page/year: -,1061,1986	85JCAE "Prehled Prumyslove Toxikologie; Organicke Latky," Marhold, J., Prague , Czechoslovakia, Avicenum, 1986 Volume(issue)/page/year: -,581,1986

Model Applicability

Unknown features are fingerprint features in the query molecule, but not found or appearing too infrequently in the training set.

1. All properties and OPS components are within expected ranges.

Feature Contribution

Top features for positive contribution				
**Fingerprint**	**Bit/Smiles**	**Feature Structure**	**Score**	**Irritant in training set**

FCFP_12	-1986158408	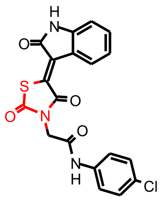 [*]N1[*][*]SC1=O	0.0821	13 out of 13
FCFP_12	436915834	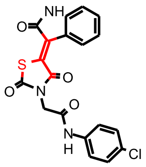 [*]\C=C\1/S[*][*]C1=[*]	0.0756	6 out of 6
FCFP_12	-1143715940	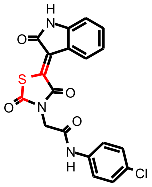 [*]=C1[*][*]C(=[*])S1	0.0575	475 out of 490

Top Features for negative contribution

Fingerprint	Bit/Smiles	Feature Structure	Score	Irritant in training set
FCFP_12	1175665944	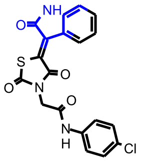 [*]C1=[*][c](:[*]):[c](NC1=O):[cH]:[*]	-1.02	2 out of 8
FCFP_12	-1838187238	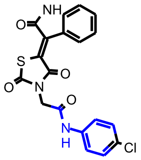 [*]C(=[*])N[c]1:[cH]:[cH]:[*]:[cH]:[cH]:1	-0.692	5 out of 12

FCFP_12	-451043714	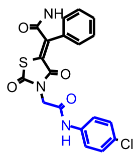 [*]CC(=O)N[c]1:[cH]:[cH]:[c]([*]):[cH]:[cH]:1	-0.65	0 out of 1

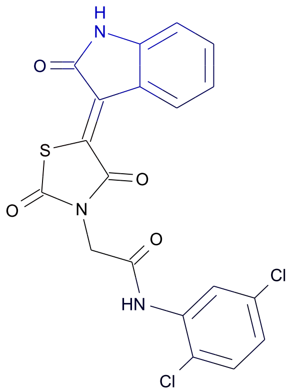

$C_{19}H_{11}Cl_2N_3O_4S$
Molecular Weight: 448.27933
ALogP: 3.028
Rotatable Bonds: 3
Acceptors: 5
Donors: 2

Model Prediction

Prediction: Non-Irritant
Probability: 0.133
Enrichment: 0.145
Bayesian Score: -5.69
Mahalanobis Distance: 11.5
Mahalanobis Distance p-value: 0.000313

Prediction: Positive if the Bayesian score is above the estimated best cutoff value from minimizing the false positive and false negative rate.
Probability: The esimated probability that the sample is in the positive category. This assumes that the Bayesian score follows a normal distribution and is different from the prediction using a cutoff.
Enrichment: An estimate of enrichment, that is, the increased likelihood (versus random) of this sample being in the category.
Bayesian Score: The standard Laplacian-modified Bayesian score.
Mahalanobis Distance: The Mahalanobis distance (MD) is the distance to the center of the training data. The larger the MD, the less trustworthy the prediction.
Mahalanobis Distance p-value: The p-value gives the fraction of training data with an MD greater than or equal to the one for the given sample, assuming normally distributed data. The smaller the p-value, the less trustworthy the prediciton. For highly non-normal X properties (e.g., fingerprints), the MD p-value is wildly inaccurate.

Structural Similar Compounds

Name	2-Anthracenesulfonic acid, 1-amino-9,10-dihydro-9,10-dioxo-4-(2,4,6 -trimethylanilino)-, monosodium salt	5-Norbornene-2,3-dicarboxylic acid, 1,4,5,6,7,7-hexachloro-	1-Amino-2-bromo-4-hydroxyanthraquinone
Structure	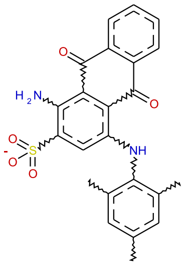	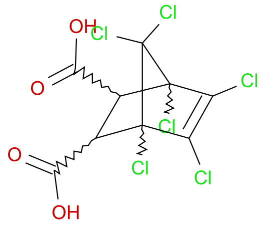	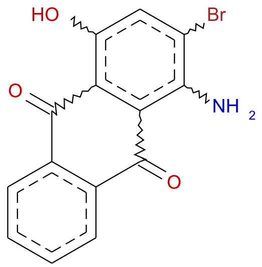
Actual Endpoint	Irritant	Irritant	Non-Irritant
Predicted Endpoint	Non-Irritant	Irritant	Non-Irritant
Distance	0.695	0.791	0.824
Reference	85JCAE "Prehled Prumyslove Toxikologie; Organicke Latky," Marhold, J., Prague , Czechoslovakia, Avicenum, 1986 Volume(issue)/page/year: -,1327,1986	85JCAE "Prehled Prumyslove Toxikologie; Organicke Latky," Marhold, J., Prague , Czechoslovakia, Avicenum, 1986 Volume(issue)/page/year: -,581,1986	28ZPAK -,83,72

Model Applicability

Unknown features are fingerprint features in the query molecule, but not found or appearing too infrequently in the training set.

1. All properties and OPS components are within expected ranges.

Feature Contribution

Top features for positive contribution				
Fingerprint	Bit/Smiles	Feature Structure	Score	Irritant in training set

FCFP_12	-1986158408	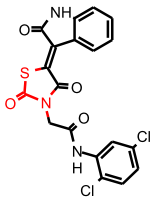 [*]N1[*][*]SC1=O	0.0821	13 out of 13
FCFP_12	436915834	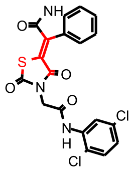 [*]\C=C\1/S[*][*]C1=[*]	0.0756	6 out of 6
FCFP_12	562194858	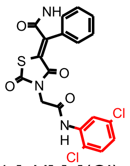 [*][c]1:[cH]:[c](Cl):[cH]:[cH]:[c]:1Cl	0.0583	2 out of 2
**Top Features for negative contribution**				
**Fingerprint**	**Bit/Smiles**	**Feature Structure**	**Score**	**Irritant in training set**
FCFP_12	1175665944	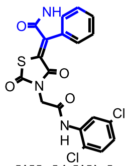 [*]C1=[*][c](:[*]):[c](NC1=O):[cH]:[*]	-1.02	2 out of 8
FCFP_12	1783756416	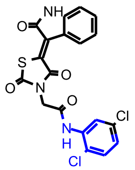 [*]N[c]1:[cH]:[*]:[cH]:[cH]:[c]:1Cl	-0.509	4 out of 8

FCFP_12	1294255210	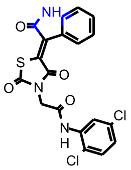 [*]C(=[*])N[c](:[*]):[*]	-0.486	12 out of 22

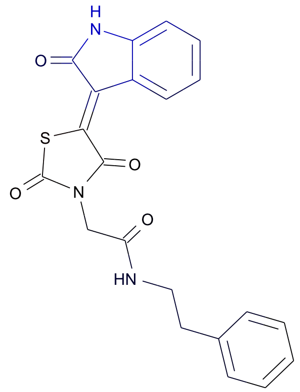

$C_{21}H_{17}N_3O_4S$
Molecular Weight: 407.44237
ALogP: 2.027
Rotatable Bonds: 5
Acceptors: 5
Donors: 2

Model Prediction

Prediction: Non-Irritant
Probability: 0.189
Enrichment: 0.205
Bayesian Score: -5.45
Mahalanobis Distance: 12
Mahalanobis Distance p-value: 2.07e-005

Prediction: Positive if the Bayesian score is above the estimated best cutoff value from minimizing the false positive and false negative rate.
Probability: The esimated probability that the sample is in the positive category. This assumes that the Bayesian score follows a normal distribution and is different from the prediction using a cutoff.
Enrichment: An estimate of enrichment, that is, the increased likelihood (versus random) of this sample being in the category.
Bayesian Score: The standard Laplacian-modified Bayesian score.
Mahalanobis Distance: The Mahalanobis distance (MD) is the distance to the center of the training data. The larger the MD, the less trustworthy the prediction.
Mahalanobis Distance p-value: The p-value gives the fraction of training data with an MD greater than or equal to the one for the given sample, assuming normally distributed data. The smaller the p-value, the less trustworthy the prediciton. For highly non-normal X properties (e.g., fingerprints), the MD p-value is wildly inaccurate.

Structural Similar Compounds

Name	2-Anthracenesulfonic acid, 1-amino-9,10-dihydro-9,10-dioxo-4-(2,4,6 -trimethylanilino)-, monosodium salt	Benzenesulfonic acid, 2-anilino-5-nitro-	Benzenesulfonamide, 4-amino-N-(5,6-dimethoxy-4-pyrimidinyl)-
Structure	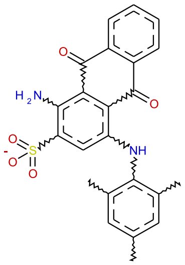	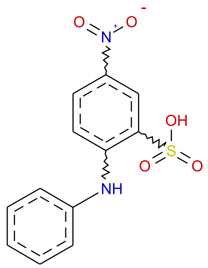	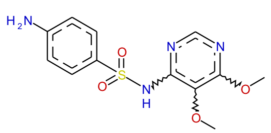
Actual Endpoint	Irritant	Irritant	Irritant
Predicted Endpoint	Non-Irritant	Non-Irritant	Non-Irritant
Distance	0.705	0.765	0.784
Reference	85JCAE "Prehled Prumyslove Toxikologie; Organicke Latky," Marhold, J., Prague , Czechoslovakia, Avicenum, 1986 Volume(issue)/page/year: -,1327,1986	85JCAE "Prehled Prumyslove Toxikologie; Organicke Latky," Marhold, J., Prague , Czechoslovakia, Avicenum, 1986 Volume(issue)/page/year: -,1061,1986	FCTXAV 14,307,76

Model Applicability

Unknown features are fingerprint features in the query molecule, but not found or appearing too infrequently in the training set.

1. All properties and OPS components are within expected ranges.

Feature Contribution

Top features for positive contribution				
**Fingerprint**	**Bit/Smiles**	**Feature Structure**	**Score**	**Irritant in training set**

FCFP_12	-1986158408	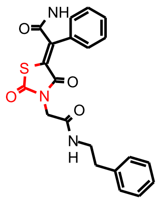 [*]N1[*][*]SC1=O	0.0821	13 out of 13
FCFP_12	436915834	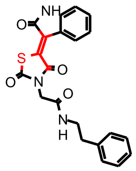 [*]\C=C\1/S[*][*]C1=[*]	0.0756	6 out of 6
FCFP_12	-1143715940	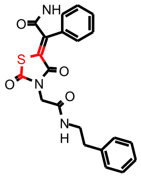 [*]=C1[*][*]C(=[*])S1	0.0575	475 out of 490

Top Features for negative contribution

**Fingerprint**	**Bit/Smiles**	**Feature Structure**	**Score**	**Irritant in training set**
FCFP_12	1175665944	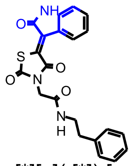 [*]C1=[*][c](:[*]):[c](NC1=O):[cH]:[*]	-1.02	2 out of 8
FCFP_12	-2002900105	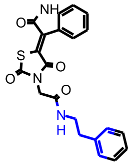 [*]NCC[c](:[cH]:[*]):[cH]:[*]	-0.65	0 out of 1

FCFP_12	1294255210	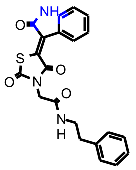 [*]C(=[*])N[c](:[*]):[*]	-0.486	12 out of 22

Sorafenib

TOPKAT_Skin_Irritancy_None_vs_Irritant

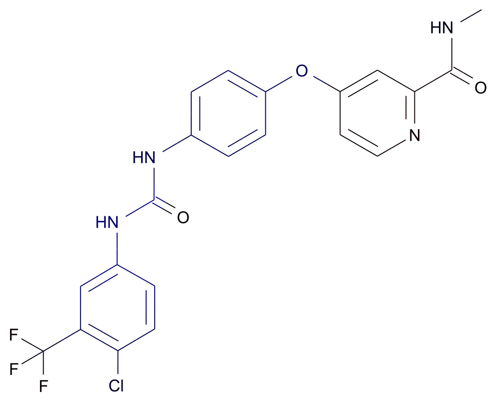

$C_{21}H_{16}ClF_3N_4O_3$

Molecular Weight: 464.82494

ALogP: 4.175

Rotatable Bonds: 6

Acceptors: 4

Donors: 3

Model Prediction

Prediction: Non-Irritant

Probability: 0.264

Enrichment: 0.287

Bayesian Score: -5.23

Mahalanobis Distance: 8.27

Mahalanobis Distance p-value: 0.791

Prediction: Positive if the Bayesian score is above the estimated best cutoff value from minimizing the false positive and false negative rate.
Probability: The esimated probability that the sample is in the positive category. This assumes that the Bayesian score follows a normal distribution and is different from the prediction using a cutoff.
Enrichment: An estimate of enrichment, that is, the increased likelihood (versus random) of this sample being in the category.
Bayesian Score: The standard Laplacian-modified Bayesian score.
Mahalanobis Distance: The Mahalanobis distance (MD) is the distance to the center of the training data. The larger the MD, the less trustworthy the prediction.
Mahalanobis Distance p-value: The p-value gives the fraction of training data with an MD greater than or equal to the one for the given sample, assuming normally distributed data. The smaller the p-value, the less trustworthy the prediciton. For highly non-normal X properties (e.g., fingerprints), the MD p-value is wildly inaccurate.

Structural Similar Compounds

Name	5-Norbornene-2,3-dicarboxylic acid, 1,4,5,6,7,7-hexachloro-	Benzenesulfonic acid, 2,2'-(4,4'-biphenylylenedivinylene)di-, disod ium salt	Sulfide, bis(4-t-butyl-m-cresyl)-
Structure	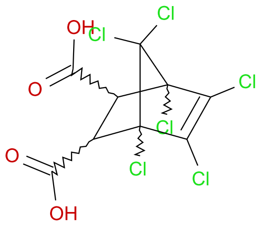	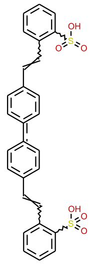	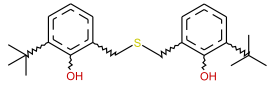
Actual Endpoint	Irritant	Irritant	Irritant
Predicted Endpoint	Irritant	Non-Irritant	Irritant
Distance	0.844	0.871	0.884
Reference	85JCAE "Prehled Prumyslove Toxikologie; Organicke Latky," Marhold, J., Prague , Czechoslovakia, Avicenum, 1986 Volume(issue)/page/year: -,581,1986	MVCRB3 MVC-Report. (Stockholm, Sweden) No.1-2, 1972-73. Discontinued. Volu me(issue)/page/year: 2,193,1973	AMIHBC AMA Archives of Industrial Hygiene and Occupational Medicine. (Chicago , IL) V.2-10, 1950-54. For publisher information, see AEHLAU. Volume(issue)/pag e/year: 5,311,1952

Model Applicability

Unknown features are fingerprint features in the query molecule, but not found or appearing too infrequently in the training set.

1. All properties and OPS components are within expected ranges.

Feature Contribution

Top features for positive contribution				
Fingerprint	Bit/Smiles	Feature Structure	Score	Irritant in training set

FCFP_12	-124655670	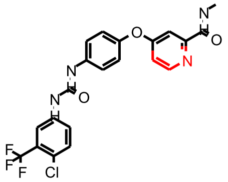 [*]:[cH]:[cH]:n:[*]	0.0821	13 out of 13
FCFP_12	-1539132615	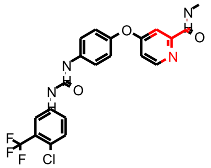 [*]C(=[*])[c](:[cH]:[*]):n:[*]	0.0795	9 out of 9
FCFP_12	-1695756380	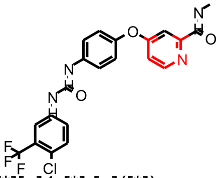 [*][c]1:[*]:[c]([*]): n:[cH]:[cH]:1	0.0772	7 out of 7
**Top Features for negative contribution**				
**Fingerprint**	**Bit/Smiles**	**Feature Structure**	**Score**	**Irritant in training set**
FCFP_12	-789307649	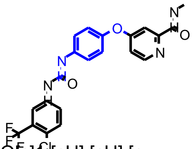 [*]O[c]1:[cH]:[cH]:[c](NC(=[*])[*]):[cH]: [cH]:1	-1.54	0 out of 4
FCFP_12	-1838187238	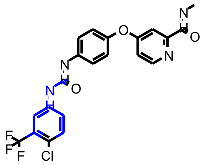 [*]C(=[*])N[c]1:[cH]: [cH]:[*]:[cH]:[cH]:1	-0.692	5 out of 12

FCFP_12	1294255210	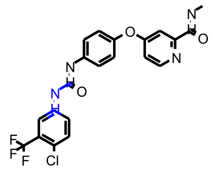 [*]C(=[*])N[c](:[*]):[*]	-0.486	12 out of 22

Sunitinib

TOPKAT_Skin_Irritancy_None_vs_Irritant

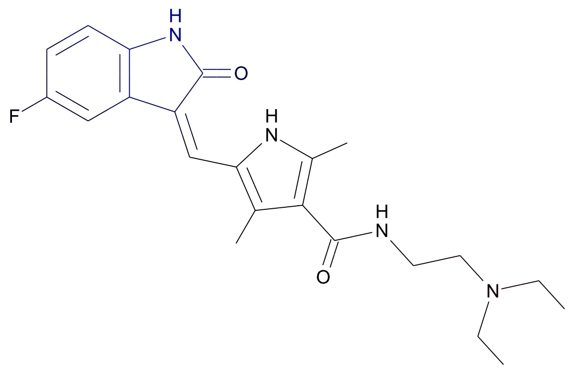

$C_{22}H_{27}FN_4O_2$
Molecular Weight: 398.47378
ALogP: 2.997
Rotatable Bonds: 7
Acceptors: 3
Donors: 3

Model Prediction

Prediction: Non-Irritant
Probability: 0.856
Enrichment: 0.93
Bayesian Score: -3.16
Mahalanobis Distance: 10.1
Mahalanobis Distance p-value: 0.0541

Prediction: Positive if the Bayesian score is above the estimated best cutoff value from minimizing the false positive and false negative rate.
Probability: The esimated probability that the sample is in the positive category. This assumes that the Bayesian score follows a normal distribution and is different from the prediction using a cutoff.
Enrichment: An estimate of enrichment, that is, the increased likelihood (versus random) of this sample being in the category.
Bayesian Score: The standard Laplacian-modified Bayesian score.
Mahalanobis Distance: The Mahalanobis distance (MD) is the distance to the center of the training data. The larger the MD, the less trustworthy the prediction.
Mahalanobis Distance p-value: The p-value gives the fraction of training data with an MD greater than or equal to the one for the given sample, assuming normally distributed data. The smaller the p-value, the less trustworthy the prediciton. For highly non-normal X properties (e.g., fingerprints), the MD p-value is wildly inaccurate.

Structural Similar Compounds

Name	5-Norbornene-2,3-dicarboxylic acid, 1,4,5,6,7,7-hexachloro-	Sulfide, bis(4-t-butyl-m-cresyl)-	p-Acetophenetidide, 3'-(bis(2-hydroxyethyl)amino)-
Structure	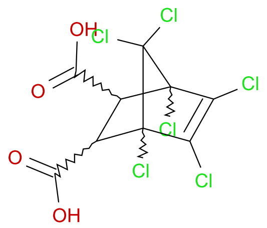	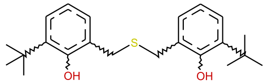	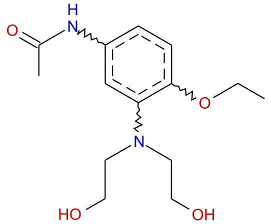
Actual Endpoint	Irritant	Irritant	Non-Irritant
Predicted Endpoint	Irritant	Irritant	Non-Irritant
Distance	0.818	0.860	0.863
Reference	85JCAE "Prehled Prumyslove Toxikologie; Organicke Latky," Marhold, J., Prague , Czechoslovakia, Avicenum, 1986 Volume(issue)/page/year: -,581,1986	AMIHBC AMA Archives of Industrial Hygiene and Occupational Medicine. (Chicago , IL) V.2-10, 1950-54. For publisher information, see AEHLAU. Volume(issue)/pag e/year: 5,311,1952	28ZPAK -,100,72

Model Applicability

Unknown features are fingerprint features in the query molecule, but not found or appearing too infrequently in the training set.

1. All properties and OPS components are within expected ranges.
2. Unknown FCFP_2 feature: 203707511: [*][c]1:[*]:[*]:[nH]:[c]:1C=[*]

Feature Contribution

Top features for positive contribution

Fingerprint	Bit/Smiles	Feature Structure	Score	Irritant in training set

FCFP_12	1852108031	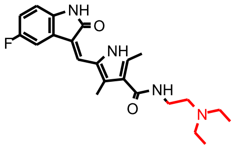<br>[*]CCN(CC)CC	0.0841	19 out of 19
FCFP_12	1851332093	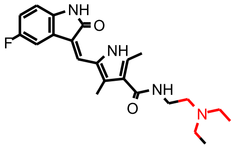<br>[*]CN(C[*])CC	0.0795	9 out of 9
FCFP_12	-371808660	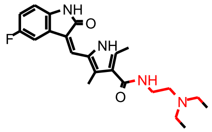<br>[*]CN(C[*])CCN[*]	0.0785	8 out of 8

Top Features for negative contribution

**Fingerprint**	**Bit/Smiles**	**Feature Structure**	**Score**	**Irritant in training set**
FCFP_12	1175665944	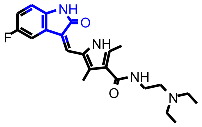<br>[*]C1=[*][c](:[*]):[c<br>](NC1=O):[cH]:[*]	-1.02	2 out of 8
FCFP_12	1294255210	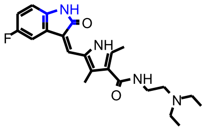<br>[*]C(=[*])N[c](:[*]):<br>[*]	-0.486	12 out of 22

FCFP_12	-773983804	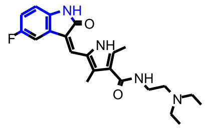<br>[*]N[c]1:[cH]:[*]:[c]([*]):[cH]:[cH]:1	-0.444	46 out of 79

TOPKAT_Carcinogenic_Potency_TD50_Mouse

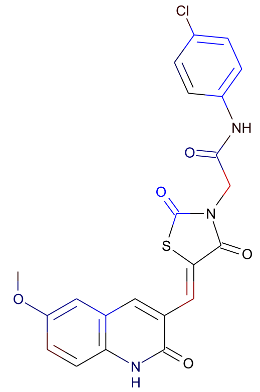

$C_{22}H_{16}ClN_3O_5S$
Molecular Weight: 469.89753
ALogP: 2.815
Rotatable Bonds: 5
Acceptors: 6
Donors: 2

Model Prediction

Prediction: 25.8
Unit: mg/kg_body_weight/day
Mahalanobis Distance: 14.4
Mahalanobis Distance p-value: 3.05e-011

Mahalanobis Distance: The Mahalanobis distance (MD) is a generalization of the Euclidean distance that accounts for correlations among the X properties. It is calculated as the distance to the center of the training data. The larger the MD, the less trustworthy the prediction.
Mahalanobis Distance p-value: The p-value gives the fraction of training data with an MD greater than or equal to the one for the given sample, assuming normally distributed data. The smaller the p-value, the less trustworthy the prediciton. For highly non-normal X properties (e.g., fingerprints), the MD p-value is wildly inaccurate.

Structural Similar Compounds

Name	Ochratoxin A	542	470
Structure	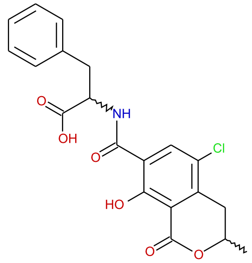	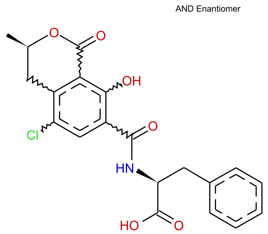	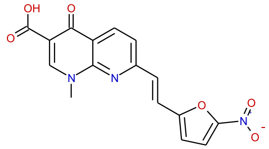
Actual Endpoint (-log C)	4.79932	4.79932	4.62839
Predicted Endpoint (-log C)	3.6353	3.6353	3.93264
Distance	0.650	0.650	0.710
Reference	CPDB	CPDB	CPDB

Model Applicability

Unknown features are fingerprint features in the query molecule, but not found or appearing too infrequently in the training set.

1. All properties and OPS components are within expected ranges.
2. Unknown ECFP_2 feature: 2131425032: [*]\C=C(\C=[*])/C(=[*])[*]
3. Unknown ECFP_2 feature: 1182722866: [*]C(=CC(=[*])[*])[*]
4. Unknown ECFP_2 feature: 1000552169: [*]\C=C\1/S[*][*]C1=[*]
5. Unknown ECFP_2 feature: -661097313: [*]CN1C(=[*])[*][*]C1=[*]
6. Unknown ECFP_2 feature: -37698365: [*]N([*])CC(=[*])[*]

Feature Contribution

Top features for positive contribution			
**Fingerprint**	**Bit/Smiles**	**Feature Structure**	**Score**
ECFP_6	1559650422	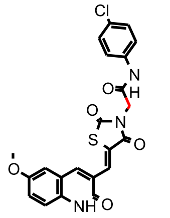 [*]C[*]	0.203

ECFP_6	-1925046727	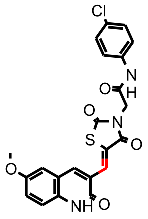 [*]C=[*]	0.145
ECFP_6	-817402818	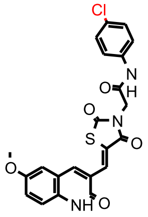 [*]Cl	0.129

Top Features for negative contribution

Fingerprint	Bit/Smiles	Feature Structure	Score
ECFP_6	2106656448	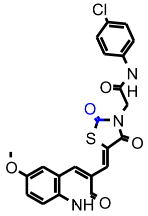 [*]C(=O)[*]	-0.275
ECFP_6	1996767644	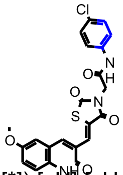 [*][c](:[*]):[cH]:[cH]:[*]	-0.251
ECFP_6	642810091	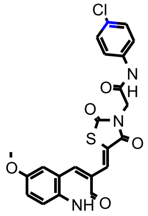 [*][c](:[*]):[*]	-0.247

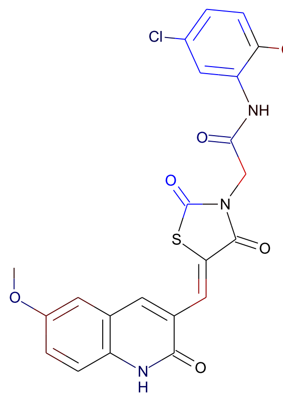

$C_{22}H_{15}Cl_2N_3O_5S$
Molecular Weight: 504.3426
ALogP: 3.479
Rotatable Bonds: 5
Acceptors: 6
Donors: 2

Model Prediction

Prediction: 20.3
Unit: mg/kg_body_weight/day
Mahalanobis Distance: 14.4
Mahalanobis Distance p-value: 2.77e-011

Mahalanobis Distance: The Mahalanobis distance (MD) is a generalization of the Euclidean distance that accounts for correlations among the X properties. It is calculated as the distance to the center of the training data. The larger the MD, the less trustworthy the prediction.
Mahalanobis Distance p-value: The p-value gives the fraction of training data with an MD greater than or equal to the one for the given sample, assuming normally distributed data. The smaller the p-value, the less trustworthy the prediciton. For highly non-normal X properties (e.g., fingerprints), the MD p-value is wildly inaccurate.

Structural Similar Compounds

Name	Ochratoxin A	542	470
Structure	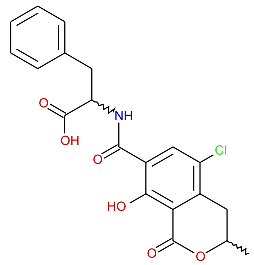	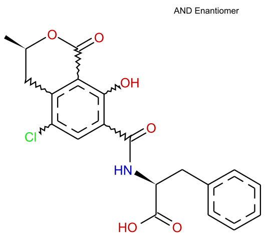	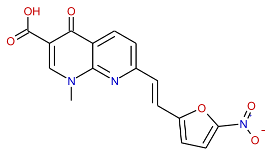
Actual Endpoint (-log C)	4.79932	4.79932	4.62839
Predicted Endpoint (-log C)	3.6353	3.6353	3.93264
Distance	0.665	0.665	0.748
Reference	CPDB	CPDB	CPDB

Model Applicability

Unknown features are fingerprint features in the query molecule, but not found or appearing too infrequently in the training set.

1. All properties and OPS components are within expected ranges.
2. Unknown ECFP_2 feature: 2131425032: [*]\C=C(\C=[*])/C(=[*])[*]
3. Unknown ECFP_2 feature: 1182722866: [*]C(=CC(=[*])[*])[*]
4. Unknown ECFP_2 feature: 1000552169: [*]\C=C\1/S[*][*]C1=[*]
5. Unknown ECFP_2 feature: -661097313: [*]CN1C(=[*])[*][*]C1=[*]
6. Unknown ECFP_2 feature: -37698365: [*]N([*])CC(=[*])[*]

Feature Contribution

Top features for positive contribution

Fingerprint	Bit/Smiles	Feature Structure	Score
ECFP_6	1559650422	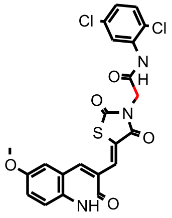 [*]C[*]	0.203

ECFP_6	-1925046727	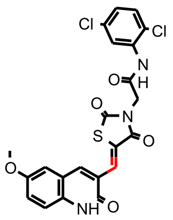<br>[*]C=[*]	0.145
ECFP_6	-817402818	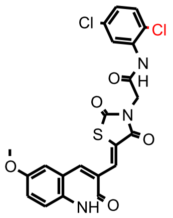<br>[*]Cl	0.129

Top Features for negative contribution

Fingerprint	Bit/Smiles	Feature Structure	Score
ECFP_6	2106656448	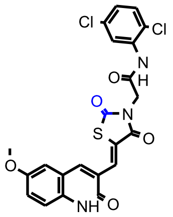<br>[*]C(=O)[*]	-0.275
ECFP_6	1996767644	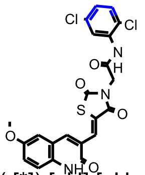<br>[*][c](:[*]):[cH]:[cH<br>]:[*]	-0.251
ECFP_6	642810091	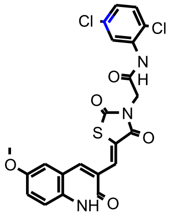<br>[*][c](:[*]):[*]	-0.247

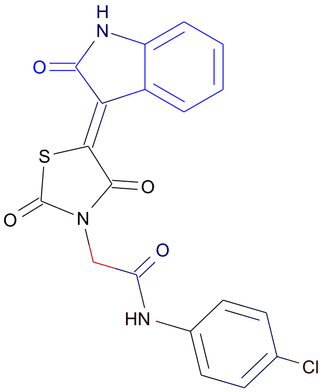

$C_{19}H_{12}ClN_3O_4S$

Molecular Weight: 413.83428

ALogP: 2.363

Rotatable Bonds: 3

Acceptors: 5

Donors: 2

Model Prediction

Prediction: 54.6

Unit: mg/kg_body_weight/day

Mahalanobis Distance: 12.8

Mahalanobis Distance p-value: 3.04e-007

Mahalanobis Distance: The Mahalanobis distance (MD) is a generalization of the Euclidean distance that accounts for correlations among the X properties. It is calculated as the distance to the center of the training data. The larger the MD, the less trustworthy the prediction.
Mahalanobis Distance p-value: The p-value gives the fraction of training data with an MD greater than or equal to the one for the given sample, assuming normally distributed data. The smaller the p-value, the less trustworthy the prediciton. For highly non-normal X properties (e.g., fingerprints), the MD p-value is wildly inaccurate.

Structural Similar Compounds

Name	Ochratoxin A	542	470
Structure	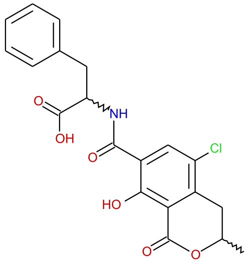	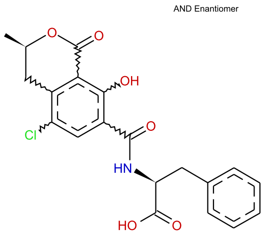	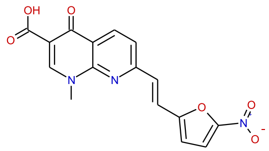
Actual Endpoint (-log C)	4.79932	4.79932	4.62839
Predicted Endpoint (-log C)	3.6353	3.6353	3.93264
Distance	0.666	0.666	0.708
Reference	CPDB	CPDB	CPDB

Model Applicability

Unknown features are fingerprint features in the query molecule, but not found or appearing too infrequently in the training set.

1. All properties and OPS components are within expected ranges.
2. Unknown ECFP_2 feature: -631778390: [*]C(=C1S[*][*]C1=[*])[*]
3. Unknown ECFP_2 feature: -661097313: [*]CN1C(=[*])[*][*]C1=[*]
4. Unknown ECFP_2 feature: -37698365: [*]N([*])CC(=[*])[*]

Feature Contribution

Top features for positive contribution			
Fingerprint	Bit/Smiles	Feature Structure	Score
ECFP_6	1559650422	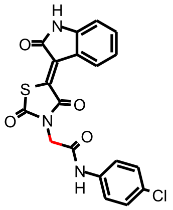 [*]C[*]	0.203

ECFP_6	-817402818	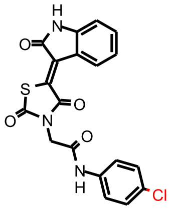 [*]Cl	0.129
ECFP_6	-1897341097	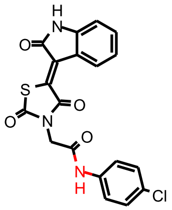 [*]N[*]	0.0284
**Top Features for negative contribution**			
**Fingerprint**	**Bit/Smiles**	**Feature Structure**	**Score**
ECFP_6	2106656448	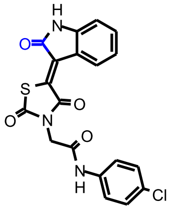 [*]C(=O)[*]	-0.275
ECFP_6	1996767644	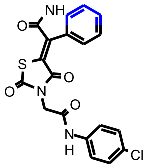 [*][c](:[*]):[cH]:[cH]:[*]	-0.251
ECFP_6	642810091	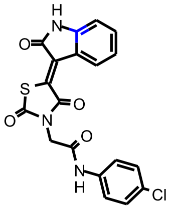 [*][c](:[*]):[*]	-0.247

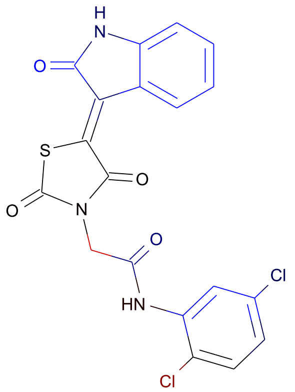

$C_{19}H_{11}Cl_2N_3O_4S$
Molecular Weight: 448.27933
ALogP: 3.028
Rotatable Bonds: 3
Acceptors: 5
Donors: 2

Model Prediction

Prediction: 71.4
Unit: mg/kg_body_weight/day
Mahalanobis Distance: 13.1
Mahalanobis Distance p-value: 6.7e-008

Mahalanobis Distance: The Mahalanobis distance (MD) is a generalization of the Euclidean distance that accounts for correlations among the X properties. It is calculated as the distance to the center of the training data. The larger the MD, the less trustworthy the prediction.
Mahalanobis Distance p-value: The p-value gives the fraction of training data with an MD greater than or equal to the one for the given sample, assuming normally distributed data. The smaller the p-value, the less trustworthy the prediciton. For highly non-normal X properties (e.g., fingerprints), the MD p-value is wildly inaccurate.

Structural Similar Compounds

Name	Ochratoxin A	542	C.I. pigment red 3
Structure	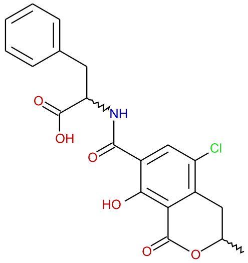	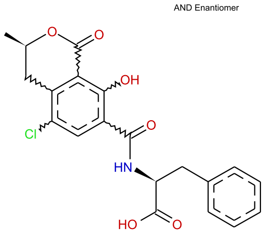	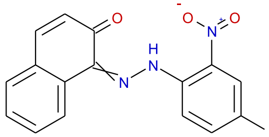
Actual Endpoint (-log C)	4.79932	4.79932	0.937339
Predicted Endpoint (-log C)	3.6353	3.6353	3.17837
Distance	0.657	0.657	0.727
Reference	CPDB	CPDB	CPDB

Model Applicability

Unknown features are fingerprint features in the query molecule, but not found or appearing too infrequently in the training set.

1. All properties and OPS components are within expected ranges.
2. Unknown ECFP_2 feature: -631778390: [*]C(=C1S[*][*]C1=[*])[*]
3. Unknown ECFP_2 feature: -661097313: [*]CN1C(=[*])[*][*]C1=[*]
4. Unknown ECFP_2 feature: -37698365: [*]N([*])CC(=[*])[*]

Feature Contribution

Top features for positive contribution

Fingerprint	Bit/Smiles	Feature Structure	Score
ECFP_6	1559650422	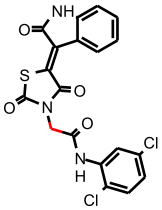 [*]C[*]	0.203

ECFP_6	-817402818	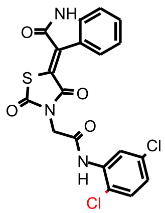 [*]Cl	0.129
ECFP_6	-1897341097	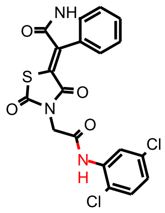 [*]N[*]	0.0284

Top Features for negative contribution

Fingerprint	Bit/Smiles	Feature Structure	Score
ECFP_6	2106656448	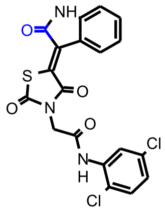 [*]C(=O)[*]	-0.275
ECFP_6	1996767644	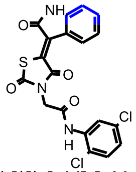 [*][c](:[*]):[cH]:[cH]:[*]	-0.251
ECFP_6	642810091	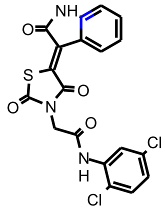 [*][c](:[*]):[*]	-0.247

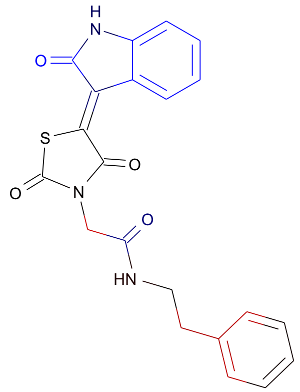

$C_{21}H_{17}N_3O_4S$
Molecular Weight: 407.44237
ALogP: 2.027
Rotatable Bonds: 5
Acceptors: 5
Donors: 2

Model Prediction

Prediction: 59.4
Unit: mg/kg_body_weight/day
Mahalanobis Distance: 11.8
Mahalanobis Distance p-value: 4.61e-005

Mahalanobis Distance: The Mahalanobis distance (MD) is a generalization of the Euclidean distance that accounts for correlations among the X properties. It is calculated as the distance to the center of the training data. The larger the MD, the less trustworthy the prediction.
Mahalanobis Distance p-value: The p-value gives the fraction of training data with an MD greater than or equal to the one for the given sample, assuming normally distributed data. The smaller the p-value, the less trustworthy the prediciton. For highly non-normal X properties (e.g., fingerprints), the MD p-value is wildly inaccurate.

Structural Similar Compounds

Name	Ochratoxin A	542	470
Structure	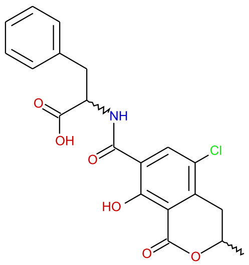	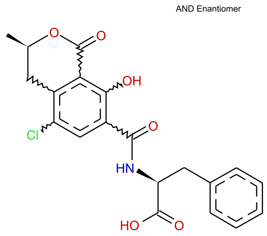	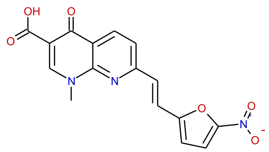
Actual Endpoint (-log C)	4.79932	4.79932	4.62839
Predicted Endpoint (-log C)	3.6353	3.6353	3.93264
Distance	0.641	0.641	0.707
Reference	CPDB	CPDB	CPDB

Model Applicability

Unknown features are fingerprint features in the query molecule, but not found or appearing too infrequently in the training set.

1. OPS PC22 out of range. Value: 3.9887. Training min, max, SD, explained variance: -3.1587, 3.8589, 1.086, 0.0147.
2. Unknown ECFP_2 feature: -631778390: [*]C(=C1S[*][*]C1=[*])[*]
3. Unknown ECFP_2 feature: -661097313: [*]CN1C(=[*])[*][*]C1=[*]
4. Unknown ECFP_2 feature: -37698365: [*]N([*])CC(=[*])[*]

Feature Contribution

Top features for positive contribution

Fingerprint	Bit/Smiles	Feature Structure	Score
ECFP_6	1559650422	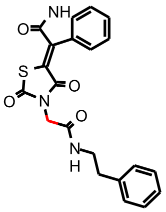 [*]C[*]	0.203

ECFP_6	-2024255407	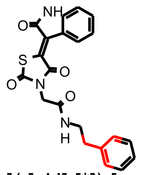<br>[*]C[c](:[cH]:[*]):[c<br>H]:[*]	0.172
ECFP_6	-1897341097	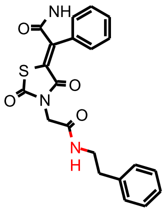<br>[*]N[*]	0.0284
**Top Features for negative contribution**			
**Fingerprint**	**Bit/Smiles**	**Feature Structure**	**Score**
ECFP_6	2106656448	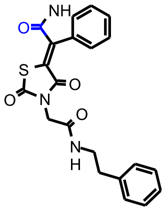<br>[*]C(=O)[*]	-0.275
ECFP_6	1996767644	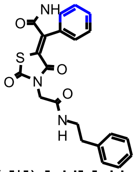<br>[*][c](:[*]):[cH]:[cH<br>]:[*]	-0.251
ECFP_6	642810091	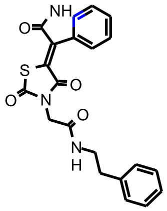<br>[*][c](:[*]):[*]	-0.247

Sorafenib

TOPKAT_Carcinogenic_Potency_TD50_Mouse

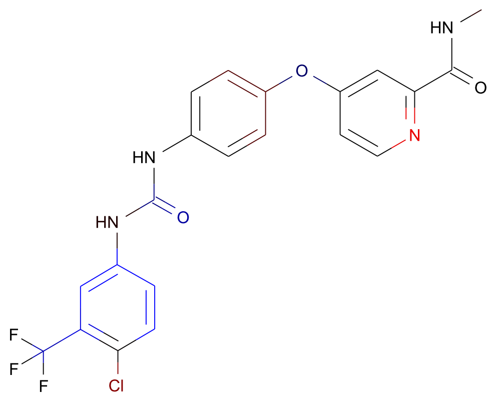

$C_{21}H_{16}ClF_3N_4O_3$

Molecular Weight: 464.82494

ALogP: 4.175

Rotatable Bonds: 6

Acceptors: 4

Donors: 3

Model Prediction

Prediction: 19.2

Unit: mg/kg_body_weight/day

Mahalanobis Distance: 12.4

Mahalanobis Distance p-value: 2.94e-006

Mahalanobis Distance: The Mahalanobis distance (MD) is a generalization of the Euclidean distance that accounts for correlations among the X properties. It is calculated as the distance to the center of the training data. The larger the MD, the less trustworthy the prediction.
Mahalanobis Distance p-value: The p-value gives the fraction of training data with an MD greater than or equal to the one for the given sample, assuming normally distributed data. The smaller the p-value, the less trustworthy the prediciton. For highly non-normal X properties (e.g., fingerprints), the MD p-value is wildly inaccurate.

Structural Similar Compounds

Name	Ochratoxin A	542	4-Chloro-6-(2,3-xylidino)-2-pyri-mi-dinylthio(N-b-hydroxy-ethyl) acetamide
Structure	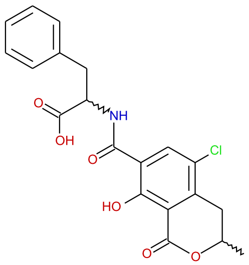	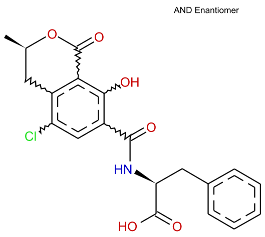	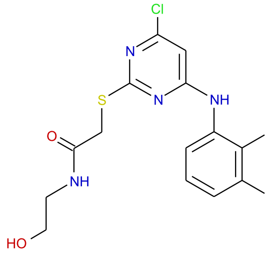
Actual Endpoint (-log C)	4.79932	4.79932	3.91517
Predicted Endpoint (-log C)	3.6353	3.6353	3.92186
Distance	0.718	0.718	0.738
Reference	CPDB	CPDB	CPDB

Model Applicability

Unknown features are fingerprint features in the query molecule, but not found or appearing too infrequently in the training set.

1. All properties and OPS components are within expected ranges.
2. Unknown ECFP_2 feature: 1338334141: [*]C(=[*])NC
3. Unknown ECFP_2 feature: 1413420509: [*]C(=[*])[c](:n:[*]):c:[*]

Feature Contribution

Top features for positive contribution

Fingerprint	Bit/Smiles	Feature Structure	Score
ECFP_6	655739385	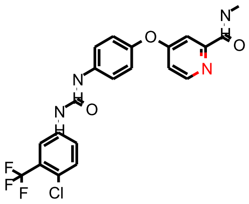 [*]:n:[*]	0.229

ECFP_6	-817402818	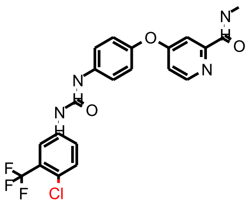<br>[*]Cl	0.129
ECFP_6	-176455838	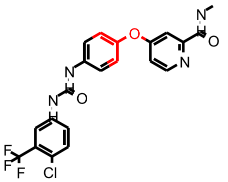<br>[*]O[c](:[cH]:[*]):[c<br>H]:[*]	0.0818

Top Features for negative contribution

Fingerprint	Bit/Smiles	Feature Structure	Score
ECFP_6	1996767644	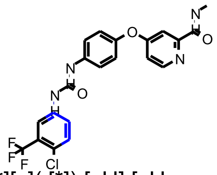<br>[*][c](:[*]):[cH]:[cH<br>]:[*]	-0.251
ECFP_6	642810091	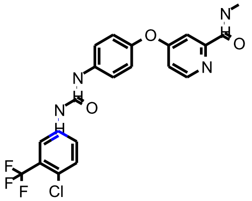<br>[*][c](:[*]):[*]	-0.247
ECFP_6	-182236392	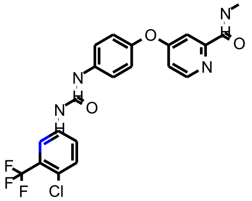<br>[*]:[cH]:[*]	-0.232

Sunitinib

TOPKAT_Carcinogenic_Potency_TD50_Mouse

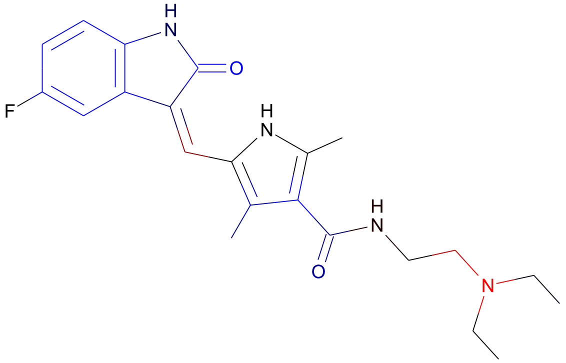

$C_{22}H_{27}FN_4O_2$

Molecular Weight: 398.47378

ALogP: 2.997

Rotatable Bonds: 7

Acceptors: 3

Donors: 3

Structural Similar Compounds

Name	455	4-Chloro-6-(2,3-xylidino)-2-pyri-mi-dinylthio(N-b-hydroxy-ethyl) acetamide	542
Structure	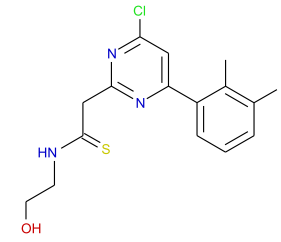	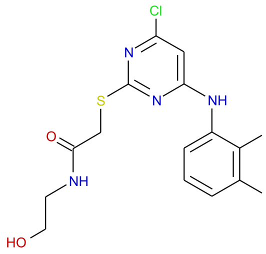	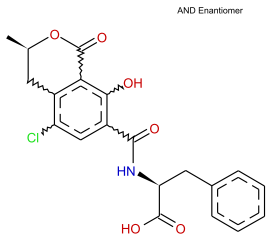
Actual Endpoint (-log C)	3.87681	3.91517	4.79932
Predicted Endpoint (-log C)	3.77582	3.92186	3.6353
Distance	0.676	0.729	0.751
Reference	CPDB	CPDB	CPDB

Model Prediction

Prediction: 33.3

Unit: mg/kg_body_weight/day

Mahalanobis Distance: 13.2

Mahalanobis Distance p-value: 5.11e-008

Mahalanobis Distance: The Mahalanobis distance (MD) is a generalization of the Euclidean distance that accounts for correlations among the X properties. It is calculated as the distance to the center of the training data. The larger the MD, the less trustworthy the prediction.
Mahalanobis Distance p-value: The p-value gives the fraction of training data with an MD greater than or equal to the one for the given sample, assuming normally distributed data. The smaller the p-value, the less trustworthy the prediciton. For highly non-normal X properties (e.g., fingerprints), the MD p-value is wildly inaccurate.

Model Applicability

Unknown features are fingerprint features in the query molecule, but not found or appearing too infrequently in the training set.

1. All properties and OPS components are within expected ranges.
2. Unknown ECFP_2 feature: -1658273810: [*]C(=[*])[c]1:[c]([*]):[*]:[*]:[c]:1[*]
3. Unknown ECFP_2 feature: 980271847: [*][c]1:[*]:[*]:[nH]:[c]:1C=[*]
4. Unknown ECFP_2 feature: 1791989338: [*][c]1:[*]:[*]:[nH]:[c]:1C
5. Unknown ECFP_2 feature: 1718013682: [*]\C=C\1/C(=[*])[*][*]:[c]1:[*]
6. Unknown ECFP_2 feature: 1182722866: [*]C(=CC(=[*])[*])[*]

Feature Contribution

Top features for positive contribution			
**Fingerprint**	**Bit/Smiles**	**Feature Structure**	**Score**

ECFP_6	-1072294614	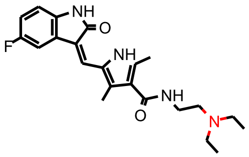 [*]N([*])[*]	0.428
ECFP_6	1559650422	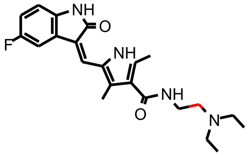 [*]C[*]	0.203
ECFP_6	-1925046727	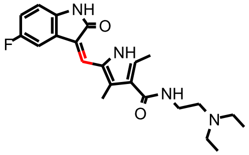 [*]C=[*]	0.145
**Top Features for negative contribution**			
**Fingerprint**	**Bit/Smiles**	**Feature Structure**	**Score**
ECFP_6	2106656448	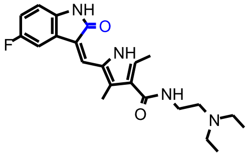 [*]C(=O)[*]	-0.275
ECFP_6	1996767644	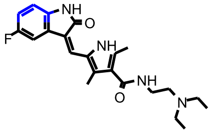 [*][c](:[*]):[cH]:[cH]:[*]	-0.251

ECFP_6	642810091	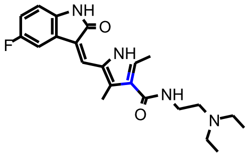<br>[*][c](:[*]):[*]	-0.247

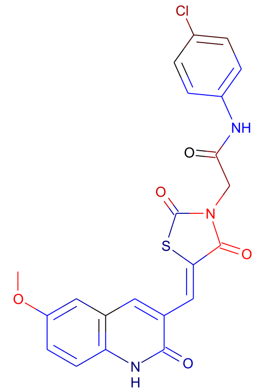

$C_{22}H_{16}ClN_3O_5S$
Molecular Weight: 469.89753
ALogP: 2.815
Rotatable Bonds: 5
Acceptors: 6
Donors: 2

Model Prediction

Prediction: 15
Unit: mg/kg_body_weight/day
Mahalanobis Distance: 15.3
Mahalanobis Distance p-value: 3.43e-012

Mahalanobis Distance: The Mahalanobis distance (MD) is a generalization of the Euclidean distance that accounts for correlations among the X properties. It is calculated as the distance to the center of the training data. The larger the MD, the less trustworthy the prediction.
Mahalanobis Distance p-value: The p-value gives the fraction of training data with an MD greater than or equal to the one for the given sample, assuming normally distributed data. The smaller the p-value, the less trustworthy the prediciton. For highly non-normal X properties (e.g., fingerprints), the MD p-value is wildly inaccurate.

Structural Similar Compounds

Name	Ochratoxin A	542	Salicylazosulfapyridine
Structure	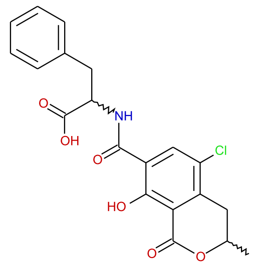	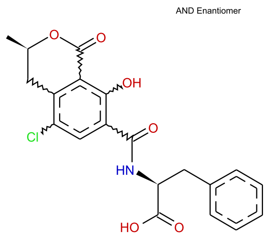	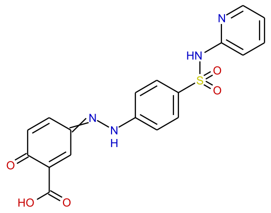
Actual Endpoint (-log C)	6.47264	6.59334	2.39891
Predicted Endpoint (-log C)	5.06501	5.06501	3.17598
Distance	0.646	0.646	0.688
Reference	CPDB	CPDB	CPDB

Model Applicability

Unknown features are fingerprint features in the query molecule, but not found or appearing too infrequently in the training set.

1. OPS PC7 out of range. Value: -5.991. Training min, max, SD, explained variance: -5.0422, 6.1749, 1.868, 0.0335.
2. OPS PC16 out of range. Value: -3.9626. Training min, max, SD, explained variance: -3.6111, 5.345, 1.406, 0.0190.

Feature Contribution

Top features for positive contribution

Fingerprint	Bit/Smiles	Feature Structure	Score
FCFP_6	136627117	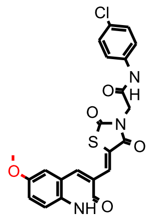 [*]OC	0.69

FCFP_6	565998553	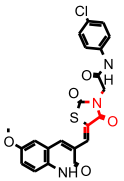 [*]N1[*][*]C(=[*])C1=O	0.357
FCFP_6	1	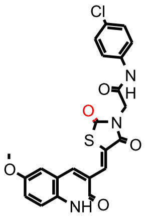 [*]=O	0.234
**Top Features for negative contribution**			
**Fingerprint**	**Bit/Smiles**	**Feature Structure**	**Score**
FCFP_6	451847724	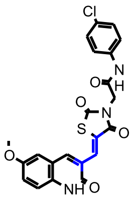 [*]C(=CC(=[*])[*])[*]	-0.436
FCFP_6	436886043	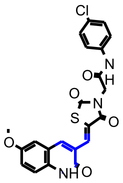 [*]\C=C(\C=[*])/C(=[*])[*]	-0.383
FCFP_6	16	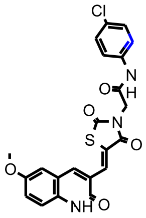 [*]:[cH]:[*]	-0.354

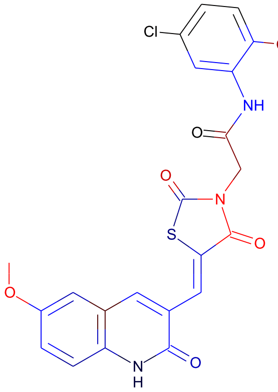

$C_{22}H_{15}Cl_2N_3O_5S$
Molecular Weight: 504.3426
ALogP: 3.479
Rotatable Bonds: 5
Acceptors: 6
Donors: 2

Model Prediction

Prediction: 13.6
Unit: mg/kg_body_weight/day
Mahalanobis Distance: 15.3
Mahalanobis Distance p-value: 3.4e-012

Mahalanobis Distance: The Mahalanobis distance (MD) is a generalization of the Euclidean distance that accounts for correlations among the X properties. It is calculated as the distance to the center of the training data. The larger the MD, the less trustworthy the prediction.
Mahalanobis Distance p-value: The p-value gives the fraction of training data with an MD greater than or equal to the one for the given sample, assuming normally distributed data. The smaller the p-value, the less trustworthy the prediciton. For highly non-normal X properties (e.g., fingerprints), the MD p-value is wildly inaccurate.

Structural Similar Compounds

Name	Ochratoxin A	542	C.I. direct brown 95
Structure	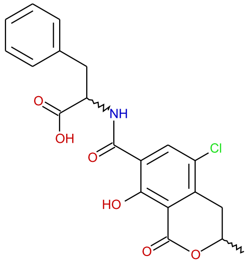	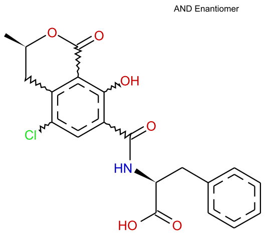	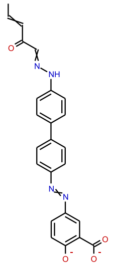
Actual Endpoint (-log C)	6.47264	6.59334	5.31387
Predicted Endpoint (-log C)	5.06501	5.06501	4.30266
Distance	0.666	0.666	0.717
Reference	CPDB	CPDB	CPDB

Model Applicability

Unknown features are fingerprint features in the query molecule, but not found or appearing too infrequently in the training set.

1. OPS PC7 out of range. Value: -6.0165. Training min, max, SD, explained variance: -5.0422, 6.1749, 1.868, 0.0335.
2. OPS PC16 out of range. Value: -3.9832. Training min, max, SD, explained variance: -3.6111, 5.345, 1.406, 0.0190.

Feature Contribution

Top features for positive contribution

Fingerprint	Bit/Smiles	Feature Structure	Score
FCFP_6	136627117	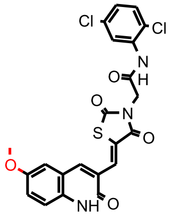 [*]OC	0.69

FCFP_6	565998553	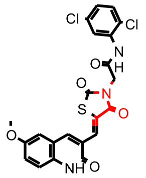 [*]N1[*][*]C(=[*])C1=O	0.357
FCFP_6	1	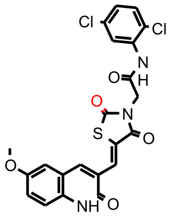 [*]=O	0.234
**Top Features for negative contribution**			
**Fingerprint**	**Bit/Smiles**	**Feature Structure**	**Score**
FCFP_6	451847724	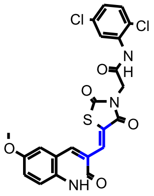 [*]C(=CC(=[*])[*])[*]	-0.436
FCFP_6	436886043	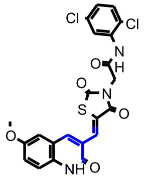 [*]\C=C(\C=[*])/C(=[*])[*]	-0.383
FCFP_6	16	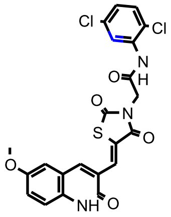 [*]:[cH]:[*]	-0.354

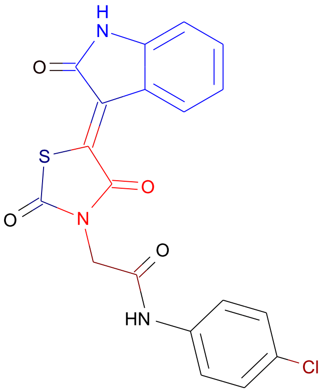

$C_{19}H_{12}ClN_3O_4S$
Molecular Weight: 413.83428
ALogP: 2.363
Rotatable Bonds: 3
Acceptors: 5
Donors: 2

Model Prediction

Prediction: 14.3
Unit: mg/kg_body_weight/day
Mahalanobis Distance: 11.6
Mahalanobis Distance p-value: 0.0028

Mahalanobis Distance: The Mahalanobis distance (MD) is a generalization of the Euclidean distance that accounts for correlations among the X properties. It is calculated as the distance to the center of the training data. The larger the MD, the less trustworthy the prediction.
Mahalanobis Distance p-value: The p-value gives the fraction of training data with an MD greater than or equal to the one for the given sample, assuming normally distributed data. The smaller the p-value, the less trustworthy the prediciton. For highly non-normal X properties (e.g., fingerprints), the MD p-value is wildly inaccurate.

Structural Similar Compounds

Name	Ochratoxin A	542	1,2-Dihydro-2-(5-nitro-2-thi-enyl) quinazolin-4(3H)-one
Structure	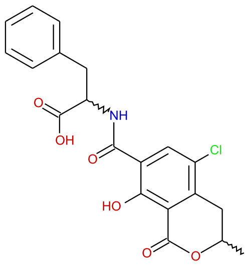	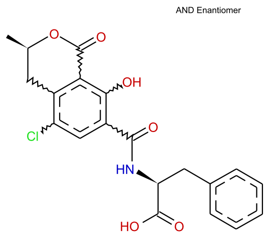	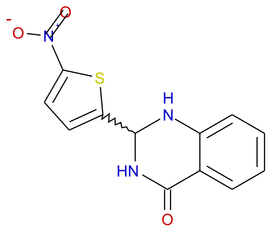
Actual Endpoint (-log C)	6.47264	6.59334	5.25509
Predicted Endpoint (-log C)	5.06501	5.06501	3.89291
Distance	0.628	0.628	0.651
Reference	CPDB	CPDB	CPDB

Model Applicability

Unknown features are fingerprint features in the query molecule, but not found or appearing too infrequently in the training set.

1. All properties and OPS components are within expected ranges.

Feature Contribution

Top features for positive contribution			
**Fingerprint**	**Bit/Smiles**	**Feature Structure**	**Score**
FCFP_6	565998553	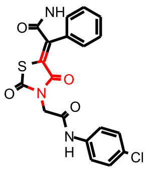 [*]N1[*][*]C(=[*])C1=O	0.357

FCFP_6	1	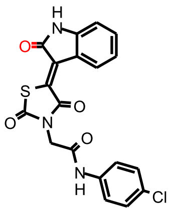<br>[*]=O	0.234
FCFP_6	32	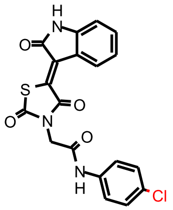<br>[*]Cl	0.154
**Top Features for negative contribution**			
**Fingerprint**	**Bit/Smiles**	**Feature Structure**	**Score**
FCFP_6	991735244	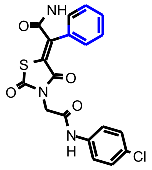<br>[*][c]1:[*]:[cH]:[cH]:[cH]:[cH]:1	-0.422
FCFP_6	16	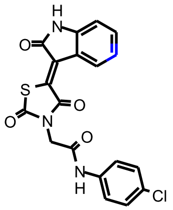<br>[*]:[cH]:[*]	-0.354
FCFP_6	590925877	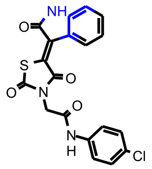<br>[*]N[c](:[cH]:[*]):[cH]:[*]	-0.323

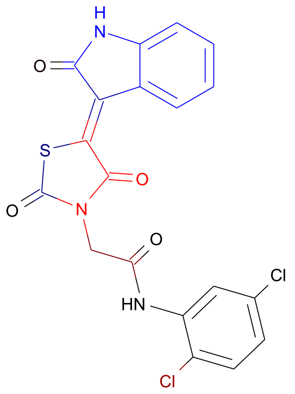

$C_{19}H_{11}Cl_2N_3O_4S$
Molecular Weight: 448.27933
ALogP: 3.028
Rotatable Bonds: 3
Acceptors: 5
Donors: 2

Model Prediction

Prediction: 13
Unit: mg/kg_body_weight/day
Mahalanobis Distance: 11.5
Mahalanobis Distance p-value: 0.00327

Mahalanobis Distance: The Mahalanobis distance (MD) is a generalization of the Euclidean distance that accounts for correlations among the X properties. It is calculated as the distance to the center of the training data. The larger the MD, the less trustworthy the prediction.
Mahalanobis Distance p-value: The p-value gives the fraction of training data with an MD greater than or equal to the one for the given sample, assuming normally distributed data. The smaller the p-value, the less trustworthy the prediciton. For highly non-normal X properties (e.g., fingerprints), the MD p-value is wildly inaccurate.

Structural Similar Compounds

Name	Ochratoxin A	542	1,2-Dihydro-2-(5-nitro-2-thi-enyl) quinazolin-4(3H)-one
Structure	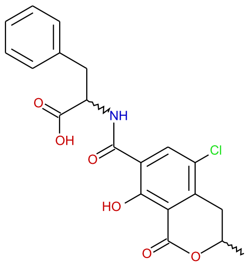	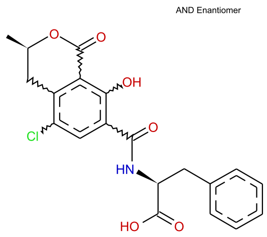	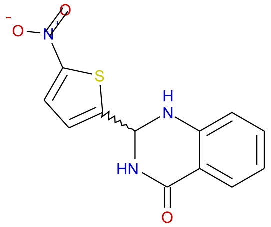
Actual Endpoint (-log C)	6.47264	6.59334	5.25509
Predicted Endpoint (-log C)	5.06501	5.06501	3.89291
Distance	0.628	0.628	0.688
Reference	CPDB	CPDB	CPDB

Model Applicability

Unknown features are fingerprint features in the query molecule, but not found or appearing too infrequently in the training set.

1. All properties and OPS components are within expected ranges.

Feature Contribution

Top features for positive contribution			
Fingerprint	Bit/Smiles	Feature Structure	Score
FCFP_6	565998553	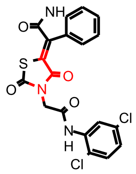 [*]N1[*][*]C(=[*])C1=O	0.357

FCFP_6	1	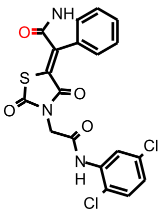 [*]=O	0.234
FCFP_6	32	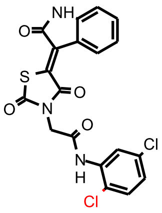 [*]Cl	0.154

Top Features for negative contribution

Fingerprint	Bit/Smiles	Feature Structure	Score
FCFP_6	991735244	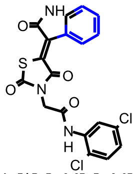 [*][c]1:[*]:[cH]:[cH]:[cH]:[cH]:1	-0.422
FCFP_6	16	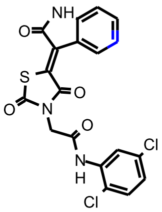 [*]:[cH]:[*]	-0.354
FCFP_6	590925877	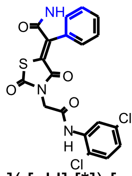 [*]N[c](:[cH]:[*]):[cH]:[*]	-0.323

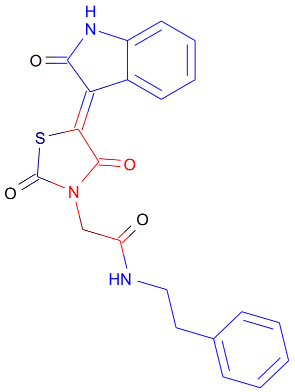

$C_{21}H_{17}N_3O_4S$

Molecular Weight: 407.44237

ALogP: 2.027

Rotatable Bonds: 5

Acceptors: 5

Donors: 2

Structural Similar Compounds

Name	Ochratoxin A	542	4,4´-Sulfonylbisacetanilide
Structure	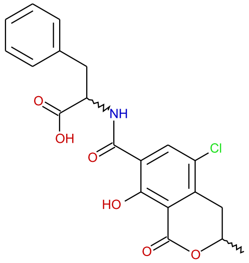	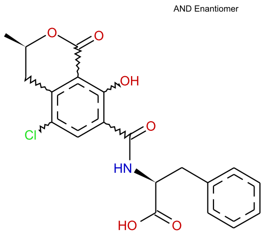	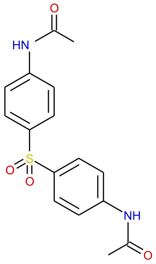
Actual Endpoint (-log C)	6.47264	6.59334	3.77655
Predicted Endpoint (-log C)	5.06501	5.06501	3.55337
Distance	0.608	0.608	0.669
Reference	CPDB	CPDB	CPDB

Model Prediction

Prediction: 121

Unit: mg/kg_body_weight/day

Mahalanobis Distance: 13

Mahalanobis Distance p-value: 5.66e-006

Mahalanobis Distance: The Mahalanobis distance (MD) is a generalization of the Euclidean distance that accounts for correlations among the X properties. It is calculated as the distance to the center of the training data. The larger the MD, the less trustworthy the prediction.
Mahalanobis Distance p-value: The p-value gives the fraction of training data with an MD greater than or equal to the one for the given sample, assuming normally distributed data. The smaller the p-value, the less trustworthy the prediciton. For highly non-normal X properties (e.g., fingerprints), the MD p-value is wildly inaccurate.

Model Applicability

Unknown features are fingerprint features in the query molecule, but not found or appearing too infrequently in the training set.

1. All properties and OPS components are within expected ranges.

Feature Contribution

Top features for positive contribution			
**Fingerprint**	**Bit/Smiles**	**Feature Structure**	**Score**
FCFP_6	565998553	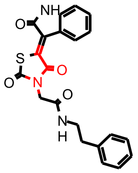 [*]N1[*][*]C(=[*])C1=O	0.357

FCFP_6	1	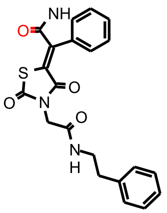 [*]=O	0.234
FCFP_6	-885550502	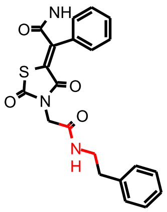 [*]CNC(=[*])[*]	0.229

Top Features for negative contribution

Fingerprint	Bit/Smiles	Feature Structure	Score
FCFP_6	-1272709286	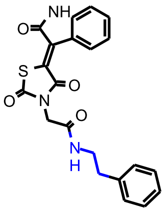 [*]CCN[*]	-0.526
FCFP_6	991735244	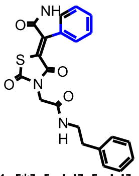 [*][c]1:[*]:[cH]:[cH]:[cH]:[cH]:1	-0.422
FCFP_6	-2093839777	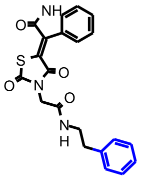 [*][c]1:[cH]:[cH]:[cH]:[cH]:[cH]:1	-0.378

Sorafenib

TOPKAT_Carcinogenic_Potency_TD50_Rat

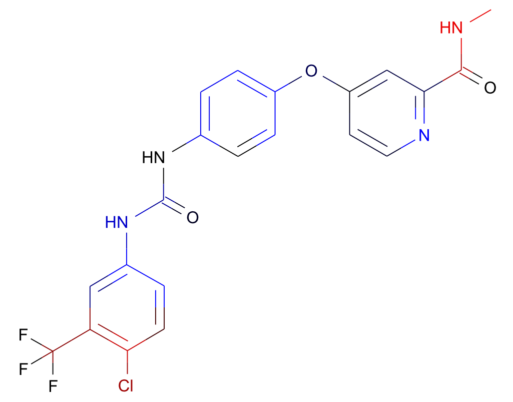

$C_{21}H_{16}ClF_3N_4O_3$

Molecular Weight: 464.82494

ALogP: 4.175

Rotatable Bonds: 6

Acceptors: 4

Donors: 3

Model Prediction

Prediction: 14.2

Unit: mg/kg_body_weight/day

Mahalanobis Distance: 20.4

Mahalanobis Distance p-value: 9.56e-031

Mahalanobis Distance: The Mahalanobis distance (MD) is a generalization of the Euclidean distance that accounts for correlations among the X properties. It is calculated as the distance to the center of the training data. The larger the MD, the less trustworthy the prediction.

Mahalanobis Distance p-value: The p-value gives the fraction of training data with an MD greater than or equal to the one for the given sample, assuming normally distributed data. The smaller the p-value, the less trustworthy the prediciton. For highly non-normal X properties (e.g., fingerprints), the MD p-value is wildly inaccurate.

Structural Similar Compounds

Name	Fluvastatin	913	Ochratoxin A
Structure	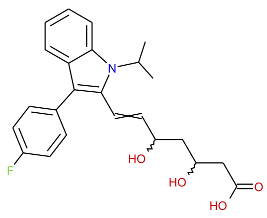	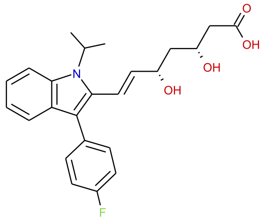	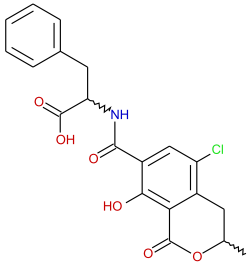
Actual Endpoint (-log C)	3.51742	3.51742	6.47264
Predicted Endpoint (-log C)	5.41573	5.41573	5.06501
Distance	0.597	0.597	0.666
Reference	CPDB	CPDB	CPDB

Model Applicability

Unknown features are fingerprint features in the query molecule, but not found or appearing too infrequently in the training set.

1. All properties and OPS components are within expected ranges.
2. Unknown FCFP_2 feature: -1029533685: [*]:[c](:[*])C(F)(F)F

Feature Contribution

Top features for positive contribution

Fingerprint	Bit/Smiles	Feature Structure	Score
FCFP_6	1	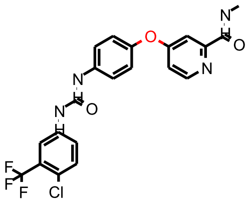 [*]=O	0.234

FCFP_6	-885550502	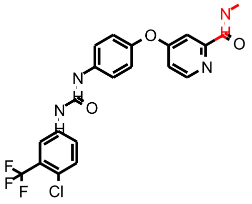 [*]CNC(=[*])[*]	0.229
FCFP_6	32	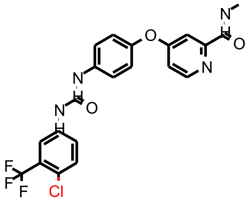 [*]Cl	0.154
**Top Features for negative contribution**			
**Fingerprint**	**Bit/Smiles**	**Feature Structure**	**Score**
FCFP_6	16	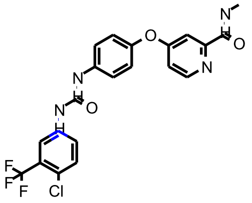 [*]:[cH]:[*]	-0.354
FCFP_6	590925877	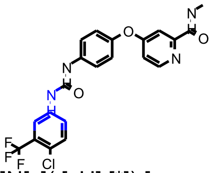 [*]N[c](:[cH]:[*]):[cH]:[*]	-0.323
FCFP_6	1674451008	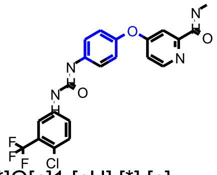 [*]O[c]1:[cH]:[*]:[c]([*]):[cH]:[cH]:1	-0.233

Sunitinib

TOPKAT_Carcinogenic_Potency_TD50_Rat

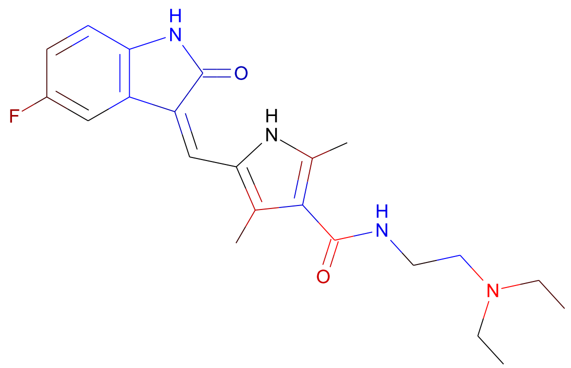

$C_{22}H_{27}FN_4O_2$
Molecular Weight: 398.47378
ALogP: 2.997
Rotatable Bonds: 7
Acceptors: 3
Donors: 3

Model Prediction

Prediction: 4.13
Unit: mg/kg_body_weight/day
Mahalanobis Distance: 14
Mahalanobis Distance p-value: 2.69e-008

Mahalanobis Distance: The Mahalanobis distance (MD) is a generalization of the Euclidean distance that accounts for correlations among the X properties. It is calculated as the distance to the center of the training data. The larger the MD, the less trustworthy the prediction.
Mahalanobis Distance p-value: The p-value gives the fraction of training data with an MD greater than or equal to the one for the given sample, assuming normally distributed data. The smaller the p-value, the less trustworthy the prediciton. For highly non-normal X properties (e.g., fingerprints), the MD p-value is wildly inaccurate.

Structural Similar Compounds

Name	913	Fluvastatin	542
Structure	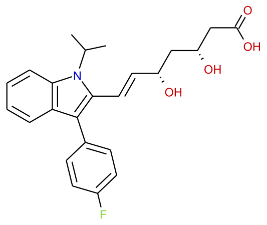	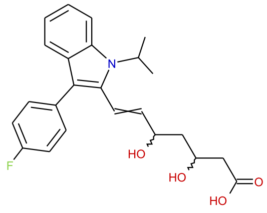	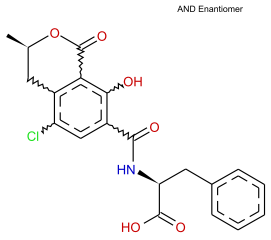
Actual Endpoint (-log C)	3.51742	3.51742	6.59334
Predicted Endpoint (-log C)	5.41573	5.41573	5.06501
Distance	0.636	0.636	0.671
Reference	CPDB	CPDB	CPDB

Model Applicability

Unknown features are fingerprint features in the query molecule, but not found or appearing too infrequently in the training set.

1. OPS PC18 out of range. Value: 6.1602. Training min, max, SD, explained variance: -4.1023, 4.8669, 1.305, 0.0164.

Feature Contribution

Top features for positive contribution			
**Fingerprint**	**Bit/Smiles**	**Feature Structure**	**Score**
FCFP_6	9	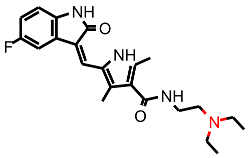 [*]N([*])[*]	0.385

FCFP_6	-587569116	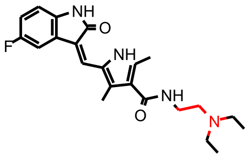 [*]CCN([*])[*]	0.319
FCFP_6	1	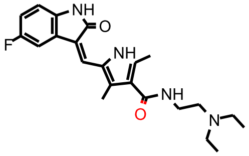 [*]=O	0.234
**Top Features for negative contribution**			
**Fingerprint**	**Bit/Smiles**	**Feature Structure**	**Score**
FCFP_6	-1272709286	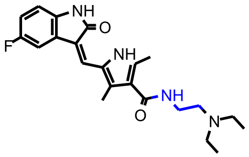 [*]CCN[*]	-0.526
FCFP_6	16	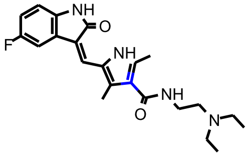 [*]:[cH]:[*]	-0.354
FCFP_6	590925877	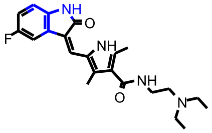 [*]N[c](:[cH]:[*]):[cH]:[*]	-0.323

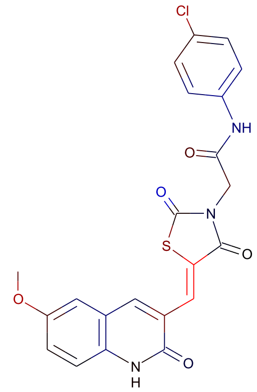

$C_{22}H_{16}ClN_3O_5S$
Molecular Weight: 469.89753
ALogP: 2.815
Rotatable Bonds: 5
Acceptors: 6
Donors: 2

Model Prediction

Prediction: 0.00186
Unit: g/kg_body_weight
Mahalanobis Distance: 32.5
Mahalanobis Distance p-value: 3.17e-029

Mahalanobis Distance: The Mahalanobis distance (MD) is a generalization of the Euclidean distance that accounts for correlations among the X properties. It is calculated as the distance to the center of the training data. The larger the MD, the less trustworthy the prediction.
Mahalanobis Distance p-value: The p-value gives the fraction of training data with an MD greater than or equal to the one for the given sample, assuming normally distributed data. The smaller the p-value, the less trustworthy the prediciton. For highly non-normal X properties (e.g., fingerprints), the MD p-value is wildly inaccurate.

Structural Similar Compounds

Name	GLIPIZIDE	GLYBURIDE	CHLORSULFURON
Structure	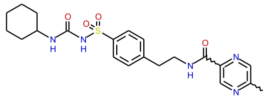	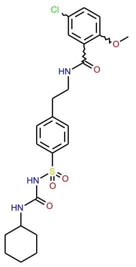	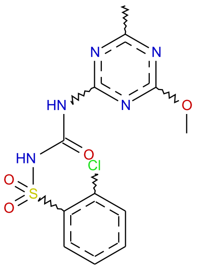
Actual Endpoint (-log C)	3.94991	4.21661	4.15566
Predicted Endpoint (-log C)	3.95594	4.21035	3.79771
Distance	0.604	0.613	0.643
Reference	NDA-17583	UPJ-26452	EPA COVER SHEET 0027;880301;(1)

Model Applicability

Unknown features are fingerprint features in the query molecule, but not found or appearing too infrequently in the training set.

1. OPS PC22 out of range. Value: -6.8151. Training min, max, SD, explained variance: -4.3287, 5.3383, 1.588, 0.0110.
2. Unknown ECFP_6 feature: -154530762: [*]N[*]
3. Unknown ECFP_6 feature: 912478223: [*]S[*]
4. Unknown ECFP_6 feature: 99947387: [*]:[c](:[*])Cl
5. Unknown ECFP_6 feature: -176494269: [*]:[cH]:[c](Cl):[cH]:[*]
6. Unknown ECFP_6 feature: -177077903: [*]N[c](:[cH]:[*]):[cH]:[*]
7. Unknown ECFP_6 feature: 1335833675: [*]N[c](:[cH]:[*]):[c]([*]):[*]
8. Unknown ECFP_6 feature: 1336666212: [*][c](:[*]):[c](C=[*]):[cH]:[*]
9. Unknown ECFP_6 feature: 464808839: [*]C(=C[c](:[*]):[*])[*]
10. Unknown ECFP_6 feature: 2131425032: [*]\C=C(\C=[*])/C(=[*])[*]
11. Unknown ECFP_6 feature: 1298725959: [*]NC(=O)C(=[*])[*]
12. Unknown ECFP_6 feature: -1699286547: [*]C(=[*])N[c](:[*]):[*]
13. Unknown ECFP_6 feature: 1182722866: [*]C(=CC(=[*])[*])[*]
14. Unknown ECFP_6 feature: 1000552169: [*]\C=C\1/S[*][*]C1=[*]
15. Unknown ECFP_6 feature: 1945129186: [*]N1[*][*]C(=[*])C1=O
16. Unknown ECFP_6 feature: -661097313: [*]CN1C(=[*])[*][*]C1=[*]
17. Unknown ECFP_6 feature: 190445529: [*]N1[*][*]SC1=O
18. Unknown ECFP_6 feature: 2122741631: [*]=C1[*][*]C(=[*])S1

19. Unknown ECFP_6 feature: -37698365: [*]N([*])CC(=[*])[*]
20. Unknown ECFP_6 feature: 1731843802: [*]CC(=O)N[*]
21. Unknown ECFP_6 feature: 1307307440: [*]:[c](:[*])OC

Feature Contribution

Top features for positive contribution			
**Fingerprint**	**Bit/Smiles**	**Feature Structure**	**Score**
FCFP_6	451847724	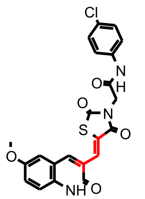 [*]C(=CC(=[*])[*])[*]	0.16
FCFP_6	-1143715940	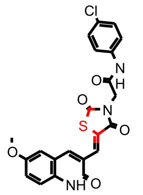 [*]=C1[*][*]C(=[*])S1	0.13
ECFP_6	1559650422	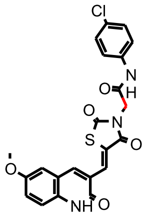 [*]C[*]	0.129

Top Features for negative contribution			
**Fingerprint**	**Bit/Smiles**	**Feature Structure**	**Score**

ECFP_6	2106656448	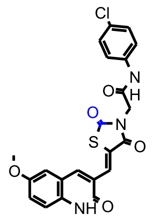 [*]C(=O)[*]	-0.11
FCFP_6	1	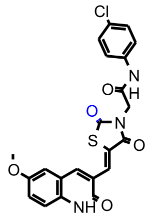 [*]=O	-0.102
ECFP_6	-1236483485	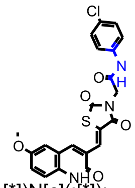 [*]C(=[*])N[c](:[*]):[*]	-0.0747

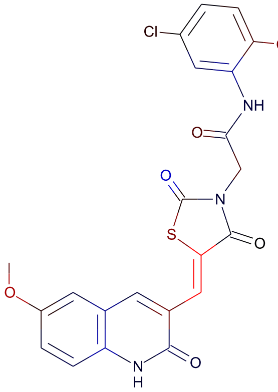

$C_{22}H_{15}Cl_2N_3O_5S$
Molecular Weight: 504.3426
ALogP: 3.479
Rotatable Bonds: 5
Acceptors: 6
Donors: 2

Model Prediction

Prediction: 0.00163
Unit: g/kg_body_weight
Mahalanobis Distance: 32.6
Mahalanobis Distance p-value: 2.73e-029

Mahalanobis Distance: The Mahalanobis distance (MD) is a generalization of the Euclidean distance that accounts for correlations among the X properties. It is calculated as the distance to the center of the training data. The larger the MD, the less trustworthy the prediction.
Mahalanobis Distance p-value: The p-value gives the fraction of training data with an MD greater than or equal to the one for the given sample, assuming normally distributed data. The smaller the p-value, the less trustworthy the prediciton. For highly non-normal X properties (e.g., fingerprints), the MD p-value is wildly inaccurate.

Structural Similar Compounds

Name	GLYBURIDE	GLIPIZIDE	CHLORSULFURON
Structure	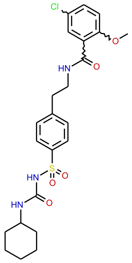	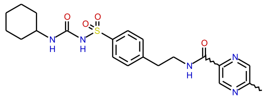	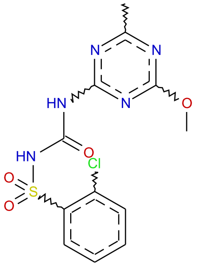
Actual Endpoint (-log C)	4.21661	3.94991	4.15566
Predicted Endpoint (-log C)	4.21035	3.95594	3.79771
Distance	0.591	0.646	0.703
Reference	UPJ-26452	NDA-17583	EPA COVER SHEET 0027;880301;(1)

Model Applicability

Unknown features are fingerprint features in the query molecule, but not found or appearing too infrequently in the training set.

1. OPS PC22 out of range. Value: -6.8257. Training min, max, SD, explained variance: -4.3287, 5.3383, 1.588, 0.0110.
2. Unknown ECFP_6 feature: -154530762: [*]N[*]
3. Unknown ECFP_6 feature: 912478223: [*]S[*]
4. Unknown ECFP_6 feature: 99947387: [*]:[c](:[*])Cl
5. Unknown ECFP_6 feature: -176494269: [*]:[cH]:[c](Cl):[cH]:[*]
6. Unknown ECFP_6 feature: 1335108269: [*]N[c](:[cH]:[*]):[c]([*]):[*]
7. Unknown ECFP_6 feature: 1335833675: [*]N[c](:[cH]:[*]):[c]([*]):[*]
8. Unknown ECFP_6 feature: 1336666212: [*][c](:[*]):[c](C=[*]):[cH]:[*]
9. Unknown ECFP_6 feature: 464808839: [*]C(=C[c](:[*]):[*])[*]
10. Unknown ECFP_6 feature: 2131425032: [*]\C=C(\C=[*])/C(=[*])[*]
11. Unknown ECFP_6 feature: 1298725959: [*]NC(=O)C(=[*])[*]
12. Unknown ECFP_6 feature: -1699286547: [*]C(=[*])N[c](:[*]):[*]
13. Unknown ECFP_6 feature: 1182722866: [*]C(=CC(=[*])[*])[*]
14. Unknown ECFP_6 feature: 1000552169: [*]\C=C\1/S[*][*]C1=[*]
15. Unknown ECFP_6 feature: 1945129186: [*]N1[*][*]C(=[*])C1=O
16. Unknown ECFP_6 feature: -661097313: [*]CN1C(=[*])[*][*]C1=[*]
17. Unknown ECFP_6 feature: 190445529: [*]N1[*][*]SC1=O
18. Unknown ECFP_6 feature: 2122741631: [*]=C1[*][*]C(=[*])S1

19. Unknown ECFP_6 feature: -37698365: [*]N([*])CC(=[*])[*]
20. Unknown ECFP_6 feature: 1731843802: [*]CC(=O)N[*]
21. Unknown ECFP_6 feature: 1307307440: [*]:[c](:[*])OC

Feature Contribution

Top features for positive contribution			
**Fingerprint**	**Bit/Smiles**	**Feature Structure**	**Score**
FCFP_6	451847724	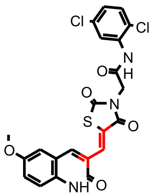 [*]C(=CC(=[*])[*])[*]	0.16
FCFP_6	-1143715940	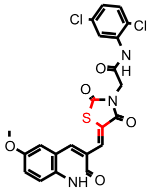 [*]=C1[*][*]C(=[*])S1	0.13
ECFP_6	1559650422	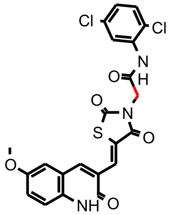 [*]C[*]	0.129

Top Features for negative contribution			
**Fingerprint**	**Bit/Smiles**	**Feature Structure**	**Score**

ECFP_6	2106656448	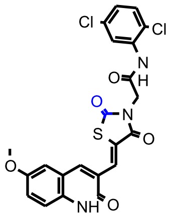 [*]C(=O)[*]	-0.11
FCFP_6	1	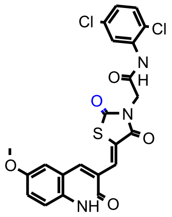 [*]=O	-0.102
ECFP_6	-1236483485	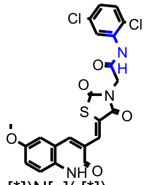 [*]C(=[*])N[c](:[*]): [*]	-0.0747

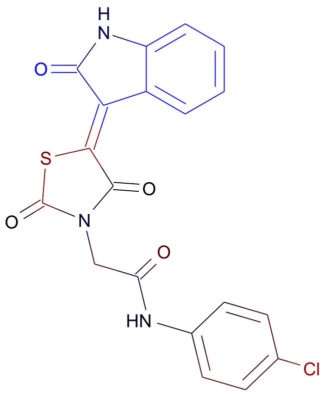

$C_{19}H_{12}ClN_3O_4S$
Molecular Weight: 413.83428
ALogP: 2.363
Rotatable Bonds: 3
Acceptors: 5
Donors: 2

Structural Similar Compounds

Name	CHLORSULFURON	PIROXICAM	DANTROLENE.NA
Structure	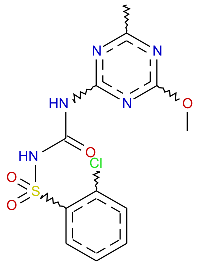	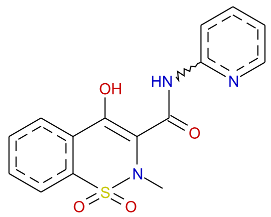	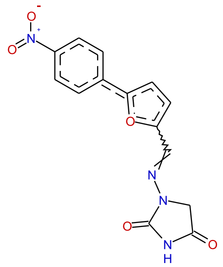
Actual Endpoint (-log C)	4.15566	5.52028	4.19625
Predicted Endpoint (-log C)	3.79771	4.06087	4.62637
Distance	0.587	0.596	0.600
Reference	EPA COVER SHEET 0027;880301;(1)	NDA-18147	NDA-17443

Model Prediction

Prediction: 0.0298
Unit: g/kg_body_weight
Mahalanobis Distance: 30.1
Mahalanobis Distance p-value: 8.84e-025

Mahalanobis Distance: The Mahalanobis distance (MD) is a generalization of the Euclidean distance that accounts for correlations among the X properties. It is calculated as the distance to the center of the training data. The larger the MD, the less trustworthy the prediction.
Mahalanobis Distance p-value: The p-value gives the fraction of training data with an MD greater than or equal to the one for the given sample, assuming normally distributed data. The smaller the p-value, the less trustworthy the prediciton. For highly non-normal X properties (e.g., fingerprints), the MD p-value is wildly inaccurate.

Model Applicability

Unknown features are fingerprint features in the query molecule, but not found or appearing too infrequently in the training set.

1. OPS PC22 out of range. Value: -4.9837. Training min, max, SD, explained variance: -4.3287, 5.3383, 1.588, 0.0110.
2. Unknown ECFP_6 feature: -154530762: [*]N[*]
3. Unknown ECFP_6 feature: 912478223: [*]S[*]
4. Unknown ECFP_6 feature: 1997021792: [*]:[cH]:[cH]:[cH]:[*]
5. Unknown ECFP_6 feature: 1335833675: [*]N[c](:[cH]:[*]):[c]([*]):[*]
6. Unknown ECFP_6 feature: -1699286547: [*]C(=[*])N[c](:[*]):[*]
7. Unknown ECFP_6 feature: 1298725959: [*]NC(=O)C(=[*])[*]
8. Unknown ECFP_6 feature: 1790105651: [*]C(=C1C(=[*])[*][*]:[c]1:[*])[*]
9. Unknown ECFP_6 feature: -631778390: [*]C(=C1S[*][*]C1=[*])[*]
10. Unknown ECFP_6 feature: 2122741631: [*]=C1[*][*]C(=[*])S1
11. Unknown ECFP_6 feature: 190445529: [*]N1[*][*]SC1=O
12. Unknown ECFP_6 feature: -661097313: [*]CN1C(=[*])[*][*]C1=[*]
13. Unknown ECFP_6 feature: 1945129186: [*]N1[*][*]C(=[*])C1=O
14. Unknown ECFP_6 feature: -37698365: [*]N([*])CC(=[*])[*]
15. Unknown ECFP_6 feature: 1731843802: [*]CC(=O)N[*]
16. Unknown ECFP_6 feature: -177077903: [*]N[c](:[cH]:[*]):[cH]:[*]
17. Unknown ECFP_6 feature: -176494269: [*]:[cH]:[c](Cl):[cH]:[*]
18. Unknown ECFP_6 feature: 99947387: [*]:[c](:[*])Cl

Feature Contribution

Top features for positive contribution			
**Fingerprint**	**Bit/Smiles**	**Feature Structure**	**Score**
FCFP_6	-1143715940	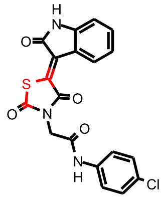<br>[*]=C1[*][*]C(=[*])S1	0.13
ECFP_6	1559650422	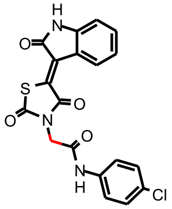<br>[*]C[*]	0.129
FCFP_6	32	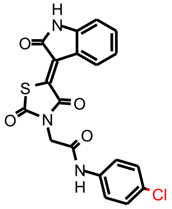<br>[*]Cl	0.101

Top Features for negative contribution			
**Fingerprint**	**Bit/Smiles**	**Feature Structure**	**Score**
FCFP_6	991735244	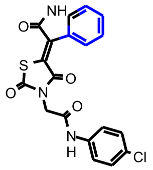<br>[*][c]1:[*]:[cH]:[cH]<br>:[cH]:[cH]:1	-0.134

ECFP_6	1564392544	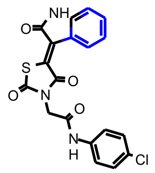 [*][c]1:[*]:[cH]:[cH]:[cH]:[cH]:1	-0.133
ECFP_6	2106656448	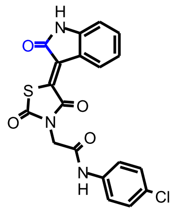 [*]C(=O)[*]	-0.11

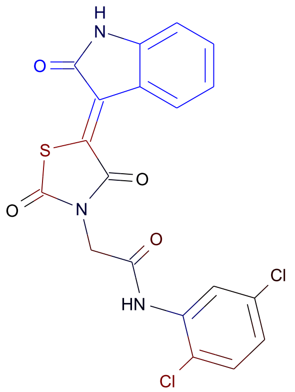

$C_{19}H_{11}Cl_2N_3O_4S$
Molecular Weight: 448.27933
ALogP: 3.028
Rotatable Bonds: 3
Acceptors: 5
Donors: 2

Model Prediction

Prediction: 0.0284
Unit: g/kg_body_weight
Mahalanobis Distance: 30.1
Mahalanobis Distance p-value: 7.98e-025

Mahalanobis Distance: The Mahalanobis distance (MD) is a generalization of the Euclidean distance that accounts for correlations among the X properties. It is calculated as the distance to the center of the training data. The larger the MD, the less trustworthy the prediction.
Mahalanobis Distance p-value: The p-value gives the fraction of training data with an MD greater than or equal to the one for the given sample, assuming normally distributed data. The smaller the p-value, the less trustworthy the prediciton. For highly non-normal X properties (e.g., fingerprints), the MD p-value is wildly inaccurate.

Structural Similar Compounds

Name	CHLORSULFURON	DANTROLENE.NA	PIROXICAM
Structure	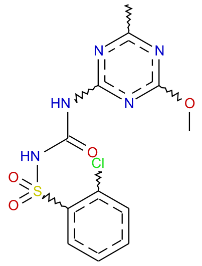	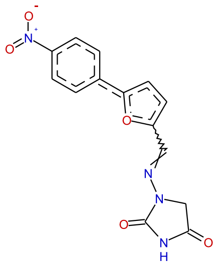	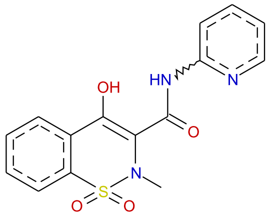
Actual Endpoint (-log C)	4.15566	4.19625	5.52028
Predicted Endpoint (-log C)	3.79771	4.62637	4.06087
Distance	0.634	0.656	0.668
Reference	EPA COVER SHEET 0027;880301;(1)	NDA-17443	NDA-18147

Model Applicability

Unknown features are fingerprint features in the query molecule, but not found or appearing too infrequently in the training set.

1. OPS PC22 out of range. Value: -4.9943. Training min, max, SD, explained variance: -4.3287, 5.3383, 1.588, 0.0110.
2. Unknown ECFP_6 feature: -154530762: [*]N[*]
3. Unknown ECFP_6 feature: 912478223: [*]S[*]
4. Unknown ECFP_6 feature: 1997021792: [*]:[cH]:[cH]:[cH]:[*]
5. Unknown ECFP_6 feature: 1335833675: [*]N[c](:[cH]:[*]):[c]([*]):[*]
6. Unknown ECFP_6 feature: -1699286547: [*]C(=[*])N[c](:[*]):[*]
7. Unknown ECFP_6 feature: 1298725959: [*]NC(=O)C(=[*])[*]
8. Unknown ECFP_6 feature: 1790105651: [*]C(=C1C(=[*])[*][*]:[c]1:[*])[*]
9. Unknown ECFP_6 feature: -631778390: [*]C(=C1S[*][*]C1=[*])[*]
10. Unknown ECFP_6 feature: 2122741631: [*]=C1[*][*]C(=[*])S1
11. Unknown ECFP_6 feature: 190445529: [*]N1[*][*]SC1=O
12. Unknown ECFP_6 feature: -661097313: [*]CN1C(=[*])[*][*]C1=[*]
13. Unknown ECFP_6 feature: 1945129186: [*]N1[*][*]C(=[*])C1=O
14. Unknown ECFP_6 feature: -37698365: [*]N([*])CC(=[*])[*]
15. Unknown ECFP_6 feature: 1731843802: [*]CC(=O)N[*]
16. Unknown ECFP_6 feature: 1335108269: [*]N[c](:[cH]:[*]):[c]([*]):[*]
17. Unknown ECFP_6 feature: -176494269: [*]:[cH]:[c](Cl):[cH]:[*]
18. Unknown ECFP_6 feature: 99947387: [*]:[c](:[*])Cl

Feature Contribution

Top features for positive contribution

Fingerprint	Bit/Smiles	Feature Structure	Score
FCFP_6	-1143715940	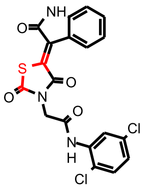 [*]=C1[*][*]C(=[*])S1	0.13
ECFP_6	1559650422	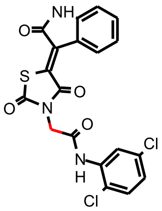 [*]C[*]	0.129
FCFP_6	32	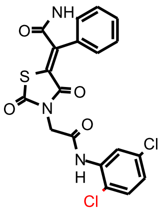 [*]Cl	0.101

Top Features for negative contribution

Fingerprint	Bit/Smiles	Feature Structure	Score
FCFP_6	991735244	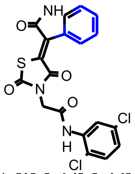 [*][c]1:[*]:[cH]:[cH]:[cH]:[cH]:1	-0.134

ECFP_6	1564392544	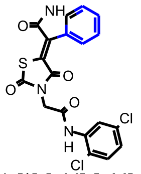 [*][c]1:[*]:[cH]:[cH]:[cH]:[cH]:1	-0.133
ECFP_6	2106656448	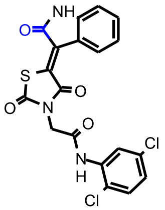 [*]C(=O)[*]	-0.11

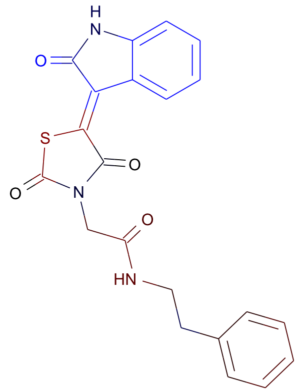

$C_{21}H_{17}N_3O_4S$
Molecular Weight: 407.44237
ALogP: 2.027
Rotatable Bonds: 5
Acceptors: 5
Donors: 2

Model Prediction

Prediction: 0.0695
Unit: g/kg_body_weight
Mahalanobis Distance: 32.5
Mahalanobis Distance p-value: 3.22e-029

Mahalanobis Distance: The Mahalanobis distance (MD) is a generalization of the Euclidean distance that accounts for correlations among the X properties. It is calculated as the distance to the center of the training data. The larger the MD, the less trustworthy the prediction.
Mahalanobis Distance p-value: The p-value gives the fraction of training data with an MD greater than or equal to the one for the given sample, assuming normally distributed data. The smaller the p-value, the less trustworthy the prediciton. For highly non-normal X properties (e.g., fingerprints), the MD p-value is wildly inaccurate.

Structural Similar Compounds

Name	GLIPIZIDE	DANTROLENE.NA	CHLORSULFURON
Structure	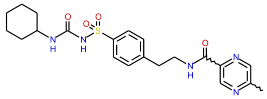	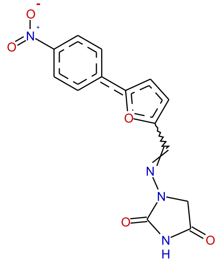	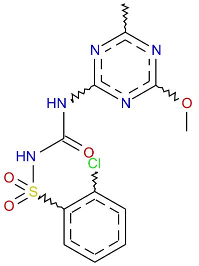
Actual Endpoint (-log C)	3.94991	4.19625	4.15566
Predicted Endpoint (-log C)	3.95594	4.62637	3.79771
Distance	0.583	0.600	0.614
Reference	NDA-17583	NDA-17443	EPA COVER SHEET 0027;880301;(1)

Model Applicability

Unknown features are fingerprint features in the query molecule, but not found or appearing too infrequently in the training set.

1. OPS PC22 out of range. Value: -5.6255. Training min, max, SD, explained variance: -4.3287, 5.3383, 1.588, 0.0110.
2. Unknown ECFP_6 feature: -154530762: [*]N[*]
3. Unknown ECFP_6 feature: 912478223: [*]S[*]
4. Unknown ECFP_6 feature: 1997021792: [*]:[cH]:[cH]:[cH]:[*]
5. Unknown ECFP_6 feature: 1335833675: [*]N[c](:[cH]:[*]):[c]([*]):[*]
6. Unknown ECFP_6 feature: -1699286547: [*]C(=[*])N[c](:[*]):[*]
7. Unknown ECFP_6 feature: 1298725959: [*]NC(=O)C(=[*])[*]
8. Unknown ECFP_6 feature: 1790105651: [*]C(=C1C(=[*])[*][*]:[c]1:[*])[*]
9. Unknown ECFP_6 feature: -631778390: [*]C(=C1S[*][*]C1=[*])[*]
10. Unknown ECFP_6 feature: 2122741631: [*]=C1[*][*]C(=[*])S1
11. Unknown ECFP_6 feature: 190445529: [*]N1[*][*]SC1=O
12. Unknown ECFP_6 feature: -661097313: [*]CN1C(=[*])[*][*]C1=[*]
13. Unknown ECFP_6 feature: 1945129186: [*]N1[*][*]C(=[*])C1=O
14. Unknown ECFP_6 feature: -37698365: [*]N([*])CC(=[*])[*]
15. Unknown ECFP_6 feature: 1731843802: [*]CC(=O)N[*]
16. Unknown ECFP_6 feature: 497523368: [*]CNC(=[*])[*]
17. Unknown ECFP_6 feature: -1791034651: [*]CCN[*]
18. Unknown ECFP_6 feature: -1795525632: [*]CC[c](:[*]):[*]

Feature Contribution

Top features for positive contribution

Fingerprint	Bit/Smiles	Feature Structure	Score
FCFP_6	-1143715940	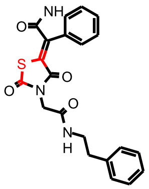 [*]=C1[*][*]C(=[*])S1	0.13
ECFP_6	1559650422	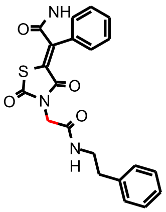 [*]C[*]	0.129
FCFP_6	3	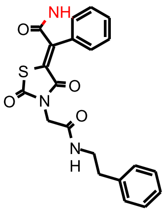 [*]N[*]	0.0924

Top Features for negative contribution

Fingerprint	Bit/Smiles	Feature Structure	Score
FCFP_6	991735244	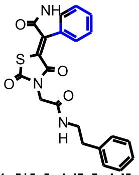 [*][c]1:[*]:[cH]:[cH]:[cH]:[cH]:1	-0.134

ECFP_6	1564392544	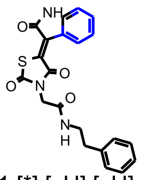 [*][c]1:[*]:[cH]:[cH]:[cH]:[cH]:1	-0.133
ECFP_6	2106656448	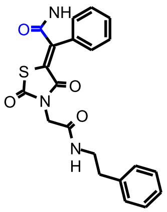 [*]C(=O)[*]	-0.11

Sorafenib

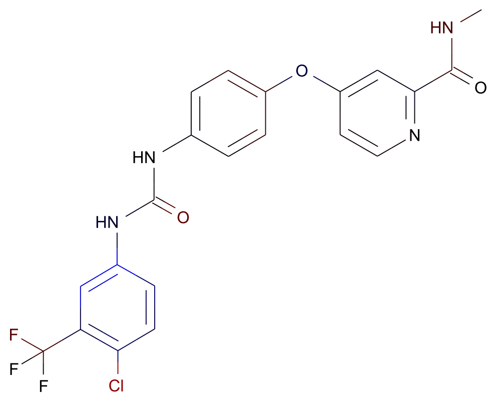

$C_{21}H_{16}ClF_3N_4O_3$
Molecular Weight: 464.82494
ALogP: 4.175
Rotatable Bonds: 6
Acceptors: 4
Donors: 3

Model Prediction

Prediction: 0.00483
Unit: g/kg_body_weight
Mahalanobis Distance: 30
Mahalanobis Distance p-value: 1.21e-024

Mahalanobis Distance: The Mahalanobis distance (MD) is a generalization of the Euclidean distance that accounts for correlations among the X properties. It is calculated as the distance to the center of the training data. The larger the MD, the less trustworthy the prediction.
Mahalanobis Distance p-value: The p-value gives the fraction of training data with an MD greater than or equal to the one for the given sample, assuming normally distributed data. The smaller the p-value, the less trustworthy the prediciton. For highly non-normal X properties (e.g., fingerprints), the MD p-value is wildly inaccurate.

Structural Similar Compounds

Name	GLYBURIDE	D & C RED 9	SODIUM ACIFLUORFEN
Structure	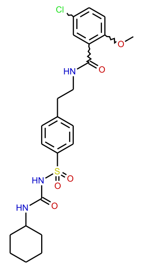	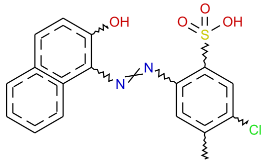	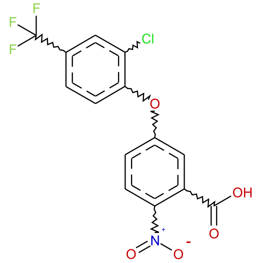
Actual Endpoint (-log C)	4.21661	3.87715	4.16036
Predicted Endpoint (-log C)	4.21035	3.6546	4.65915
Distance	0.636	0.722	0.736
Reference	UPJ-26452	NTP REPORT # 225	EPA COVER SHEET 0192;891101;(1)

Model Applicability

Unknown features are fingerprint features in the query molecule, but not found or appearing too infrequently in the training set.

1. All properties and OPS components are within expected ranges.
2. Unknown ECFP_6 feature: -1046436026: [*]F
3. Unknown ECFP_6 feature: 99947387: [*]:[c](:[*])Cl
4. Unknown ECFP_6 feature: 226796801: [*]C([*])([*])F
5. Unknown ECFP_6 feature: 1305253718: [*]:[c](:[*])O[c](:[*]):[*]
6. Unknown ECFP_6 feature: -677309799: [*][c](:[*]):n:[cH]:[*]
7. Unknown ECFP_6 feature: 1338334141: [*]C(=[*])NC
8. Unknown ECFP_6 feature: -177077903: [*]N[c](:[cH]:[*]):[cH]:[*]
9. Unknown ECFP_6 feature: 1336678434: [*][c](:[*]):[c](:[cH]:[*])C([*])([*])[*]
10. Unknown ECFP_6 feature: -649580166: [*]NC(=O)N[*]
11. Unknown ECFP_6 feature: -1952889961: [*]:[c](:[*])C(F)(F)F
12. Unknown ECFP_6 feature: 1413420509: [*]C(=[*])[c](:[cH]:[*]):n:[*]
13. Unknown ECFP_6 feature: 1996163143: [*]:[cH]:[cH]:n:[*]
14. Unknown ECFP_6 feature: 1430169877: [*]NC(=O)[c](:[*]):[*]
15. Unknown ECFP_6 feature: 864287155: [*]NC

Feature Contribution

Top features for positive contribution

Fingerprint	Bit/Smiles	Feature Structure	Score
ECFP_6	-176455838	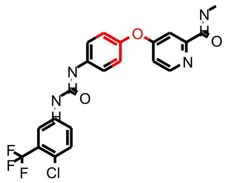 [*]O[c](:[cH]:[*]):[cH]:[*]	0.106
FCFP_6	32	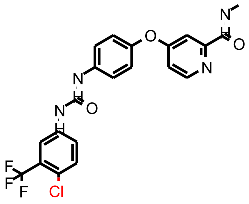 [*]Cl	0.101
FCFP_6	3	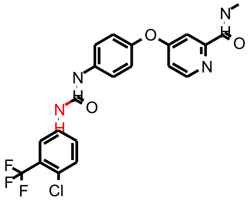 [*]N[*]	0.0924
**Top Features for negative contribution**			
**Fingerprint**	**Bit/Smiles**	**Feature Structure**	**Score**
FCFP_6	1	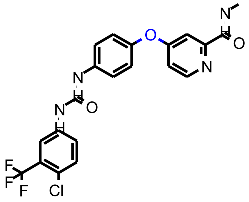 [*]=O	-0.102

ECFP_6	-1236483485	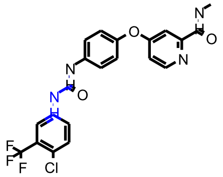 [*]C(=[*])N[c](:[*]):[*]	-0.0747
FCFP_6	203677720	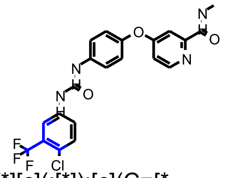 [*][c](:[*]):[c](C=[*]):[cH]:[*]	-0.0713

Sunitinib

TOPKAT_Chronic_LOAEL

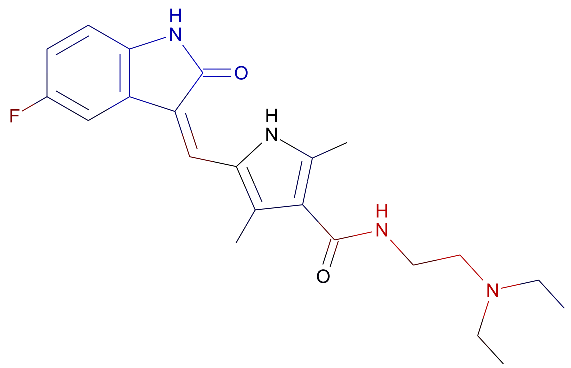

$C_{22}H_{27}FN_4O_2$
Molecular Weight: 398.47378
ALogP: 2.997
Rotatable Bonds: 7
Acceptors: 3
Donors: 3

Model Prediction

Prediction: 0.0397
Unit: g/kg_body_weight
Mahalanobis Distance: 38.1
Mahalanobis Distance p-value: 6.11e-039

Mahalanobis Distance: The Mahalanobis distance (MD) is a generalization of the Euclidean distance that accounts for correlations among the X properties. It is calculated as the distance to the center of the training data. The larger the MD, the less trustworthy the prediction.
Mahalanobis Distance p-value: The p-value gives the fraction of training data with an MD greater than or equal to the one for the given sample, assuming normally distributed data. The smaller the p-value, the less trustworthy the prediciton. For highly non-normal X properties (e.g., fingerprints), the MD p-value is wildly inaccurate.

Structural Similar Compounds

Name	METOCLOPRAMIDE	PROPAFENONE.HCL	ISOXABEN
Structure	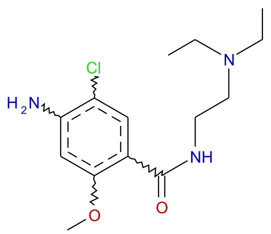	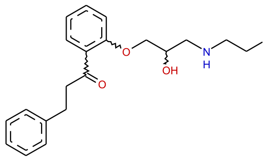	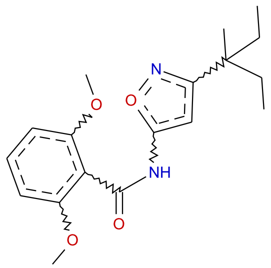
Actual Endpoint (-log C)	4.47683	3.10196	3.81665
Predicted Endpoint (-log C)	3.8785	2.93237	4.42315
Distance	0.630	0.693	0.693
Reference	NDA-17854	NDA-19151	EPA COVER SHEET 0339;881201;(1)

Model Applicability

Unknown features are fingerprint features in the query molecule, but not found or appearing too infrequently in the training set.

1. All properties and OPS components are within expected ranges.
2. Unknown ECFP_6 feature: -1046436026: [*]F
3. Unknown ECFP_6 feature: -152683720: [*]:[nH]:[*]
4. Unknown ECFP_6 feature: -154530762: [*]N[*]
5. Unknown ECFP_6 feature: 220735655: [*]:[c](:[*])F
6. Unknown ECFP_6 feature: -1866225067: [*]CN(C[*])C[*]
7. Unknown ECFP_6 feature: 558201926: [*][c]1:[*]:[*]:[c]([*]):[nH]:1
8. Unknown ECFP_6 feature: 497523368: [*]CNC(=[*])[*]
9. Unknown ECFP_6 feature: -1699286547: [*]C(=[*])N[c](:[*]):[*]
10. Unknown ECFP_6 feature: -1789942192: [*]CCN([*])[*]
11. Unknown ECFP_6 feature: -1658273810: [*]C(=[*])[c]1:[c]([*]):[*]:[*]:[c]:1[*]
12. Unknown ECFP_6 feature: 1576255326: [*][c]1:[*]:[*]:[c]([*]):[c]:1C
13. Unknown ECFP_6 feature: 980271847: [*][c]1:[*]:[*]:[nH]:[c]:1C=[*]
14. Unknown ECFP_6 feature: -1791034651: [*]CCN[*]
15. Unknown ECFP_6 feature: 1791989338: [*][c]1:[*]:[*]:[nH]:[c]:1C
16. Unknown ECFP_6 feature: -949131419: [*]N([*])CC
17. Unknown ECFP_6 feature: 1430169877: [*]NC(=O)[c](:[*]):[*]
18. Unknown ECFP_6 feature: 1718013682: [*]\C=C\1/C(=[*])[*][*]:[c]1:[*]
19. Unknown ECFP_6 feature: 1182722866: [*]C(=CC(=[*])[*])[*]

20. Unknown ECFP_6 feature: 1335833675: [*]N[c](:[cH]:[*]):[c]([*]):[*]
21. Unknown ECFP_6 feature: 1298725959: [*]NC(=O)C(=[*])[*]
22. Unknown ECFP_6 feature: -176686665: [*]:[cH]:[c](F):[cH]:[*]

Feature Contribution

Top features for positive contribution			
**Fingerprint**	**Bit/Smiles**	**Feature Structure**	**Score**
ECFP_6	1559650422	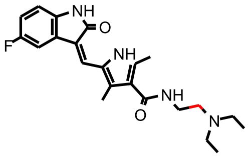 [*]C[*]	0.129
FCFP_6	32	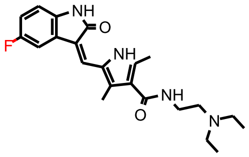 [*]Cl	0.101
FCFP_6	3	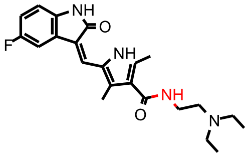 [*]N[*]	0.0924

Top Features for negative contribution			
**Fingerprint**	**Bit/Smiles**	**Feature Structure**	**Score**

ECFP_6	2106656448	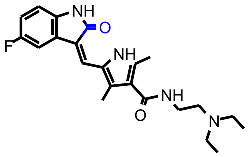<br>[*]C(=O)[*]	-0.11
FCFP_6	1	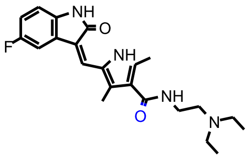<br>[*]=O	-0.102
FCFP_6	136597326	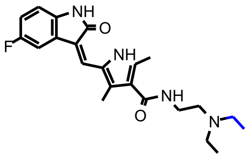<br>[*]CC	-0.0815

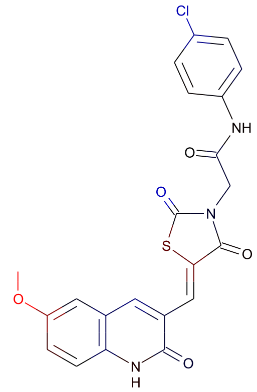

$C_{22}H_{16}ClN_3O_5S$
Molecular Weight: 469.89753
ALogP: 2.815
Rotatable Bonds: 5
Acceptors: 6
Donors: 2

Model Prediction

Prediction: 0.0264
Unit: g/kg_body_weight
Mahalanobis Distance: 11.5
Mahalanobis Distance p-value: 1.06e-007

Mahalanobis Distance: The Mahalanobis distance (MD) is a generalization of the Euclidean distance that accounts for correlations among the X properties. It is calculated as the distance to the center of the training data. The larger the MD, the less trustworthy the prediction.
Mahalanobis Distance p-value: The p-value gives the fraction of training data with an MD greater than or equal to the one for the given sample, assuming normally distributed data. The smaller the p-value, the less trustworthy the prediciton. For highly non-normal X properties (e.g., fingerprints), the MD p-value is wildly inaccurate.

Structural Similar Compounds

Name	FUROSEMIDE	SALICYLAZOSULFAPYRIDINE	COUMAPHOS
Structure	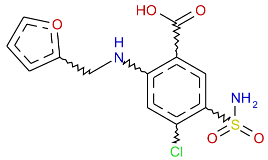	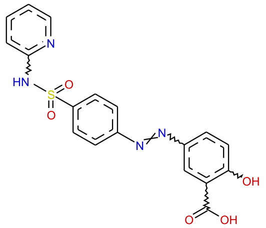	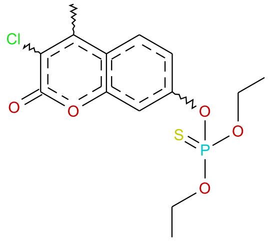
Actual Endpoint (-log C)	4.04236	3.375	5.60537
Predicted Endpoint (-log C)	2.8614	2.80292	4.15004
Distance	0.665	0.740	0.766
Reference	NCI/NTP TR-356	NCI/NTP TR-457	NCI/NTP TR-96

Model Applicability

Unknown features are fingerprint features in the query molecule, but not found or appearing too infrequently in the training set.

1. OPS PC5 out of range. Value: 5.3542. Training min, max, SD, explained variance: -3.3892, 5.0834, 1.644, 0.0611.

Feature Contribution

Top features for positive contribution

Fingerprint	Bit/Smiles	Feature Structure	Score
FCFP_2	136627117	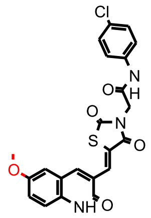 [*]OC	0.173

FCFP_2	-1143715940	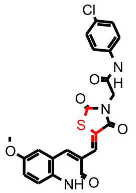 [*]=C1[*][*]C(=[*])S1	0.095
FCFP_2	1036089772	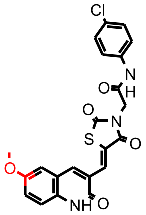 [*]:[c](:[*])OC	0.0749

Top Features for negative contribution

Fingerprint	Bit/Smiles	Feature Structure	Score
FCFP_2	71476542	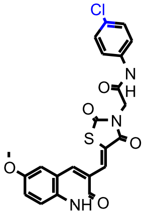 [*]:[c](:[*])Cl	-0.134
FCFP_2	1872154524	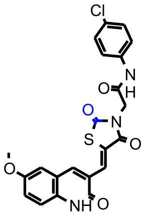 [*]C(=O)[*]	-0.105
FCFP_2	203677720	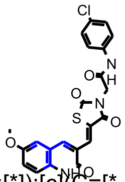 [*][c](:[*]):[c](C=[*]):[cH]:[*]	-0.0829

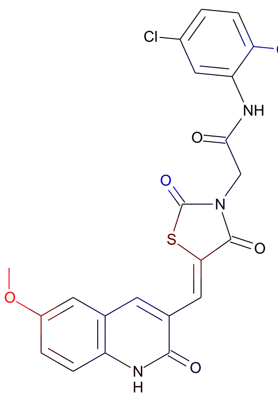

$C_{22}H_{15}Cl_2N_3O_5S$

Molecular Weight: 504.3426

ALogP: 3.479

Rotatable Bonds: 5

Acceptors: 6

Donors: 2

Model Prediction

Prediction: 0.0211

Unit: g/kg_body_weight

Mahalanobis Distance: 11.6

Mahalanobis Distance p-value: 6.59e-008

Mahalanobis Distance: The Mahalanobis distance (MD) is a generalization of the Euclidean distance that accounts for correlations among the X properties. It is calculated as the distance to the center of the training data. The larger the MD, the less trustworthy the prediction.

Mahalanobis Distance p-value: The p-value gives the fraction of training data with an MD greater than or equal to the one for the given sample, assuming normally distributed data. The smaller the p-value, the less trustworthy the prediciton. For highly non-normal X properties (e.g., fingerprints), the MD p-value is wildly inaccurate.

Structural Similar Compounds

Name	FUROSEMIDE	SALICYLAZOSULFAPYRIDINE	COUMAPHOS
Structure	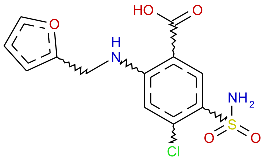	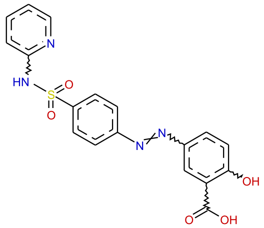	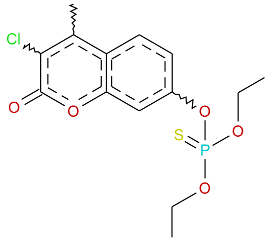
Actual Endpoint (-log C)	4.04236	3.375	5.60537
Predicted Endpoint (-log C)	2.8614	2.80292	4.15004
Distance	0.730	0.759	0.783
Reference	NCI/NTP TR-356	NCI/NTP TR-457	NCI/NTP TR-96

Model Applicability

Unknown features are fingerprint features in the query molecule, but not found or appearing too infrequently in the training set.

1. OPS PC5 out of range. Value: 5.472. Training min, max, SD, explained variance: -3.3892, 5.0834, 1.644, 0.0611.
2. OPS PC9 out of range. Value: 3.4423. Training min, max, SD, explained variance: -2.8548, 3.3954, 1.263, 0.0360.

Feature Contribution

Top features for positive contribution

Fingerprint	Bit/Smiles	Feature Structure	Score
FCFP_2	136627117	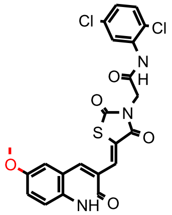 [*]OC	0.173

FCFP_2	-1143715940	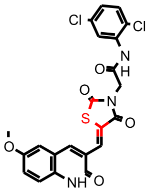 [*]=C1[*][*]C(=[*])S1	0.095
FCFP_2	1036089772	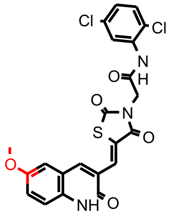 [*]:[c](:[*])OC	0.0749
**Top Features for negative contribution**			
**Fingerprint**	**Bit/Smiles**	**Feature Structure**	**Score**
FCFP_2	71476542	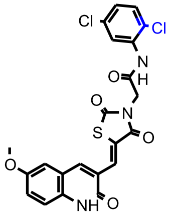 [*]:[c](:[*])Cl	-0.134
FCFP_2	1872154524	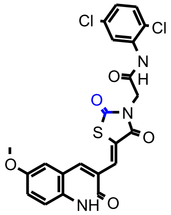 [*]C(=O)[*]	-0.105
FCFP_2	203677720	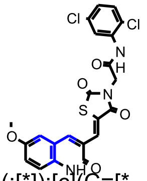 [*][c](:[*]):[c](C=[*]):[cH]:[*]	-0.0829

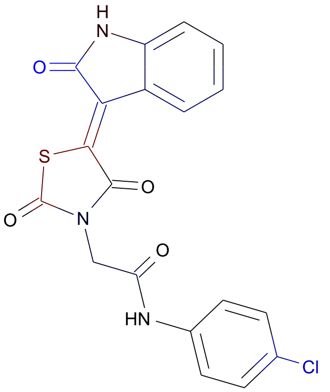

$C_{19}H_{12}ClN_3O_4S$
Molecular Weight: 413.83428
ALogP: 2.363
Rotatable Bonds: 3
Acceptors: 5
Donors: 2

Model Prediction

Prediction: 0.0592
Unit: g/kg_body_weight
Mahalanobis Distance: 10.1
Mahalanobis Distance p-value: 2.57e-005

Mahalanobis Distance: The Mahalanobis distance (MD) is a generalization of the Euclidean distance that accounts for correlations among the X properties. It is calculated as the distance to the center of the training data. The larger the MD, the less trustworthy the prediction.
Mahalanobis Distance p-value: The p-value gives the fraction of training data with an MD greater than or equal to the one for the given sample, assuming normally distributed data. The smaller the p-value, the less trustworthy the prediciton. For highly non-normal X properties (e.g., fingerprints), the MD p-value is wildly inaccurate.

Structural Similar Compounds

Name	FUROSEMIDE	DAPSONE	ACETOHEXAMIDE
Structure	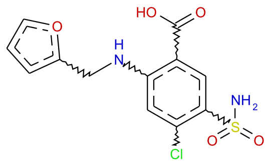	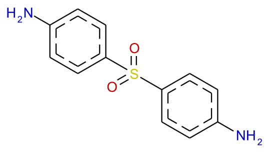	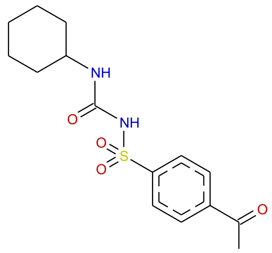
Actual Endpoint (-log C)	4.04236	3.66258	2.55683
Predicted Endpoint (-log C)	2.8614	3.26993	3.62413
Distance	0.586	0.644	0.661
Reference	NCI/NTP TR-356	NCI/NTP TR-20	NCI/NTP TR-050

Model Applicability

Unknown features are fingerprint features in the query molecule, but not found or appearing too infrequently in the training set.

1. OPS PC5 out of range. Value: 5.5028. Training min, max, SD, explained variance: -3.3892, 5.0834, 1.644, 0.0611.

Feature Contribution

Top features for positive contribution			
**Fingerprint**	**Bit/Smiles**	**Feature Structure**	**Score**
FCFP_2	-1143715940	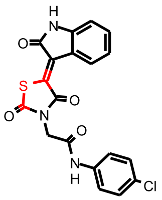 [*]=C1[*][*]C(=[*])S1	0.095

FCFP_2	3	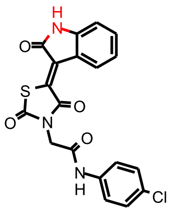 [*]N[*]	0.0737
FCFP_2	565998553	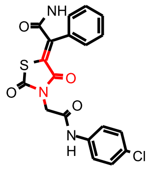 [*]N1[*][*]C(=[*])C1=O	0.00813

Top Features for negative contribution

**Fingerprint**	**Bit/Smiles**	**Feature Structure**	**Score**
FCFP_2	71476542	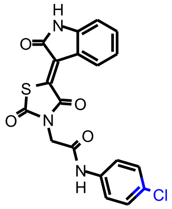 [*]:[c](:[*])Cl	-0.134
FCFP_2	1872154524	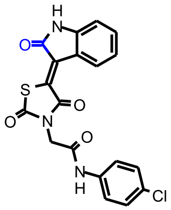 [*]C(=O)[*]	-0.105
FCFP_2	203677720	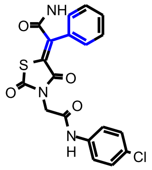 [*][c](:[*]):[c](C=[*]):[cH]:[*]	-0.0829

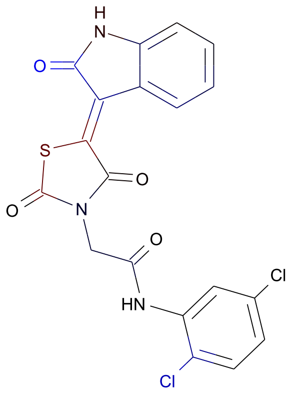

$C_{19}H_{11}Cl_2N_3O_4S$
Molecular Weight: 448.27933
ALogP: 3.028
Rotatable Bonds: 3
Acceptors: 5
Donors: 2

Model Prediction

Prediction: 0.0478
Unit: g/kg_body_weight
Mahalanobis Distance: 10.2
Mahalanobis Distance p-value: 1.82e-005

Mahalanobis Distance: The Mahalanobis distance (MD) is a generalization of the Euclidean distance that accounts for correlations among the X properties. It is calculated as the distance to the center of the training data. The larger the MD, the less trustworthy the prediction.
Mahalanobis Distance p-value: The p-value gives the fraction of training data with an MD greater than or equal to the one for the given sample, assuming normally distributed data. The smaller the p-value, the less trustworthy the prediciton. For highly non-normal X properties (e.g., fingerprints), the MD p-value is wildly inaccurate.

Structural Similar Compounds

Name	FUROSEMIDE	1-AMINO-2,4-DIBROMOANTHRAQUINONE	OXAZEPAM
Structure	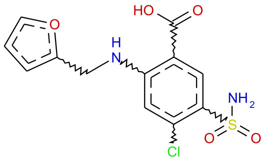	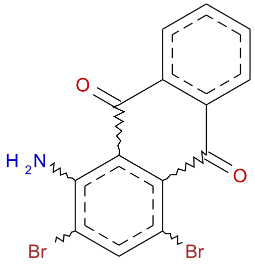	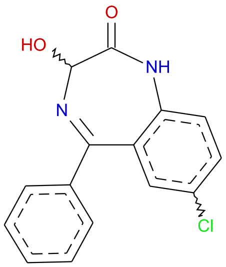
Actual Endpoint (-log C)	4.04236	2.82966	3.05262
Predicted Endpoint (-log C)	2.8614	3.92444	3.13073
Distance	0.639	0.676	0.696
Reference	NCI/NTP TR-356	NCI/NTP TR-383	NCI/NTP TR-468

Model Applicability

Unknown features are fingerprint features in the query molecule, but not found or appearing too infrequently in the training set.

1. OPS PC5 out of range. Value: 5.6206. Training min, max, SD, explained variance: -3.3892, 5.0834, 1.644, 0.0611.

Feature Contribution

Top features for positive contribution			
**Fingerprint**	**Bit/Smiles**	**Feature Structure**	**Score**
FCFP_2	-1143715940	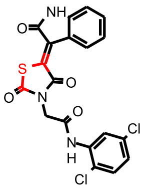 [*]=C1[*][*]C(=[*])S1	0.095

FCFP_2	3	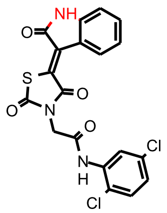 [*]N[*]	0.0737
FCFP_2	565998553	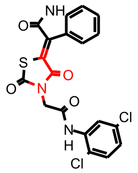 [*]N1[*][*]C(=[*])C1=O	0.00813

Top Features for negative contribution

Fingerprint	Bit/Smiles	Feature Structure	Score
FCFP_2	71476542	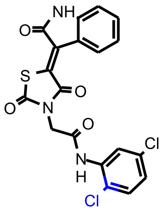 [*]:[c](:[*])Cl	-0.134
FCFP_2	1872154524	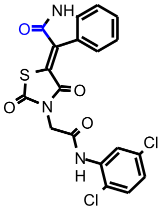 [*]C(=O)[*]	-0.105
FCFP_2	203677720	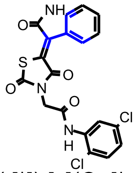 [*][c](:[*]):[c](C=[*]):[cH]:[*]	-0.0829

14c

TOPKAT_Rat_Maximum_Tolerated_Dose_Feed

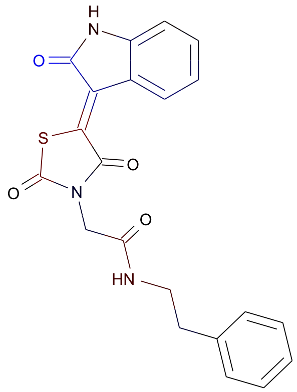

$C_{21}H_{17}N_3O_4S$

Molecular Weight: 407.44237

ALogP: 2.027

Rotatable Bonds: 5

Acceptors: 5

Donors: 2

Model Prediction

Prediction: 0.0423

Unit: g/kg_body_weight

Mahalanobis Distance: 11.7

Mahalanobis Distance p-value: 4.23e-008

Mahalanobis Distance: The Mahalanobis distance (MD) is a generalization of the Euclidean distance that accounts for correlations among the X properties. It is calculated as the distance to the center of the training data. The larger the MD, the less trustworthy the prediction.
Mahalanobis Distance p-value: The p-value gives the fraction of training data with an MD greater than or equal to the one for the given sample, assuming normally distributed data. The smaller the p-value, the less trustworthy the prediciton. For highly non-normal X properties (e.g., fingerprints), the MD p-value is wildly inaccurate.

Structural Similar Compounds

Name	FUROSEMIDE	ACETOHEXAMIDE	DAPSONE
Structure	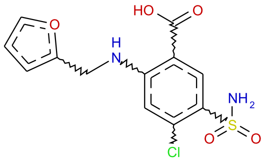	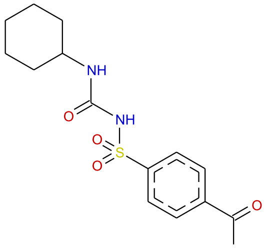	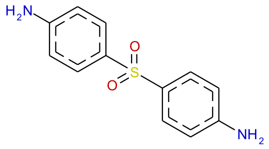
Actual Endpoint (-log C)	4.04236	2.55683	3.66258
Predicted Endpoint (-log C)	2.8614	3.62413	3.26993
Distance	0.613	0.631	0.666
Reference	NCI/NTP TR-356	NCI/NTP TR-050	NCI/NTP TR-20

Model Applicability

Unknown features are fingerprint features in the query molecule, but not found or appearing too infrequently in the training set.

1. All properties and OPS components are within expected ranges.

Feature Contribution

Top features for positive contribution			
**Fingerprint**	**Bit/Smiles**	**Feature Structure**	**Score**
FCFP_2	-885550502	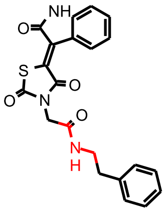 [*]CNC(=[*])[*]	0.115

FCFP_2	-1143715940	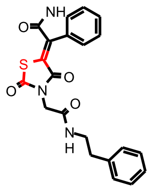 [*]=C1[*][*]C(=[*])S1	0.095
FCFP_2	3	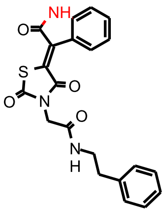 [*]N[*]	0.0737
**Top Features for negative contribution**			
**Fingerprint**	**Bit/Smiles**	**Feature Structure**	**Score**
FCFP_2	1872154524	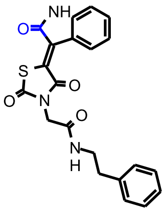 [*]C(=O)[*]	-0.105
FCFP_2	203677720	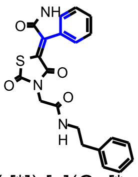 [*][c](:[*]):[c](C=[*])):[cH]:[*]	-0.0829
FCFP_2	1	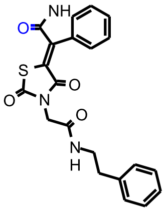 [*]=O	-0.0796

Sorafenib

TOPKAT_Rat_Maximum_Tolerated_Dose_Feed

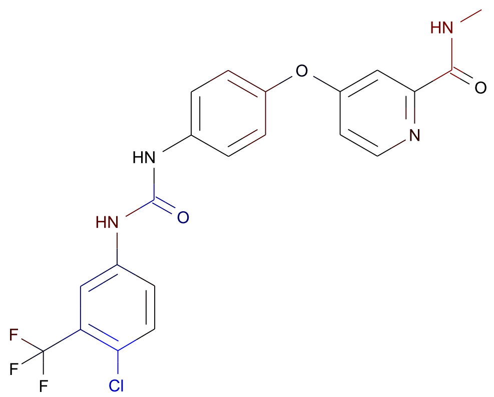

$C_{21}H_{16}ClF_3N_4O_3$

Molecular Weight: 464.82494

ALogP: 4.175

Rotatable Bonds: 6

Acceptors: 4

Donors: 3

Model Prediction

Prediction: 0.0885

Unit: g/kg_body_weight

Mahalanobis Distance: 12.4

Mahalanobis Distance p-value: 1.76e-009

Mahalanobis Distance: The Mahalanobis distance (MD) is a generalization of the Euclidean distance that accounts for correlations among the X properties. It is calculated as the distance to the center of the training data. The larger the MD, the less trustworthy the prediction.
Mahalanobis Distance p-value: The p-value gives the fraction of training data with an MD greater than or equal to the one for the given sample, assuming normally distributed data. The smaller the p-value, the less trustworthy the prediciton. For highly non-normal X properties (e.g., fingerprints), the MD p-value is wildly inaccurate.

Structural Similar Compounds

Name	FUROSEMIDE	PHENOLPHTHALEIN	DISPERSE YELLOW 3
Structure	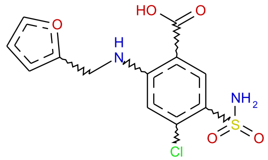	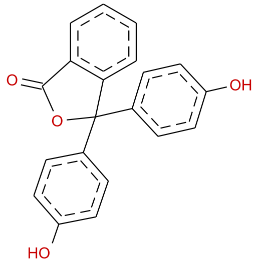	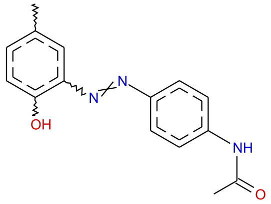
Actual Endpoint (-log C)	4.04236	2.20184	2.77703
Predicted Endpoint (-log C)	2.8614	2.8857	2.80195
Distance	0.741	0.780	0.799
Reference	NCI/NTP TR-356	NCI/NTP TR-465	NCI/NTP TR-222

Model Applicability

Unknown features are fingerprint features in the query molecule, but not found or appearing too infrequently in the training set.

1. All properties and OPS components are within expected ranges.

Feature Contribution

Top features for positive contribution

Fingerprint	Bit/Smiles	Feature Structure	Score
FCFP_2	-885550502	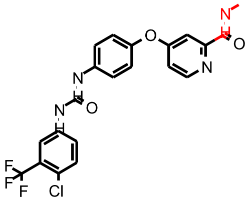 [*]CNC(=[*])[*]	0.115

FCFP_2	3	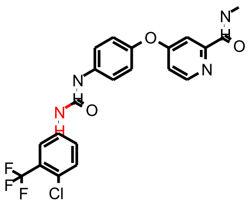 [*]N[*]	0.0737
FCFP_2	332760439	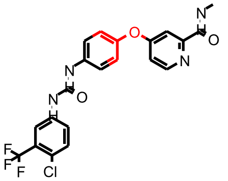 [*]O[c](:[cH]:[*]):[c H]:[*]	0.0611

Top Features for negative contribution

**Fingerprint**	**Bit/Smiles**	**Feature Structure**	**Score**
FCFP_2	71476542	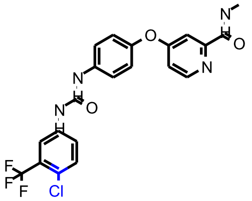 [*]:[c](:[*])Cl	-0.134
FCFP_2	1872154524	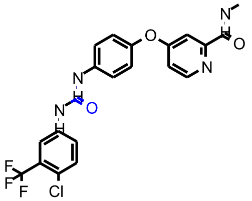 [*]C(=O)[*]	-0.105
FCFP_2	203677720	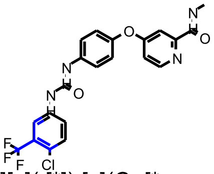 [*][c](:[*]):[c](C=[*]):[cH]:[*]	-0.0829

Sunitinib

TOPKAT_Rat_Maximum_Tolerated_Dose_Feed

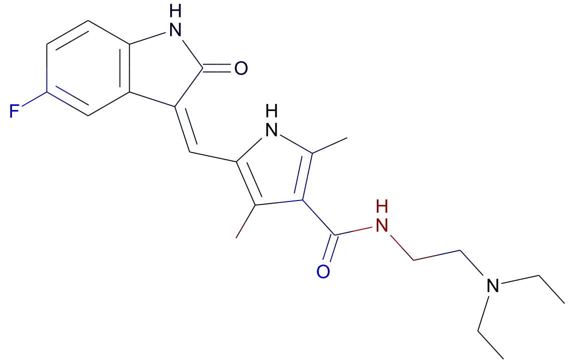

$C_{22}H_{27}FN_4O_2$

Molecular Weight: 398.47378

ALogP: 2.997

Rotatable Bonds: 7

Acceptors: 3

Donors: 3

Model Prediction

Prediction: 0.178

Unit: g/kg_body_weight

Mahalanobis Distance: 9.78

Mahalanobis Distance p-value: 9.94e-005

Mahalanobis Distance: The Mahalanobis distance (MD) is a generalization of the Euclidean distance that accounts for correlations among the X properties. It is calculated as the distance to the center of the training data. The larger the MD, the less trustworthy the prediction.
Mahalanobis Distance p-value: The p-value gives the fraction of training data with an MD greater than or equal to the one for the given sample, assuming normally distributed data. The smaller the p-value, the less trustworthy the prediciton. For highly non-normal X properties (e.g., fingerprints), the MD p-value is wildly inaccurate.

Structural Similar Compounds

Name	CHLORPROPAMIDE	DISPERSE YELLOW 3	TOLBUTAMIDE
Structure	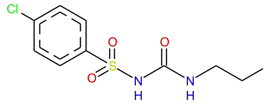	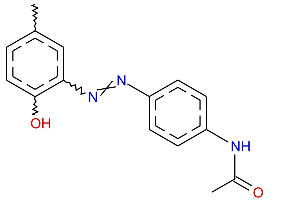	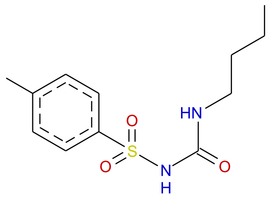
Actual Endpoint (-log C)	3.0107	2.77703	2.3985
Predicted Endpoint (-log C)	3.18321	2.80195	3.32272
Distance	0.657	0.661	0.672
Reference	NCI/NTP TR-045	NCI/NTP TR-222	NCI/NTP TR-031

Model Applicability

Unknown features are fingerprint features in the query molecule, but not found or appearing too infrequently in the training set.

1. All properties and OPS components are within expected ranges.

Feature Contribution

Top features for positive contribution			
**Fingerprint**	**Bit/Smiles**	**Feature Structure**	**Score**
FCFP_2	-885550502	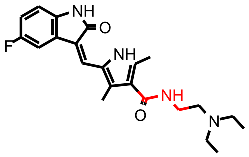 [*]CNC(=[*])[*]	0.115

FCFP_2	3	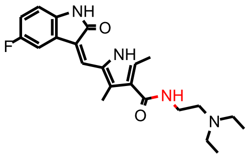 [*]N[*]	0.0737
FCFP_2	136120670	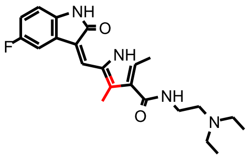 [*]:[c](:[*])C	0.064

Top Features for negative contribution

**Fingerprint**	**Bit/Smiles**	**Feature Structure**	**Score**
FCFP_2	71476542	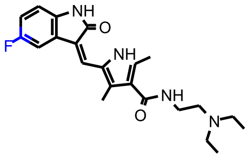 [*]:[c](:[*])Cl	-0.134
FCFP_2	1872154524	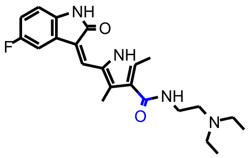 [*]C(=O)[*]	-0.105
FCFP_2	203677720	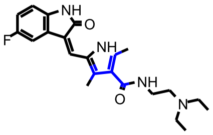 [*][c](:[*]):[c](C=[*]):[cH]:[*]	-0.0829

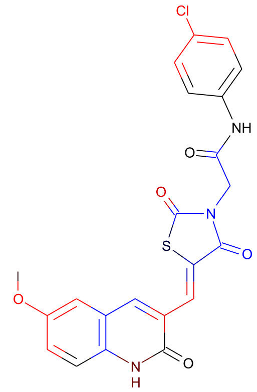

$C_{22}H_{16}ClN_3O_5S$
Molecular Weight: 469.89753
ALogP: 2.815
Rotatable Bonds: 5
Acceptors: 6
Donors: 2

Model Prediction

Prediction: 0.000779
Unit: g/kg_body_weight
Mahalanobis Distance: 11.7
Mahalanobis Distance p-value: 2.5e-008

Mahalanobis Distance: The Mahalanobis distance (MD) is a generalization of the Euclidean distance that accounts for correlations among the X properties. It is calculated as the distance to the center of the training data. The larger the MD, the less trustworthy the prediction.
Mahalanobis Distance p-value: The p-value gives the fraction of training data with an MD greater than or equal to the one for the given sample, assuming normally distributed data. The smaller the p-value, the less trustworthy the prediciton. For highly non-normal X properties (e.g., fingerprints), the MD p-value is wildly inaccurate.

Structural Similar Compounds

Name	OCHRATOXIN	PENICILLIN VK	SULFISOOXAZOLE
Structure	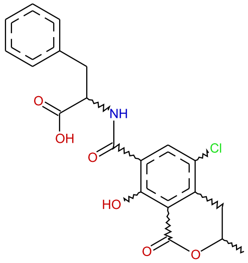	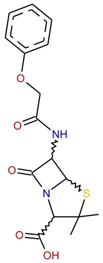	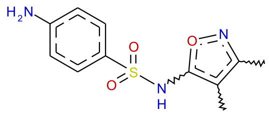
Actual Endpoint (-log C)	6.28396	2.54455	2.82494
Predicted Endpoint (-log C)	5.12358	3.9702	3.0705
Distance	0.628	0.725	0.908
Reference	NCI/NTP TR-358	NCI/NTP TR-336	NCI/NTP TR-138

Model Applicability

Unknown features are fingerprint features in the query molecule, but not found or appearing too infrequently in the training set.

1. Molecular_Weight out of range. Value: 469.9. Training min, max, mean, SD: 68.074, 434.63, 171.13, 85.06.
2. OPS PC5 out of range. Value: -4.1326. Training min, max, SD, explained variance: -3.4, 4.1587, 1.489, 0.0686.
3. OPS PC10 out of range. Value: 2.8975. Training min, max, SD, explained variance: -3.9696, 2.3971, 0.982, 0.0298.
4. Unknown FCFP_2 feature: 436915834: [*]\C=C\1/S[*][*]C1=[*]
5. Unknown FCFP_2 feature: -1986158408: [*]N1[*][*]SC1=O

Feature Contribution

Top features for positive contribution

Fingerprint	Bit/Smiles	Feature Structure	Score

FCFP_2	332760439	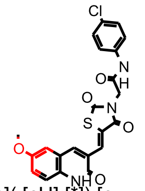 [*]O[c](:[cH]:[*]):[cH]:[*]	0.672
FCFP_2	32	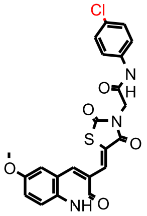 [*]Cl	0.526
FCFP_2	1	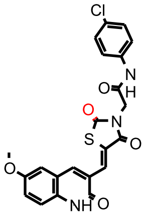 [*]=O	0.511

Top Features for negative contribution

Fingerprint	Bit/Smiles	Feature Structure	Score
FCFP_2	203677720	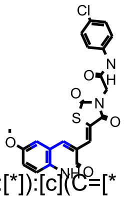 [*][c](:[*]):[c](C=[*]):[cH]:[*]	-0.406
FCFP_2	565998553	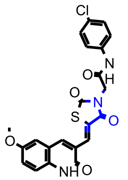 [*]N1[*][*]C(=[*])C1=O	-0.348

FCFP_2	1872154524	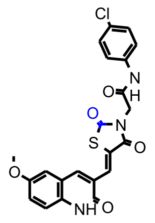 [*]C(=O)[*]	-0.307

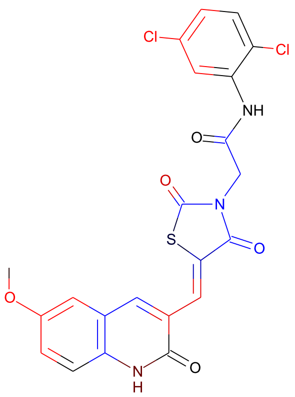

$C_{22}H_{15}Cl_2N_3O_5S$

Molecular Weight: 504.3426

ALogP: 3.479

Rotatable Bonds: 5

Acceptors: 6

Donors: 2

Model Prediction

Prediction: 0.00054

Unit: g/kg_body_weight

Mahalanobis Distance: 12

Mahalanobis Distance p-value: 1.01e-008

Mahalanobis Distance: The Mahalanobis distance (MD) is a generalization of the Euclidean distance that accounts for correlations among the X properties. It is calculated as the distance to the center of the training data. The larger the MD, the less trustworthy the prediction.

Mahalanobis Distance p-value: The p-value gives the fraction of training data with an MD greater than or equal to the one for the given sample, assuming normally distributed data. The smaller the p-value, the less trustworthy the prediciton. For highly non-normal X properties (e.g., fingerprints), the MD p-value is wildly inaccurate.

Structural Similar Compounds

Name	OCHRATOXIN	PENICILLIN VK	SULFISOOXAZOLE
Structure	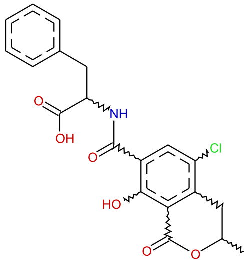	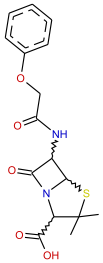	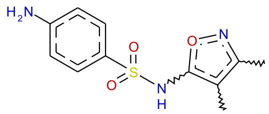
Actual Endpoint (-log C)	6.28396	2.54455	2.82494
Predicted Endpoint (-log C)	5.12358	3.9702	3.0705
Distance	0.667	0.808	0.994
Reference	NCI/NTP TR-358	NCI/NTP TR-336	NCI/NTP TR-138

Model Applicability

Unknown features are fingerprint features in the query molecule, but not found or appearing too infrequently in the training set.

1. Molecular_Weight out of range. Value: 504.34. Training min, max, mean, SD: 68.074, 434.63, 171.13, 85.06.
2. OPS PC5 out of range. Value: -4.1507. Training min, max, SD, explained variance: -3.4, 4.1587, 1.489, 0.0686.
3. OPS PC10 out of range. Value: 2.77. Training min, max, SD, explained variance: -3.9696, 2.3971, 0.982, 0.0298.
4. Unknown FCFP_2 feature: 436915834: [*]\C=C\1/S[*][*]C1=[*]
5. Unknown FCFP_2 feature: -1986158408: [*]N1[*][*]SC1=O

Feature Contribution

Top features for positive contribution

Fingerprint	Bit/Smiles	Feature Structure	Score

FCFP_2	332760439	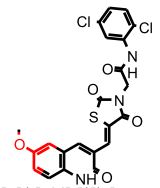 [*]O[c](:[cH]:[*]):[cH]:[*]	0.672
FCFP_2	32	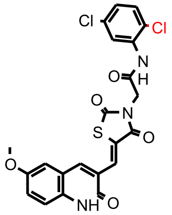 [*]Cl	0.526
FCFP_2	1	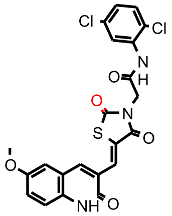 [*]=O	0.511

Top Features for negative contribution

Fingerprint	Bit/Smiles	Feature Structure	Score
FCFP_2	203677720	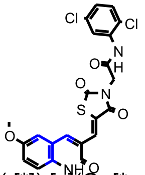 [*][c](:[*]):[c](C=[*]):[cH]:[*]	-0.406
FCFP_2	565998553	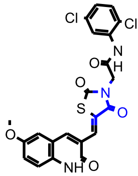 [*]N1[*][*]C(=[*])C1=O	-0.348

FCFP_2	1872154524	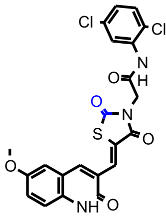<br>[*]C(=O)[*]	-0.307

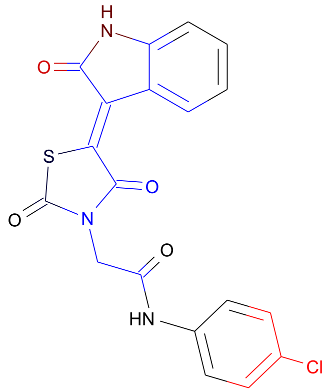

$C_{19}H_{12}ClN_3O_4S$
Molecular Weight: 413.83428
ALogP: 2.363
Rotatable Bonds: 3
Acceptors: 5
Donors: 2

Model Prediction

Prediction: 0.0146
Unit: g/kg_body_weight
Mahalanobis Distance: 8.45
Mahalanobis Distance p-value: 0.000601

Mahalanobis Distance: The Mahalanobis distance (MD) is a generalization of the Euclidean distance that accounts for correlations among the X properties. It is calculated as the distance to the center of the training data. The larger the MD, the less trustworthy the prediction.
Mahalanobis Distance p-value: The p-value gives the fraction of training data with an MD greater than or equal to the one for the given sample, assuming normally distributed data. The smaller the p-value, the less trustworthy the prediciton. For highly non-normal X properties (e.g., fingerprints), the MD p-value is wildly inaccurate.

Structural Similar Compounds

Name	OCHRATOXIN	PENICILLIN VK	SULFISOOXAZOLE
Structure	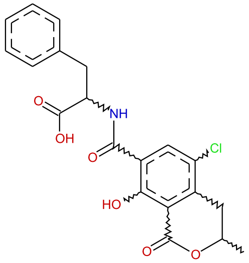	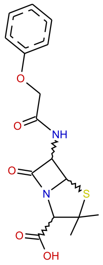	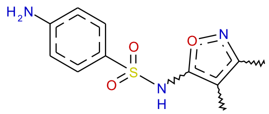
Actual Endpoint (-log C)	6.28396	2.54455	2.82494
Predicted Endpoint (-log C)	5.12358	3.9702	3.0705
Distance	0.624	0.691	0.703
Reference	NCI/NTP TR-358	NCI/NTP TR-336	NCI/NTP TR-138

Model Applicability

Unknown features are fingerprint features in the query molecule, but not found or appearing too infrequently in the training set.

1. OPS PC5 out of range. Value: -3.9678. Training min, max, SD, explained variance: -3.4, 4.1587, 1.489, 0.0686.
2. OPS PC10 out of range. Value: 2.9633. Training min, max, SD, explained variance: -3.9696, 2.3971, 0.982, 0.0298.
3. Unknown FCFP_2 feature: -1678275541: [*]C(=C1C(=[*])[*][*]:[c]1:[*])[*]
4. Unknown FCFP_2 feature: 436915834: [*]\C=C\1/S[*][*]C1=[*]
5. Unknown FCFP_2 feature: -1986158408: [*]N1[*][*]SC1=O

Feature Contribution

Top features for positive contribution			
**Fingerprint**	**Bit/Smiles**	**Feature Structure**	**Score**
FCFP_2	32	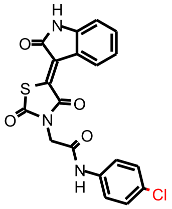 [*]Cl	0.526

FCFP_2	1	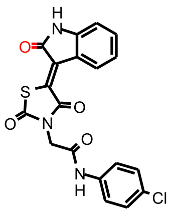 [*]=O	0.511
FCFP_2	367998008	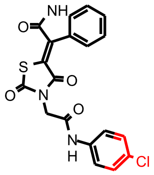 [*]:[cH]:[c](Cl):[cH]:[*]	0.413
**Top Features for negative contribution**			
**Fingerprint**	**Bit/Smiles**	**Feature Structure**	**Score**
FCFP_2	203677720	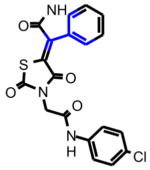 [*][c](:[*]):[c](C=[*]):[cH]:[*]	-0.406
FCFP_2	565998553	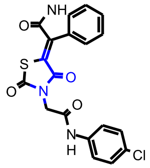 [*]N1[*][*]C(=[*])C1=O	-0.348
FCFP_2	1872154524	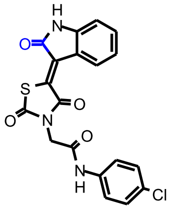 [*]C(=O)[*]	-0.307

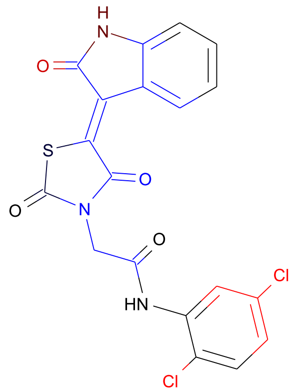

$C_{19}H_{11}Cl_2N_3O_4S$

Molecular Weight: 448.27933

ALogP: 3.028

Rotatable Bonds: 3

Acceptors: 5

Donors: 2

Structural Similar Compounds

Name	OCHRATOXIN	PENICILLIN VK	SULFISOOXAZOLE
Structure	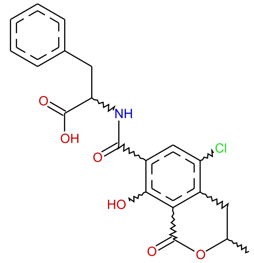	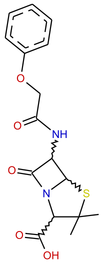	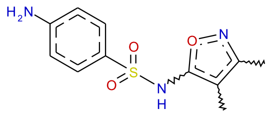
Actual Endpoint (-log C)	6.28396	2.54455	2.82494
Predicted Endpoint (-log C)	5.12358	3.9702	3.0705
Distance	0.622	0.756	0.795
Reference	NCI/NTP TR-358	NCI/NTP TR-336	NCI/NTP TR-138

Model Prediction

Prediction: 0.0102

Unit: g/kg_body_weight

Mahalanobis Distance: 8.86

Mahalanobis Distance p-value: 0.000184

Mahalanobis Distance: The Mahalanobis distance (MD) is a generalization of the Euclidean distance that accounts for correlations among the X properties. It is calculated as the distance to the center of the training data. The larger the MD, the less trustworthy the prediction.
Mahalanobis Distance p-value: The p-value gives the fraction of training data with an MD greater than or equal to the one for the given sample, assuming normally distributed data. The smaller the p-value, the less trustworthy the prediciton. For highly non-normal X properties (e.g., fingerprints), the MD p-value is wildly inaccurate.

Model Applicability

Unknown features are fingerprint features in the query molecule, but not found or appearing too infrequently in the training set.

1. Molecular_Weight out of range. Value: 448.28. Training min, max, mean, SD: 68.074, 434.63, 171.13, 85.06.
2. OPS PC5 out of range. Value: -3.9859. Training min, max, SD, explained variance: -3.4, 4.1587, 1.489, 0.0686.
3. OPS PC10 out of range. Value: 2.8356. Training min, max, SD, explained variance: -3.9696, 2.3971, 0.982, 0.0298.
4. Unknown FCFP_2 feature: -1678275541: [*]C(=C1C(=[*])[*][*]:[c]1:[*])[*]
5. Unknown FCFP_2 feature: 436915834: [*]\C=C\1/S[*][*]C1=[*]
6. Unknown FCFP_2 feature: -1986158408: [*]N1[*][*]SC1=O

Feature Contribution

Top features for positive contribution

Fingerprint	Bit/Smiles	Feature Structure	Score

FCFP_2	32	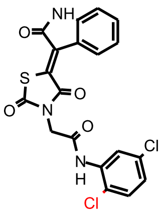 [*]Cl	0.526
FCFP_2	1	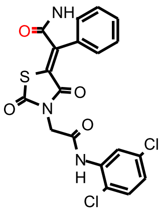 [*]=O	0.511
FCFP_2	367998008	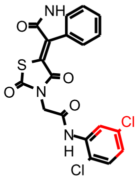 [*]:[cH]:[c](Cl):[cH] :[*]	0.413

Top Features for negative contribution

Fingerprint	Bit/Smiles	Feature Structure	Score
FCFP_2	203677720	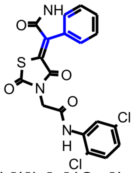 [*][c](:[*]):[c](C=[*]):[cH]:[*]	-0.406
FCFP_2	565998553	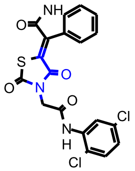 [*]N1[*][*]C(=[*])C1= O	-0.348

FCFP_2	1872154524	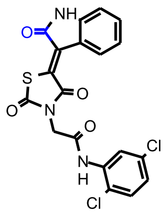<br>[*]C(=O)[*]	-0.307

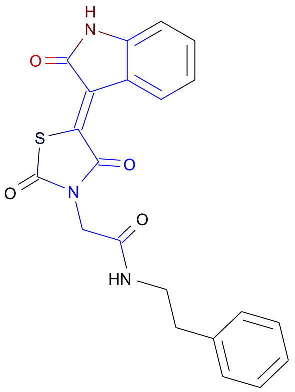

$C_{21}H_{17}N_3O_4S$
Molecular Weight: 407.44237
ALogP: 2.027
Rotatable Bonds: 5
Acceptors: 5
Donors: 2

Model Prediction

Prediction: 0.33
Unit: g/kg_body_weight
Mahalanobis Distance: 7.34
Mahalanobis Distance p-value: 0.0112

Mahalanobis Distance: The Mahalanobis distance (MD) is a generalization of the Euclidean distance that accounts for correlations among the X properties. It is calculated as the distance to the center of the training data. The larger the MD, the less trustworthy the prediction.
Mahalanobis Distance p-value: The p-value gives the fraction of training data with an MD greater than or equal to the one for the given sample, assuming normally distributed data. The smaller the p-value, the less trustworthy the prediciton. For highly non-normal X properties (e.g., fingerprints), the MD p-value is wildly inaccurate.

Structural Similar Compounds

Name	PENICILLIN VK	OCHRATOXIN	SULFISOOXAZOLE
Structure	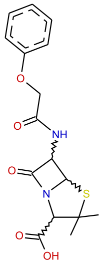	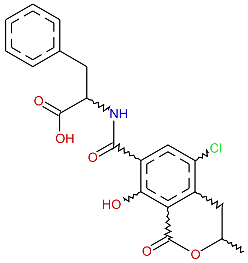	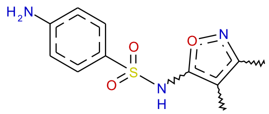
Actual Endpoint (-log C)	2.54455	6.28396	2.82494
Predicted Endpoint (-log C)	3.9702	5.12358	3.0705
Distance	0.626	0.630	0.700
Reference	NCI/NTP TR-336	NCI/NTP TR-358	NCI/NTP TR-138

Model Applicability

Unknown features are fingerprint features in the query molecule, but not found or appearing too infrequently in the training set.

1. All properties and OPS components are within expected ranges.
2. Unknown FCFP_2 feature: -1678275541: [*]C(=C1C(=[*])[*][*]:[c]1:[*])[*]
3. Unknown FCFP_2 feature: 436915834: [*]\C=C\1/S[*][*]C1=[*]
4. Unknown FCFP_2 feature: -1986158408: [*]N1[*][*]SC1=O

Feature Contribution

Top features for positive contribution			
Fingerprint	Bit/Smiles	Feature Structure	Score
FCFP_2	1	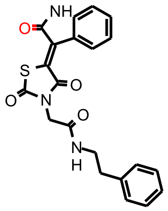 [*]=O	0.511

FCFP_2	3	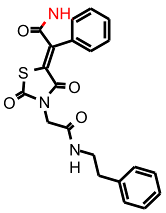 [*]N[*]	0.104
**Top Features for negative contribution**			
**Fingerprint**	**Bit/Smiles**	**Feature Structure**	**Score**
FCFP_2	203677720	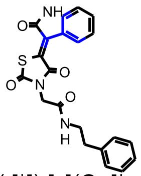 [*][c](:[*]):[c](C=[*]):[cH]:[*]	-0.406
FCFP_2	565998553	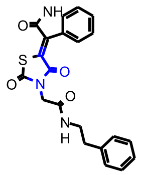 [*]N1[*][*]C(=[*])C1= O	-0.348
FCFP_2	1872154524	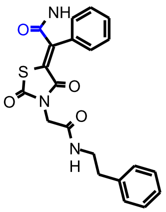 [*]C(=O)[*]	-0.307

Sorafenib

TOPKAT_Rat_Maximum_Tolerated_Dose_Gavage

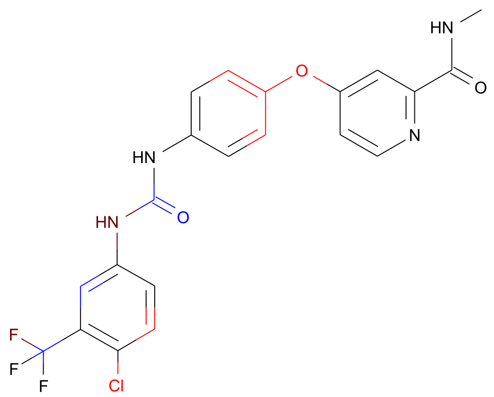

$C_{21}H_{16}ClF_3N_4O_3$

Molecular Weight: 464.82494

ALogP: 4.175

Rotatable Bonds: 6

Acceptors: 4

Donors: 3

Structural Similar Compounds

Name	OCHRATOXIN	SULFISOOXAZOLE	PENICILLIN VK
Structure	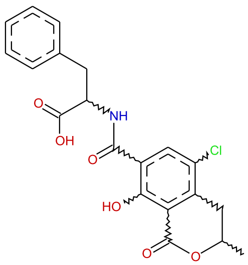	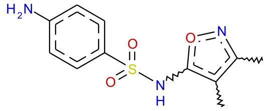	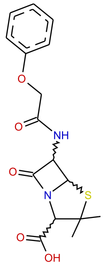
Actual Endpoint (-log C)	6.28396	2.82494	2.54455
Predicted Endpoint (-log C)	5.12358	3.0705	3.9702
Distance	0.758	0.997	1.159
Reference	NCI/NTP TR-358	NCI/NTP TR-138	NCI/NTP TR-336

Model Prediction

Prediction: 0.000918

Unit: g/kg_body_weight

Mahalanobis Distance: 12.2

Mahalanobis Distance p-value: 4.69e-009

Mahalanobis Distance: The Mahalanobis distance (MD) is a generalization of the Euclidean distance that accounts for correlations among the X properties. It is calculated as the distance to the center of the training data. The larger the MD, the less trustworthy the prediction.
Mahalanobis Distance p-value: The p-value gives the fraction of training data with an MD greater than or equal to the one for the given sample, assuming normally distributed data. The smaller the p-value, the less trustworthy the prediciton. For highly non-normal X properties (e.g., fingerprints), the MD p-value is wildly inaccurate.

Model Applicability

Unknown features are fingerprint features in the query molecule, but not found or appearing too infrequently in the training set.

1. Molecular_Weight out of range. Value: 464.82. Training min, max, mean, SD: 68.074, 434.63, 171.13, 85.06.
2. Num_AromaticRings out of range. Value: 3. Training min, max, mean, SD: 0, 2, 0.5625, 0.693.
3. OPS PC5 out of range. Value: -3.5737. Training min, max, SD, explained variance: -3.4, 4.1587, 1.489, 0.0686.
4. OPS PC7 out of range. Value: -3.8342. Training min, max, SD, explained variance: -2.8003, 2.9332, 1.16, 0.0416.
5. Unknown FCFP_2 feature: 1499521844: [*]NC(=O)N[*]
6. Unknown FCFP_2 feature: -1029533685: [*]:[c](:[*])C(F)(F)F
7. Unknown FCFP_2 feature: 136686699: [*]NC

Feature Contribution

Top features for positive contribution			
Fingerprint	Bit/Smiles	Feature Structure	Score

FCFP_2	332760439	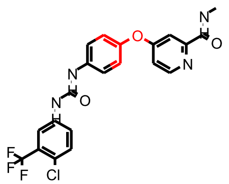 [*]O[c](:[cH]:[*]):[cH]:[*]	0.672
FCFP_2	32	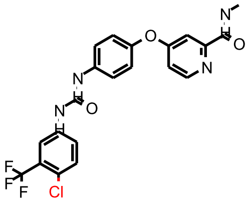 [*]Cl	0.526
FCFP_2	1	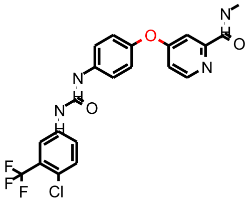 [*]=O	0.511
**Top Features for negative contribution**			
**Fingerprint**	**Bit/Smiles**	**Feature Structure**	**Score**
FCFP_2	203677720	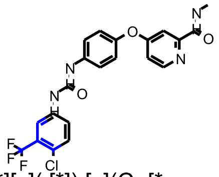 [*][c](:[*]):[c](C=[*]):[cH]:[*]	-0.406
FCFP_2	1872154524	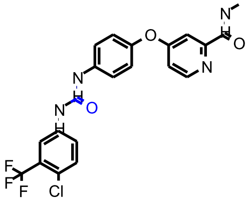 [*]C(=O)[*]	-0.307

FCFP_2	0	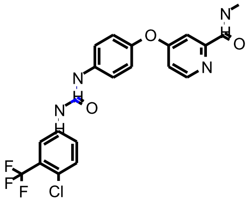<br>[*]C=[*]	-0.29

Sunitinib

TOPKAT_Rat_Maximum_Tolerated_Dose_Gavage

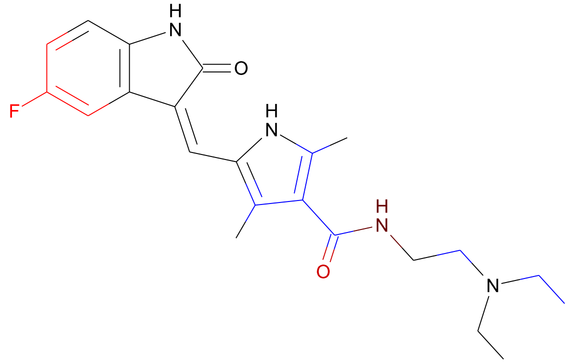

$C_{22}H_{27}FN_4O_2$

Molecular Weight: 398.47378

ALogP: 2.997

Rotatable Bonds: 7

Acceptors: 3

Donors: 3

Model Prediction

Prediction: 0.161

Unit: g/kg_body_weight

Mahalanobis Distance: 9.68

Mahalanobis Distance p-value: 1.53e-005

Mahalanobis Distance: The Mahalanobis distance (MD) is a generalization of the Euclidean distance that accounts for correlations among the X properties. It is calculated as the distance to the center of the training data. The larger the MD, the less trustworthy the prediction.
Mahalanobis Distance p-value: The p-value gives the fraction of training data with an MD greater than or equal to the one for the given sample, assuming normally distributed data. The smaller the p-value, the less trustworthy the prediciton. For highly non-normal X properties (e.g., fingerprints), the MD p-value is wildly inaccurate.

Structural Similar Compounds

Name	OCHRATOXIN	SULFISOOXAZOLE	PROBENECID
Structure	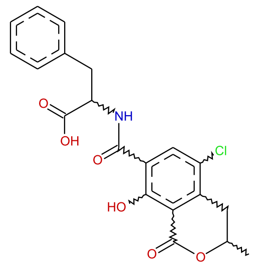	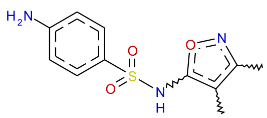	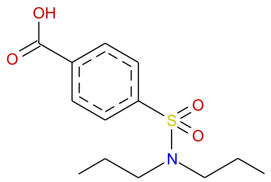
Actual Endpoint (-log C)	6.28396	2.82494	2.85333
Predicted Endpoint (-log C)	5.12358	3.0705	2.4258
Distance	0.784	0.908	0.912
Reference	NCI/NTP TR-358	NCI/NTP TR-138	NCI/NTP TR-395

Model Applicability

Unknown features are fingerprint features in the query molecule, but not found or appearing too infrequently in the training set.

1. OPS PC7 out of range. Value: -3.6239. Training min, max, SD, explained variance: -2.8003, 2.9332, 1.16, 0.0416.
2. Unknown FCFP_2 feature: 19: [*]:[nH]:[*]
3. Unknown FCFP_2 feature: 2005402822: [*][c]1:[*]:[*]:[c]([*]):[nH]:1
4. Unknown FCFP_2 feature: 203707511: [*][c]1:[*]:[*]:[nH]:[c]:1C=[*]
5. Unknown FCFP_2 feature: -1678275541: [*]C(=C1C(=[*])[*][*]:[c]1:[*])[*]

Feature Contribution

Top features for positive contribution

Fingerprint	Bit/Smiles	Feature Structure	Score
FCFP_2	32	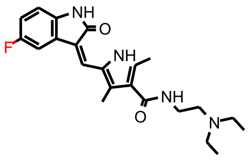 [*]Cl	0.526

FCFP_2	1	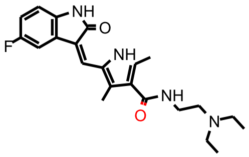 [*]=O	0.511
FCFP_2	367998008	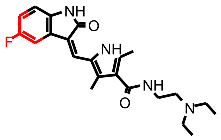 [*]:[cH]:[c](Cl):[cH]:[*]	0.413

Top Features for negative contribution

Fingerprint	Bit/Smiles	Feature Structure	Score
FCFP_2	136597326	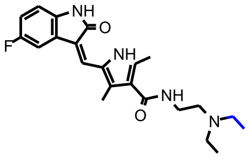 [*]CC	-0.489
FCFP_2	203677720	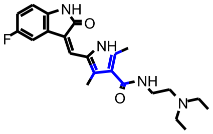 [*][c](:[*]):[c](C=[*]):[cH]:[*]	-0.406
FCFP_2	1872154524	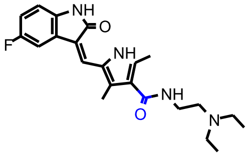 [*]C(=O)[*]	-0.307

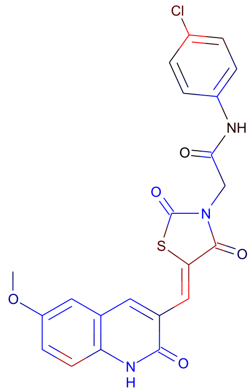

$C_{22}H_{16}ClN_3O_5S$
Molecular Weight: 469.89753
ALogP: 2.815
Rotatable Bonds: 5
Acceptors: 6
Donors: 2

Model Prediction

Prediction: 0.936
Unit: g/kg_body_weight
Mahalanobis Distance: 24.1
Mahalanobis Distance p-value: 4.69e-026

Mahalanobis Distance: The Mahalanobis distance (MD) is a generalization of the Euclidean distance that accounts for correlations among the X properties. It is calculated as the distance to the center of the training data. The larger the MD, the less trustworthy the prediction.
Mahalanobis Distance p-value: The p-value gives the fraction of training data with an MD greater than or equal to the one for the given sample, assuming normally distributed data. The smaller the p-value, the less trustworthy the prediciton. For highly non-normal X properties (e.g., fingerprints), the MD p-value is wildly inaccurate.

Structural Similar Compounds

Name	PIRETANIDE	ETHYL-bis-COUMACETATE	OCHRATOXIN A
Structure	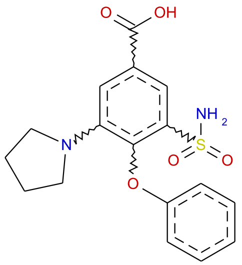	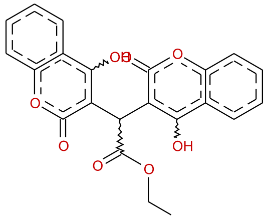	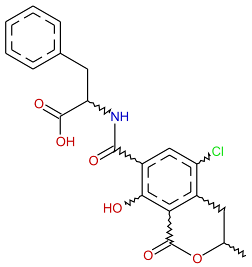
Actual Endpoint (-log C)	1.811	2.687	4.305
Predicted Endpoint (-log C)	1.83976	2.7054	3.03558
Distance	0.600	0.612	0.614
Reference	DRFUD4 2;393;77	FEPRA7 10;303;51	FCTXAV 6;479;68

Model Applicability

Unknown features are fingerprint features in the query molecule, but not found or appearing too infrequently in the training set.

1. OPS PC58 out of range. Value: -4.1027. Training min, max, SD, explained variance: -4.0974, 5.4085, 1.034, 0.0039.
2. Unknown ECFP_2 feature: 2131425032: [*]\C=C(\C=[*])/C(=[*])[*]
3. Unknown ECFP_2 feature: 1000552169: [*]\C=C\1/S[*][*]C1=[*]
4. Unknown FCFP_6 feature: 16: [*]:[cH]:[*]
5. Unknown FCFP_6 feature: 71476542: [*]:[c](:[*])Cl
6. Unknown FCFP_6 feature: 1618154665: [*][c](:[*]):[cH]:[cH]:[*]
7. Unknown FCFP_6 feature: 451371068: [*]C(=C[c](:[*]):[*])[*]

Feature Contribution

Top features for positive contribution			
Fingerprint	Bit/Smiles	Feature Structure	Score

ECFP_6	642810091	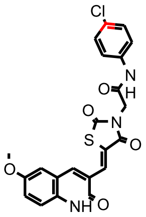 [*][c](:[*]):[*]	0.281
ECFP_6	-1897341097	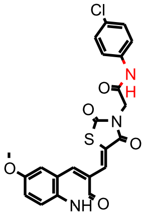 [*]N[*]	0.216
FCFP_6	436915834	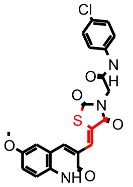 [*]\C=C\1/S[*][*]C1=[*]	0.184

Top Features for negative contribution

**Fingerprint**	**Bit/Smiles**	**Feature Structure**	**Score**
ECFP_6	2106656448	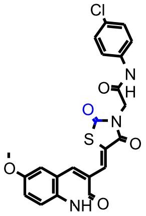 [*]C(=O)[*]	-0.352
ECFP_6	-817402818	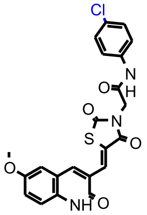 [*]Cl	-0.263

ECFP_6	-176455838	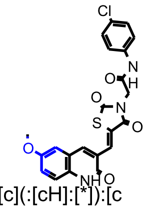 [*]O[c](:[cH]:[*]):[cH]:[*]	-0.257

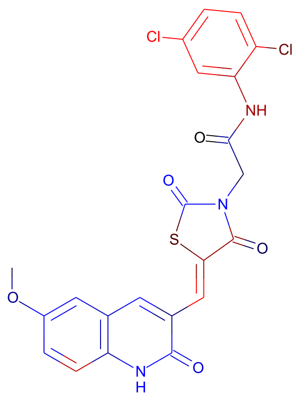

$C_{22}H_{15}Cl_2N_3O_5S$

Molecular Weight: 504.3426

ALogP: 3.479

Rotatable Bonds: 5

Acceptors: 6

Donors: 2

Model Prediction

Prediction: 0.316

Unit: g/kg_body_weight

Mahalanobis Distance: 24.1

Mahalanobis Distance p-value: 3.74e-026

Mahalanobis Distance: The Mahalanobis distance (MD) is a generalization of the Euclidean distance that accounts for correlations among the X properties. It is calculated as the distance to the center of the training data. The larger the MD, the less trustworthy the prediction.
Mahalanobis Distance p-value: The p-value gives the fraction of training data with an MD greater than or equal to the one for the given sample, assuming normally distributed data. The smaller the p-value, the less trustworthy the prediciton. For highly non-normal X properties (e.g., fingerprints), the MD p-value is wildly inaccurate.

Structural Similar Compounds

Name	OCHRATOXIN A	BROMOFENOXIME	ETHYL-bis-COUMACETATE
Structure	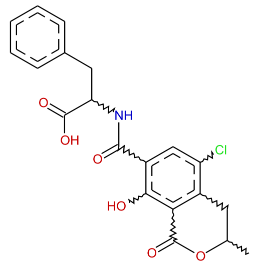	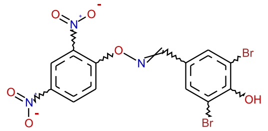	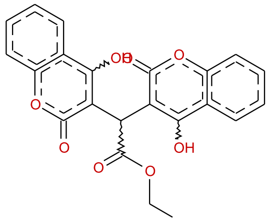
Actual Endpoint (-log C)	4.305	2.622	2.687
Predicted Endpoint (-log C)	3.03558	3.41798	2.7054
Distance	0.647	0.662	0.666
Reference	FCTXAV 6;479;68	85ARAE 2;203;77	FEPRA7 10;303;51

Model Applicability

Unknown features are fingerprint features in the query molecule, but not found or appearing too infrequently in the training set.

1. OPS PC58 out of range. Value: -4.1222. Training min, max, SD, explained variance: -4.0974, 5.4085, 1.034, 0.0039.
2. Unknown ECFP_2 feature: 2131425032: [*]\C=C(\C=[*])/C(=[*])[*]
3. Unknown ECFP_2 feature: 1000552169: [*]\C=C\1/S[*][*]C1=[*]
4. Unknown FCFP_6 feature: 16: [*]:[cH]:[*]
5. Unknown FCFP_6 feature: 71476542: [*]:[c](:[*])Cl
6. Unknown FCFP_6 feature: 1618154665: [*][c](:[*]):[cH]:[cH]:[*]
7. Unknown FCFP_6 feature: 451371068: [*]C(=C[c](:[*]):[*])[*]

Feature Contribution

Top features for positive contribution

Fingerprint	Bit/Smiles	Feature Structure	Score

ECFP_6	642810091	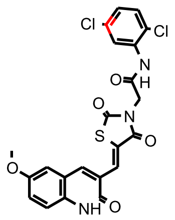 [*][c](:[*]):[*]	0.281
ECFP_6	-1897341097	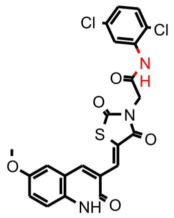 [*]N[*]	0.216
ECFP_6	577592657	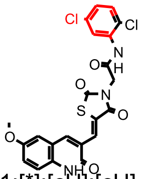 [*][c]1:[*]:[cH]:[cH]:[c](Cl):[cH]:1	0.194
**Top Features for negative contribution**			
**Fingerprint**	**Bit/Smiles**	**Feature Structure**	**Score**
ECFP_6	2106656448	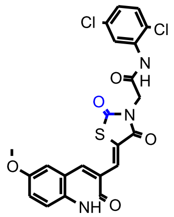 [*]C(=O)[*]	-0.352
ECFP_6	-817402818	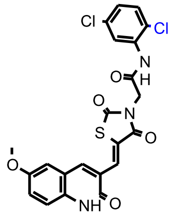 [*]Cl	-0.263

ECFP_6	-176455838	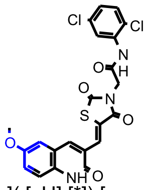<br>[*]O[c](:[cH]:[*]):[cH]:[*]	-0.257

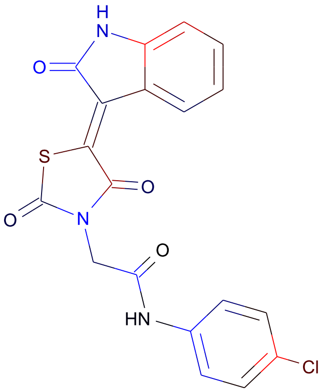

$C_{19}H_{12}ClN_3O_4S$
Molecular Weight: 413.83428
ALogP: 2.363
Rotatable Bonds: 3
Acceptors: 5
Donors: 2

Model Prediction

Prediction: 1.67
Unit: g/kg_body_weight
Mahalanobis Distance: 21.3
Mahalanobis Distance p-value: 2.24e-013

Mahalanobis Distance: The Mahalanobis distance (MD) is a generalization of the Euclidean distance that accounts for correlations among the X properties. It is calculated as the distance to the center of the training data. The larger the MD, the less trustworthy the prediction.
Mahalanobis Distance p-value: The p-value gives the fraction of training data with an MD greater than or equal to the one for the given sample, assuming normally distributed data. The smaller the p-value, the less trustworthy the prediciton. For highly non-normal X properties (e.g., fingerprints), the MD p-value is wildly inaccurate.

Structural Similar Compounds

Name	PIRETANIDE	1H-1;4-BENZODIAZEPINE-1-CARBOXAMIDE; 2;3-DIHYDRO-N-METHYL-7-NITRO-2-OXO-5-PHENYL-	PIROXICAM
Structure	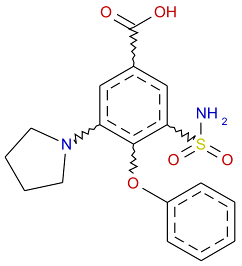	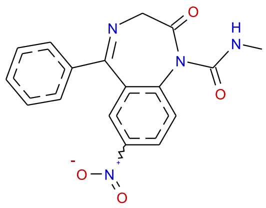	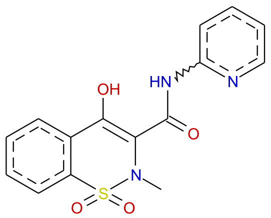
Actual Endpoint (-log C)	1.811	2.171	3.186
Predicted Endpoint (-log C)	1.83976	2.64752	2.63418
Distance	0.568	0.610	0.623
Reference	DRFUD4 2;393;77	TAKHAA 29;153;70	ARZNAD 28;1714;78

Model Applicability

Unknown features are fingerprint features in the query molecule, but not found or appearing too infrequently in the training set.

1. All properties and OPS components are within expected ranges.
2. Unknown FCFP_6 feature: 16: [*]:[cH]:[*]
3. Unknown FCFP_6 feature: 1618154665: [*][c](:[*]):[cH]:[cH]:[*]
4. Unknown FCFP_6 feature: -1678275541: [*]C(=C1C(=[*])[*][*]:[c]1:[*])[*]
5. Unknown FCFP_6 feature: 71476542: [*]:[c](:[*])Cl

Feature Contribution

Top features for positive contribution

Fingerprint	Bit/Smiles	Feature Structure	Score

ECFP_6	642810091	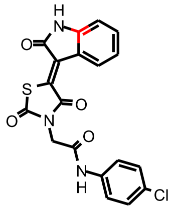 [*][c](:[*]):[*]	0.281
ECFP_6	-1897341097	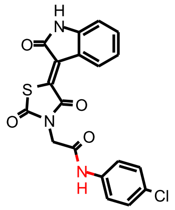 [*]N[*]	0.216
FCFP_6	436915834	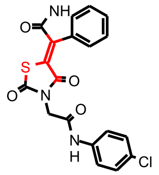 [*]\C=C\1/S[*][*]C1=[*]	0.184

Top Features for negative contribution

**Fingerprint**	**Bit/Smiles**	**Feature Structure**	**Score**
ECFP_6	2106656448	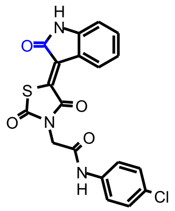 [*]C(=O)[*]	-0.352
ECFP_6	-817402818	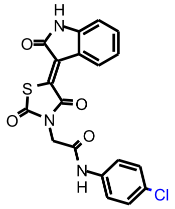 [*]Cl	-0.263

FCFP_6	566058135	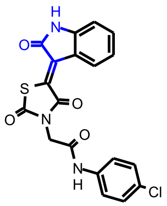<br>[*]NC(=O)C(=[*])[*]	-0.216

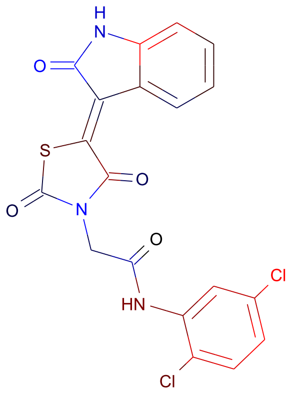

$C_{19}H_{11}Cl_2N_3O_4S$

Molecular Weight: 448.27933

ALogP: 3.028

Rotatable Bonds: 3

Acceptors: 5

Donors: 2

Model Prediction

Prediction: 0.707

Unit: g/kg_body_weight

Mahalanobis Distance: 21.3

Mahalanobis Distance p-value: 2.05e-013

Mahalanobis Distance: The Mahalanobis distance (MD) is a generalization of the Euclidean distance that accounts for correlations among the X properties. It is calculated as the distance to the center of the training data. The larger the MD, the less trustworthy the prediction.
Mahalanobis Distance p-value: The p-value gives the fraction of training data with an MD greater than or equal to the one for the given sample, assuming normally distributed data. The smaller the p-value, the less trustworthy the prediciton. For highly non-normal X properties (e.g., fingerprints), the MD p-value is wildly inaccurate.

Structural Similar Compounds

Name	PIRETANIDE	OCHRATOXIN A	CLONITRALID
Structure	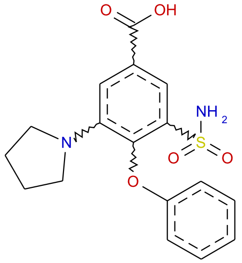	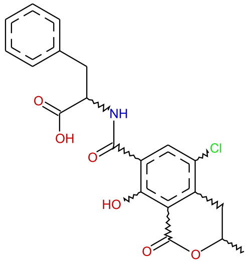	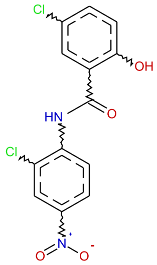
Actual Endpoint (-log C)	1.811	4.305	2.816
Predicted Endpoint (-log C)	1.83976	3.03558	2.74335
Distance	0.618	0.623	0.640
Reference	DRFUD4 2;393;77	FCTXAV 6;479;68	85ARAE 3;103;76/77

Model Applicability

Unknown features are fingerprint features in the query molecule, but not found or appearing too infrequently in the training set.

1. All properties and OPS components are within expected ranges.
2. Unknown FCFP_6 feature: 16: [*]:[cH]:[*]
3. Unknown FCFP_6 feature: 1618154665: [*][c](:[*]):[cH]:[cH]:[*]
4. Unknown FCFP_6 feature: -1678275541: [*]C(=C1C(=[*])[*][*]:[c]1:[*])[*]
5. Unknown FCFP_6 feature: 71476542: [*]:[c](:[*])Cl

Feature Contribution

Top features for positive contribution

Fingerprint	Bit/Smiles	Feature Structure	Score
ECFP_6	642810091	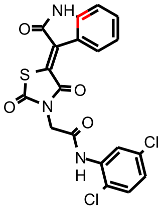 [*][c](:[*]):[*]	0.281

ECFP_6	-1897341097	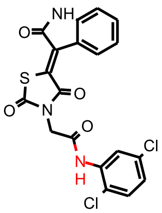 [*]N[*]	0.216
ECFP_6	577592657	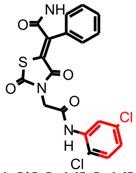 [*][c]1:[*]:[cH]:[cH] :[c](Cl):[cH]:1	0.194
**Top Features for negative contribution**			
**Fingerprint**	**Bit/Smiles**	**Feature Structure**	**Score**
ECFP_6	2106656448	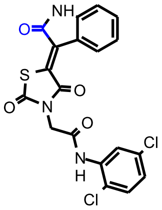 [*]C(=O)[*]	-0.352
ECFP_6	-817402818	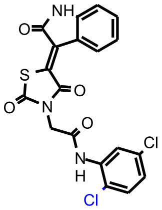 [*]Cl	-0.263
FCFP_6	566058135	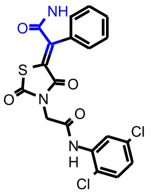 [*]NC(=O)C(=[*])[*]	-0.216

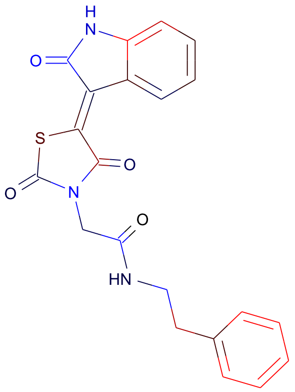

$C_{21}H_{17}N_{3}O_{4}S$
Molecular Weight: 407.44237
ALogP: 2.027
Rotatable Bonds: 5
Acceptors: 5
Donors: 2

Structural Similar Compounds

Name	PIRETANIDE	CARBAMIC ACID; [1-[(5-CYANOPENTYL)CARBAMOYL]BENZIMIDAZOL-2-YL]-; METHYL ESTER	PENICILLIN G; POTASSIUM SALT (K STRIPPED)
Structure	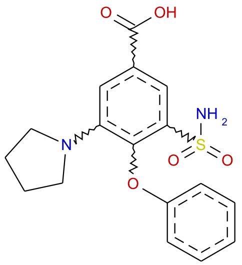	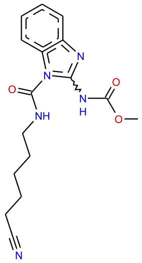	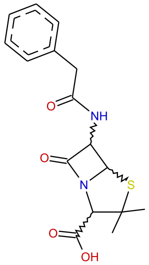
Actual Endpoint (-log C)	1.811	2.12	1.698
Predicted Endpoint (-log C)	1.83976	1.78415	1.80749
Distance	0.521	0.571	0.617
Reference	DRFUD4 2;393;77	85ARAE 4;118;76/77	AIPTAK 123;295;60

Model Prediction

Prediction: 1.93
Unit: g/kg_body_weight
Mahalanobis Distance: 23.3
Mahalanobis Distance p-value: 2.09e-022

Mahalanobis Distance: The Mahalanobis distance (MD) is a generalization of the Euclidean distance that accounts for correlations among the X properties. It is calculated as the distance to the center of the training data. The larger the MD, the less trustworthy the prediction.
Mahalanobis Distance p-value: The p-value gives the fraction of training data with an MD greater than or equal to the one for the given sample, assuming normally distributed data. The smaller the p-value, the less trustworthy the prediciton. For highly non-normal X properties (e.g., fingerprints), the MD p-value is wildly inaccurate.

Model Applicability

Unknown features are fingerprint features in the query molecule, but not found or appearing too infrequently in the training set.

1. All properties and OPS components are within expected ranges.
2. Unknown FCFP_6 feature: 16: [*]:[cH]:[*]
3. Unknown FCFP_6 feature: 1618154665: [*][c](:[*]):[cH]:[cH]:[*]
4. Unknown FCFP_6 feature: -1678275541: [*]C(=C1C(=[*])[*][*]:[c]1:[*])[*]

Feature Contribution

Top features for positive contribution

Fingerprint	Bit/Smiles	Feature Structure	Score
ECFP_6	642810091	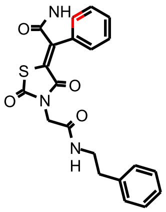 [*][c](:[*]):[*]	0.281

ECFP_6	-1897341097	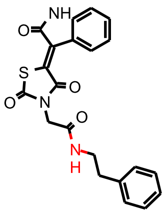 [*]N[*]	0.216
ECFP_6	1571214559	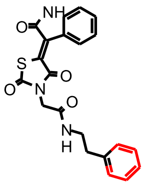 [*]1:[cH]:[cH]:[cH]:[cH]:[cH]:1	0.19

Top Features for negative contribution

Fingerprint	Bit/Smiles	Feature Structure	Score
ECFP_6	2106656448	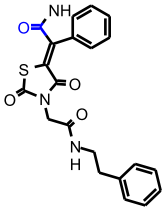 [*]C(=O)[*]	-0.352
ECFP_6	497523368	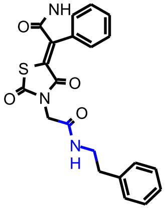 [*]CNC(=[*])[*]	-0.301
FCFP_6	566058135	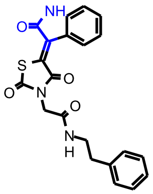 [*]NC(=O)C(=[*])[*]	-0.216

Sorafenib

TOPKAT_Rat_Oral_LD50

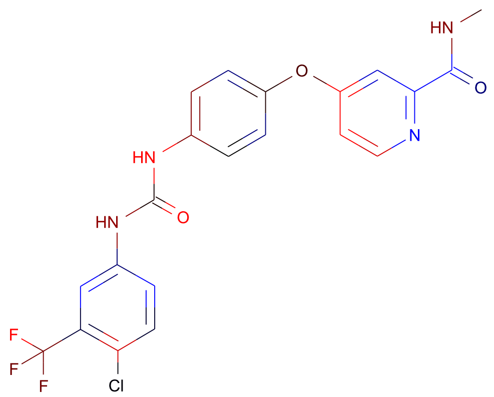

$C_{21}H_{16}ClF_3N_4O_3$
Molecular Weight: 464.82494
ALogP: 4.175
Rotatable Bonds: 6
Acceptors: 4
Donors: 3

Structural Similar Compounds

Name	FLUBENDAZOLE	PHOSPHORAMIDOTHIOIC ACID; ACETIMIDOYL-; O;O-bis-(p-CHLOROPHENYL)ESTER	BEZAFIBRATE
Structure	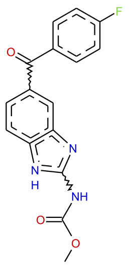	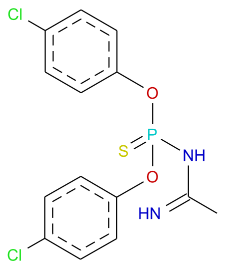	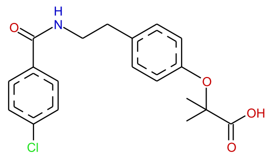
Actual Endpoint (-log C)	2.088	5.006	1.946
Predicted Endpoint (-log C)	2.69288	3.23989	2.54395
Distance	0.697	0.703	0.721
Reference	YRTMA6 9;11;78	FMCHA2 -;C149;89	ARZNAD 30;2023;80

Model Prediction

Prediction: 0.823
Unit: g/kg_body_weight
Mahalanobis Distance: 21
Mahalanobis Distance p-value: 1.93e-012

Mahalanobis Distance: The Mahalanobis distance (MD) is a generalization of the Euclidean distance that accounts for correlations among the X properties. It is calculated as the distance to the center of the training data. The larger the MD, the less trustworthy the prediction.
Mahalanobis Distance p-value: The p-value gives the fraction of training data with an MD greater than or equal to the one for the given sample, assuming normally distributed data. The smaller the p-value, the less trustworthy the prediciton. For highly non-normal X properties (e.g., fingerprints), the MD p-value is wildly inaccurate.

Model Applicability

Unknown features are fingerprint features in the query molecule, but not found or appearing too infrequently in the training set.

1. All properties and OPS components are within expected ranges.
2. Unknown FCFP_6 feature: 16: [*]:[cH]:[*]
3. Unknown FCFP_6 feature: 71476542: [*]:[c](:[*])Cl
4. Unknown FCFP_6 feature: 1747237384: [*][c](:[*]):n:[cH]:[*]
5. Unknown FCFP_6 feature: 1618154665: [*][c](:[*]):[cH]:[cH]:[*]
6. Unknown FCFP_6 feature: 136686699: [*]NC

Feature Contribution

Top features for positive contribution

Fingerprint	Bit/Smiles	Feature Structure	Score

FCFP_6	71953198	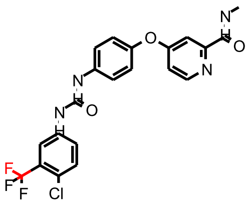 [*]C([*])([*])F	0.392
ECFP_6	-1046436026	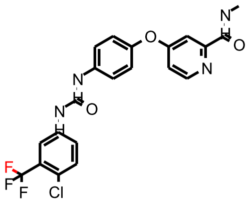 [*]F	0.349
ECFP_6	642810091	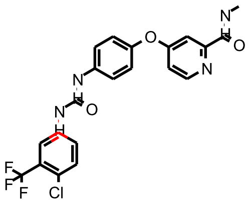 [*][c](:[*]):[*]	0.281
**Top Features for negative contribution**			
**Fingerprint**	**Bit/Smiles**	**Feature Structure**	**Score**
ECFP_6	226796801	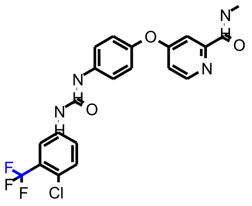 [*]C([*])([*])F	-0.32
ECFP_6	-817402818	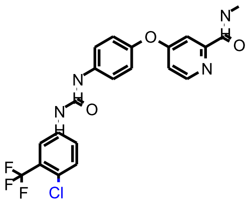 [*]Cl	-0.263

ECFP_6	-176455838	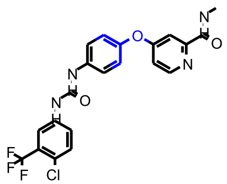 [*]O[c](:[cH]:[*]):[cH]:[*]	-0.257

Sunitinib

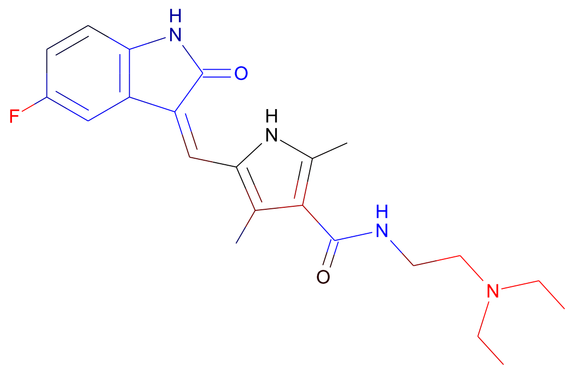

$C_{22}H_{27}FN_4O_2$
Molecular Weight: 398.47378
ALogP: 2.997
Rotatable Bonds: 7
Acceptors: 3
Donors: 3

Model Prediction

Prediction: 2.88
Unit: g/kg_body_weight
Mahalanobis Distance: 23.7
Mahalanobis Distance p-value: 3.38e-024

Mahalanobis Distance: The Mahalanobis distance (MD) is a generalization of the Euclidean distance that accounts for correlations among the X properties. It is calculated as the distance to the center of the training data. The larger the MD, the less trustworthy the prediction.
Mahalanobis Distance p-value: The p-value gives the fraction of training data with an MD greater than or equal to the one for the given sample, assuming normally distributed data. The smaller the p-value, the less trustworthy the prediciton. For highly non-normal X properties (e.g., fingerprints), the MD p-value is wildly inaccurate.

Structural Similar Compounds

Name	METHYLERGONOVINE	CLEBOPRIDE; MALATE SALT (MALATE STRIPPED)	DOMPERIDONE
Structure	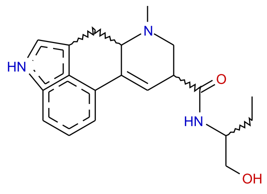	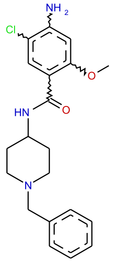	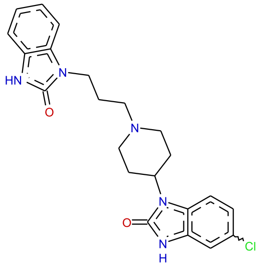
Actual Endpoint (-log C)	3.562	2.168	1.91
Predicted Endpoint (-log C)	2.94539	2.89926	2.49077
Distance	0.567	0.599	0.601
Reference	27ZIAQ -;163;73	OYYAA2 25;803;83	YACHDS 8;3991;80

Model Applicability

Unknown features are fingerprint features in the query molecule, but not found or appearing too infrequently in the training set.

1. All properties and OPS components are within expected ranges.
2. Unknown ECFP_2 feature: 980271847: [*][c]1:[*]:[*]:[nH]:[c]:1C=[*]
3. Unknown FCFP_6 feature: 19: [*]:[nH]:[*]
4. Unknown FCFP_6 feature: 16: [*]:[cH]:[*]
5. Unknown FCFP_6 feature: 71476542: [*]:[c](:[*])Cl
6. Unknown FCFP_6 feature: 2005402822: [*][c]1:[*]:[*]:[c]([*]):[nH]:1
7. Unknown FCFP_6 feature: 203707511: [*][c]1:[*]:[*]:[nH]:[c]:1C=[*]
8. Unknown FCFP_6 feature: -1678275541: [*]C(=C1C(=[*])[*][*]:[c]1:[*])[*]
9. Unknown FCFP_6 feature: 451371068: [*]C(=C[c](:[*]):[*])[*]
10. Unknown FCFP_6 feature: 1618154665: [*][c](:[*]):[cH]:[cH]:[*]

Feature Contribution

Top features for positive contribution			
Fingerprint	Bit/Smiles	Feature Structure	Score

ECFP_6	-1046436026	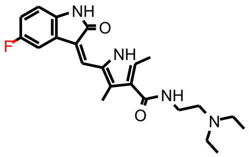 [*]F	0.349
ECFP_6	642810091	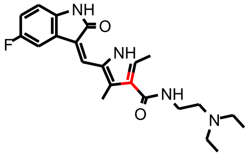 [*][c](:[*]):[*]	0.281
FCFP_6	1852108031	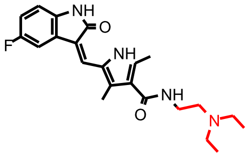 [*]CCN(CC)CC	0.226

Top Features for negative contribution

**Fingerprint**	**Bit/Smiles**	**Feature Structure**	**Score**
ECFP_6	2106656448	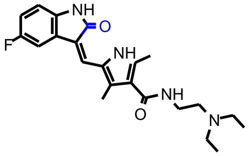 [*]C(=O)[*]	-0.352
ECFP_6	497523368	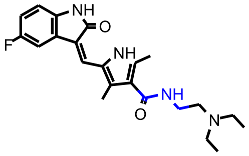 [*]CNC(=[*])[*]	-0.301

FCFP_6	566058135	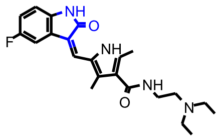<br>[*]NC(=O)C(=[*])[*]	-0.216
